# Supplementary material for: Influence of Substituents in a Six-Membered Chelate Ring of HG-Type Complexes Containing an N→Ru Bond on Their Stability and Catalytic Activity
Source: Molecules. 2023 Jan 25;28(3):1188. doi: 10.3390/molecules28031188 (PMC9921640; doi:10.3390/molecules28031188)

^{1}H NMR spectrum of compound **1a**

Acquisition Time (sec)	1.9818	***Comment***	single_pulse	***Date***			
Date Stamp							
File Name						***Frequency (MHz)***	600.17
Nucleus	1H	***Number of Transients***	8	***Origin***	ECA 600	***Original Points Count***	32768
Owner	delta	***Points Count***	32768	***Pulse Sequence***	single_pulse.ex2	***Receiver Gain***	22.00
Solvent	CHLOROFORM-d			***Spectrum Offset (Hz)***	5388.4248	***Sweep Width (Hz)***	16534.39
Temperature (degree C)	21.600						

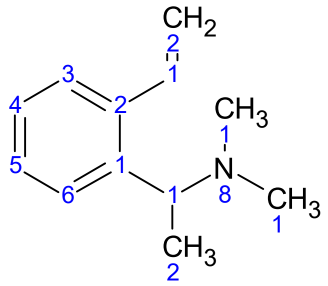

1a

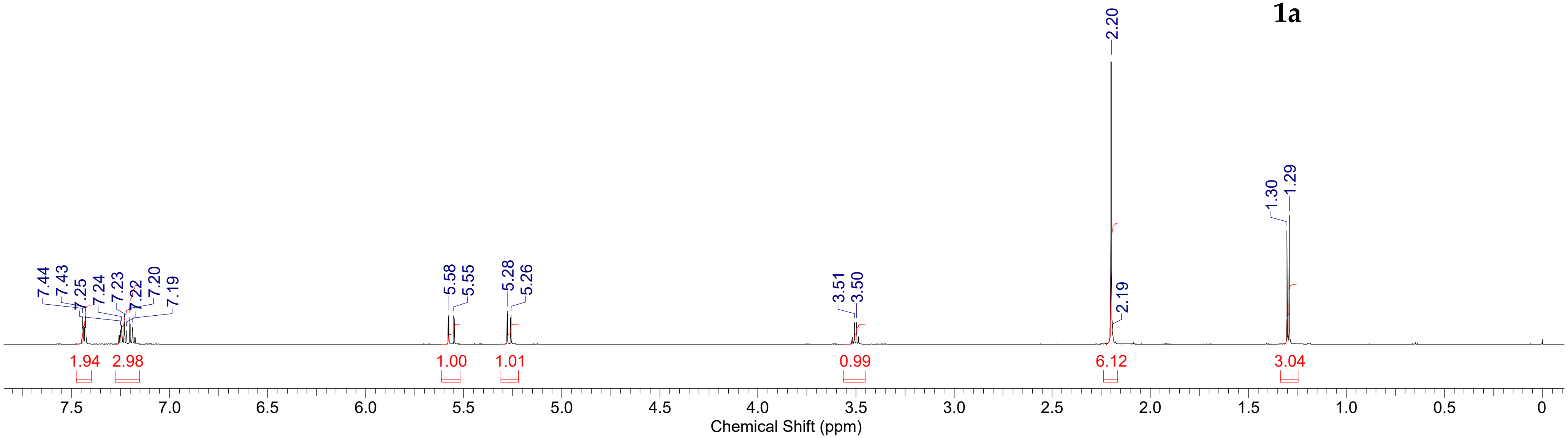

^{1}H NMR spectrum of compound **1a**

Acquisition Time (sec)	1.9818	***Comment***	single_pulse	***Date***			
Date Stamp							
File Name						***Frequency (MHz)***	600.17
Nucleus	1H	***Number of Transients***	8	***Origin***	ECA 600	***Original Points Count***	32768
Owner	delta	***Points Count***	32768	***Pulse Sequence***	single_pulse.ex2	***Receiver Gain***	22.00
Solvent	CHLOROFORM-d			***Spectrum Offset (Hz)***	5388.4248	***Sweep Width (Hz)***	16534.39
Temperature (degree C)	21.600						

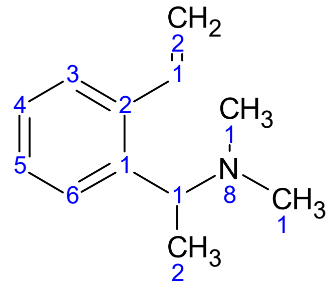

1a

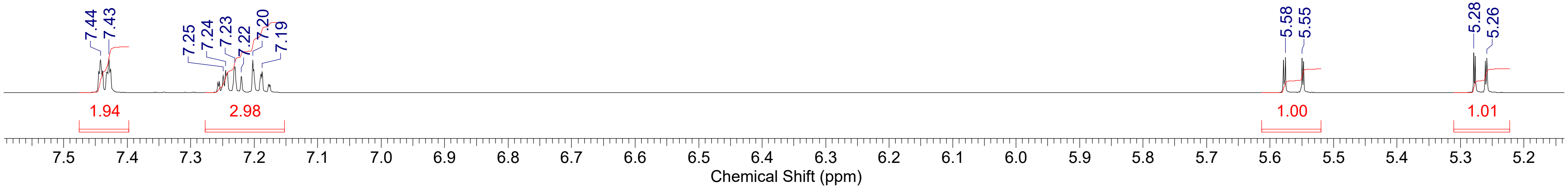

^{1}H NMR spectrum of compound **1a**

Acquisition Time (sec)	1.9818	***Comment***	single_pulse	***Date***			
Date Stamp							
File Name						***Frequency (MHz)***	600.17
Nucleus	1H	***Number of Transients***	8	***Origin***	ECA 600	***Original Points Count***	32768
Owner	delta	***Points Count***	32768	***Pulse Sequence***	single_pulse.ex2	***Receiver Gain***	22.00
Solvent	CHLOROFORM-d			***Spectrum Offset (Hz)***	5388.4248	***Sweep Width (Hz)***	16534.39
Temperature (degree C)	21.600						

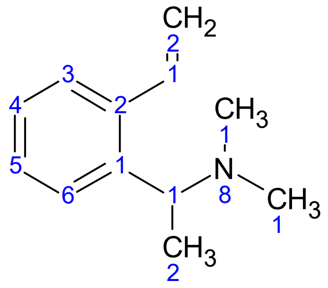

1a

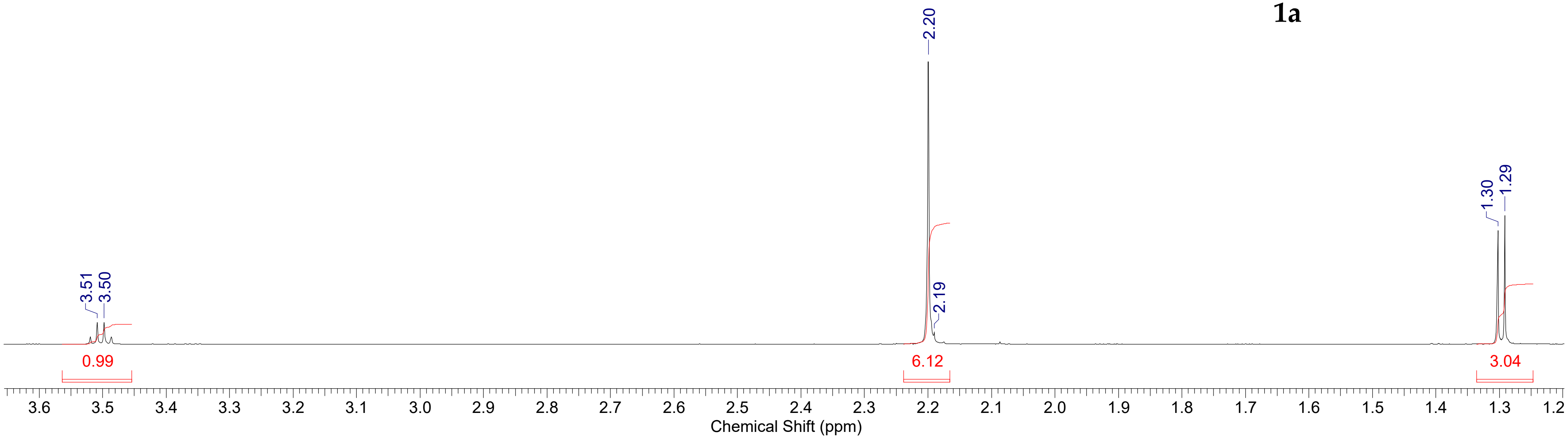

^{13}C NMR spectrum of compound **1a**

Acquisition Time (sec)	0.6921	***Comment***	single pulse decoupled gated NOE			***Date***	
Date Stamp							
File Name						***Frequency (MHz)***	150.91
Nucleus	13C	***Number of Transients***	81	***Origin***	ECA 600	***Original Points Count***	32768
Owner	delta	***Points Count***	32768	***Pulse Sequence***	single_pulse_dec	***Receiver Gain***	52.00
Solvent	CHLOROFORM-d	***Spectrum Offset (Hz)***	15091.3428	***Sweep Width (Hz)***	47348.49	***Temperature (degree C)***	22.900

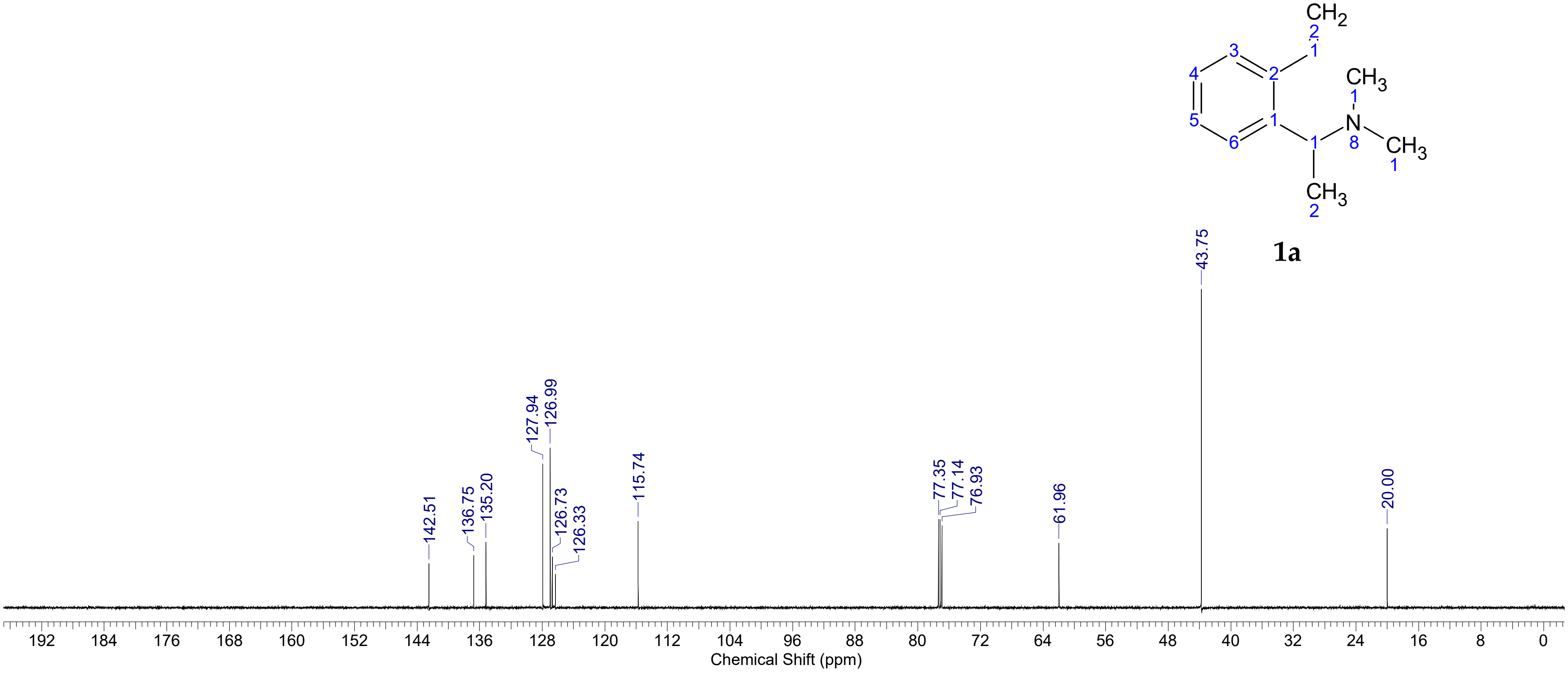

^{13}C NMR spectrum of compound **1a**

Acquisition Time (sec)	0.6921	***Comment***	single pulse decoupled gated NOE			***Date***	
Date Stamp							
File Name						***Frequency (MHz)***	150.91
Nucleus	13C	***Number of Transients***	81	***Origin***	ECA 600	***Original Points Count***	32768
Owner	delta	***Points Count***	32768	***Pulse Sequence***	single_pulse_dec	***Receiver Gain***	52.00
Solvent	CHLOROFORM-d	***Spectrum Offset (Hz)***	15091.3428	***Sweep Width (Hz)***	47348.49	***Temperature (degree C)***	22.900

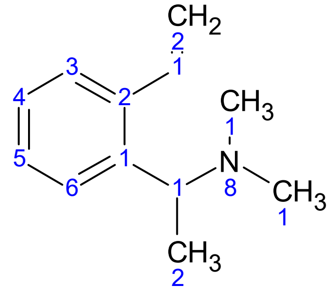

1a

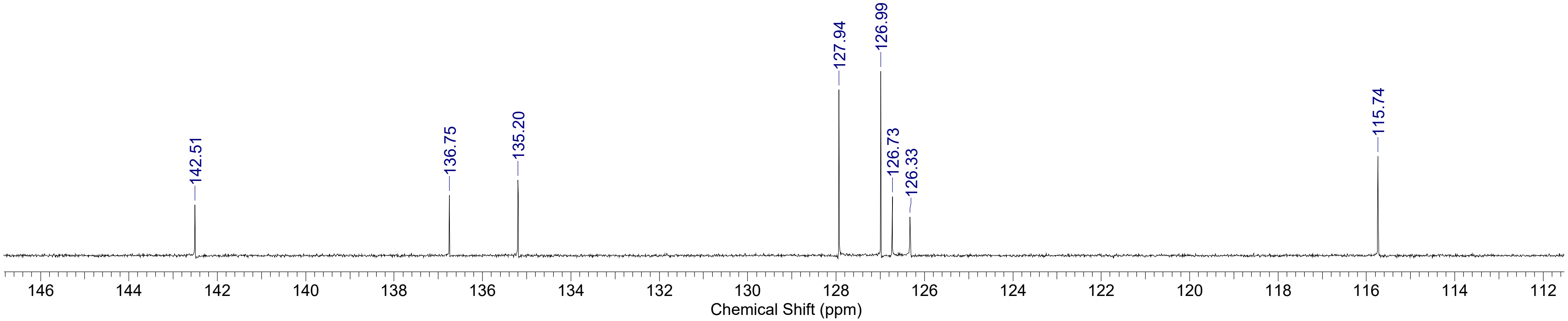

^{13}C NMR spectrum of compound **1a**

Acquisition Time (sec)	0.6921	***Comment***	single pulse decoupled gated NOE			***Date***	
Date Stamp							
File Name						***Frequency (MHz)***	150.91
Nucleus	13C	***Number of Transients***	81	***Origin***	ECA 600	***Original Points Count***	32768
Owner	delta	***Points Count***	32768	***Pulse Sequence***	single_pulse_dec	***Receiver Gain***	52.00
Solvent	CHLOROFORM-d	***Spectrum Offset (Hz)***	15091.3428	***Sweep Width (Hz)***	47348.49	***Temperature (degree C)***	22.900

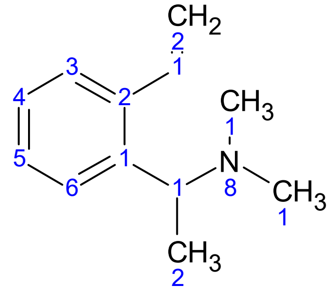

1a

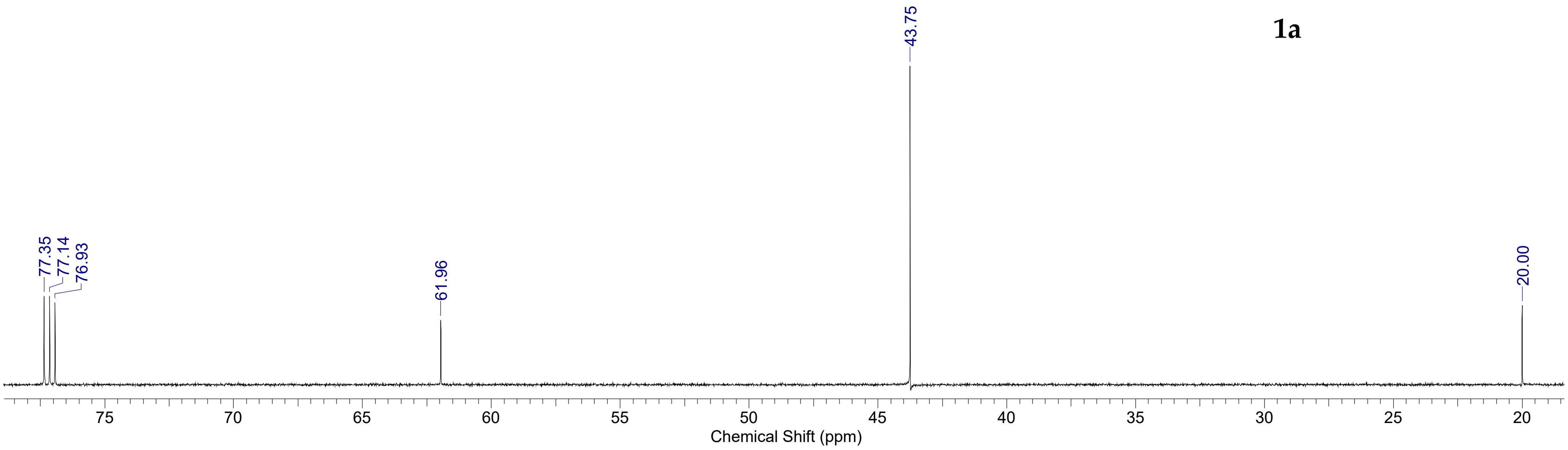

IR spectrum of compound **1a**

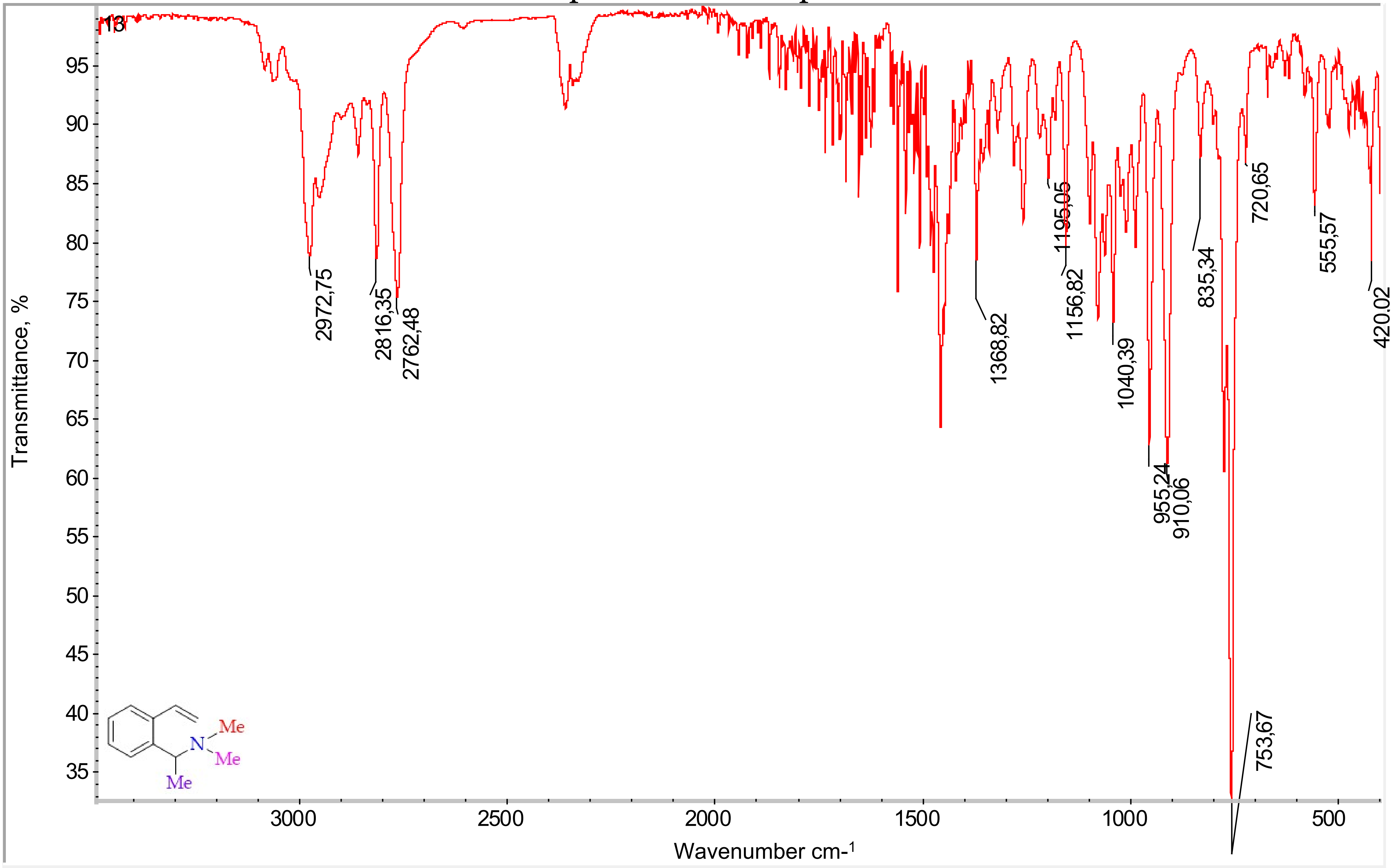

HRMS (MALDI-TOF) spectrum of compound **1a**

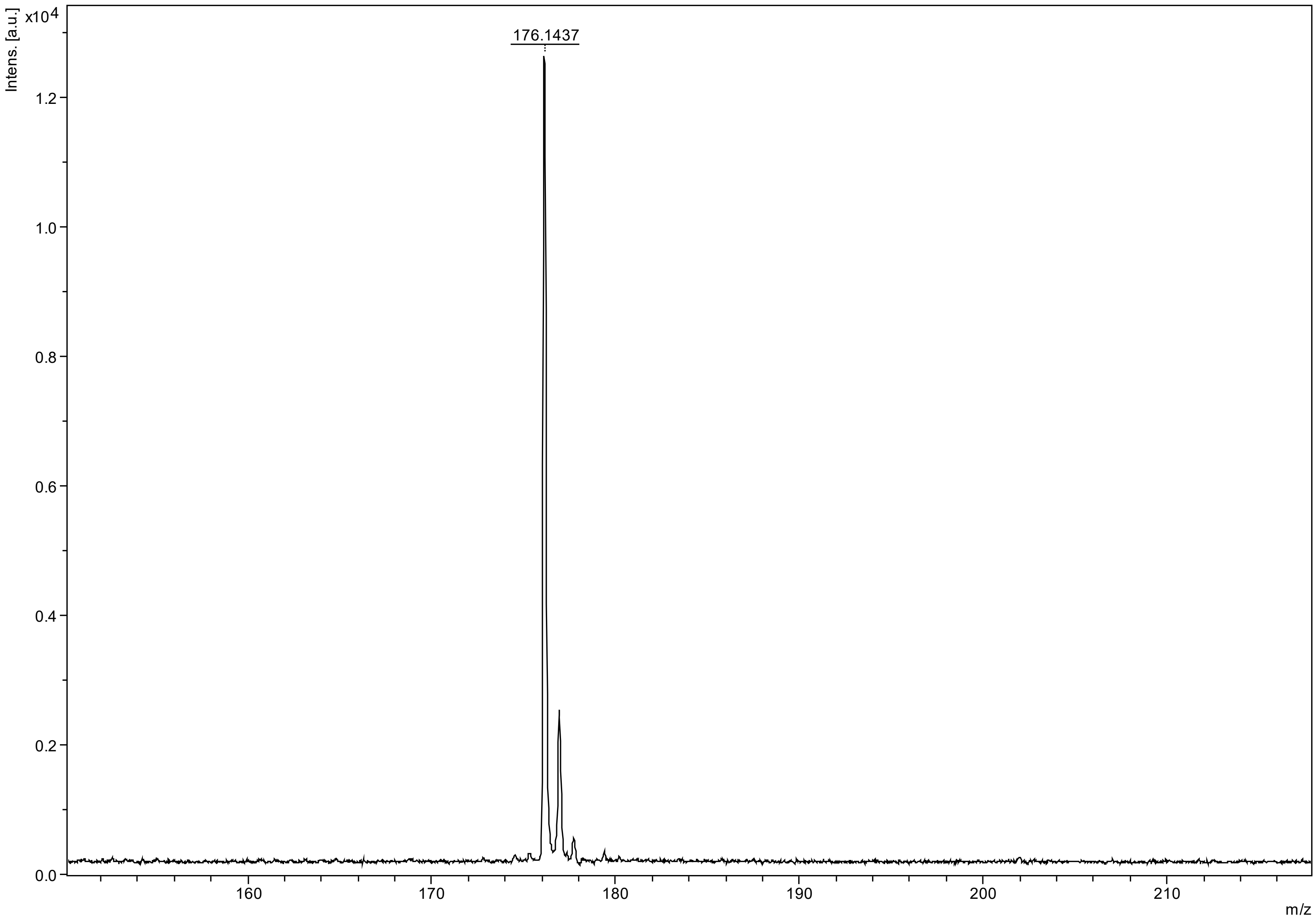

^{1}H NMR spectrum of compound **1b**

Acquisition Time (sec)	1.9818	Comment	single_pulse	Date	
Date Stamp					
File Name				Frequency (MHz)	600.17
Nucleus	1H	Number of Transients	8	Origin	ECA 600	Original Points Count	32768
Owner	delta	Points Count	32768	Pulse Sequence	single_pulse.ex2	Receiver Gain	30.00
Solvent	CHLOROFORM-d			Spectrum Offset (Hz)	5398.5166	Sweep Width (Hz)	16534.39
Temperature (degree C)	25.000						

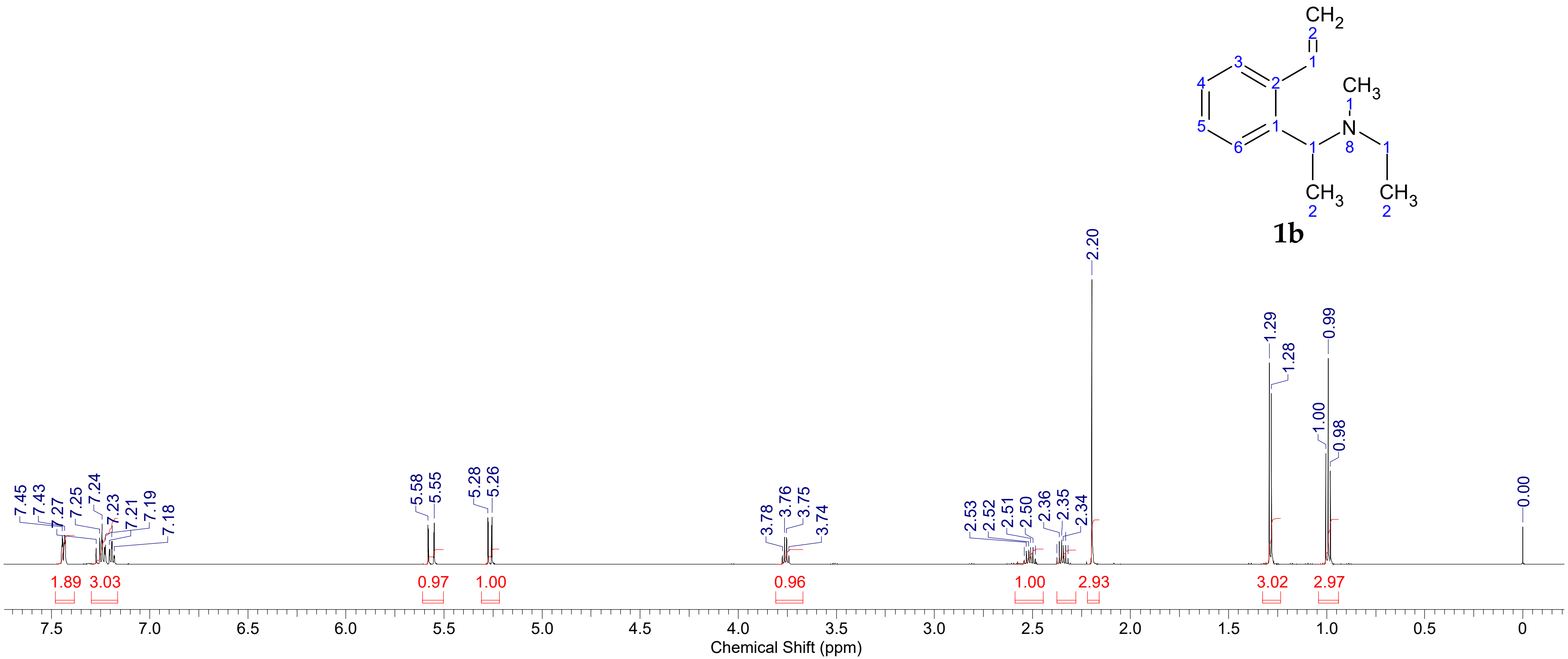

^{1}H NMR spectrum of compound **1b**

**Acquisition Time (sec)**	1.9818	**Comment**	single_pulse	**Date**			
**Date Stamp**							
**File Name**						**Frequency (MHz)**	600.17
**Nucleus**	1H	**Number of Transients**	8	**Origin**	ECA 600	**Original Points Count**	32768
**Owner**	delta	**Points Count**	32768	**Pulse Sequence**	single_pulse.ex2	**Receiver Gain**	30.00
**Solvent**	CHLOROFORM-d			**Spectrum Offset (Hz)**	5398.5166	**Sweep Width (Hz)**	16534.39
**Temperature (degree C)**	25.000						

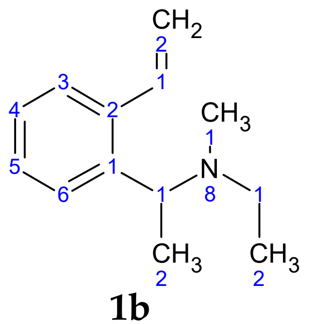

1b

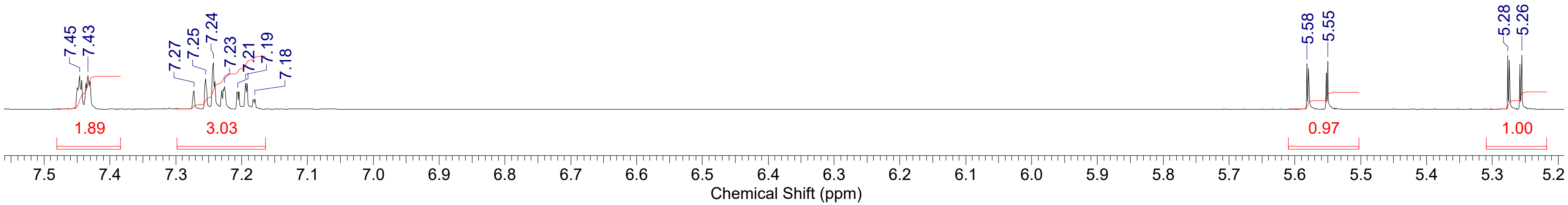

^{1}H NMR spectrum of compound **1b**

Acquisition Time (sec)	1.9818	***Comment***	single_pulse	***Date***			
Date Stamp							
File Name						***Frequency (MHz)***	600.17
Nucleus	1H	***Number of Transients***	8	***Origin***	ECA 600	***Original Points Count***	32768
Owner	delta	***Points Count***	32768	***Pulse Sequence***	single_pulse.ex2	***Receiver Gain***	30.00
Solvent	CHLOROFORM-d			***Spectrum Offset (Hz)***	5398.5166	***Sweep Width (Hz)***	16534.39
Temperature (degree C)	25.000						

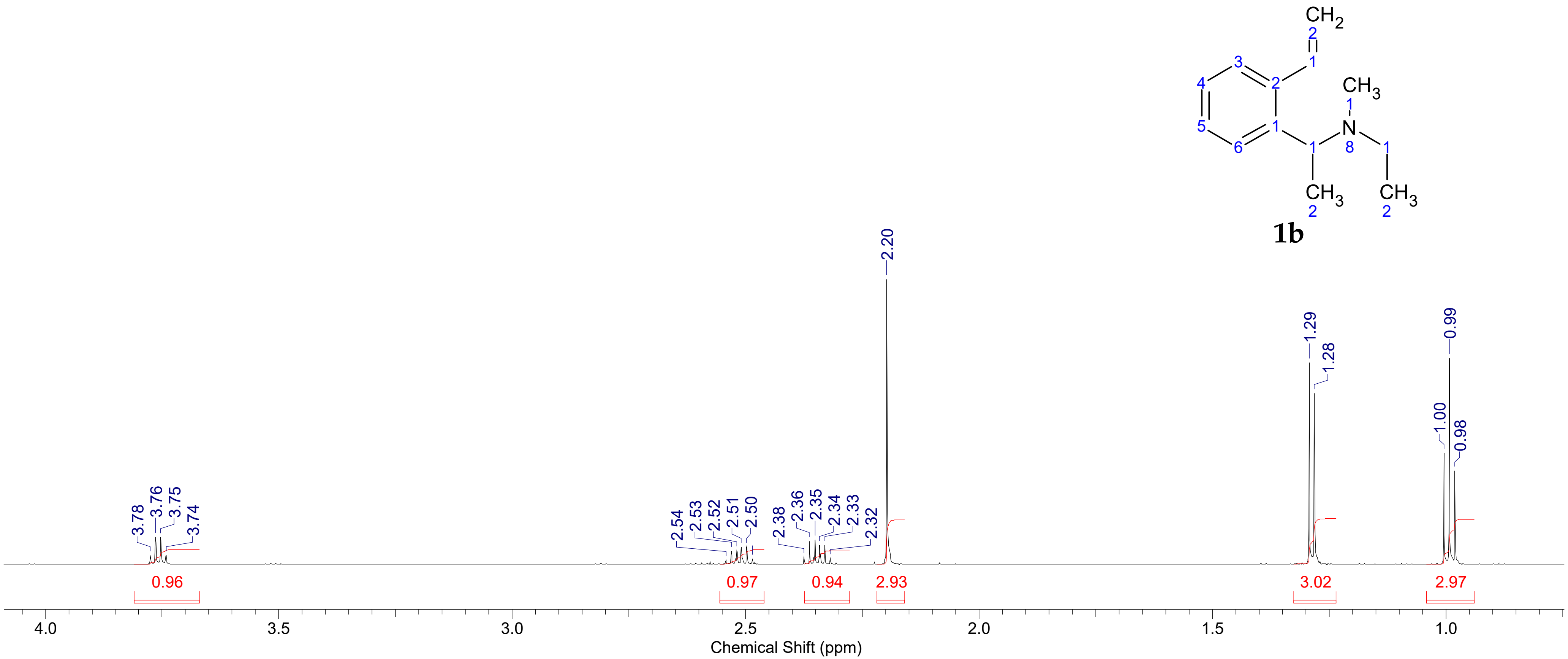

^{13}C NMR spectrum of compound **1b**

Acquisition Time (sec)	0.6921	***Comment***	single pulse decoupled gated NOE			***Date***	
Date Stamp							
File Name						***Frequency (MHz)***	150.91
Nucleus	13C	***Number of Transients***	301	***Origin***	ECA 600	***Original Points Count***	32768
Owner	delta	***Points Count***	32768	***Pulse Sequence***	single_pulse_dec	***Receiver Gain***	56.00
Solvent	CHLOROFORM-d	***Spectrum Offset (Hz)***	15091.3428	***Sweep Width (Hz)***	47348.49	***Temperature (degree C)***	22.400

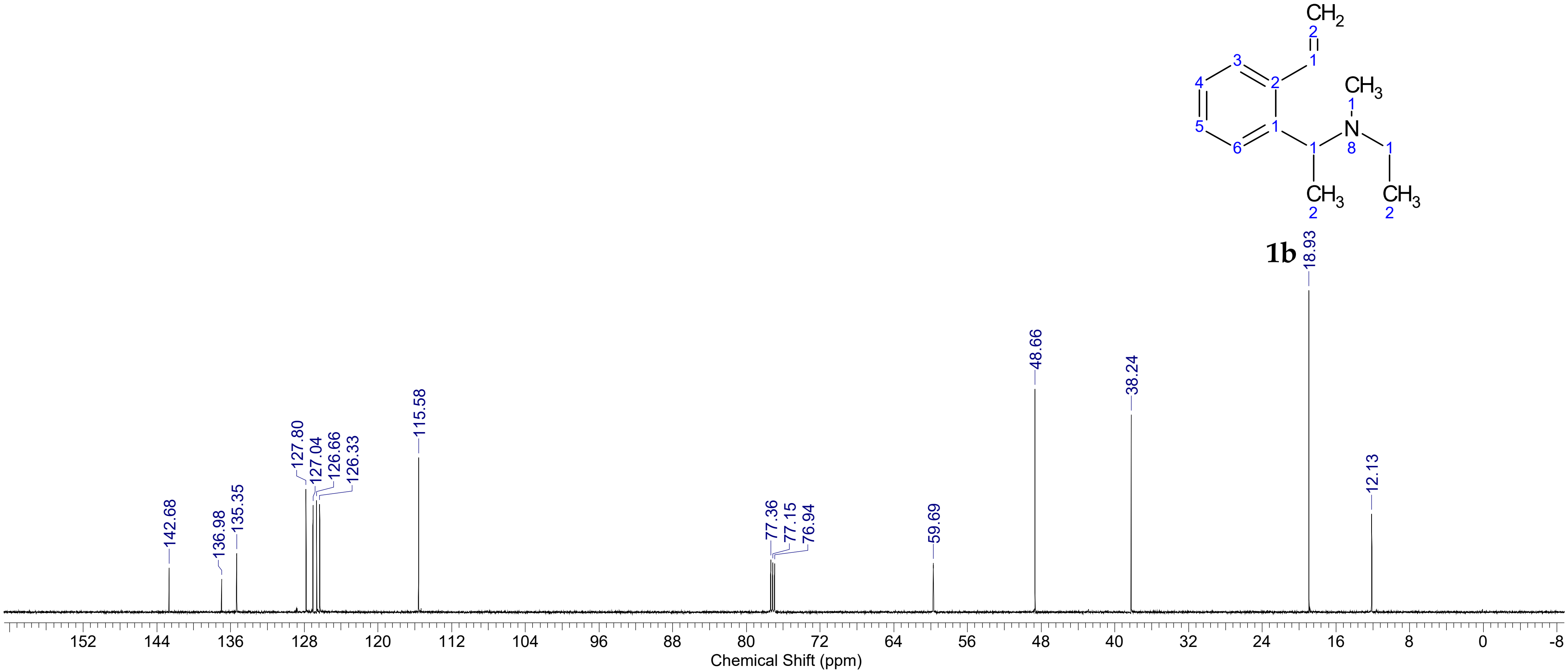

^{13}C NMR spectrum of compound **1b**

Acquisition Time (sec)	0.6921	***Comment***	single pulse decoupled gated NOE			***Date***	
Date Stamp							
File Name						***Frequency (MHz)***	150.91
Nucleus	13C	***Number of Transients***	301	***Origin***	ECA 600	***Original Points Count***	32768
Owner	delta	***Points Count***	32768	***Pulse Sequence***	single_pulse_dec	***Receiver Gain***	56.00
Solvent	CHLOROFORM-d	***Spectrum Offset (Hz)***	15091.3428	***Sweep Width (Hz)***	47348.49	***Temperature (degree C)***	22.400

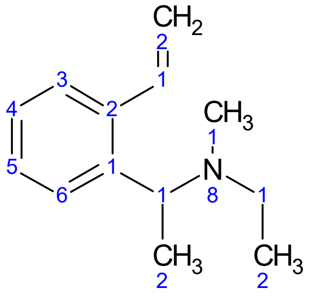

1b

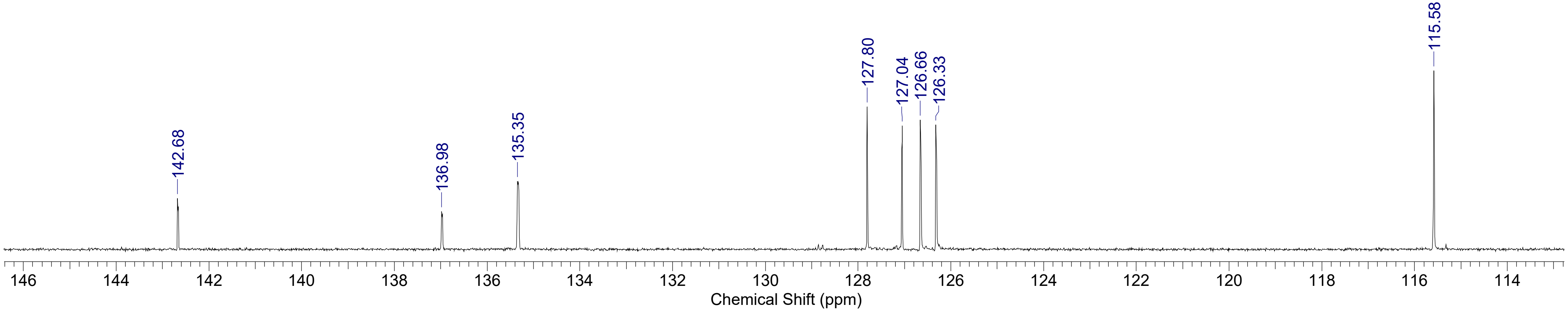

^{13}C NMR spectrum of compound **1b**

Acquisition Time (sec)	0.6921	***Comment***	single pulse decoupled gated NOE			***Date***	
Date Stamp							
File Name						***Frequency (MHz)***	150.91
Nucleus	13C	***Number of Transients***	301	***Origin***	ECA 600	***Original Points Count***	32768
Owner	delta	***Points Count***	32768	***Pulse Sequence***	single_pulse_dec	***Receiver Gain***	56.00
Solvent	CHLOROFORM-d	***Spectrum Offset (Hz)***	15091.3428	***Sweep Width (Hz)***	47348.49	***Temperature (degree C)***	22.400

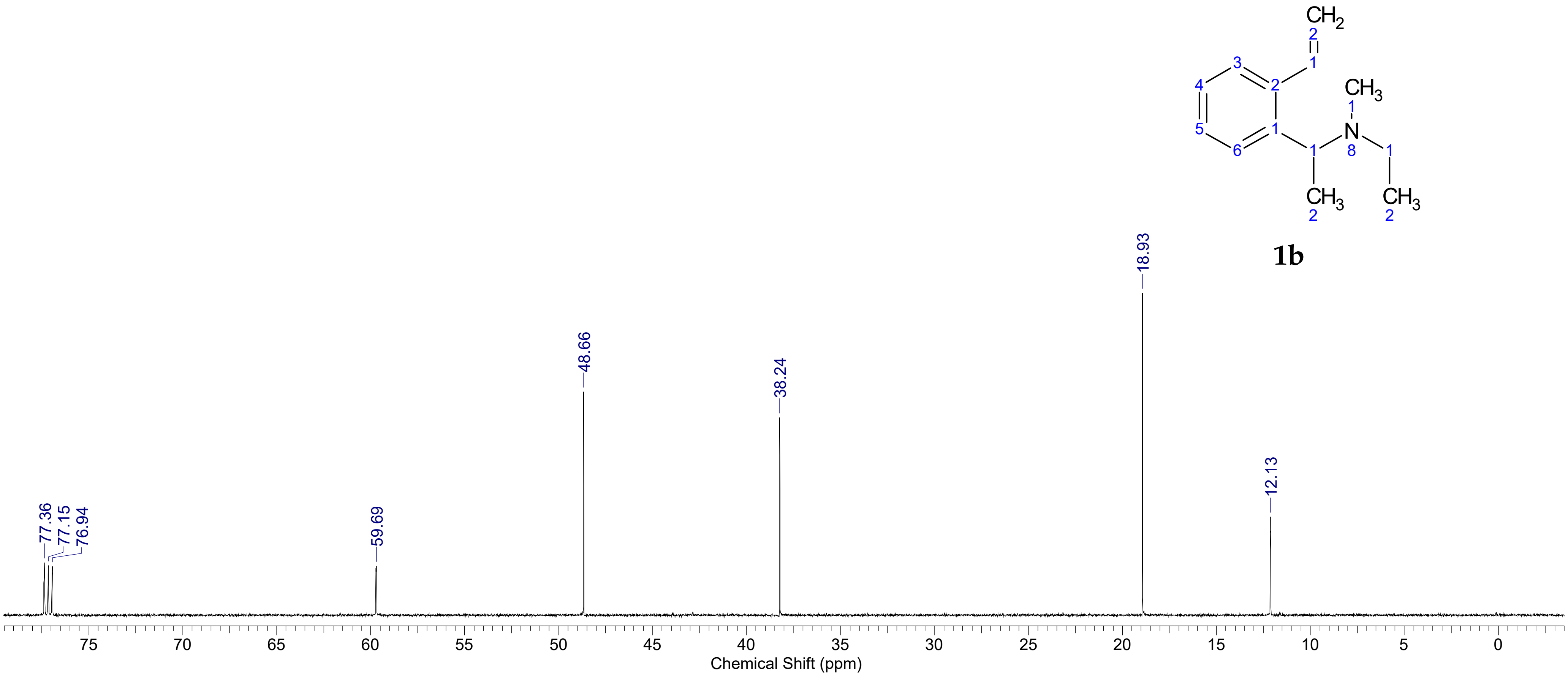

HRMS (MALDI-TOF) spectrum of compound **1b**

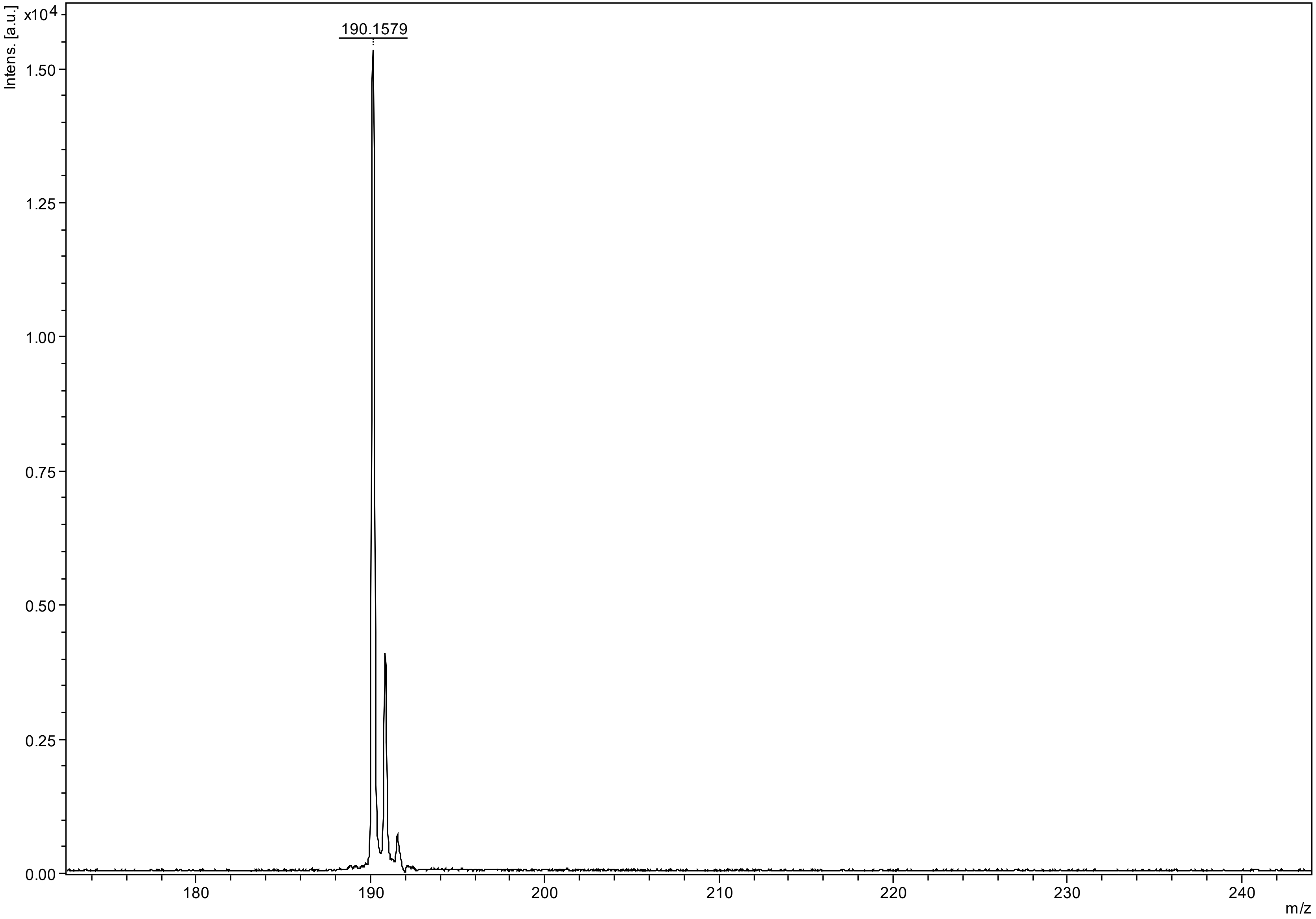

^{1}H NMR spectrum of compound **1c**

Acquisition Time (sec)	2.0972	Date				Date Stamp	
File Name							
Frequency (MHz)	700.17	Nucleus	1H	Number of Transients	8	Origin	Avance
Original Points Count	32768	Owner	nmr	Points Count	65536	Pulse Sequence	zg30
Receiver Gain	17.21	SW(cyclical) (Hz)	15625.00	Solvent	CHLOROFORM-d		
Spectrum Offset (Hz)	6272.3657	Sweep Width (Hz)	15624.76	Temperature (degree C)	24.988		

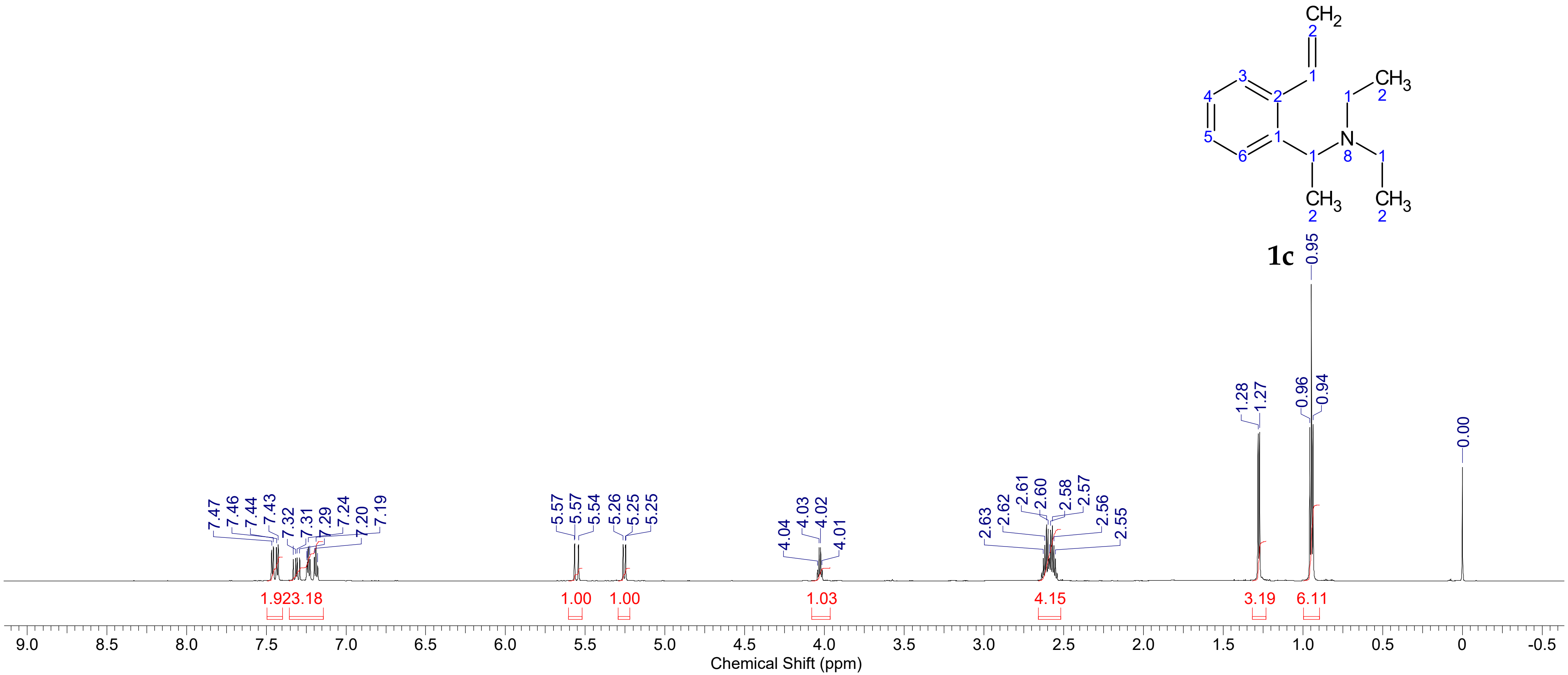

^{1}H NMR spectrum of compound **1c**

Acquisition Time (sec)	2.0972	Date				Date Stamp	
File Name							
Frequency (MHz)	700.17	Nucleus	1H	Number of Transients	8	Origin	Avance
Original Points Count	32768	Owner	nmr	Points Count	65536	Pulse Sequence	zg30
Receiver Gain	17.21	SW(cyclical) (Hz)	15625.00	Solvent	CHLOROFORM-d		
Spectrum Offset (Hz)	6272.3657	Sweep Width (Hz)	15624.76	Temperature (degree C)	24.988		

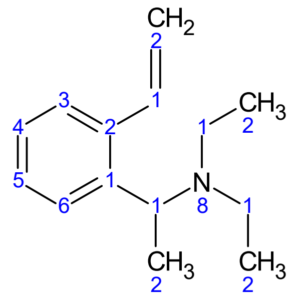

1c

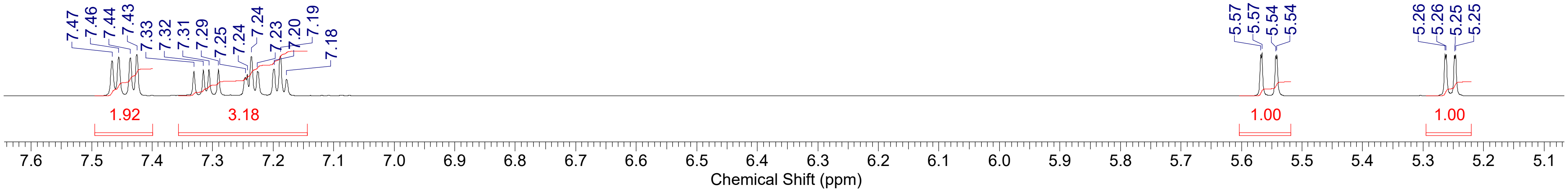

^{1}H NMR spectrum of compound **1c**

Acquisition Time (sec)	2.0972	Date				Date Stamp	
File Name							
Frequency (MHz)	700.17	Nucleus	1H	Number of Transients	8	Origin	Avance
Original Points Count	32768	Owner	nmr	Points Count	65536	Pulse Sequence	zg30
Receiver Gain	17.21	SW(cyclical) (Hz)	15625.00	Solvent	CHLOROFORM-d		
Spectrum Offset (Hz)	6272.3657	Sweep Width (Hz)	15624.76	Temperature (degree C)	24.988		

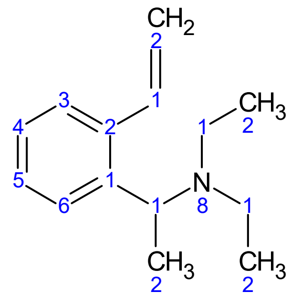

1c

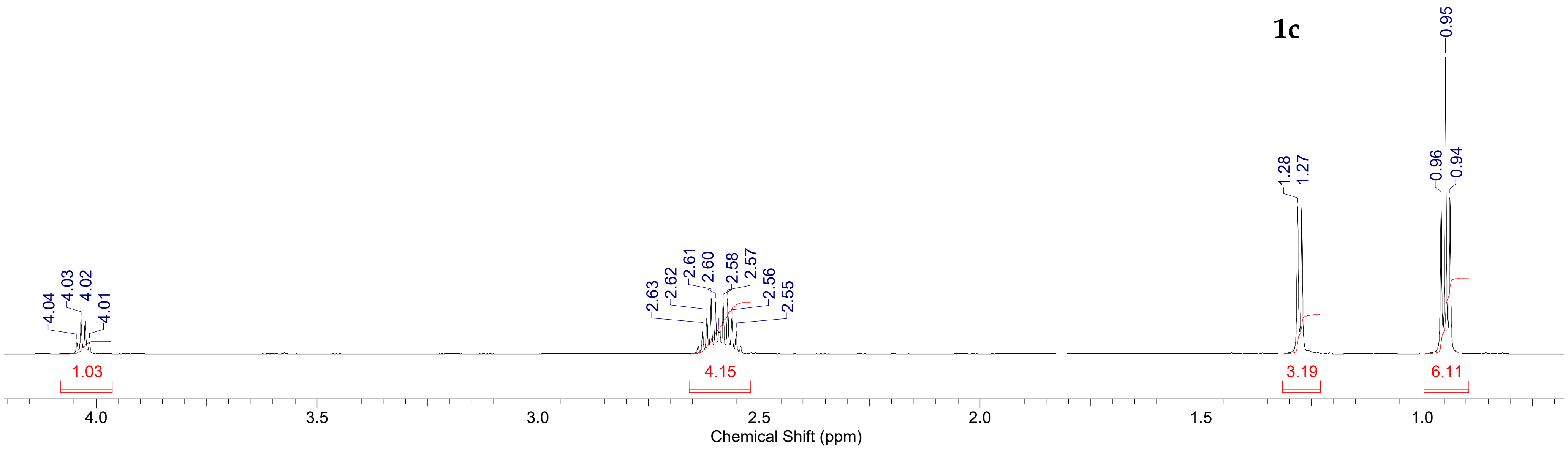

^{13}C NMR spectrum of compound **1c**

Acquisition Time (sec)	0.7406	Date				Date Stamp	
File Name							
Frequency (MHz)	176.06	Nucleus	13C	Number of Transients	400	Origin	Avance
Original Points Count	32768	Owner	nmr	Points Count	32768	Pulse Sequence	zgpg30
Receiver Gain	101.00	SW(cyclical) (Hz)	44247.79	Solvent	CHLOROFORM-d	Spectrum Offset (Hz)	19362.2637
Sweep Width (Hz)	44246.44	Temperature (degree C)	24.995				

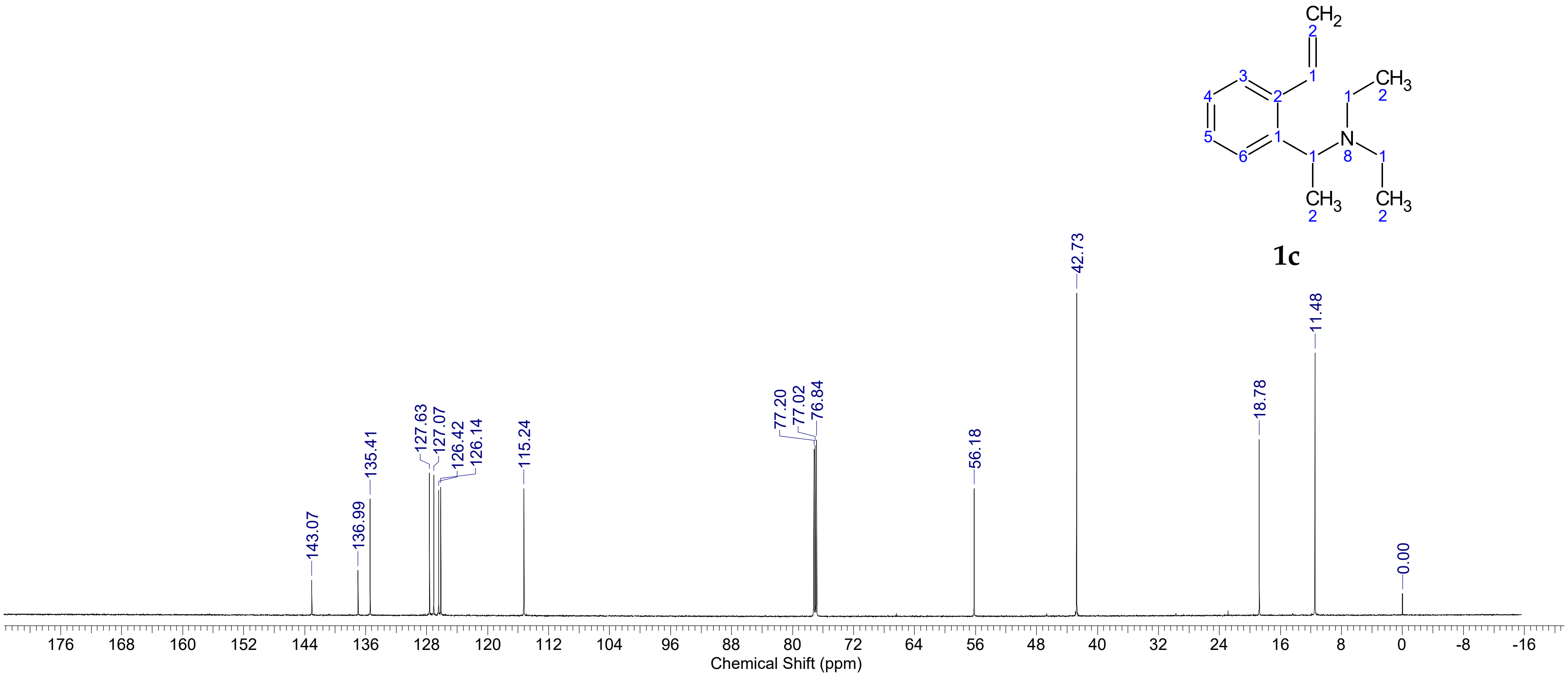

^{13}C NMR spectrum of compound **1c**

Acquisition Time (sec)	0.7406	Date				Date Stamp	
File Name							
Frequency (MHz)	176.06	Nucleus	13C	Number of Transients	400	Origin	Avance
Original Points Count	32768	Owner	nmr	Points Count	32768	Pulse Sequence	zgpg30
Receiver Gain	101.00	SW(cyclical) (Hz)	44247.79	Solvent	CHLOROFORM-d	Spectrum Offset (Hz)	19362.2637
Sweep Width (Hz)	44246.44	Temperature (degree C)	24.995				

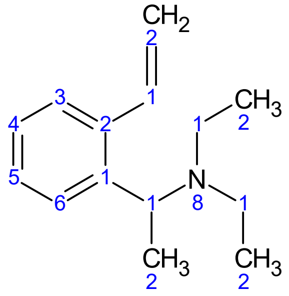

1c

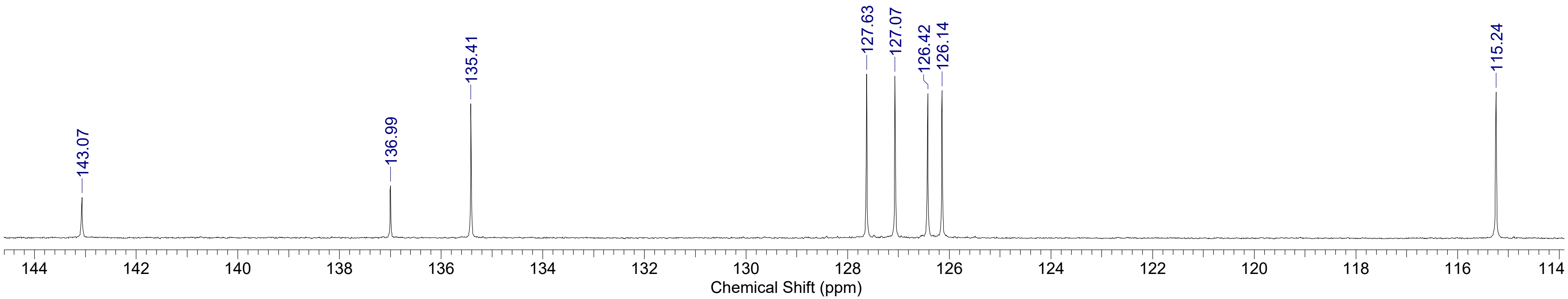

^{13}C NMR spectrum of compound **1c**

Acquisition Time (sec)	0.7406	***Date***				***Date Stamp***	
File Name							
Frequency (MHz)	176.06	***Nucleus***	13C	***Number of Transients***	400	***Origin***	Avance
Original Points Count	32768	***Owner***	nmr	***Points Count***	32768	***Pulse Sequence***	zgpg30
Receiver Gain	101.00	***SW(cyclical) (Hz)***	44247.79	***Solvent***	CHLOROFORM-d	***Spectrum Offset (Hz)***	19362.2637
Sweep Width (Hz)	44246.44	***Temperature (degree C)***	24.995				

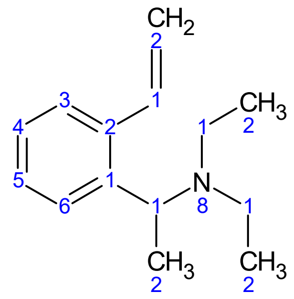

1c

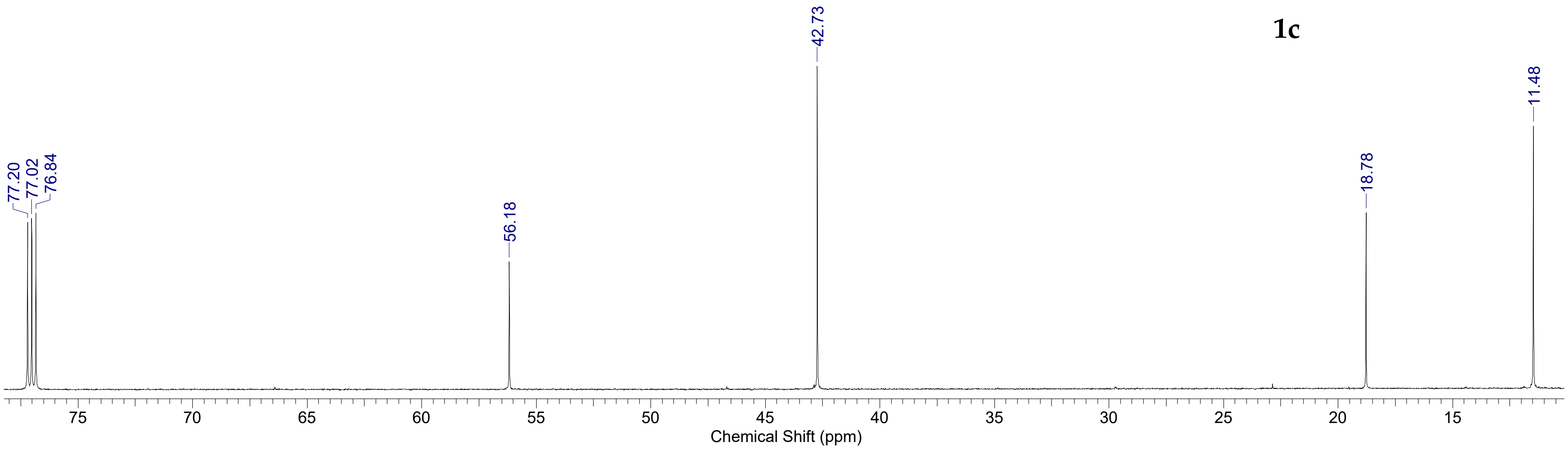

IR spectrum of compound **1c**

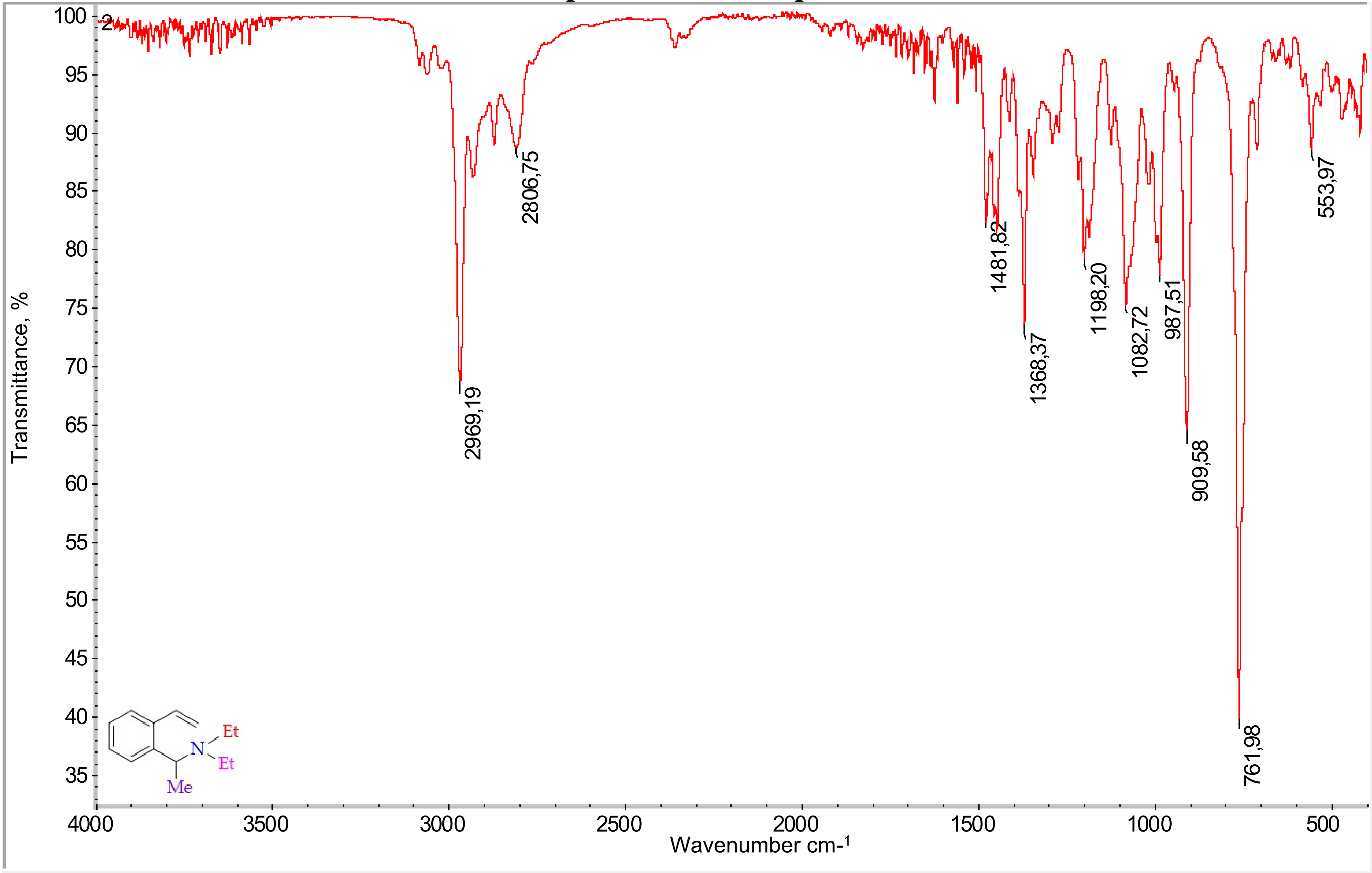

HRMS (MALDI-TOF) spectrum of compound **1c**

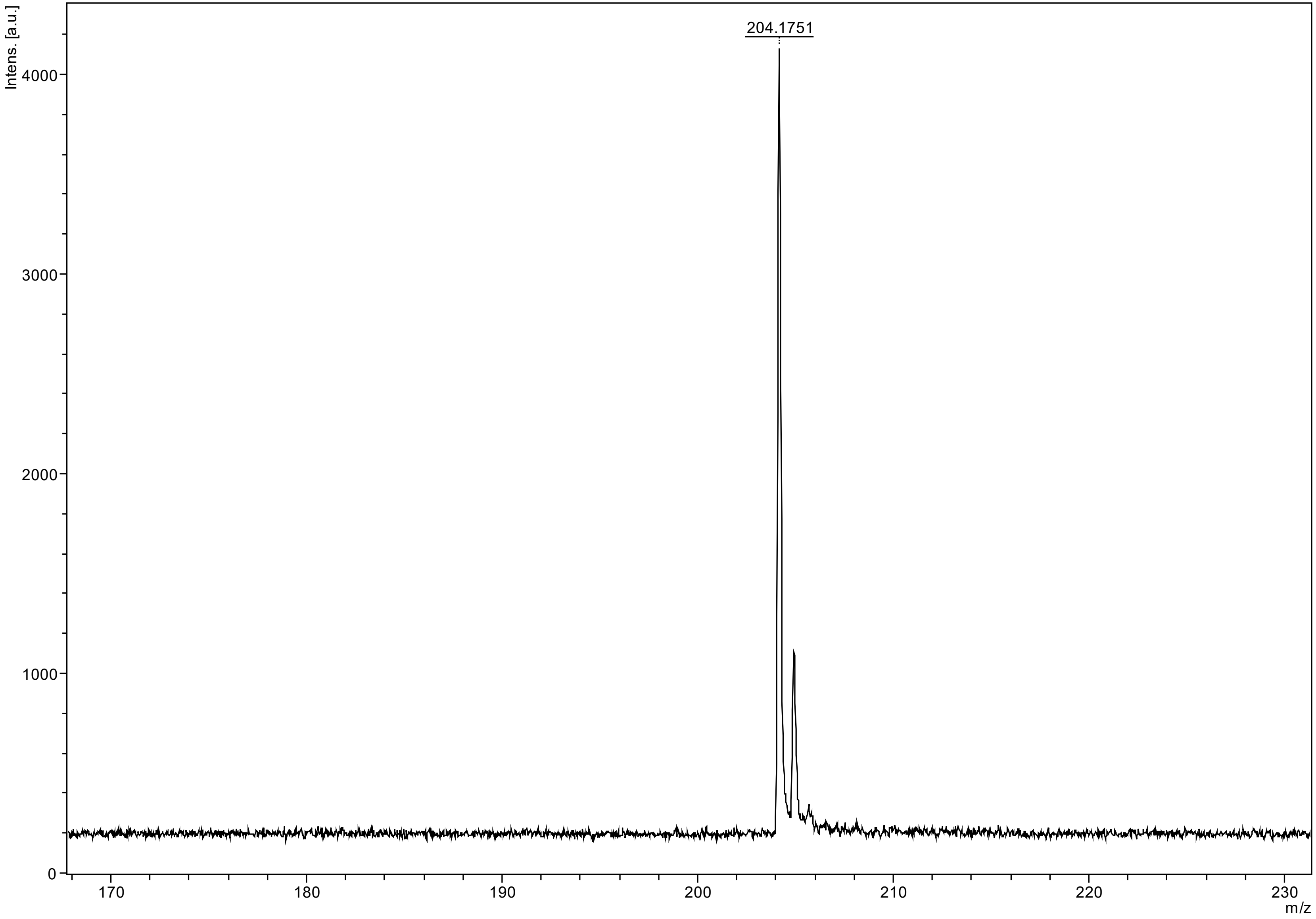

^{1}H NMR spectrum of compound **1d**

Acquisition Time (sec)	1.9818	***Comment***	single_pulse	***Date***			
Date Stamp							
File Name						***Frequency (MHz)***	600.17
Nucleus	1H	***Number of Transients***	8	***Origin***	ECA 600	***Original Points Count***	32768
Owner	CKP	***Points Count***	32768	***Pulse Sequence***	single_pulse.ex2	***Receiver Gain***	24.00
Solvent	CHLOROFORM-d	***Spectrum Offset (Hz)***	5394.4800	***Sweep Width (Hz)***	16534.39		

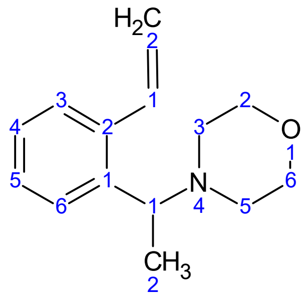

1d

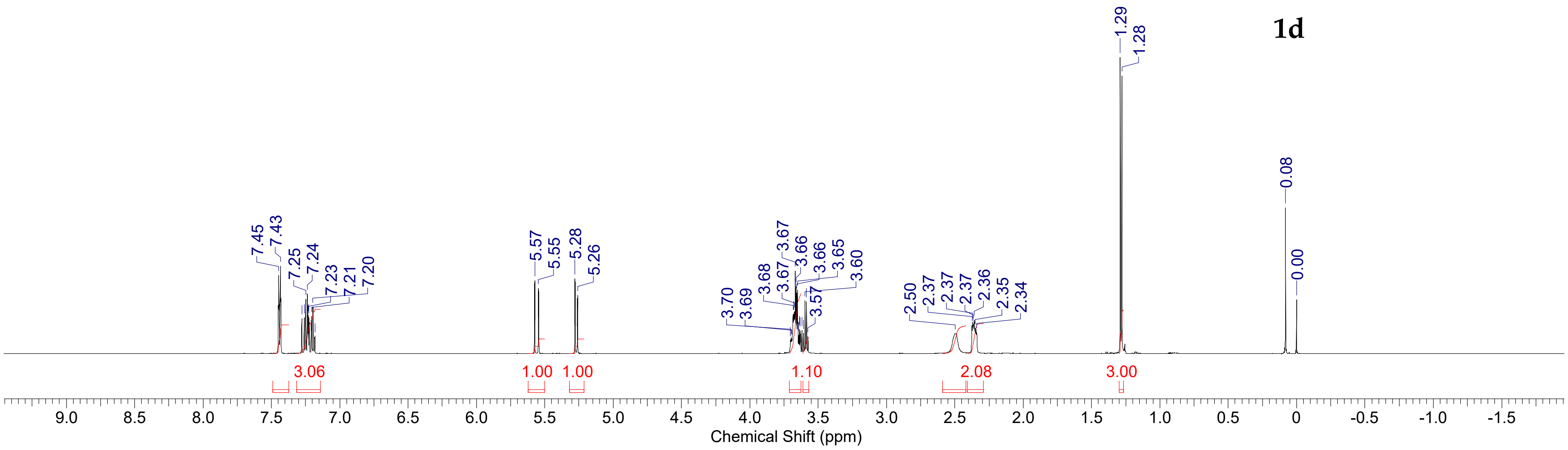

^{1}H NMR spectrum of compound **1d**

Acquisition Time (sec)	1.9818	***Comment***	single_pulse	***Date***			
Date Stamp							
File Name						***Frequency (MHz)***	600.17
Nucleus	1H	***Number of Transients***	8	***Origin***	ECA 600	***Original Points Count***	32768
Owner	CKP	***Points Count***	32768	***Pulse Sequence***	single_pulse.ex2	***Receiver Gain***	24.00
Solvent	CHLOROFORM-d	***Spectrum Offset (Hz)***	5394.4800	***Sweep Width (Hz)***	16534.39		

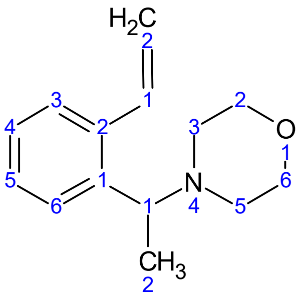

1d

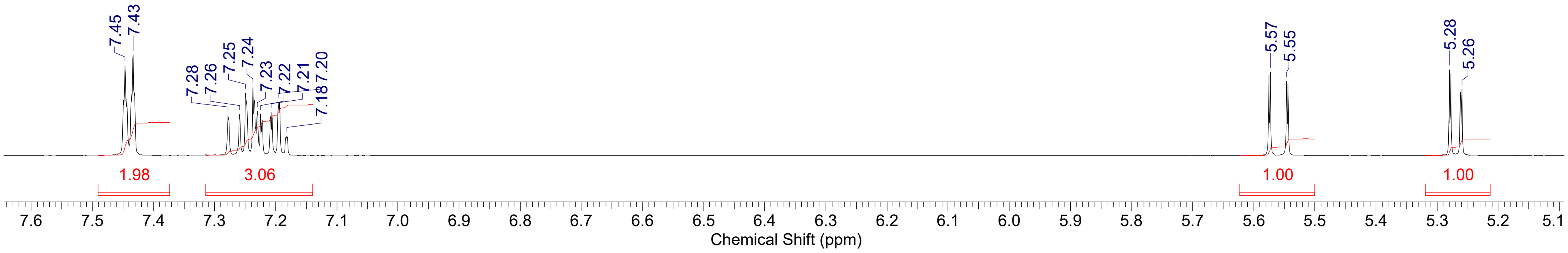

^{1}H NMR spectrum of compound **1d**

Acquisition Time (sec)	1.9818	***Comment***	single_pulse	***Date***			
Date Stamp							
File Name						***Frequency (MHz)***	600.17
Nucleus	1H	***Number of Transients***	8	***Origin***	ECA 600	***Original Points Count***	32768
Owner	CKP	***Points Count***	32768	***Pulse Sequence***	single_pulse.ex2	***Receiver Gain***	24.00
Solvent	CHLOROFORM-d	***Spectrum Offset (Hz)***	5394.4800	***Sweep Width (Hz)***	16534.39		

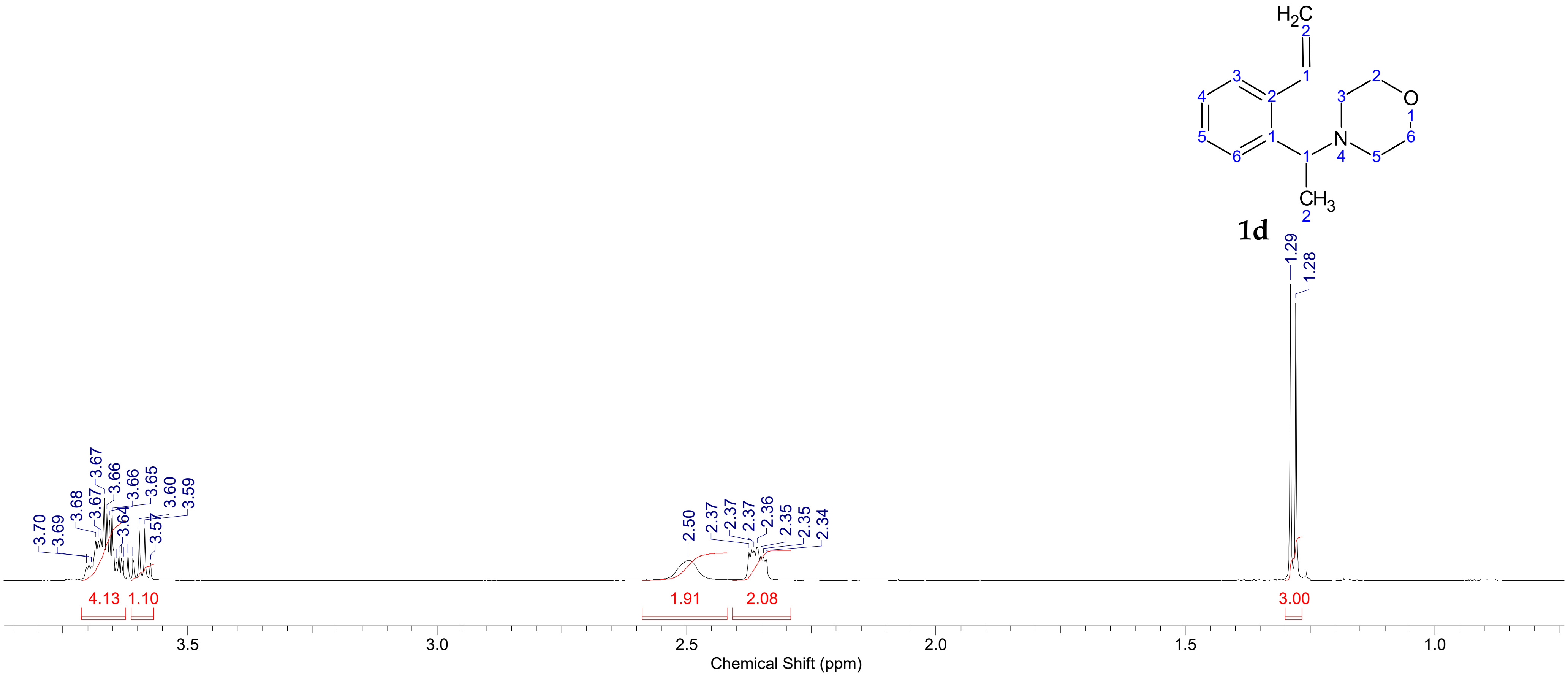

^{13}C NMR spectrum of compound **1d**

**Acquisition Time (sec)**	0.6921	**Comment**	single pulse decoupled gated NOE			**Date**	
**Date Stamp**							
**File Name**						**Frequency (MHz)**	150.91
**Nucleus**	13C	**Number of Transients**	1000	**Origin**	ECA 600	**Original Points Count**	32768
**Owner**	CKP	**Points Count**	32768	**Pulse Sequence**	single_pulse_dec	**Receiver Gain**	58.00
**Solvent**	CHLOROFORM-d	**Spectrum Offset (Hz)**	15072.1279	**Sweep Width (Hz)**	47348.49		

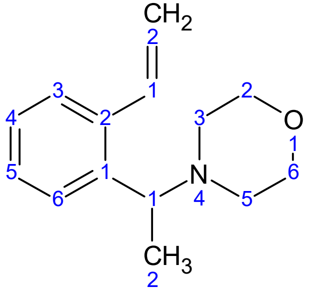

1d

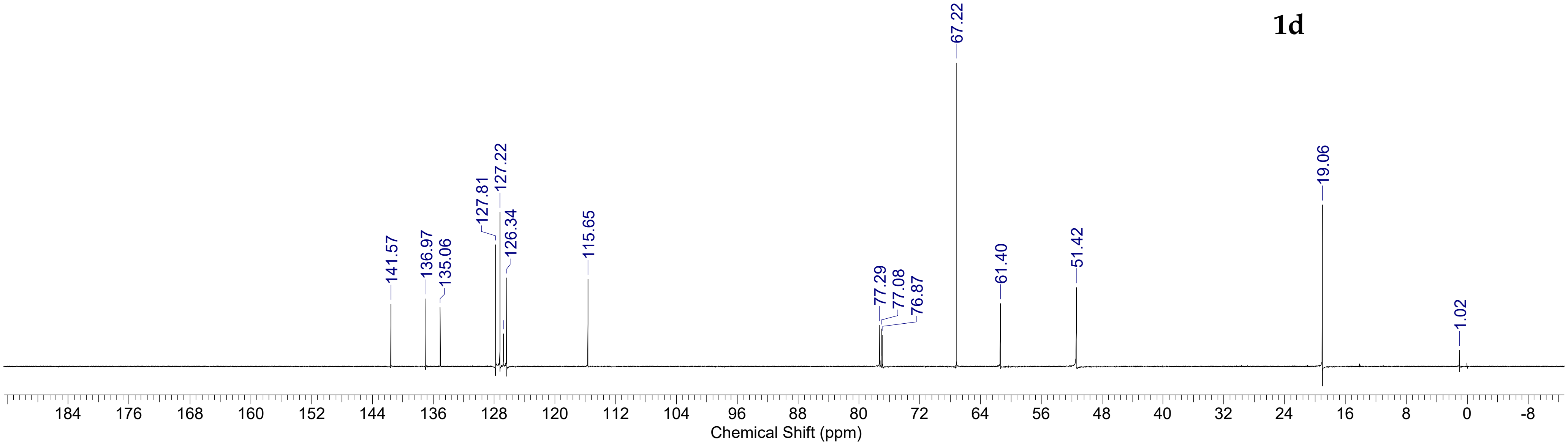

^{13}C NMR spectrum of compound **1d**

Acquisition Time (sec)	0.6921	***Comment***	single pulse decoupled gated NOE			***Date***	
Date Stamp							
File Name						***Frequency (MHz)***	150.91
Nucleus	13C	***Number of Transients***	1000	***Origin***	ECA 600	***Original Points Count***	32768
Owner	CKP	***Points Count***	32768	***Pulse Sequence***	single_pulse_dec	***Receiver Gain***	58.00
Solvent	CHLOROFORM-d	***Spectrum Offset (Hz)***	15072.1279	***Sweep Width (Hz)***	47348.49		

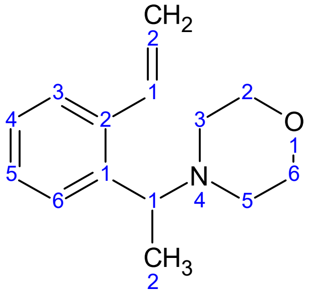

1d

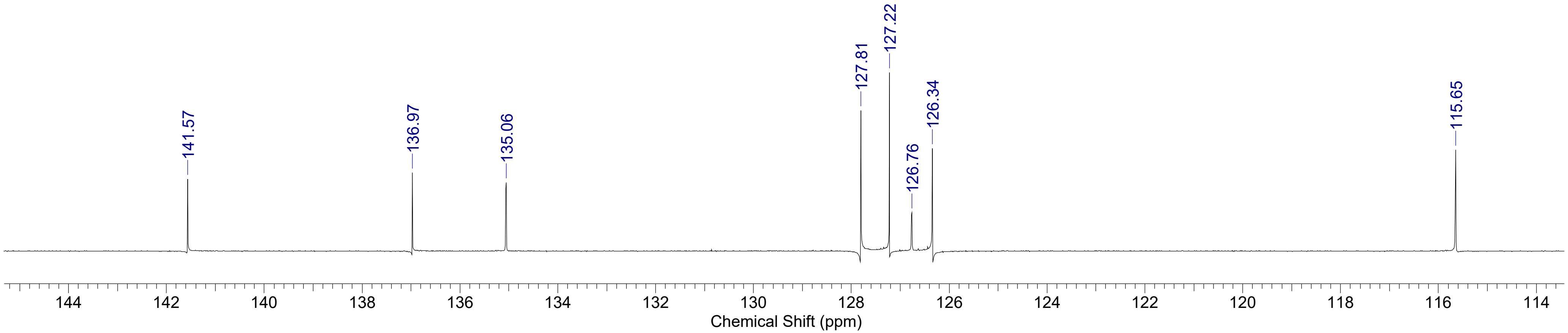

^{13}C NMR spectrum of compound **1d**

Acquisition Time (sec) 0.6921		***Comment***	single pulse decoupled gated NOE			***Date***	
Date Stamp							
File Name						***Frequency (MHz)***	150.91
Nucleus	13C	***Number of Transients***	1000	***Origin***	ECA 600	***Original Points Count***	32768
Owner	CKP	***Points Count***	32768	***Pulse Sequence***	single_pulse_dec	***Receiver Gain***	58.00
Solvent	CHLOROFORM-d	***Spectrum Offset (Hz)***	15072.1279	***Sweep Width (Hz)***	47348.49		

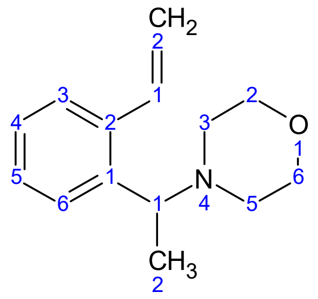

1d

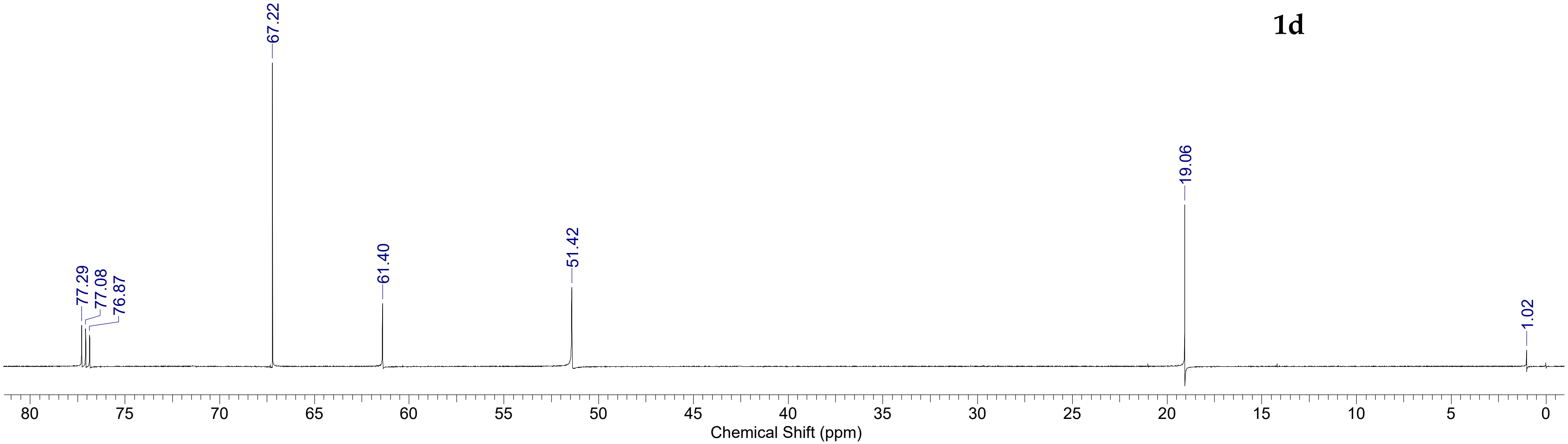

IR spectrum of compound **1d**

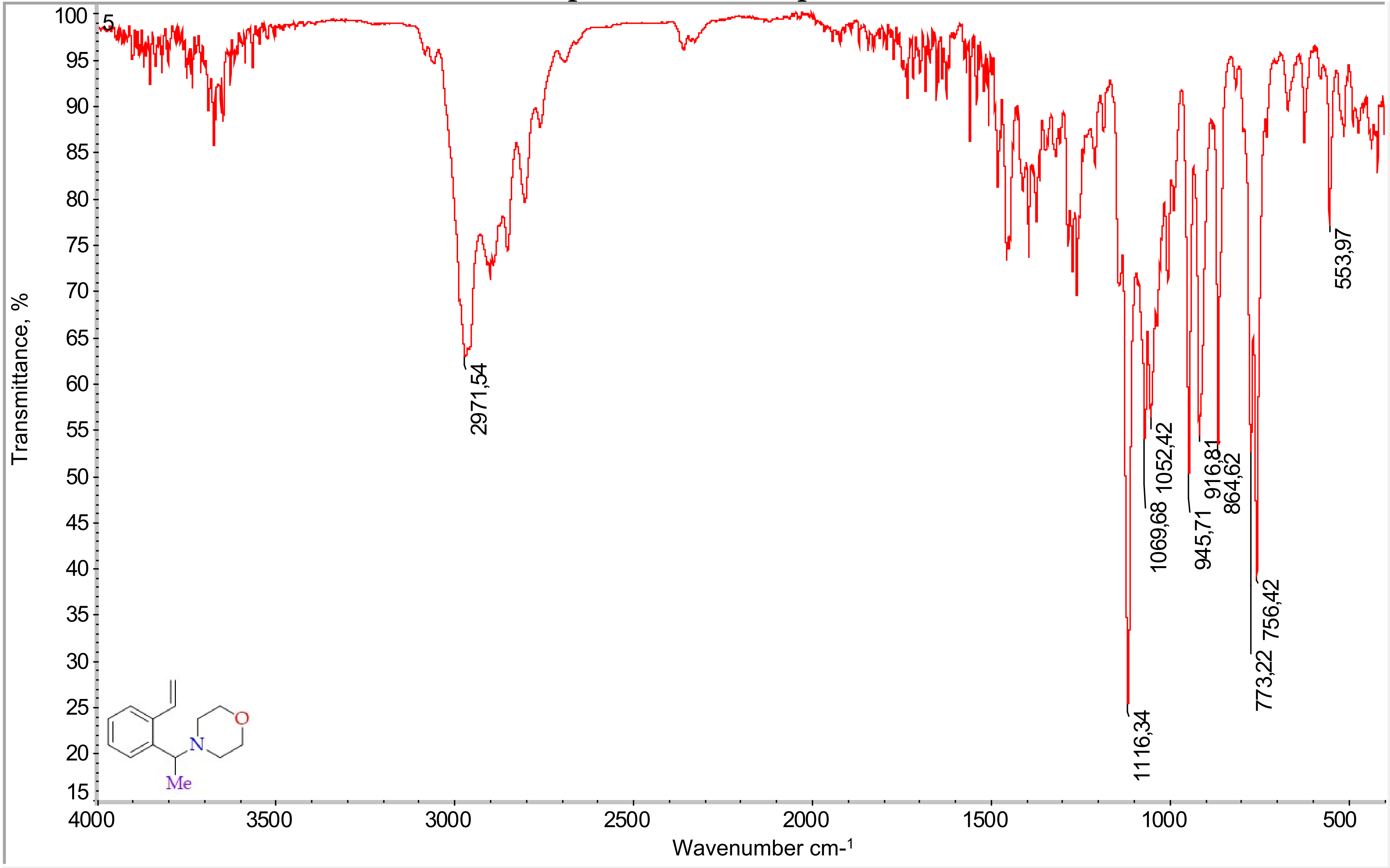

HRMS (MALDI-TOF) spectrum of compound **1d**

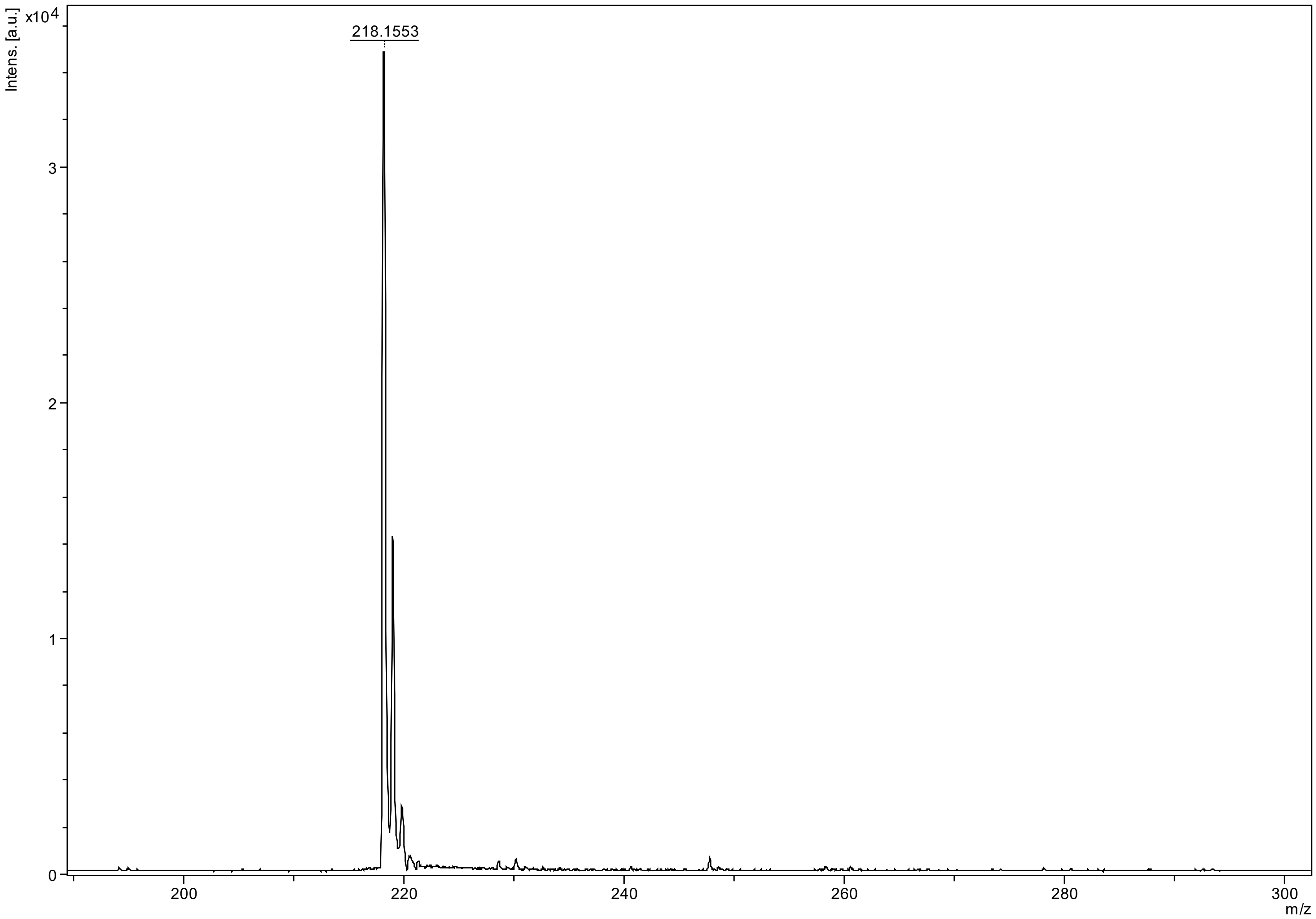

^{1}H NMR spectrum of compound **2a**

Acquisition Time (sec)	1.9818	***Comment***	single_pulse	***Date***			
Date Stamp							
File Name							
Frequency (MHz)	600.17	***Nucleus***	1H	***Number of Transients***	4	***Origin***	ECA 600
Original Points Count	32768	***Owner***	CKP	***Points Count***	32768	***Pulse Sequence***	single_pulse.ex2
Receiver Gain	26.00	***Solvent***	CHLOROFORM-d	***Spectrum Offset (Hz)***	4521.0088	***Sweep Width (Hz)***	16534.39

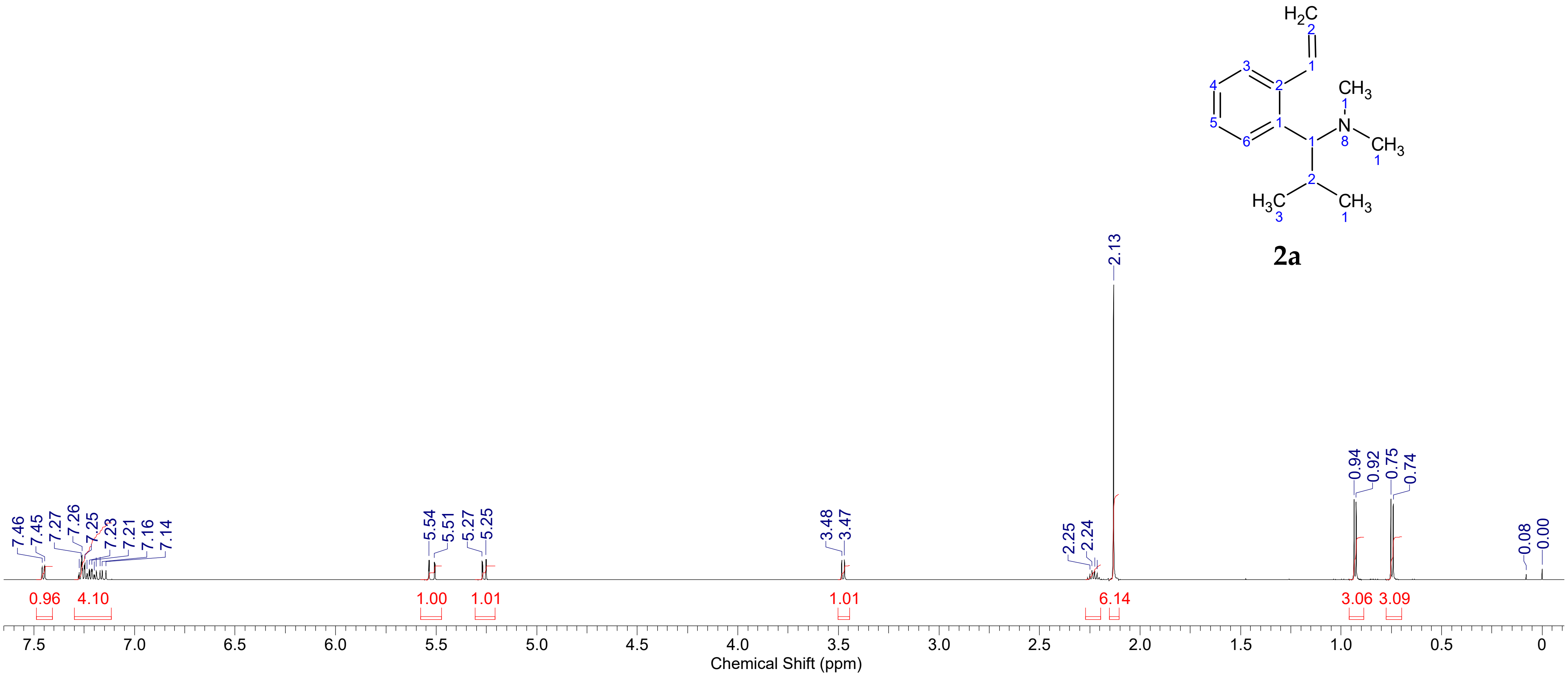

^{1}H NMR spectrum of compound **2a**

Acquisition Time (sec)	1.9818	***Comment***	single_pulse	***Date***			
Date Stamp							
File Name							
Frequency (MHz)	600.17	***Nucleus***	1H	***Number of Transients***	4	***Origin***	ECA 600
Original Points Count	32768	***Owner***	CKP	***Points Count***	32768	***Pulse Sequence***	single_pulse.ex2
Receiver Gain	26.00	***Solvent***	CHLOROFORM-d	***Spectrum Offset (Hz)***	4521.0088	***Sweep Width (Hz)***	16534.39

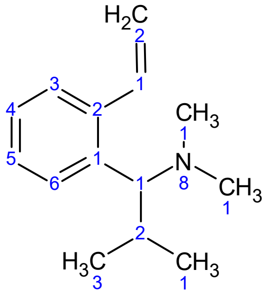

2a

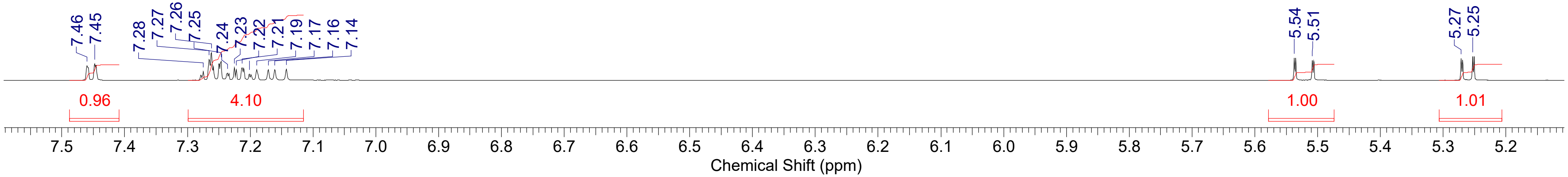

^{1}H NMR spectrum of compound **2a**

Acquisition Time (sec)	1.9818	Comment	single_pulse	Date			
Date Stamp							
File Name							
Frequency (MHz)	600.17	Nucleus	1H	Number of Transients	4	Origin	ECA 600
Original Points Count	32768	Owner	CKP	Points Count	32768	Pulse Sequence	single_pulse.ex2
Receiver Gain	26.00	Solvent	CHLOROFORM-d	Spectrum Offset (Hz)	4521.0088	Sweep Width (Hz)	16534.39

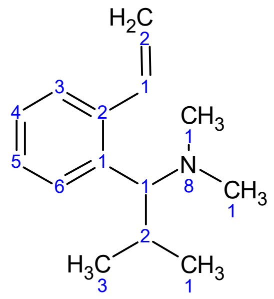

2a

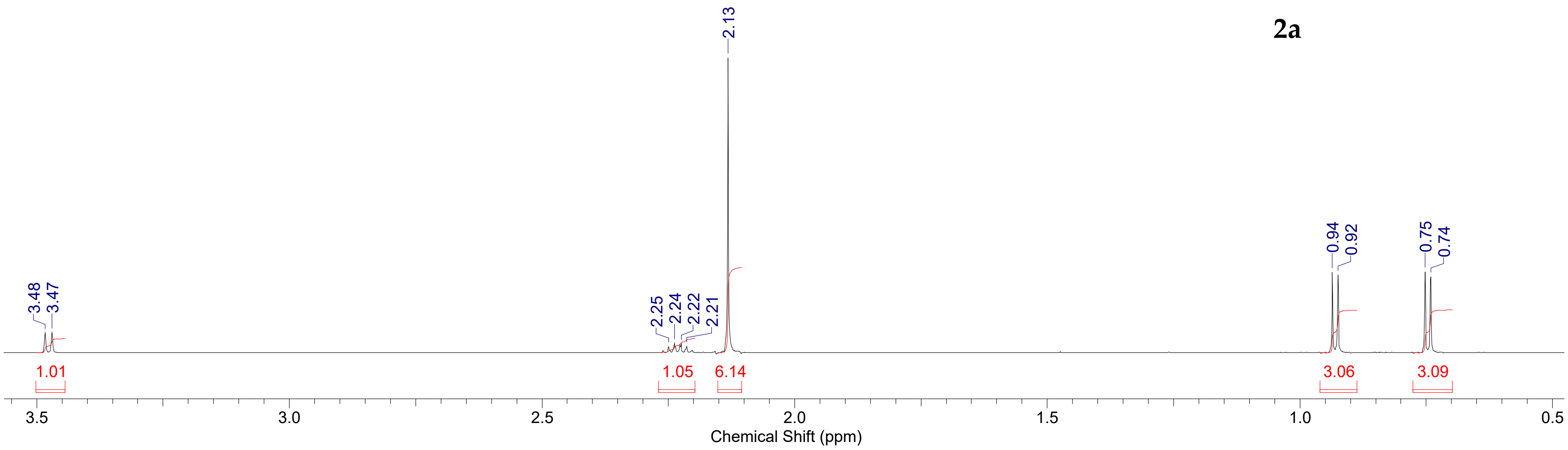

^{13}C NMR spectrum of compound **2a**

Acquisition Time (sec)	0.6921	***Comment***	single pulse decoupled gated NOE			***Date***	
Date Stamp							
File Name						***Frequency (MHz)***	150.91
Nucleus	13C	***Number of Transients***	1000	***Origin***	ECA 600	***Original Points Count***	32768
Owner	CKP	***Points Count***	32768	***Pulse Sequence***	single_pulse_dec	***Receiver Gain***	54.00
Solvent	CHLOROFORM-d	***Spectrum Offset (Hz)***	15076.4629	***Sweep Width (Hz)***	47348.49		

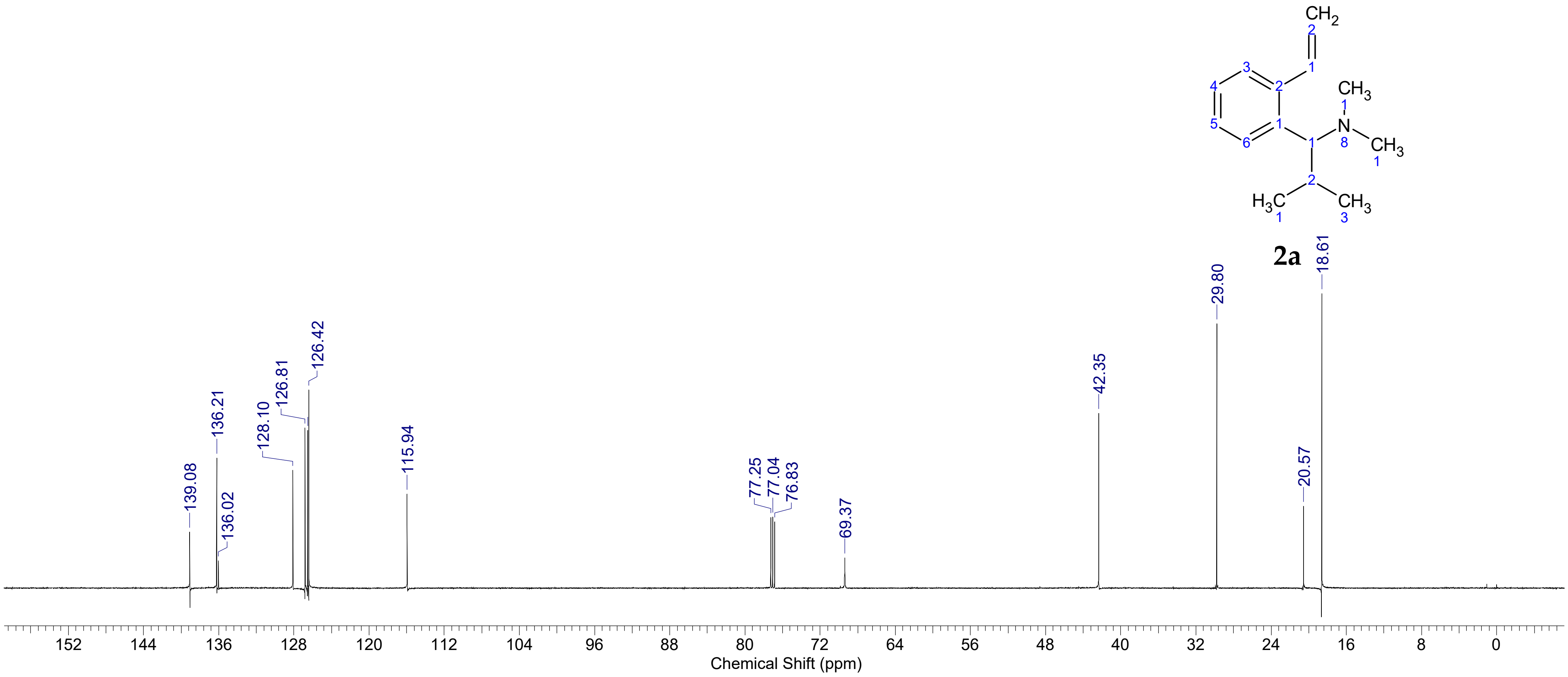

^{13}C NMR spectrum of compound **2a**

Acquisition Time (sec)	0.6921	***Comment***	single pulse decoupled gated NOE			***Date***	
Date Stamp							
File Name						***Frequency (MHz)***	150.91
Nucleus	13C	***Number of Transients***	1000	***Origin***	ECA 600	***Original Points Count***	32768
Owner	CKP	***Points Count***	32768	***Pulse Sequence***	single_pulse_dec	***Receiver Gain***	54.00
Solvent	CHLOROFORM-d	***Spectrum Offset (Hz)***	15076.4629	***Sweep Width (Hz)***	47348.49		

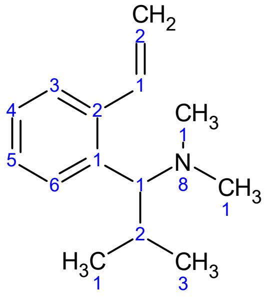

2a

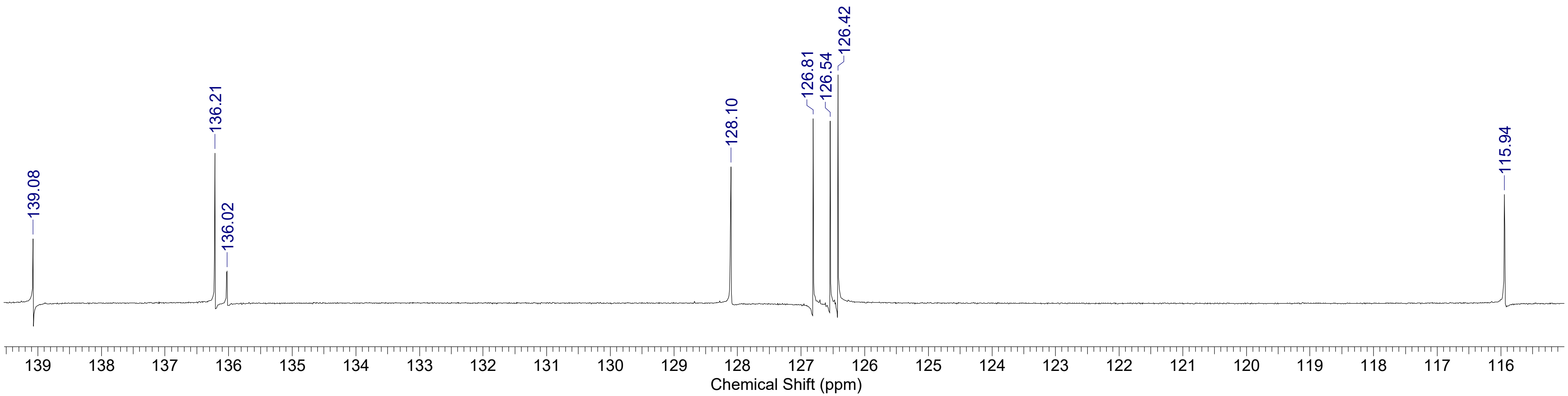

^{13}C NMR spectrum of compound **2a**

Acquisition Time (sec)	0.6921	***Comment***	single pulse decoupled gated NOE			***Date***	
Date Stamp							
File Name						***Frequency (MHz)***	150.91
Nucleus	13C	***Number of Transients***	1000	***Origin***	ECA 600	***Original Points Count***	32768
Owner	CKP	***Points Count***	32768	***Pulse Sequence***	single_pulse_dec	***Receiver Gain***	54.00
Solvent	CHLOROFORM-d	***Spectrum Offset (Hz)***	15076.4629	***Sweep Width (Hz)***	47348.49		

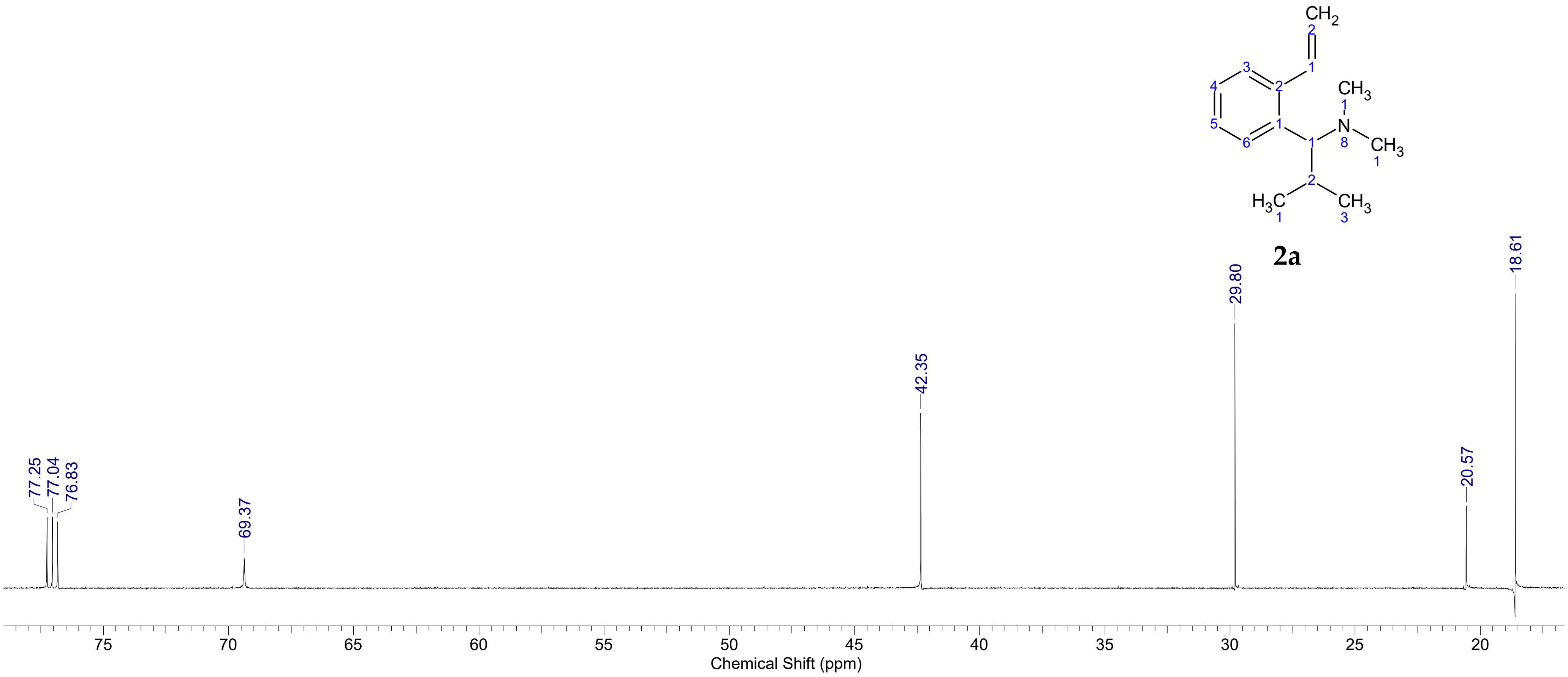

HRMS (MALDI-TOF) spectrum of compound **2a**

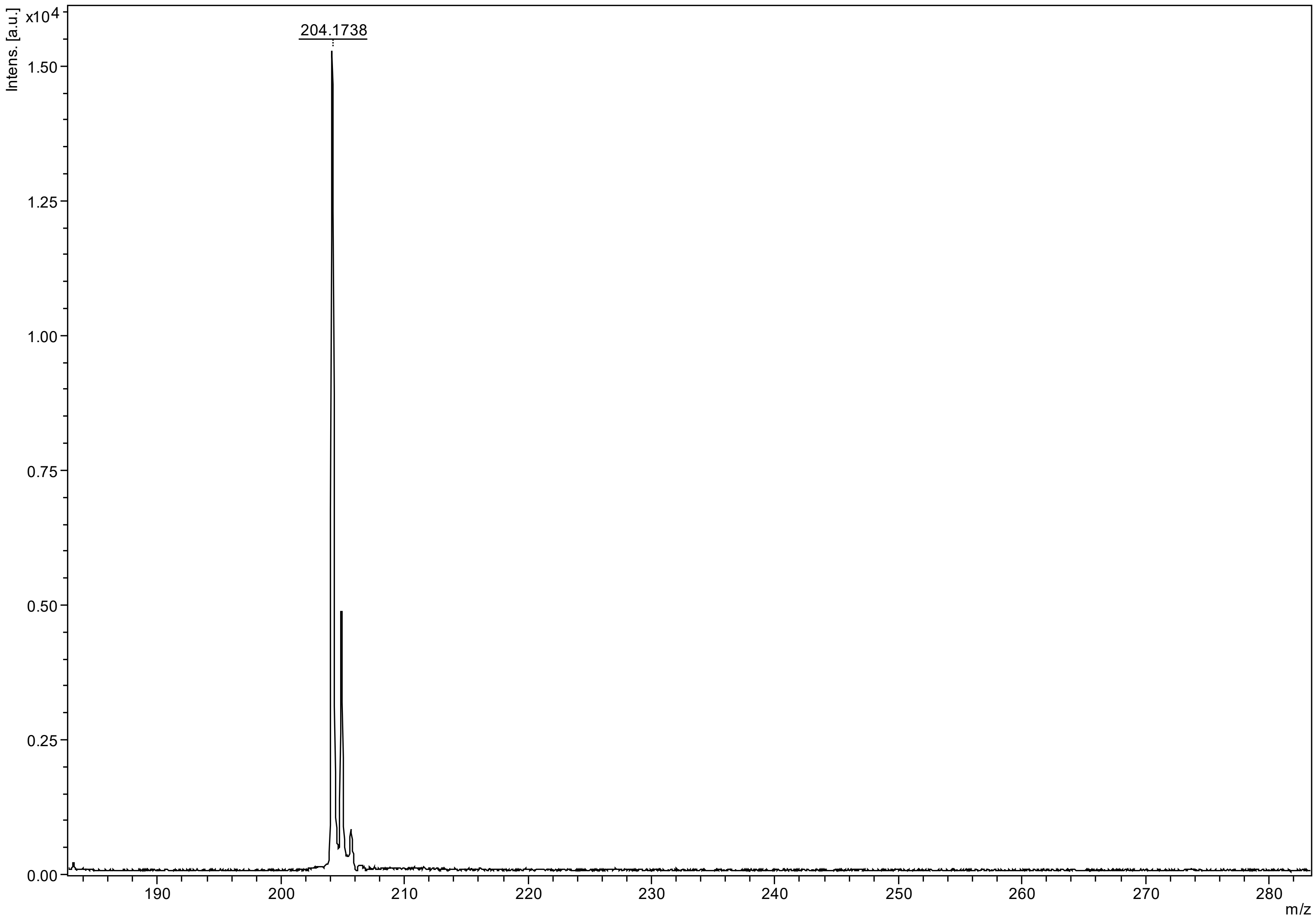

^{1}H NMR spectrum of compound **2b**

Acquisition Time (sec)	1.9818	***Comment***	single_pulse	***Date***			
Date Stamp							
File Name							
Frequency (MHz)	600.17	***Nucleus***	1H	***Number of Transients***	8	***Origin***	ECA 600
Original Points Count	32768	***Owner***	CKP	***Points Count***	32768	***Pulse Sequence***	single_pulse.ex2
Receiver Gain	24.00	***Solvent***	CHLOROFORM-d	***Spectrum Offset (Hz)***	5390.9478	***Sweep Width (Hz)***	16534.39

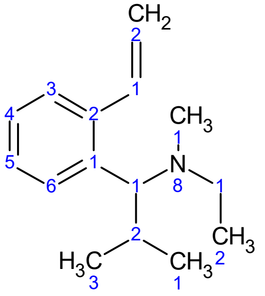

2b

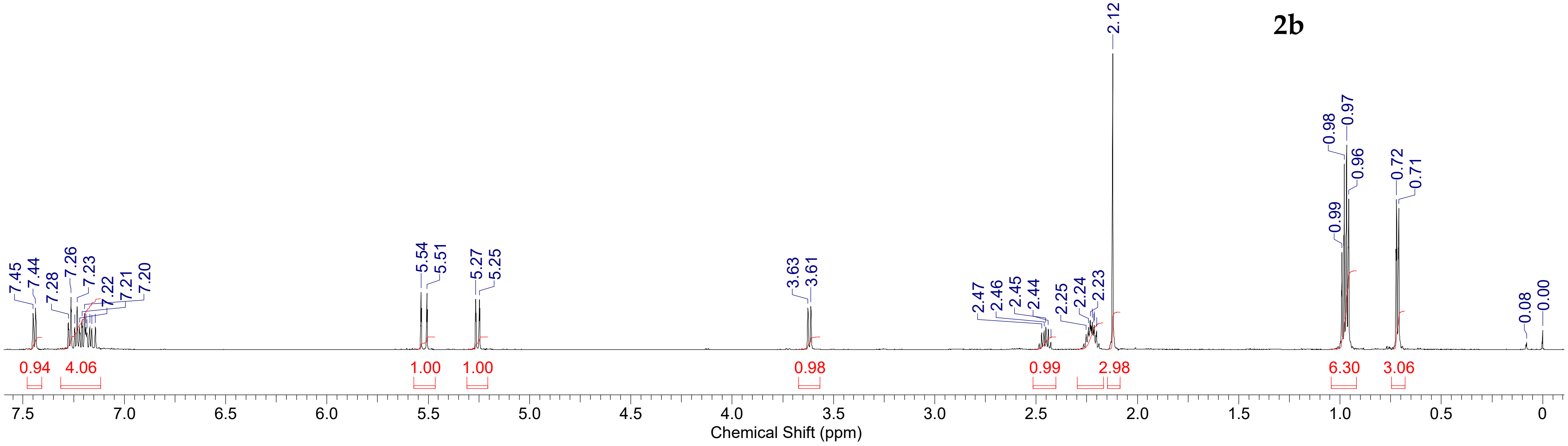

^{1}H NMR spectrum of compound **2b**

Acquisition Time (sec)	1.9818	***Comment***	single_pulse	***Date***			
Date Stamp							
File Name							
Frequency (MHz)	600.17	***Nucleus***	1H	***Number of Transients***	8	***Origin***	ECA 600
Original Points Count	32768	***Owner***	CKP	***Points Count***	32768	***Pulse Sequence***	single_pulse.ex2
Receiver Gain	24.00	***Solvent***	CHLOROFORM-d	***Spectrum Offset (Hz)***	5390.9478	***Sweep Width (Hz)***	16534.39

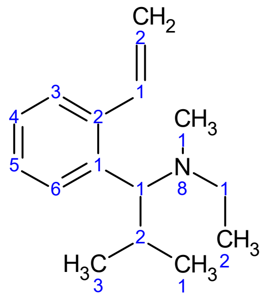

2b

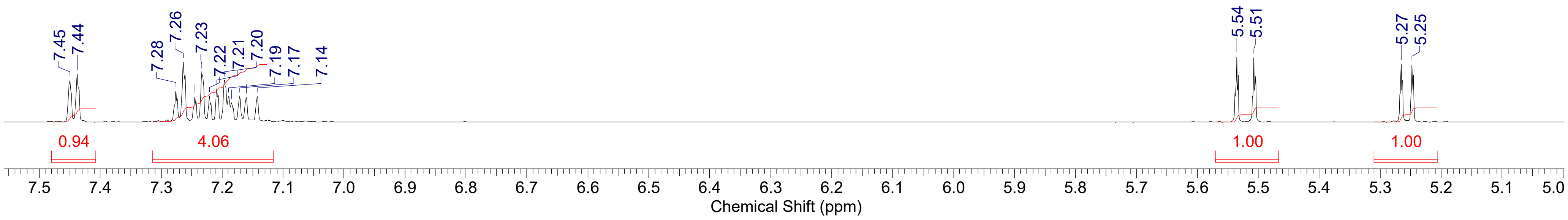

^{1}H NMR spectrum of compound **2b**

Acquisition Time (sec)	1.9818	***Comment***	single_pulse	***Date***			
Date Stamp							
File Name							
Frequency (MHz)	600.17	***Nucleus***	1H	***Number of Transients***	8	***Origin***	ECA 600
Original Points Count	32768	***Owner***	CKP	***Points Count***	32768	***Pulse Sequence***	single_pulse.ex2
Receiver Gain	24.00	***Solvent***	CHLOROFORM-d	***Spectrum Offset (Hz)***	5390.9478	***Sweep Width (Hz)***	16534.39

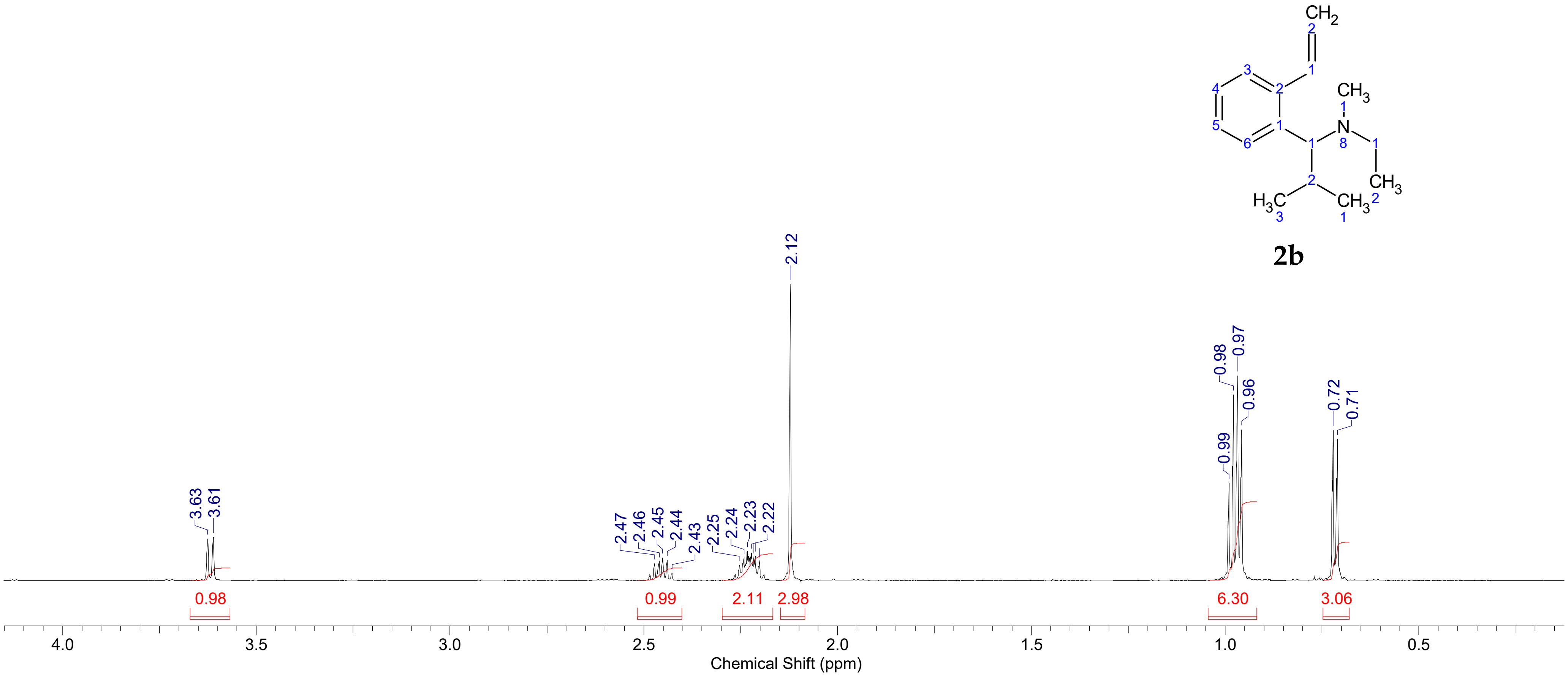

^{13}C NMR spectrum of compound **2b**

Acquisition Time (sec)	0.6921	***Comment***	single pulse decoupled gated NOE			***Date***	
Date Stamp							
File Name							
Frequency (MHz)	150.91	***Nucleus***	13C	***Number of Transients***	800	***Origin***	ECA 600
Original Points Count	32768	***Owner***	CKP	***Points Count***	32768	***Pulse Sequence***	single_pulse_dec
Receiver Gain	54.00	***Solvent***	CHLOROFORM-d	***Spectrum Offset (Hz)***	15075.0176	***Sweep Width (Hz)***	47348.49

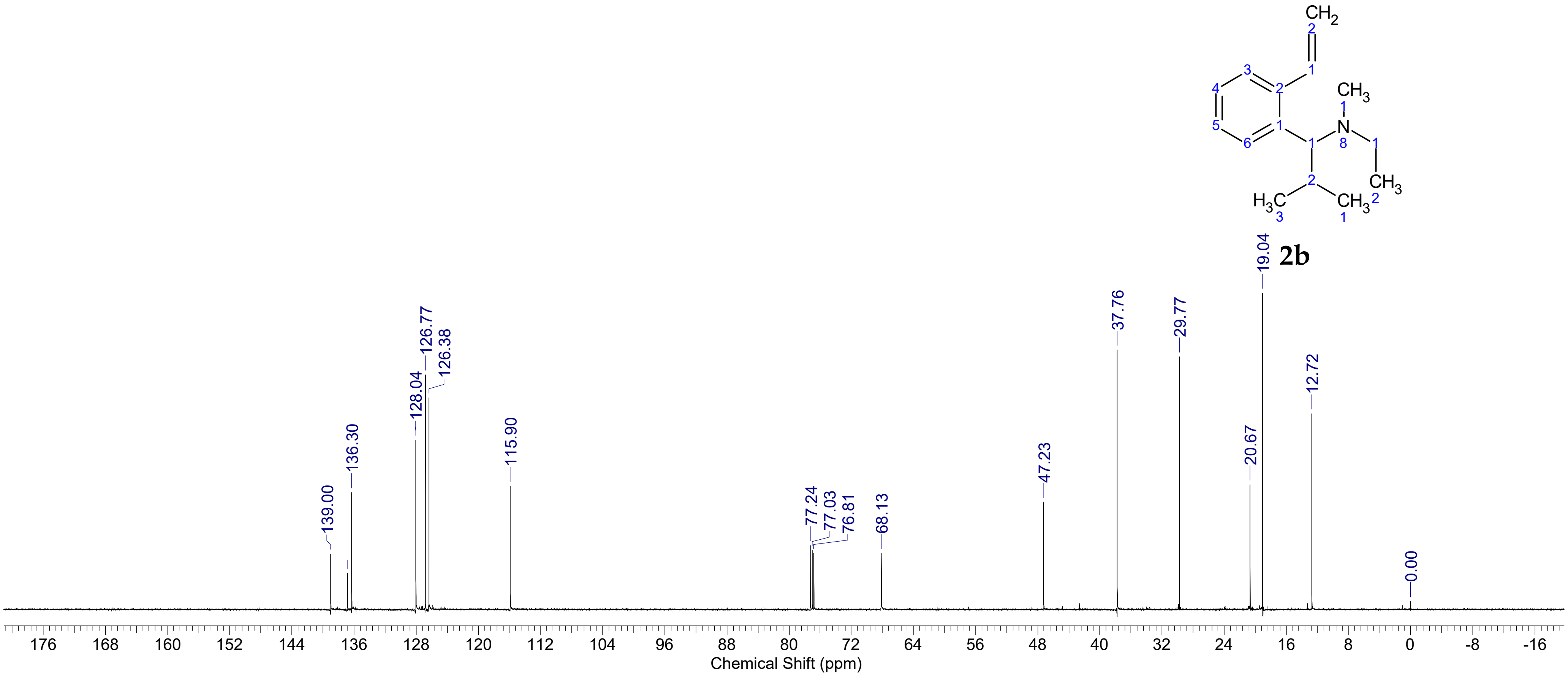

^{13}C NMR spectrum of compound **2b**

Acquisition Time (sec)	0.6921	***Comment***	single pulse decoupled gated NOE			***Date***	
Date Stamp							
File Name							
Frequency (MHz)	150.91	***Nucleus***	13C	***Number of Transients***	800	***Origin***	ECA 600
Original Points Count	32768	***Owner***	CKP	***Points Count***	32768	***Pulse Sequence***	single_pulse_dec
Receiver Gain	54.00	***Solvent***	CHLOROFORM-d	***Spectrum Offset (Hz)***	15075.0176	***Sweep Width (Hz)***	47348.49

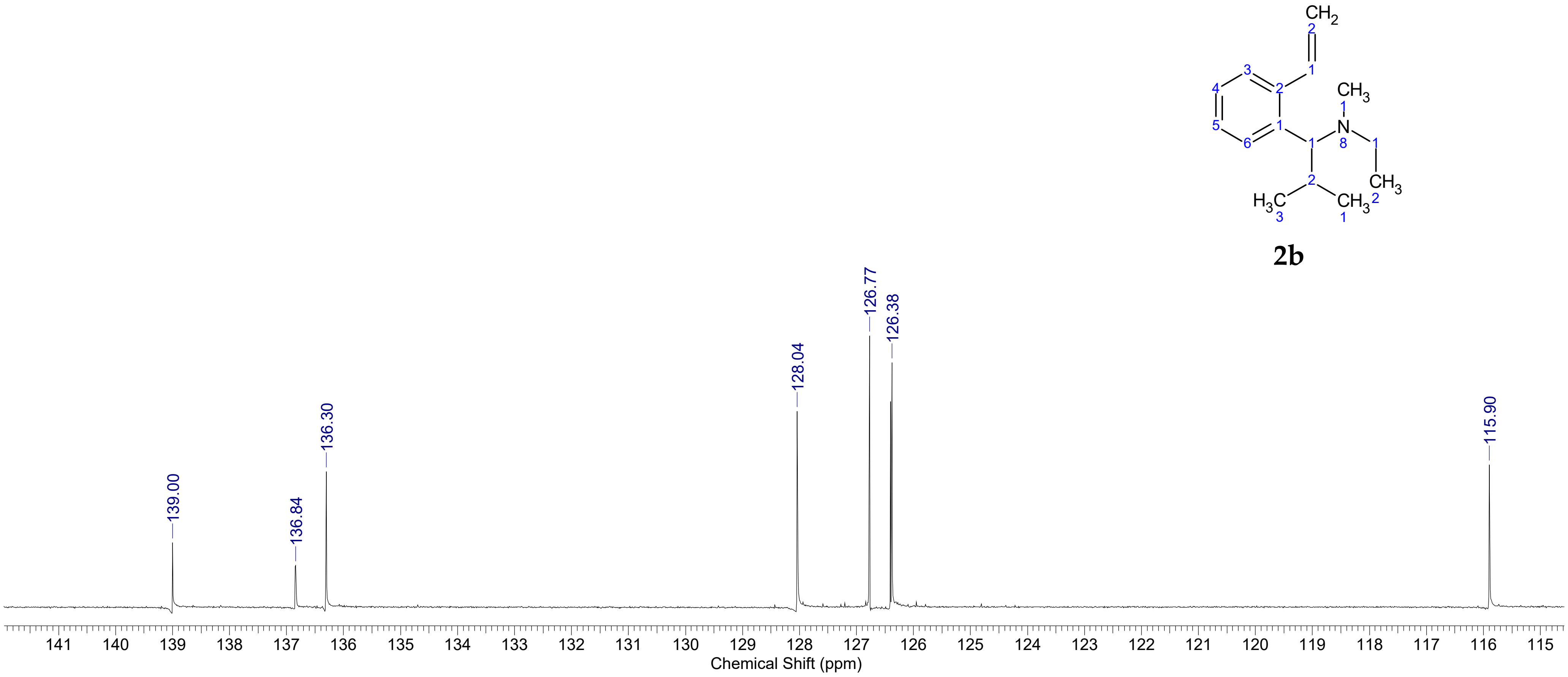

^{13}C NMR spectrum of compound **2b**

Acquisition Time (sec)	0.6921	***Comment***	single pulse decoupled gated NOE			***Date***	
Date Stamp							
File Name							
Frequency (MHz)	150.91	***Nucleus***	13C	***Number of Transients***	800	***Origin***	ECA 600
Original Points Count	32768	***Owner***	CKP	***Points Count***	32768	***Pulse Sequence***	single_pulse_dec
Receiver Gain	54.00	***Solvent***	CHLOROFORM-d	***Spectrum Offset (Hz)***	15075.0176	***Sweep Width (Hz)***	47348.49

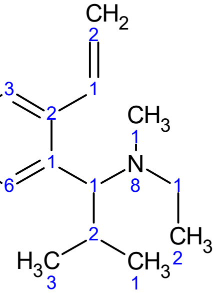

2b

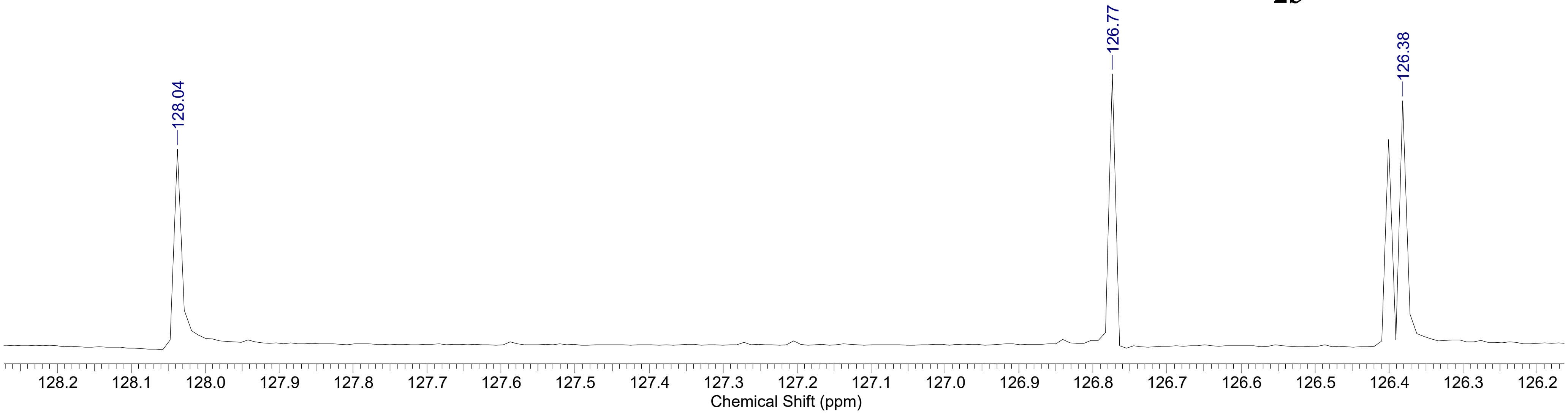

^{13}C NMR spectrum of compound **2b**

Acquisition Time (sec)	0.6921	***Comment***	single pulse decoupled gated NOE			***Date***	
Date Stamp							
File Name							
Frequency (MHz)	150.91	***Nucleus***	13C	***Number of Transients***	800	***Origin***	ECA 600
Original Points Count	32768	***Owner***	CKP	***Points Count***	32768	***Pulse Sequence***	single_pulse_dec
Receiver Gain	54.00	***Solvent***	CHLOROFORM-d	***Spectrum Offset (Hz)***	15075.0176	***Sweep Width (Hz)***	47348.49

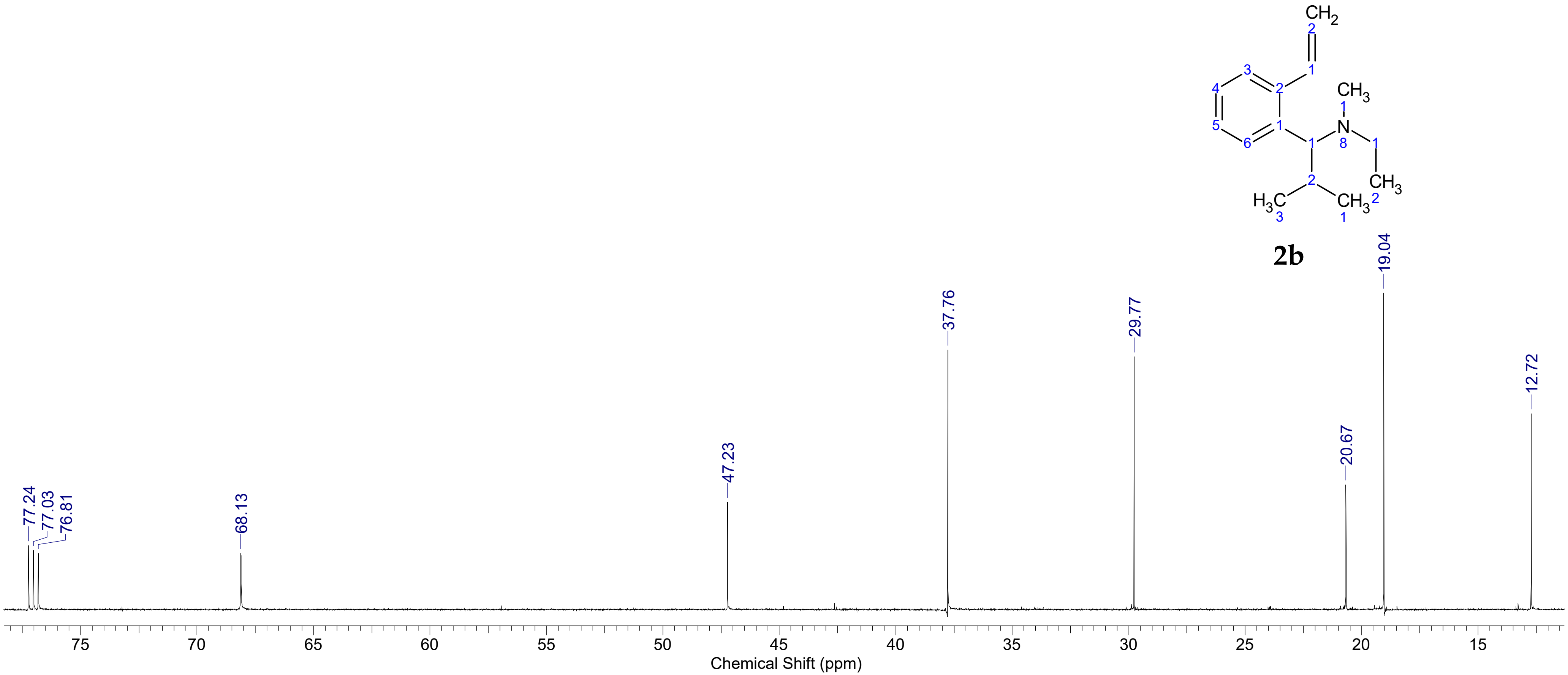

IR spectrum of compound **2b**

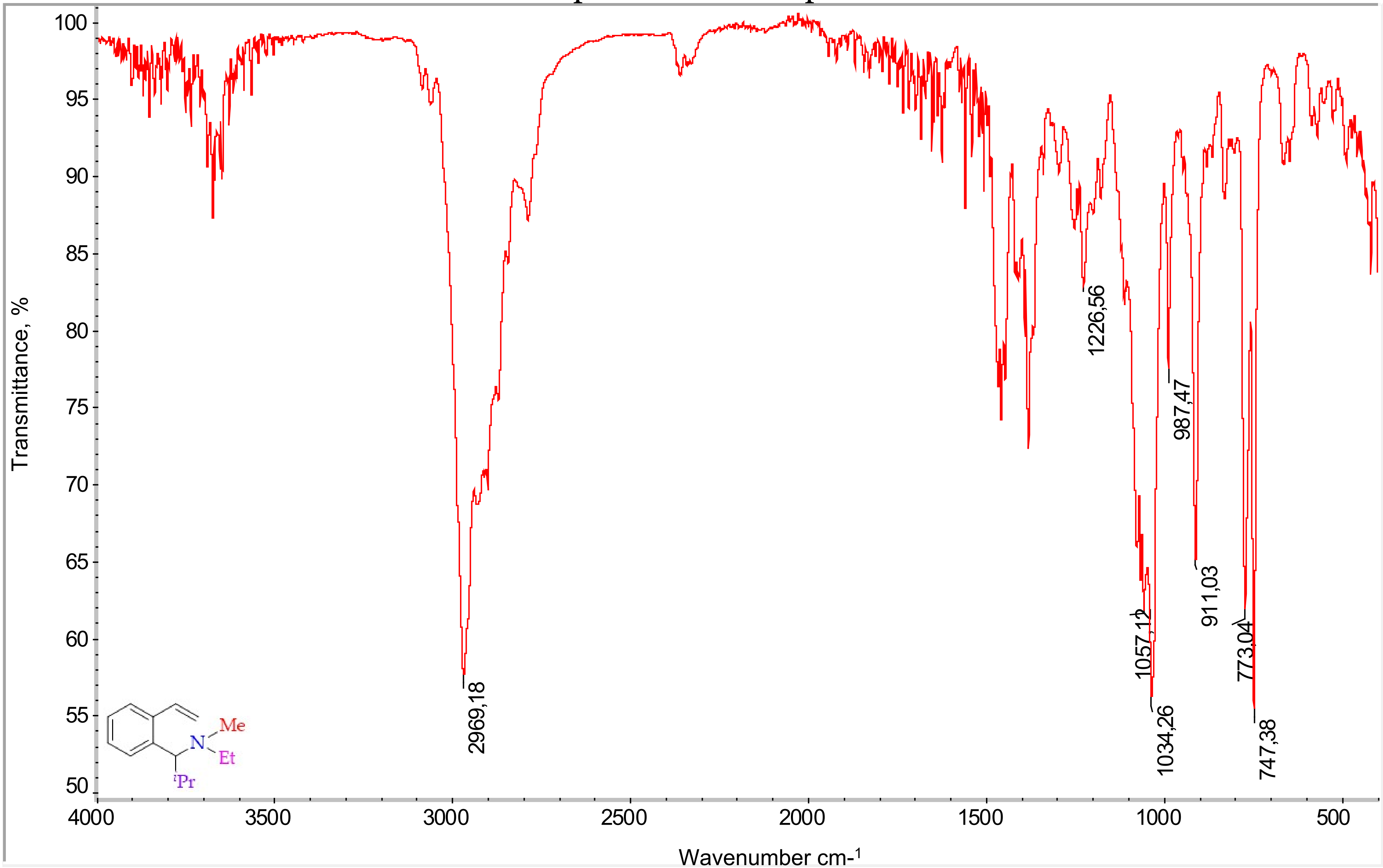

HRMS (MALDI-TOF) spectrum of compound **2b**

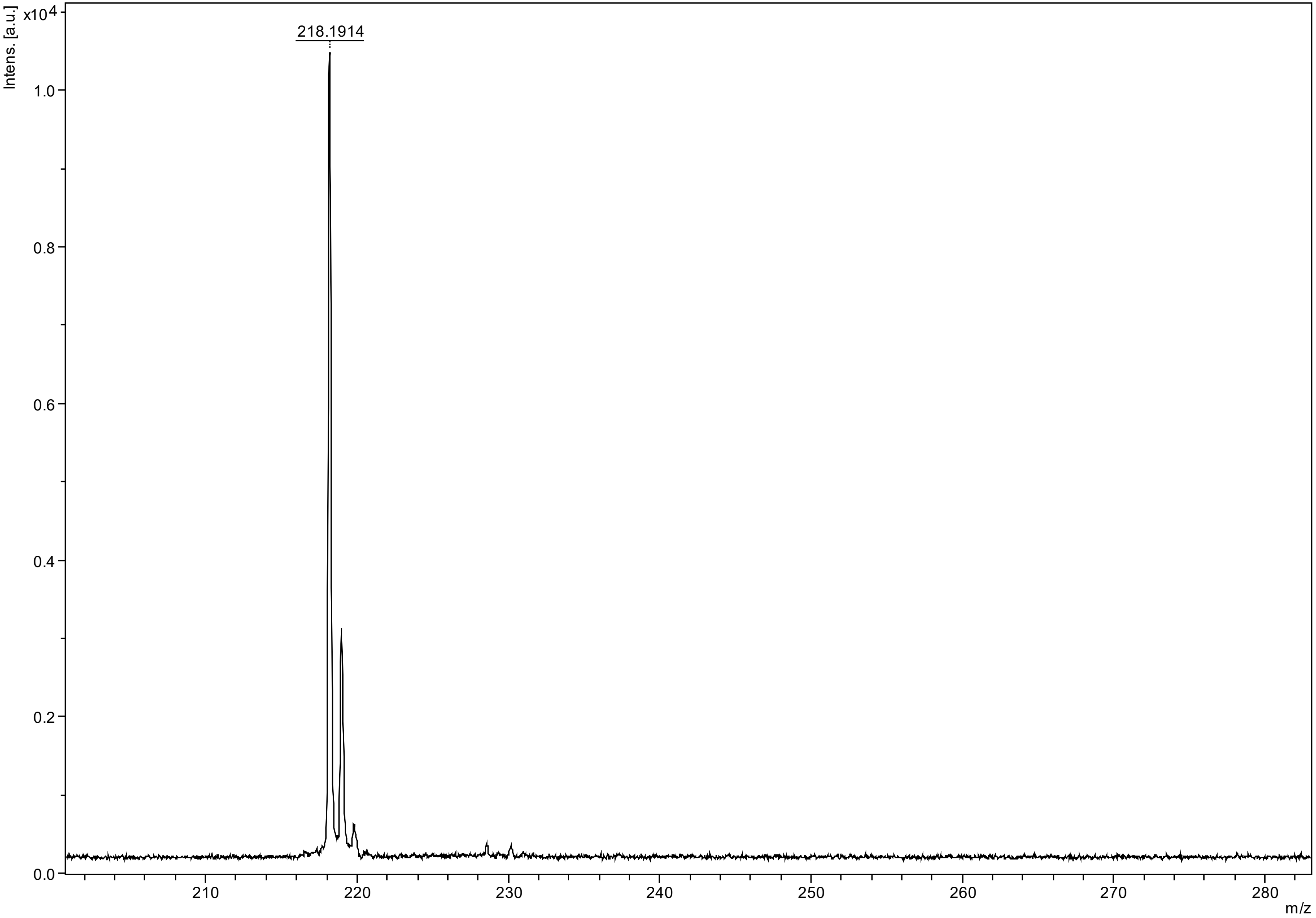

^{1}H NMR spectrum of compound **2c**

Acquisition Time (sec)	2.0972	Date		Date Stamp	
File Name					
Frequency (MHz)	700.17	Nucleus	1H	Number of Transients	8	Origin	Avance
Original Points Count	32768	Owner	nmr	Points Count	65536	Pulse Sequence	zg30
Receiver Gain	15.57	SW(cyclical) (Hz)	15625.00	Solvent	CHLOROFORM-d		
Spectrum Offset (Hz)	6271.2432	Sweep Width (Hz)	15624.76	Temperature (degree C)	24.997		

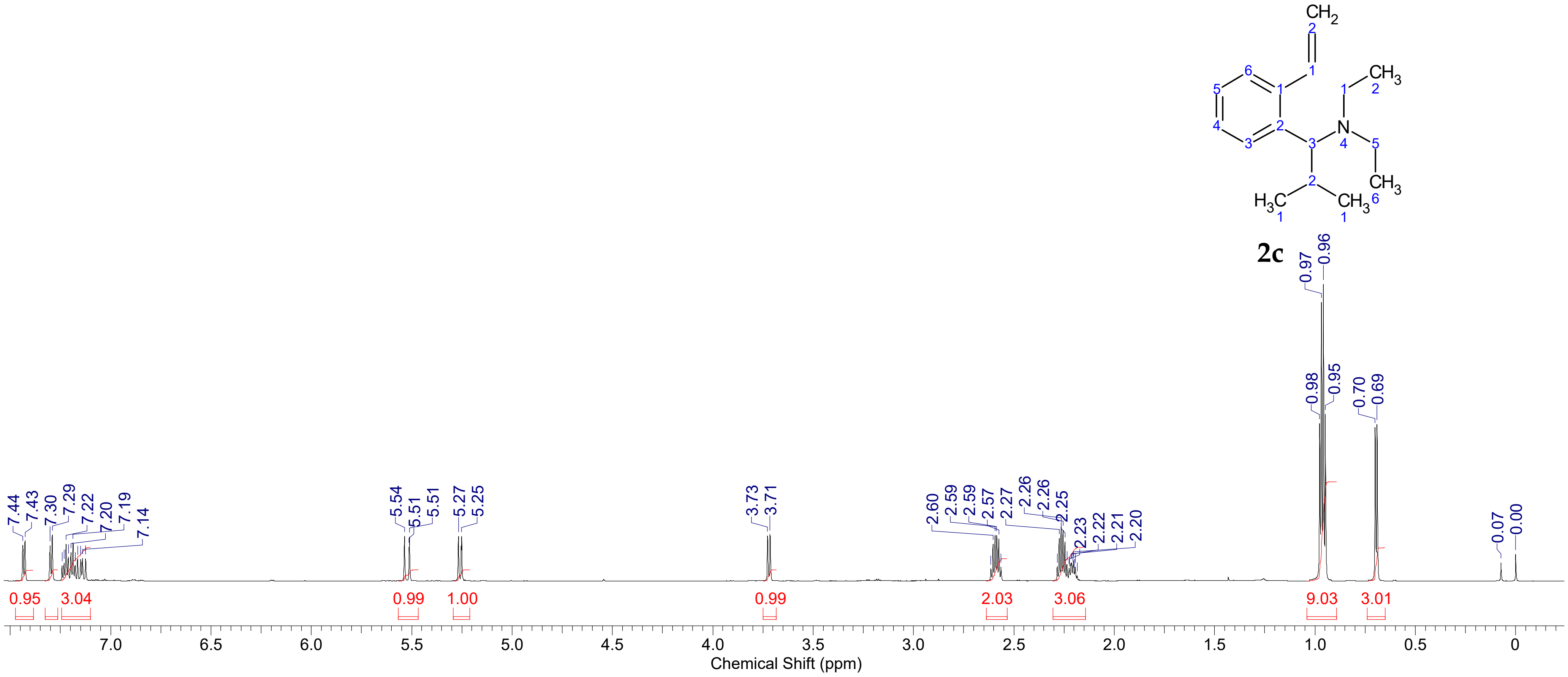

^{1}H NMR spectrum of compound **2c**

Acquisition Time (sec)	2.0972	***Date***				***Date Stamp***	
File Name							
Frequency (MHz)	700.17	***Nucleus***	1H	***Number of Transients***	8	***Origin***	Avance
Original Points Count	32768	***Owner***	nmr	***Points Count***	65536	***Pulse Sequence***	zg30
Receiver Gain	15.57	***SW(cyclical) (Hz)***	15625.00	***Solvent***	CHLOROFORM-d		
Spectrum Offset (Hz)	6271.2432	***Sweep Width (Hz)***	15624.76	***Temperature (degree C)***	24.997		

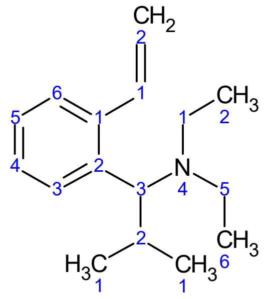

2c

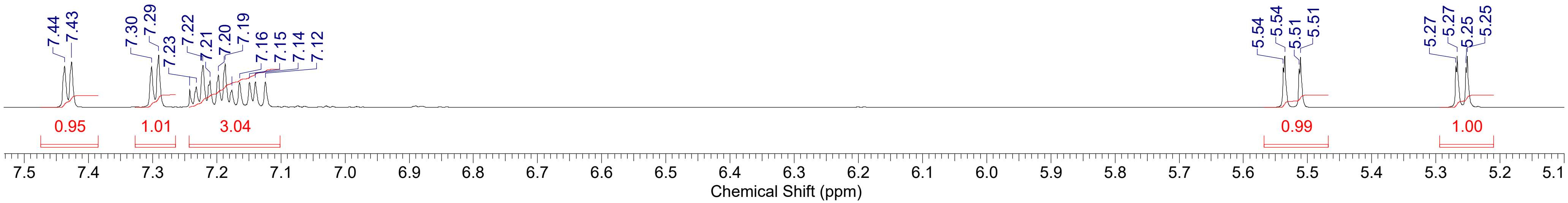

^{1}H NMR spectrum of compound **2c**

Acquisition Time (sec)	2.0972	Date				Date Stamp	
File Name							
Frequency (MHz)	700.17	Nucleus	1H	Number of Transients	8	Origin	Avance
Original Points Count	32768	Owner	nmr	Points Count	65536	Pulse Sequence	zg30
Receiver Gain	15.57	SW(cyclical) (Hz)	15625.00	Solvent	CHLOROFORM-d		
Spectrum Offset (Hz)	6271.2432	Sweep Width (Hz)	15624.76	Temperature (degree C)	24.997		

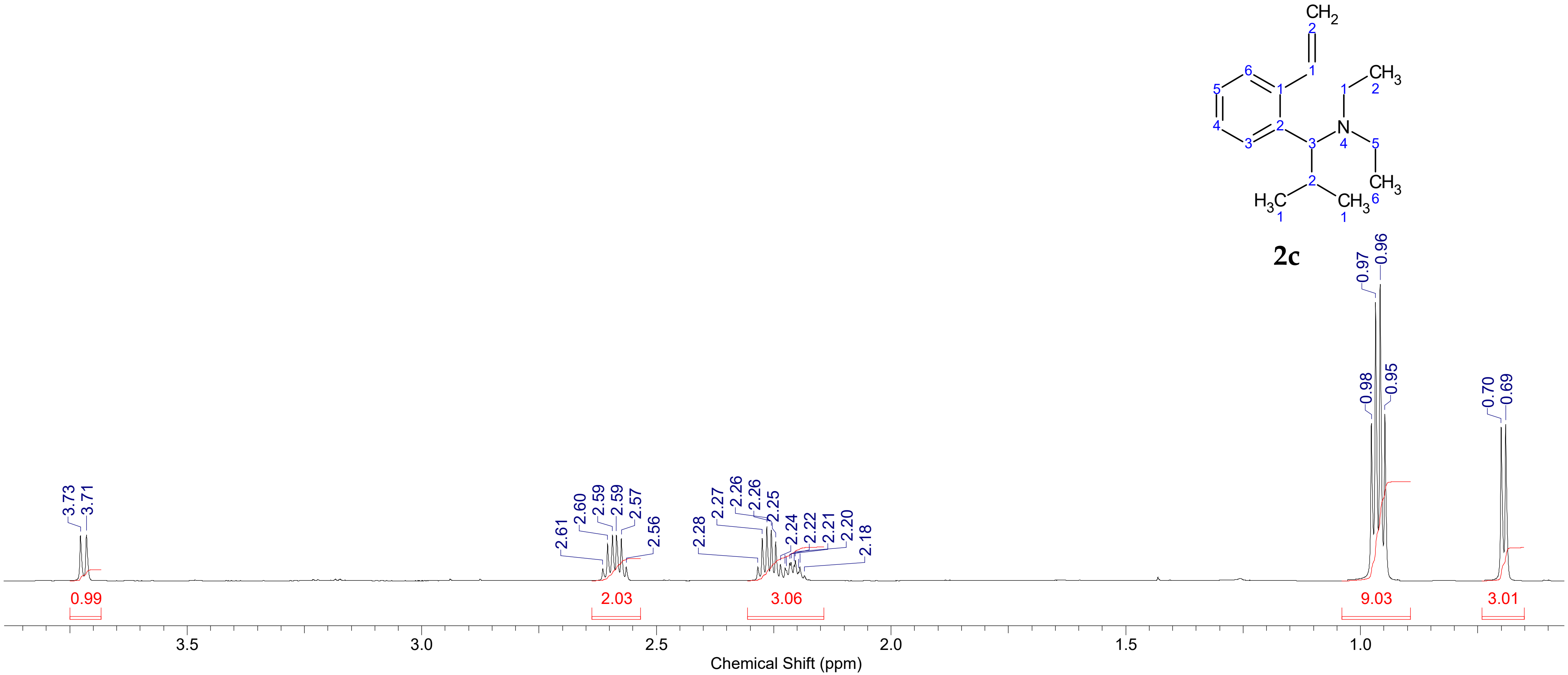

^{13}C NMR spectrum of compound **2c**

Acquisition Time (sec)	0.7406	Date				Date Stamp	
File Name							176.06
Nucleus	13C	Number of Transients	400	Origin	Avance	Original Points Count	32768
Owner	nmr	Points Count	32768	Pulse Sequence	zgpg30	Receiver Gain	101.00
SW(cyclical) (Hz)	44247.79	Solvent	CHLOROFORM-d			Spectrum Offset (Hz)	19363.1328
Sweep Width (Hz)	44246.44	Temperature (degree C)	24.990				

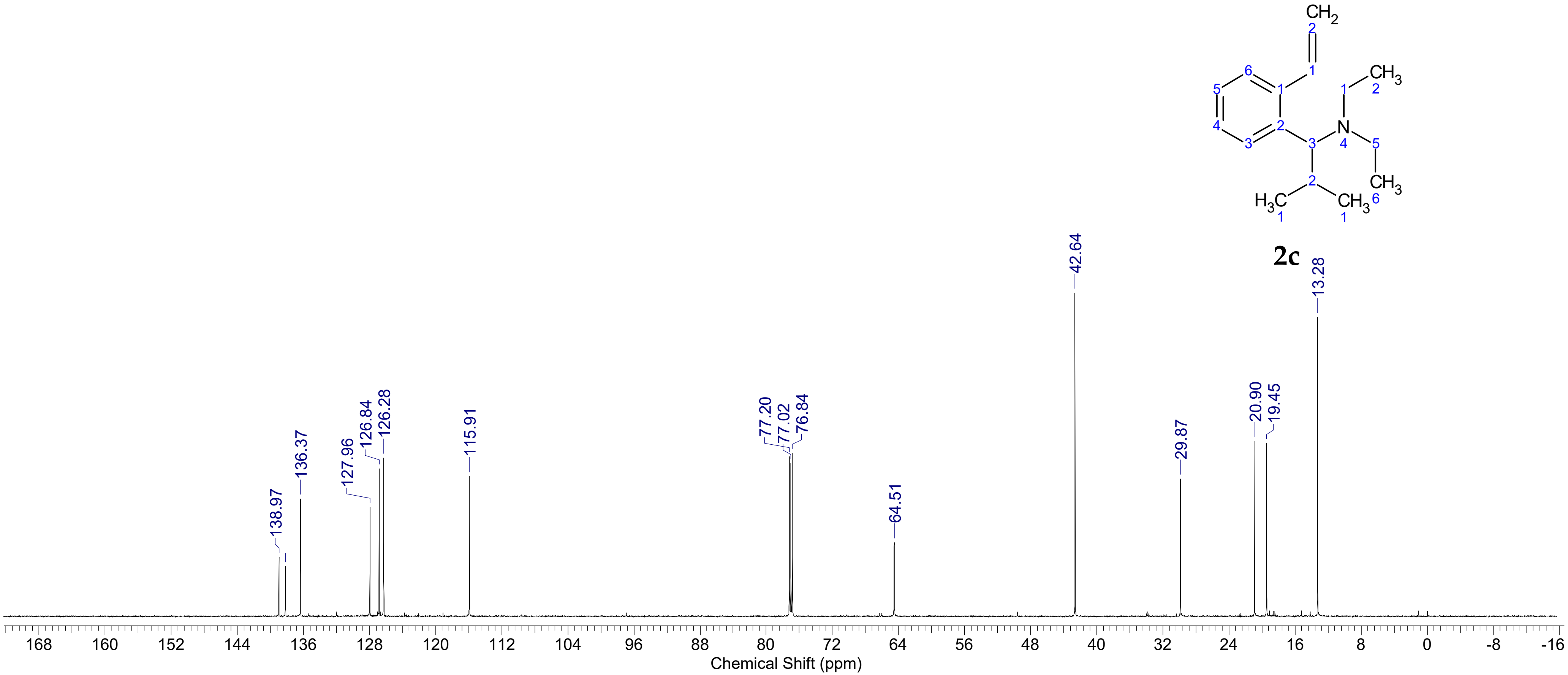

^{13}C NMR spectrum of compound **2c**

Acquisition Time (sec)	0.7406	Date				Date Stamp	
File Name							176.06
Nucleus	13C	Number of Transients	400	Origin	Avance	Original Points Count	32768
Owner	nmr	Points Count	32768	Pulse Sequence	zgpg30	Receiver Gain	101.00
SW(cyclical) (Hz)	44247.79	Solvent	CHLOROFORM-d			Spectrum Offset (Hz)	19363.1328
Sweep Width (Hz)	44246.44	Temperature (degree C)	24.990				

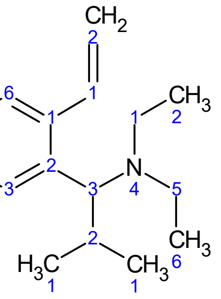

2c

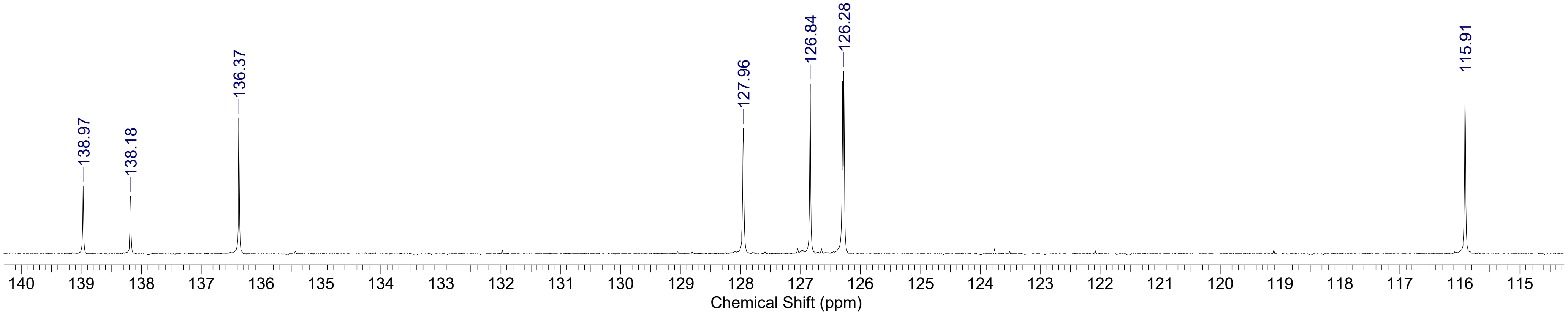

^{13}C NMR spectrum of compound **2c**

Acquisition Time (sec)	0.7406	***Date***				***Date Stamp***	
File Name							176.06
Nucleus	13C	***Number of Transients***	400	***Origin***	Avance	***Original Points Count***	32768
Owner	nmr	***Points Count***	32768	***Pulse Sequence***	zgpg30	***Receiver Gain***	101.00
SW(cyclical) (Hz)	44247.79	***Solvent***	CHLOROFORM-d			***Spectrum Offset (Hz)***	19363.1328
Sweep Width (Hz)	44246.44	***Temperature (degree C)***	24.990				

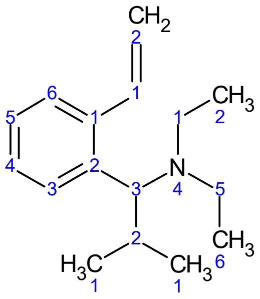

2c

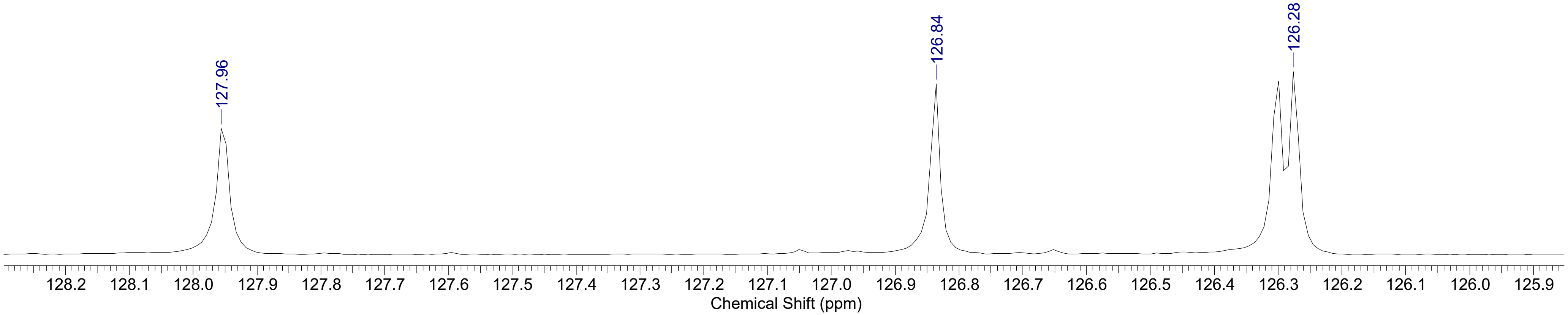

^{13}C NMR spectrum of compound **2c**

Acquisition Time (sec)	0.7406	***Date***				***Date Stamp***	
File Name							176.06
Nucleus	13C	***Number of Transients***	400	***Origin***	Avance	***Original Points Count***	32768
Owner	nmr	***Points Count***	32768	***Pulse Sequence***	zgpg30	***Receiver Gain***	101.00
SW(cyclical) (Hz)	44247.79	***Solvent***	CHLOROFORM-d			***Spectrum Offset (Hz)***	19363.1328
Sweep Width (Hz)	44246.44	***Temperature (degree C)***	24.990				

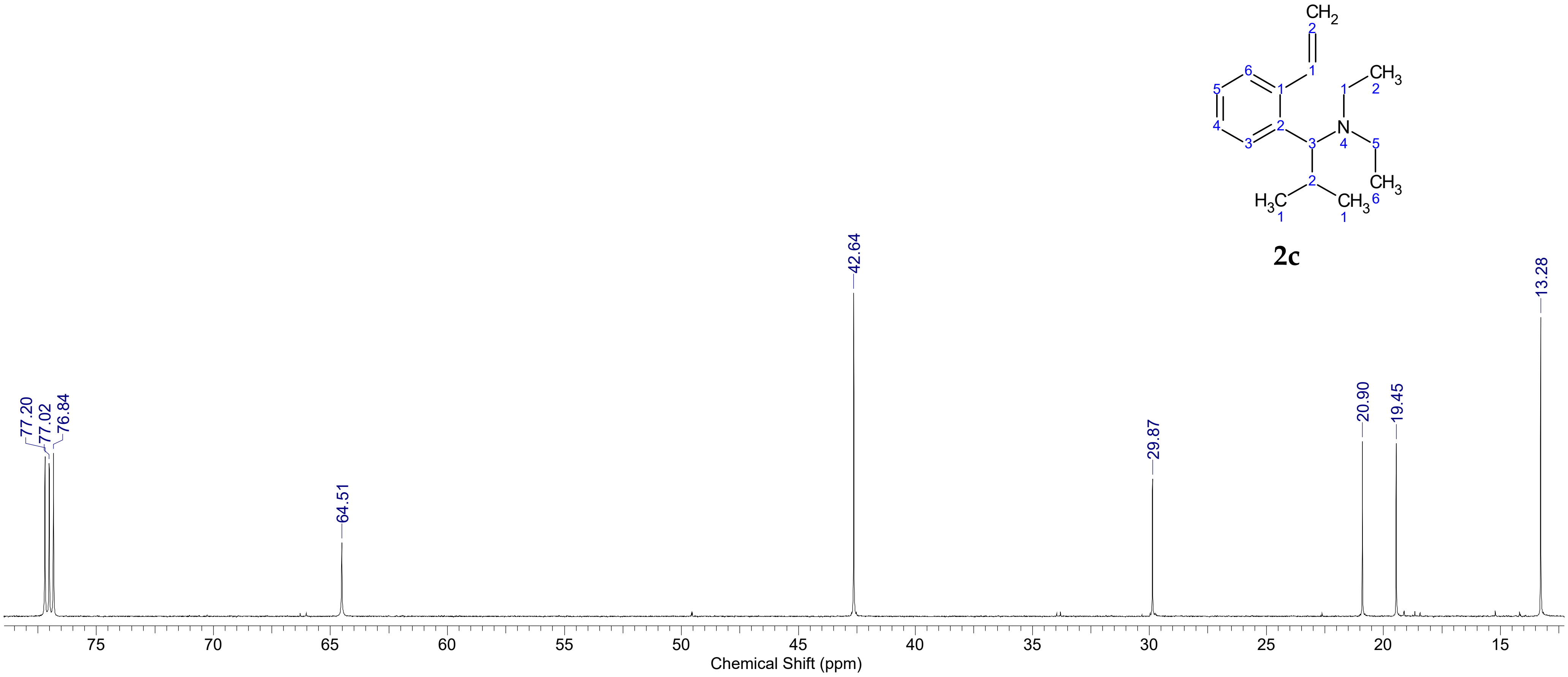

HRMS (MALDI-TOF) spectrum of compound **2c**

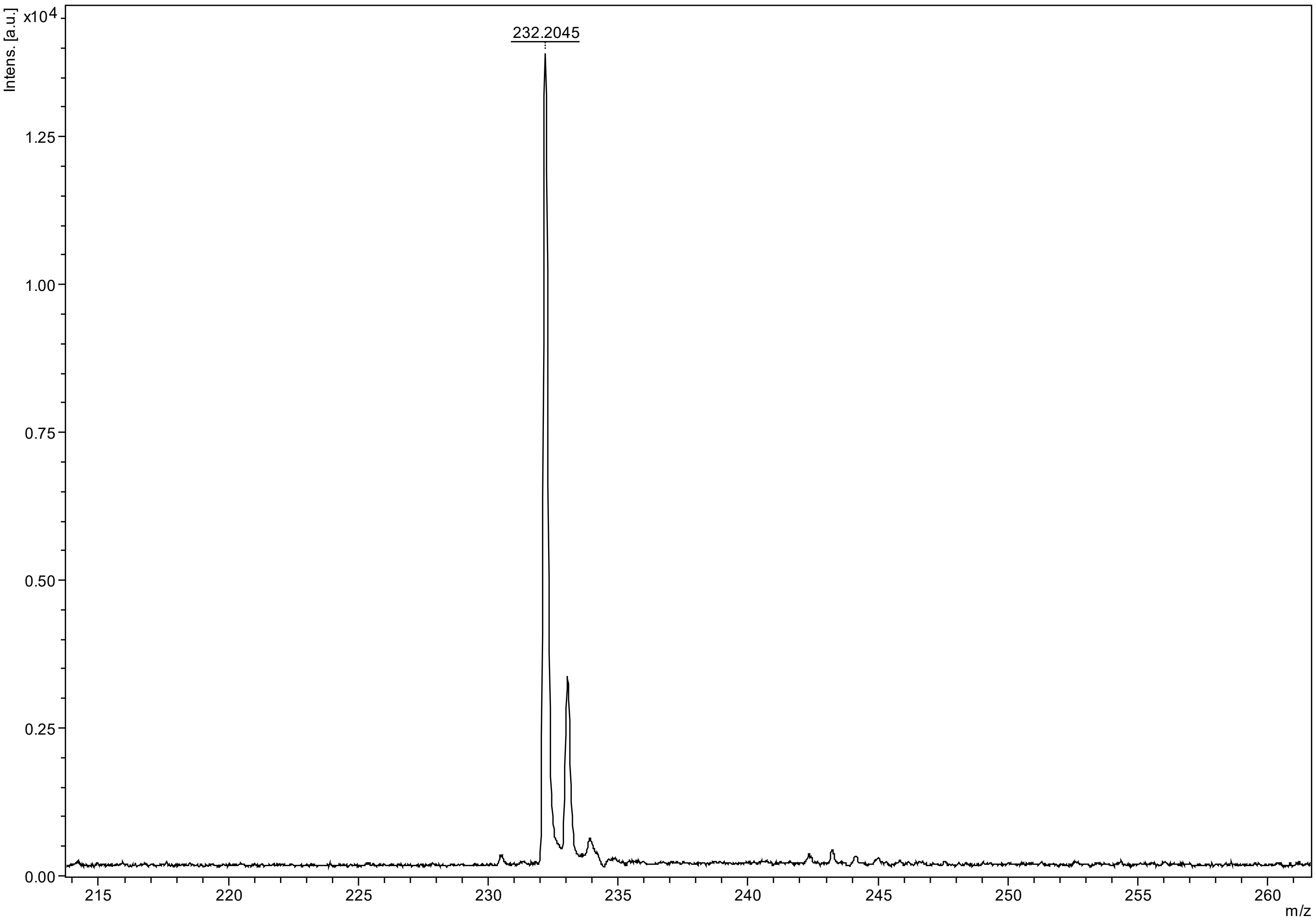

^{1}H NMR spectrum of compound **3a**

Acquisition Time (sec)	1.9818	***Comment***	single_pulse	***Date***			
Date Stamp							
File Name						***Frequency (MHz)***	600.17
Nucleus	1H	***Number of Transients***	8	***Origin***	ECA 600	***Original Points Count***	32768
Owner	CKP	***Points Count***	32768	***Pulse Sequence***	single_pulse.ex2	***Receiver Gain***	28.00
Solvent	CHLOROFORM-d	***Spectrum Offset (Hz)***	5401.5503	***Sweep Width (Hz)***	16534.39		

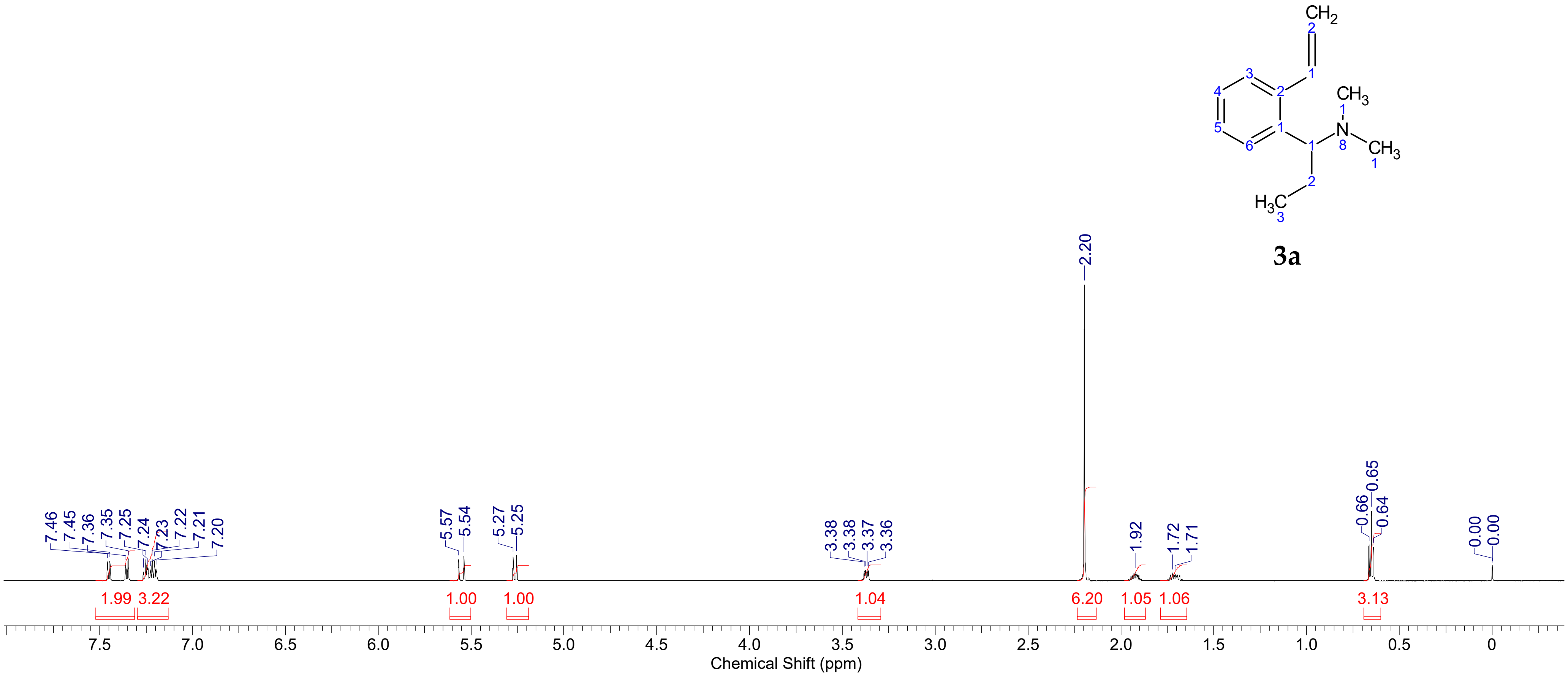

^{1}H NMR spectrum of compound **3a**

Acquisition Time (sec)	1.9818	Comment	single_pulse	Date			
Date Stamp							
File Name						Frequency (MHz)	600.17
Nucleus	1H	Number of Transients	8	Origin	ECA 600	Original Points Count	32768
Owner	CKP	Points Count	32768	Pulse Sequence	single_pulse.ex2	Receiver Gain	28.00
Solvent	CHLOROFORM-d	Spectrum Offset (Hz)	5401.5503	Sweep Width (Hz)	16534.39		

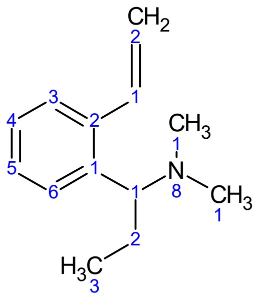

3a

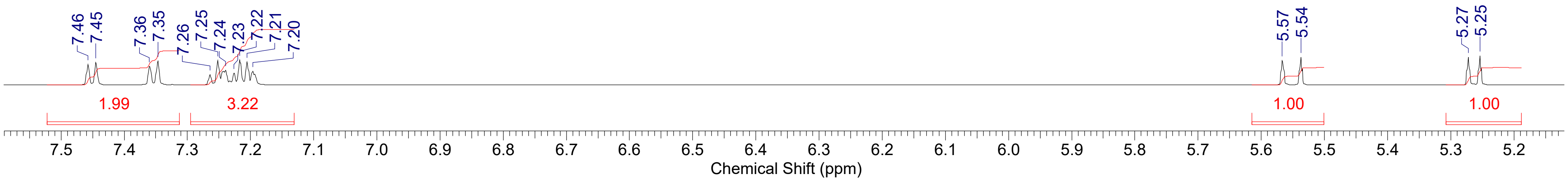

^{1}H NMR spectrum of compound **3a**

Acquisition Time (sec)	1.9818	***Comment***	single_pulse	***Date***			
Date Stamp							
File Name						***Frequency (MHz)***	600.17
Nucleus	1H	***Number of Transients***	8	***Origin***	ECA 600	***Original Points Count***	32768
Owner	CKP	***Points Count***	32768	***Pulse Sequence***	single_pulse.ex2	***Receiver Gain***	28.00
Solvent	CHLOROFORM-d	***Spectrum Offset (Hz)***	5401.5503	***Sweep Width (Hz)***	16534.39		

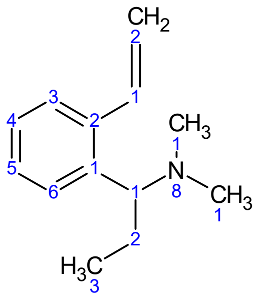

3a

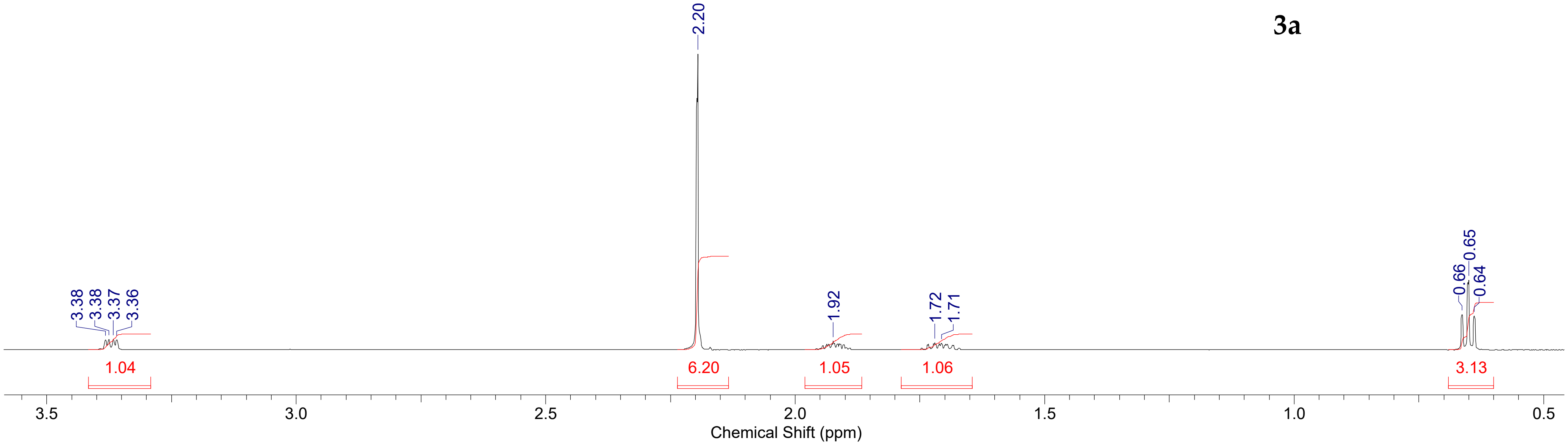

^{13}C NMR spectrum of compound **3a**

**Formula** $C_{13}H_{19}N$	**FW** 189.2967

**Acquisition Time (sec)**	0.6921	**Comment**	single pulse decoupled gated NOE			**Date**	07 Oct 2022 11:01:59
**Date Stamp**	07 Oct 2022 10:47:14						
**File Name**						**Frequency (MHz)**	150.91
**Nucleus**	13C	**Number of Transients**	1000	**Origin**	ECA 600	**Original Points Count**	32768
**Owner**	CKP	**Points Count**	32768	**Pulse Sequence**	single_pulse_dec	**Receiver Gain**	54.00
**Solvent**	CHLOROFORM-d	**Spectrum Offset (Hz)**	15077.9072	**Sweep Width (Hz)**	47348.49		

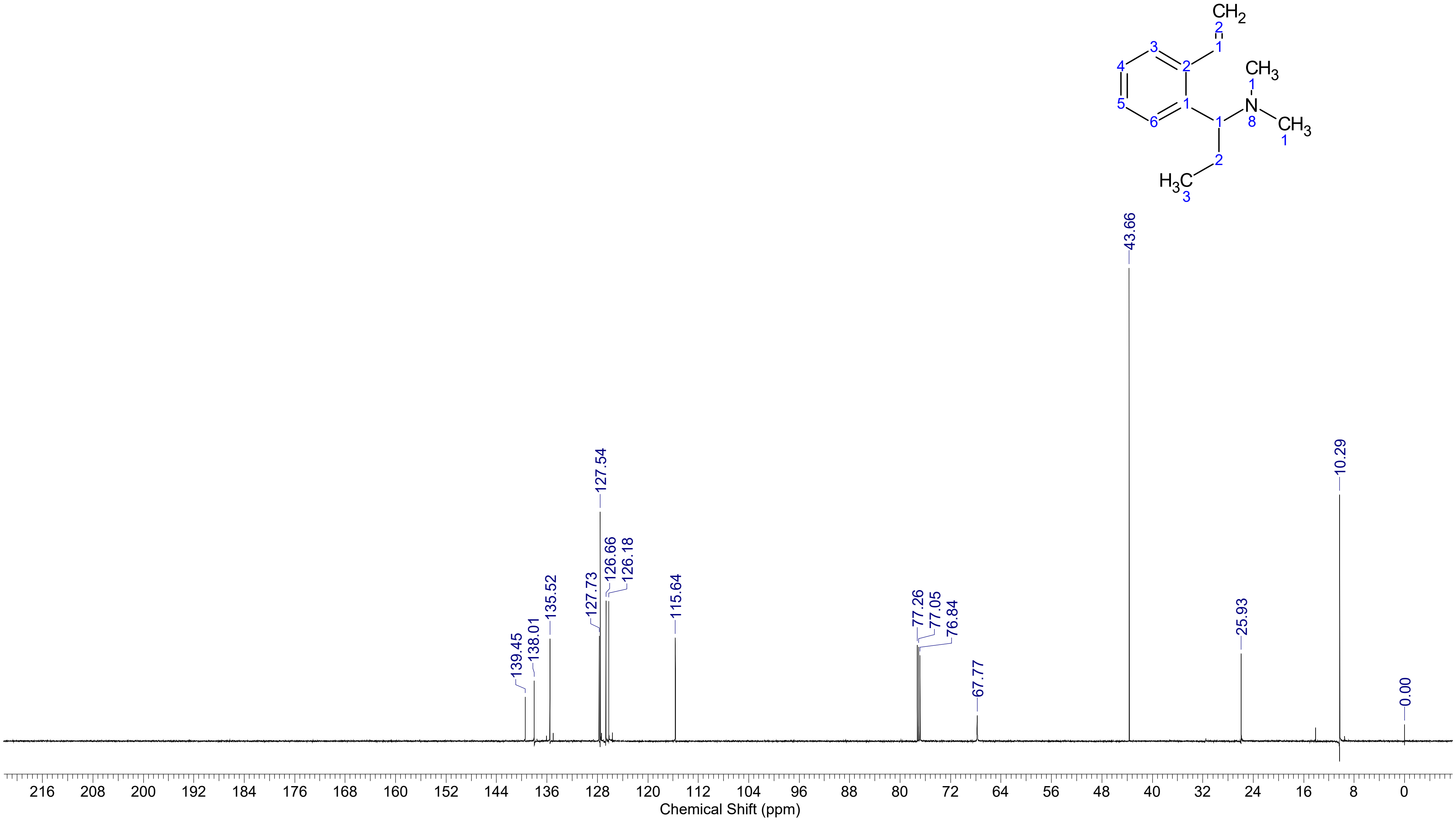

^{13}C NMR spectrum of compound **3a**

Formula	$C_{13}H_{19}N$	***FW***	189.2967

Acquisition Time (sec)	0.6921	***Comment***	single pulse decoupled gated NOE			***Date***	07 Oct 2022 11:01:59
Date Stamp	07 Oct 2022 10:47:14						
File Name						***Frequency (MHz)***	150.91
Nucleus	13C	***Number of Transients***	1000	***Origin***	ECA 600	***Original Points Count***	32768
Owner	CKP	***Points Count***	32768	***Pulse Sequence***	single_pulse_dec	***Receiver Gain***	54.00
Solvent	CHLOROFORM-d	***Spectrum Offset (Hz)***	15077.9072	***Sweep Width (Hz)***	47348.49		

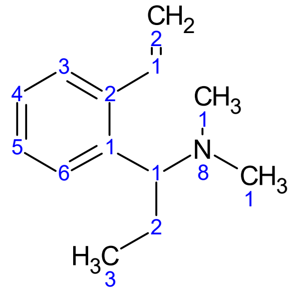

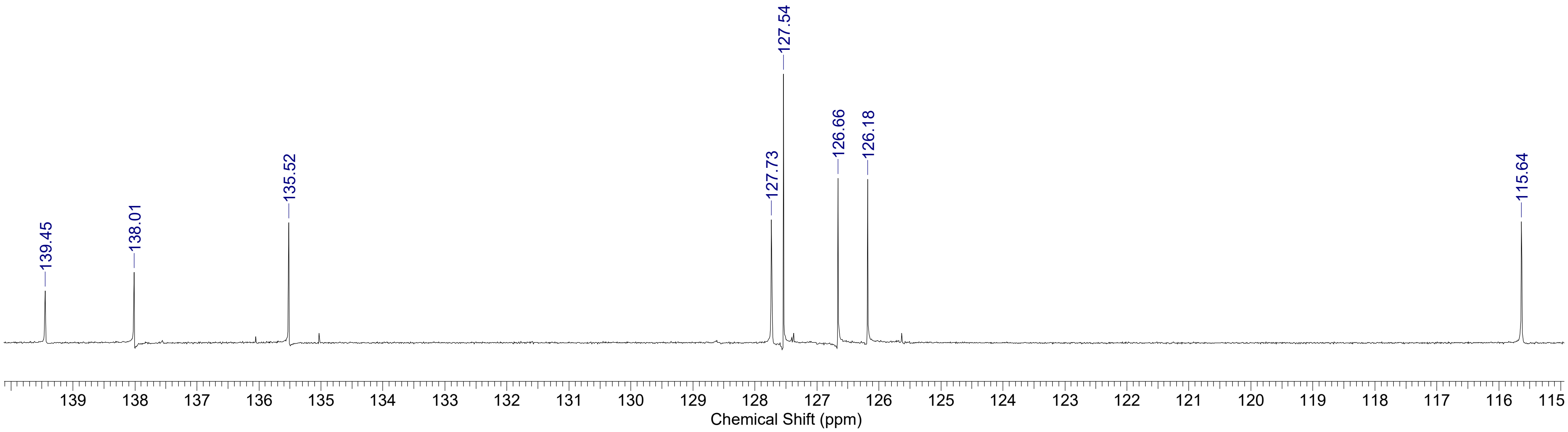

^{13}C NMR spectrum of compound **3a**

Formula $C_{13}H_{19}N$	FW 189.2967

**Acquisition Time (sec)**	0.6921	**Comment**	single pulse decoupled gated NOE			**Date**	07 Oct 2022 11:01:59
**Date Stamp**	07 Oct 2022 10:47:14						
**File Name**						**Frequency (MHz)**	150.91
**Nucleus**	13C	**Number of Transients**	1000	**Origin**	ECA 600	**Original Points Count**	32768
**Owner**	CKP	**Points Count**	32768	**Pulse Sequence**	single_pulse_dec	**Receiver Gain**	54.00
**Solvent**	CHLOROFORM-d	**Spectrum Offset (Hz)**	15077.9072	**Sweep Width (Hz)**	47348.49		

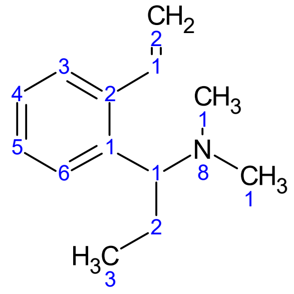

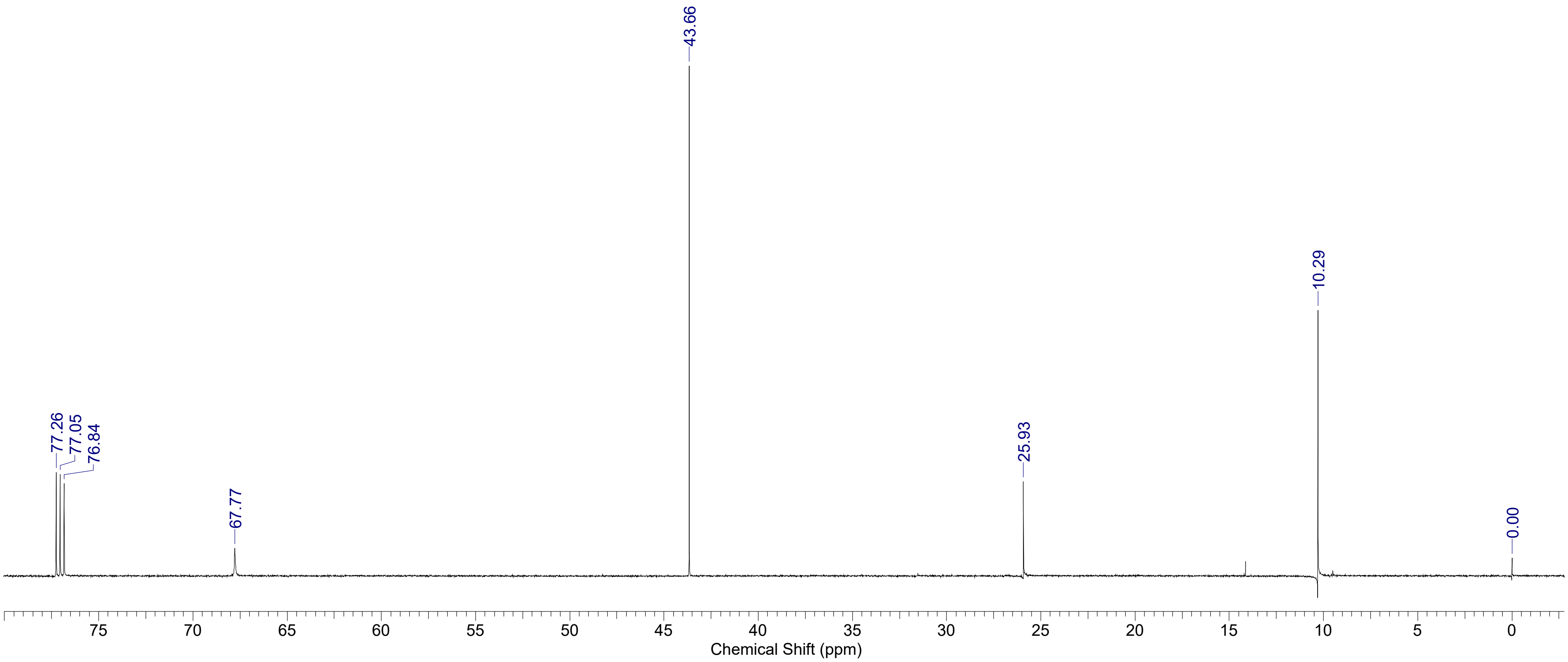

IR spectrum of compound **3a**

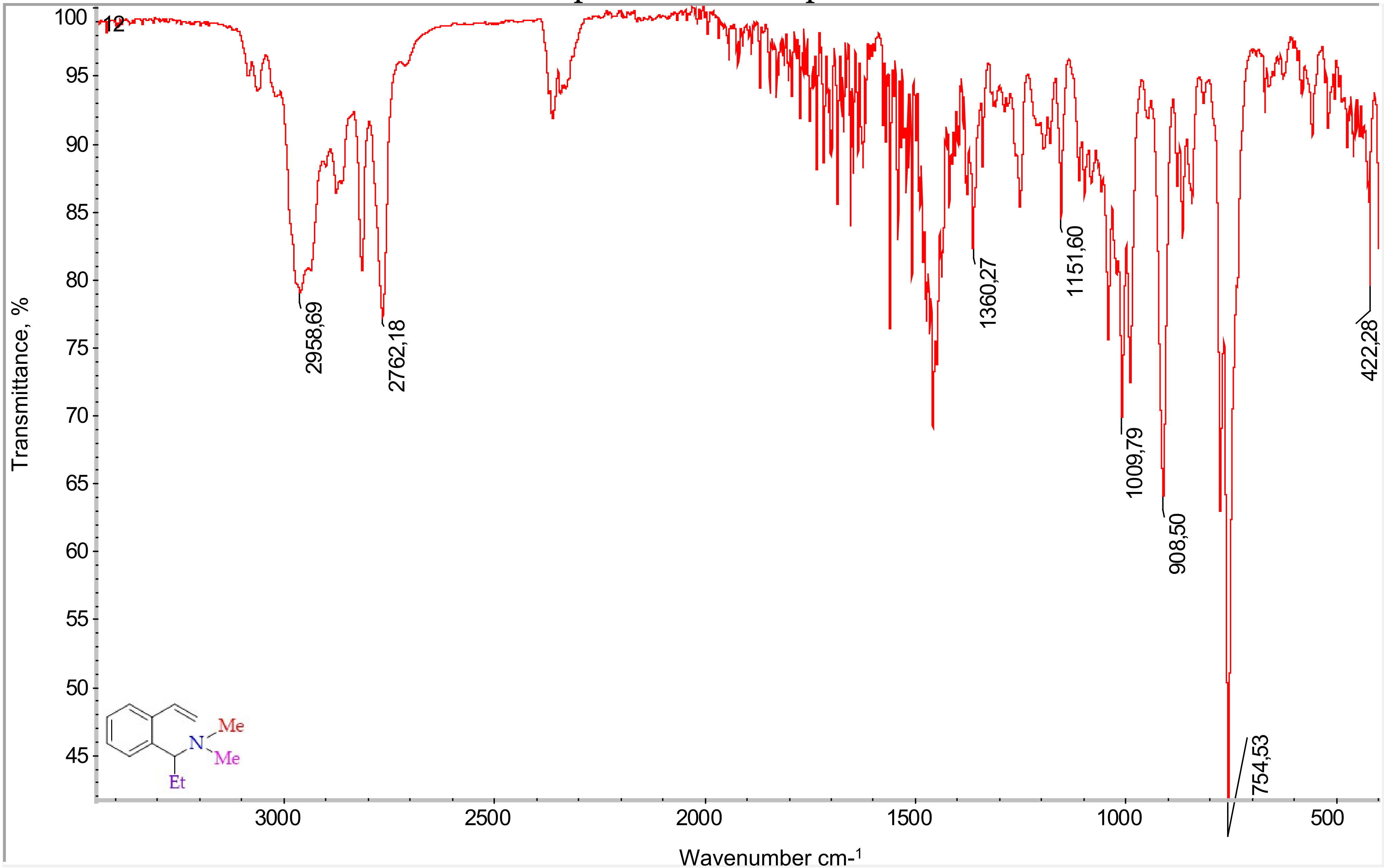

^{1}H NMR spectrum of compound **3b**

Acquisition Time (sec)	1.9818	***Comment***	single_pulse	***Date***			
Date Stamp							
File Name						***Frequency (MHz)***	600.17
Nucleus	1H	***Number of Transients***	8	***Origin***	ECA 600	***Original Points Count***	32768
Owner	CKP	***Points Count***	32768	***Pulse Sequence***	single_pulse.ex2	***Receiver Gain***	24.00
Solvent	CHLOROFORM-d	***Spectrum Offset (Hz)***	5392.4614	***Sweep Width (Hz)***	16534.39		

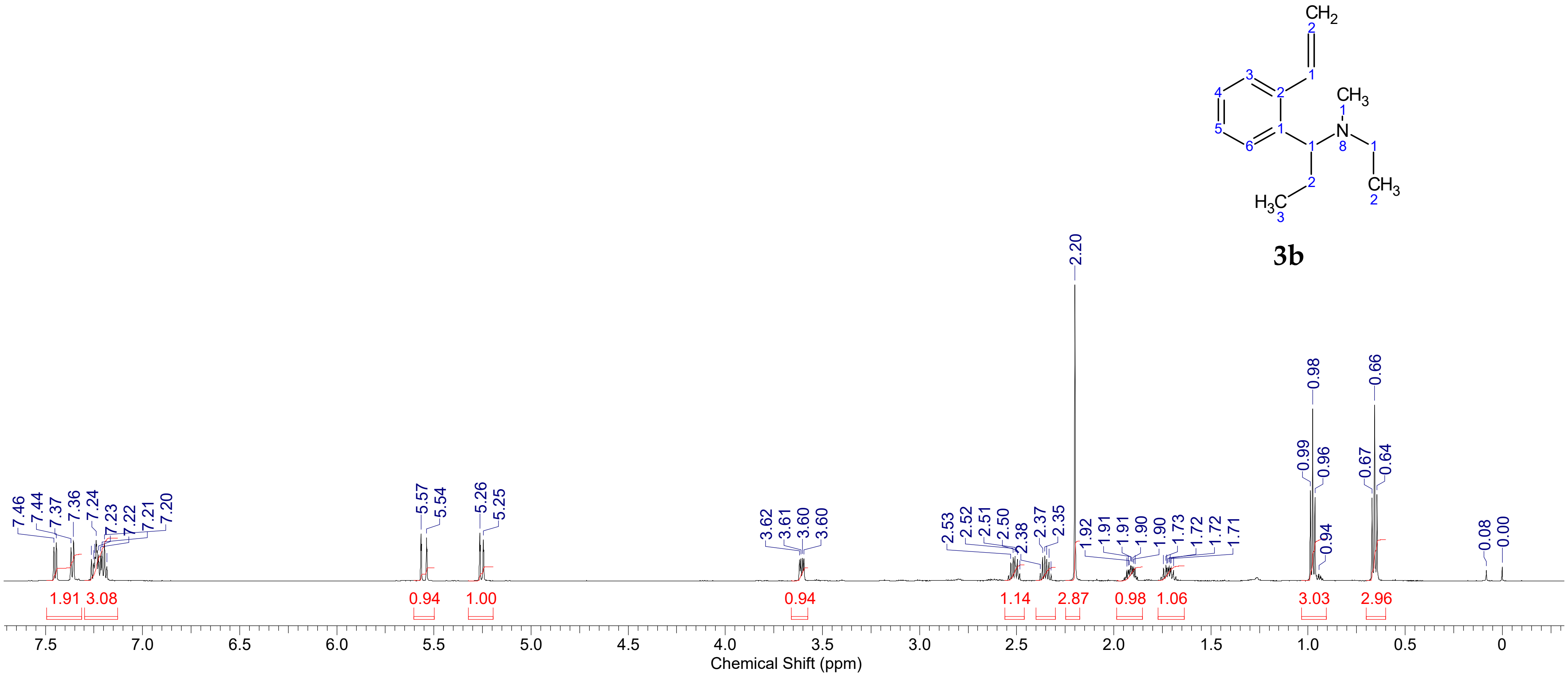

3b

^{1}H NMR spectrum of compound **3b**

Acquisition Time (sec)	1.9818	***Comment***	single_pulse	***Date***			
Date Stamp							
File Name						***Frequency (MHz)***	600.17
Nucleus	1H	***Number of Transients***	8	***Origin***	ECA 600	***Original Points Count***	32768
Owner	CKP	***Points Count***	32768	***Pulse Sequence***	single_pulse.ex2	***Receiver Gain***	24.00
Solvent	CHLOROFORM-d	***Spectrum Offset (Hz)***	5392.4614	***Sweep Width (Hz)***	16534.39		

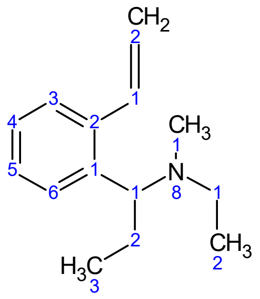

3b

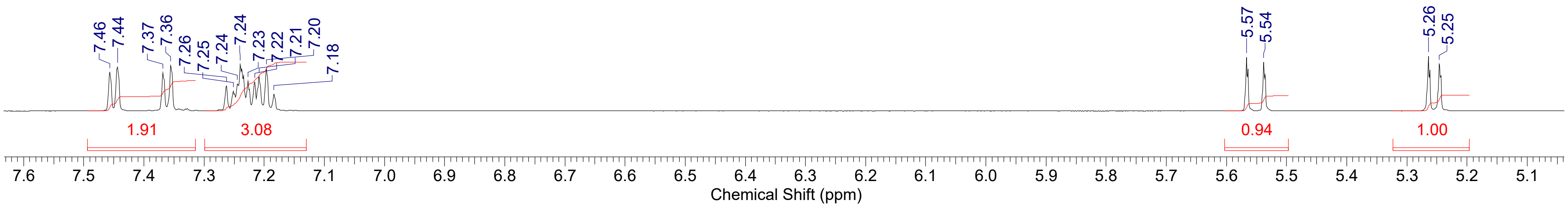

^{1}H NMR spectrum of compound **3b**

Acquisition Time (sec)	1.9818	***Comment***	single_pulse	***Date***			
Date Stamp							
File Name						***Frequency (MHz)***	600.17
Nucleus	1H	***Number of Transients***	8	***Origin***	ECA 600	***Original Points Count***	32768
Owner	CKP	***Points Count***	32768	***Pulse Sequence***	single_pulse.ex2	***Receiver Gain***	24.00
Solvent	CHLOROFORM-d	***Spectrum Offset (Hz)***	5392.4614	***Sweep Width (Hz)***	16534.39		

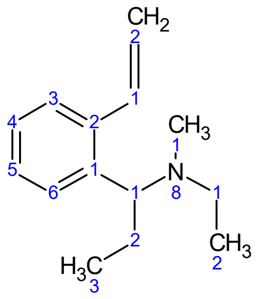

3b

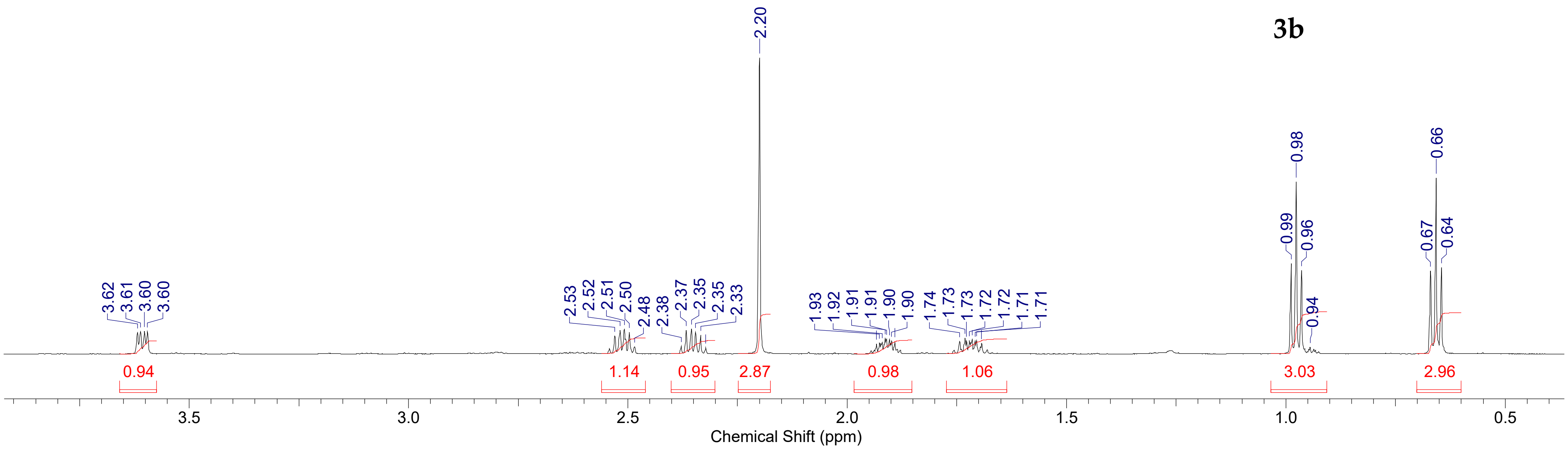

^{13}C NMR spectrum of compound **3b**

Acquisition Time (sec)	0.6921	***Comment***	single pulse decoupled gated NOE			***Date***	
Date Stamp							
File Name						***Frequency (MHz)***	150.91
Nucleus	13C	***Number of Transients***	1000	***Origin***	ECA 600	***Original Points Count***	32768
Owner	CKP	***Points Count***	32768	***Pulse Sequence***	single_pulse_dec	***Receiver Gain***	54.00
Solvent	CHLOROFORM-d	***Spectrum Offset (Hz)***	15091.3428	***Sweep Width (Hz)***	47348.49		

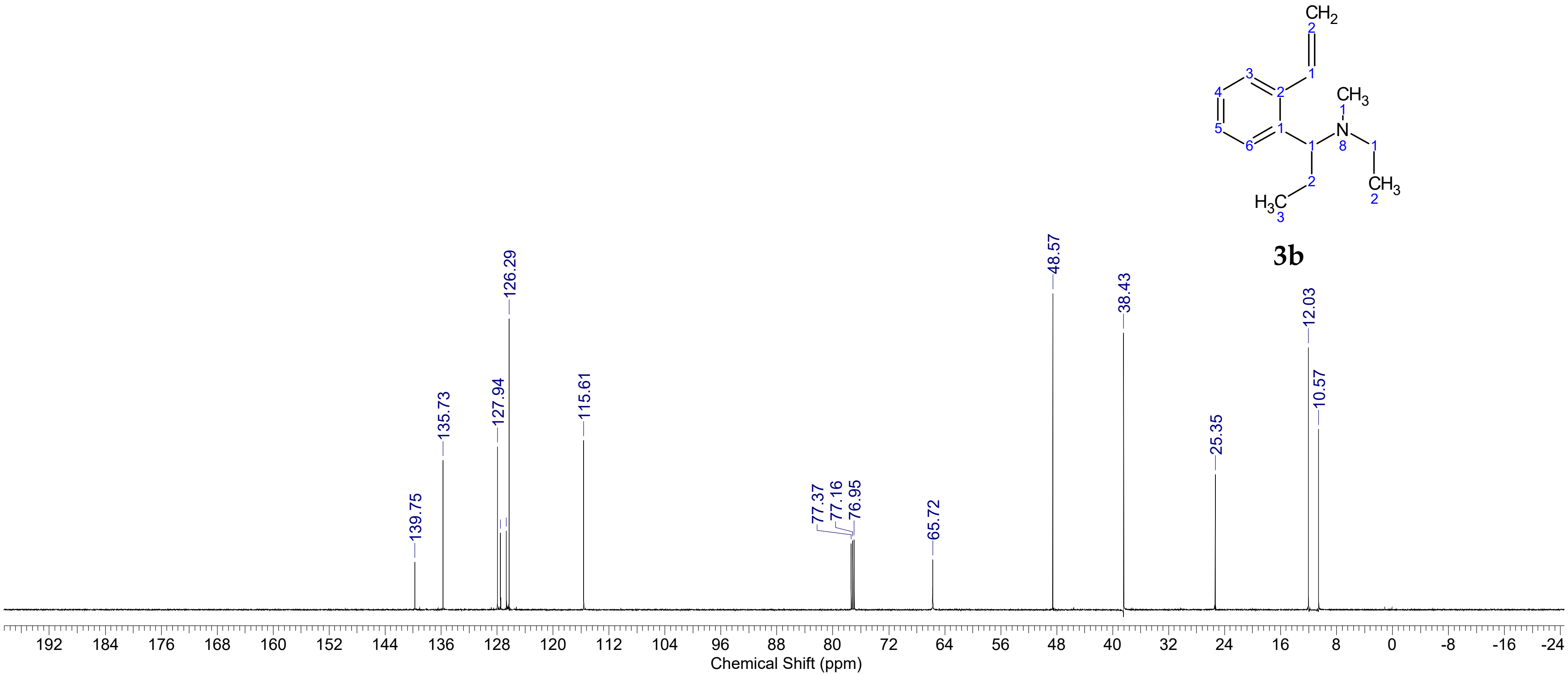

^{13}C NMR spectrum of compound **3b**

Acquisition Time (sec)	0.6921	***Comment***	single pulse decoupled gated NOE			***Date***	
Date Stamp							
File Name						***Frequency (MHz)***	150.91
Nucleus	13C	***Number of Transients***	1000	***Origin***	ECA 600	***Original Points Count***	32768
Owner	CKP	***Points Count***	32768	***Pulse Sequence***	single_pulse_dec	***Receiver Gain***	54.00
Solvent	CHLOROFORM-d	***Spectrum Offset (Hz)***	15091.3428	***Sweep Width (Hz)***	47348.49		

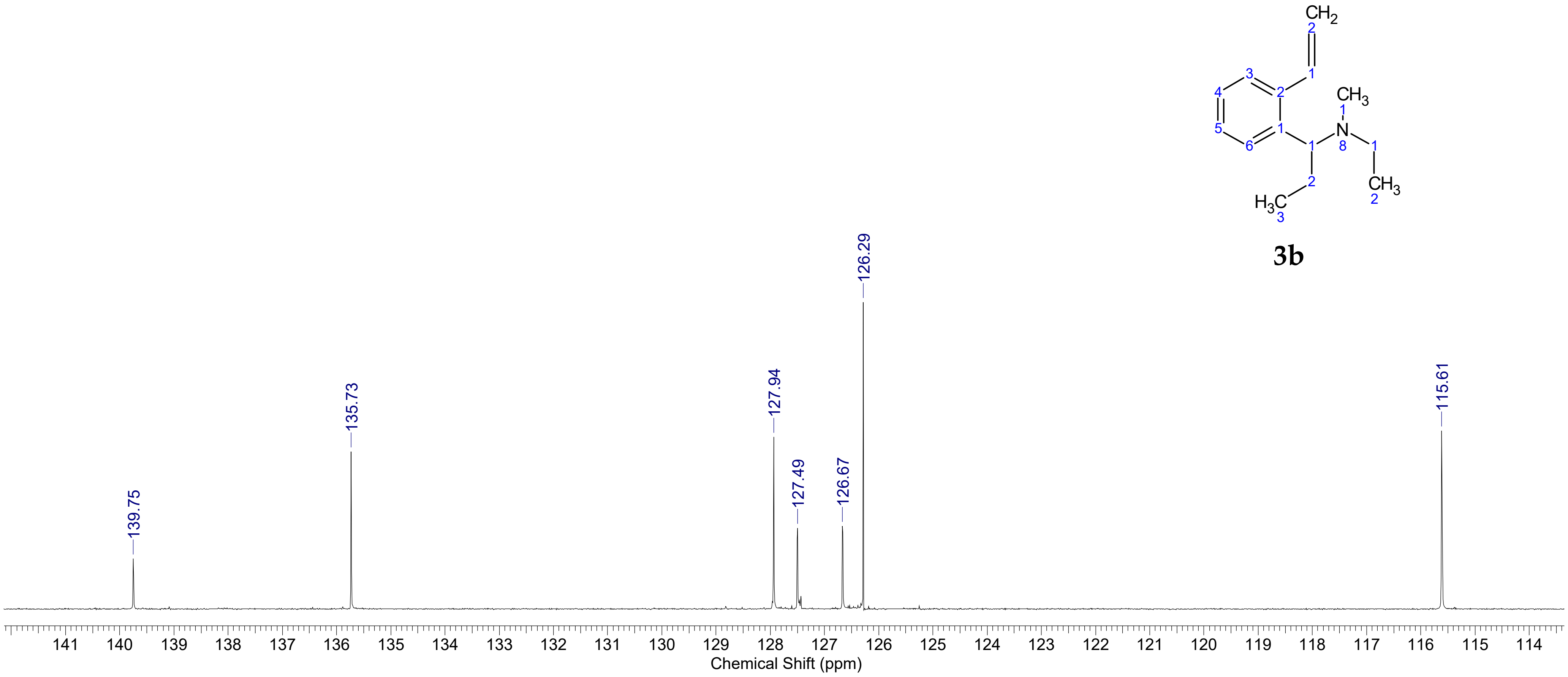

3b

^{13}C NMR spectrum of compound **3b**

Acquisition Time (sec)	0.6921	***Comment***	single pulse decoupled gated NOE			***Date***	
Date Stamp							
File Name						***Frequency (MHz)***	150.91
Nucleus	13C	***Number of Transients***	1000	***Origin***	ECA 600	***Original Points Count***	32768
Owner	CKP	***Points Count***	32768	***Pulse Sequence***	single_pulse_dec	***Receiver Gain***	54.00
Solvent	CHLOROFORM-d	***Spectrum Offset (Hz)***	14920.4023	***Sweep Width (Hz)***	47348.49		

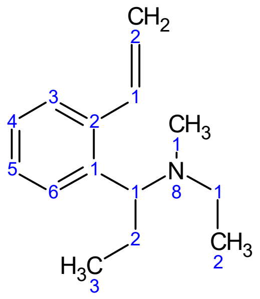

3b

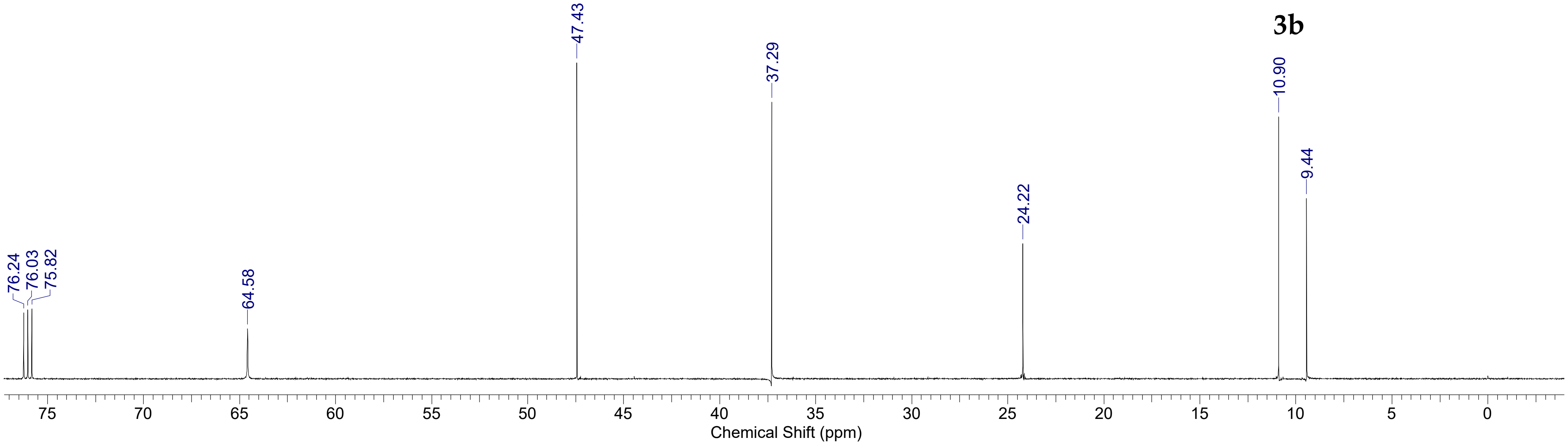

IR spectrum of compound **3b**

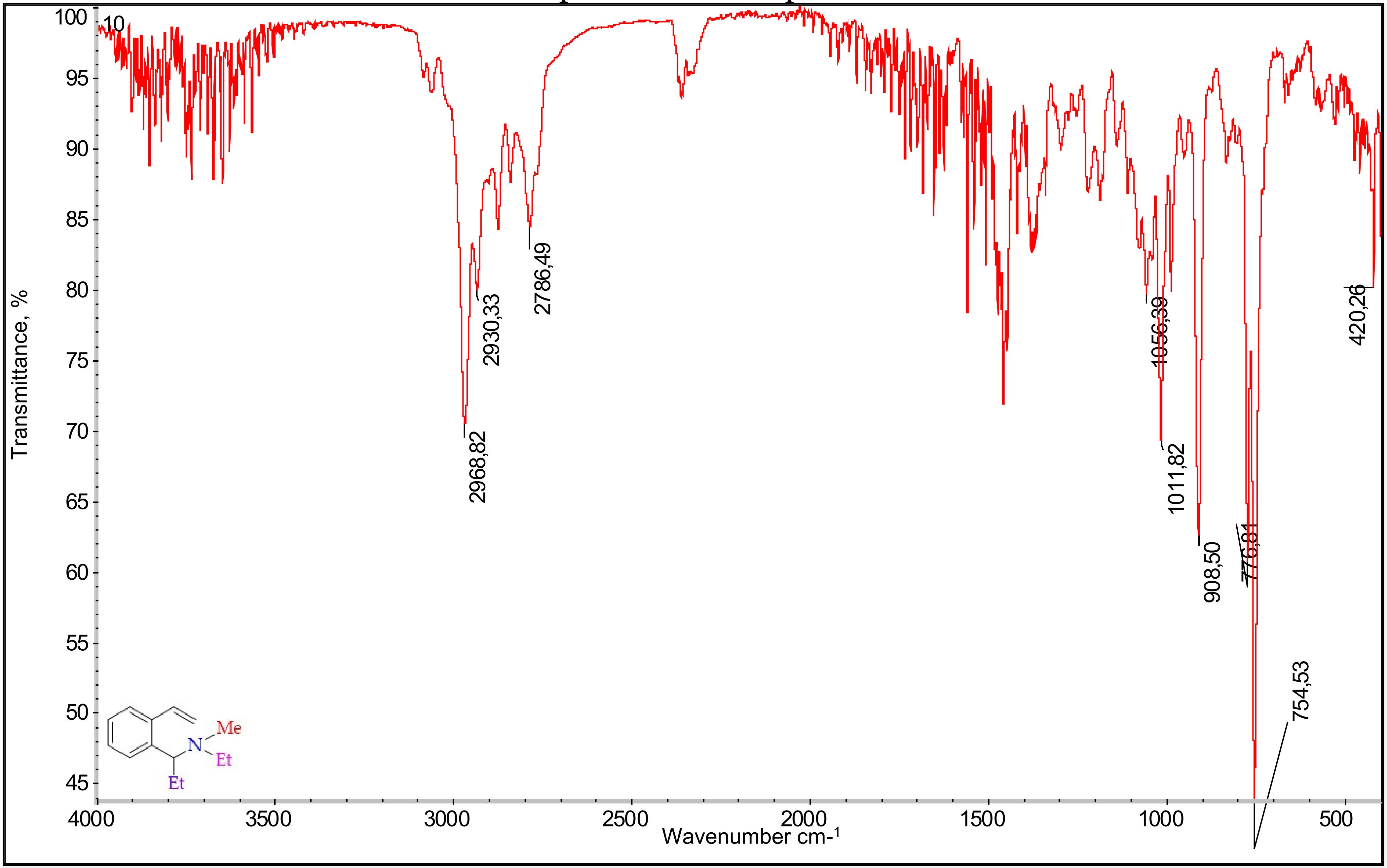

HRMS (MALDI-TOF) spectrum of compound **3b**

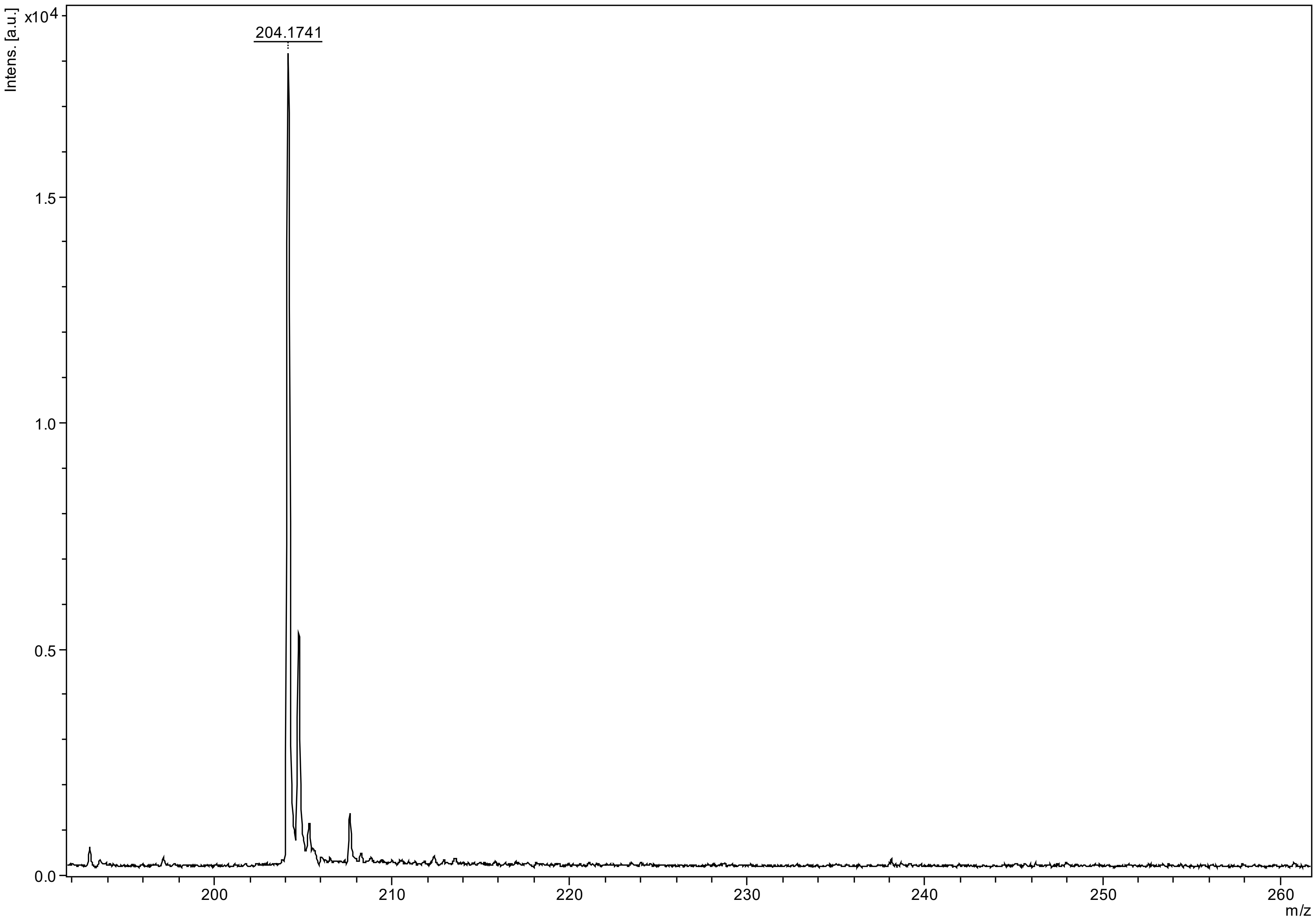

^{1}H NMR spectrum of compound **3c**

Acquisition Time (sec)	1.9818	Comment	single_pulse	Date			
Date Stamp				File Name			
Frequency (MHz)	600.17	Nucleus	1H	Number of Transients	8	Origin	ECA 600
Original Points Count	32768	Owner	CKP	Points Count	32768	Pulse Sequence	single_pulse.ex2
Receiver Gain	20.00	Solvent	CHLOROFORM-d	Spectrum Offset (Hz)	5388.4248	Sweep Width (Hz)	16534.39

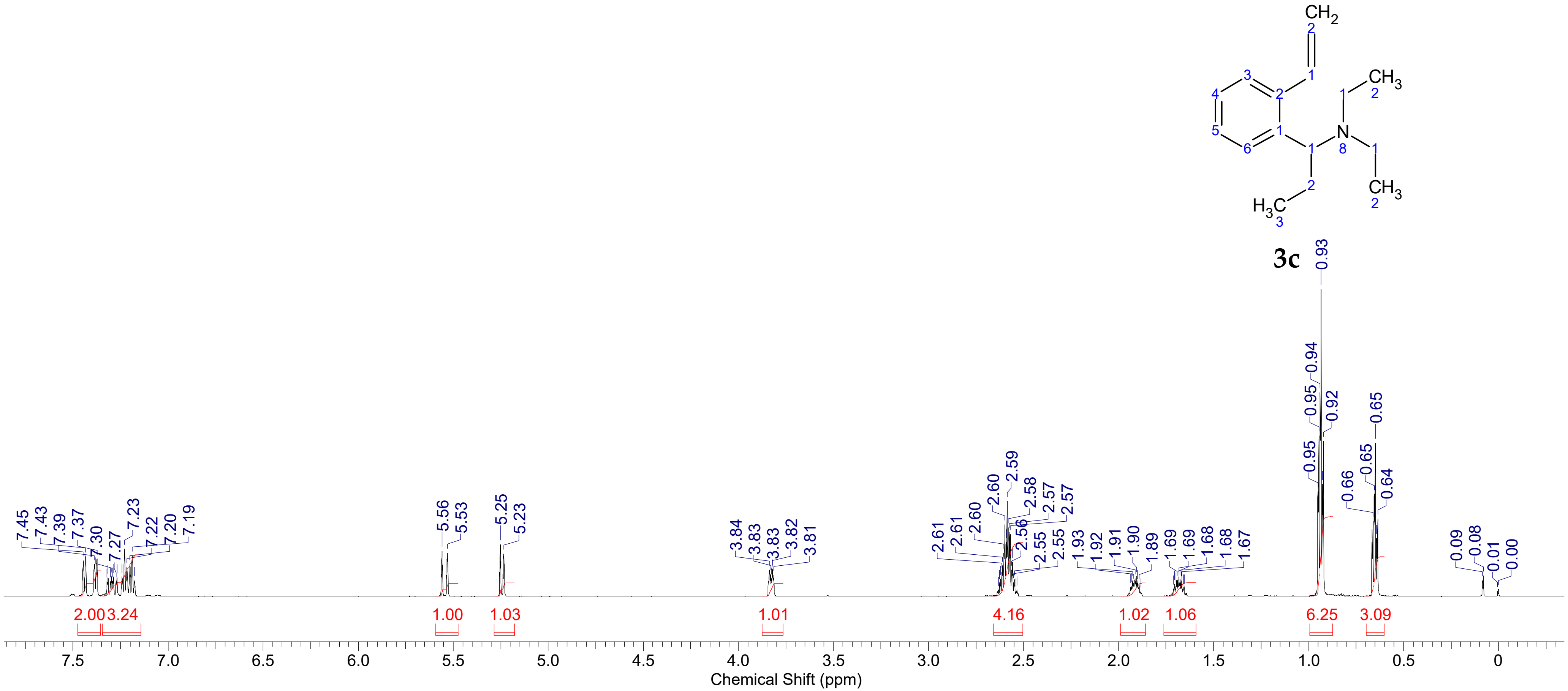

^{1}H NMR spectrum of compound **3c**

Acquisition Time (sec)	1.9818	***Comment***	single_pulse	***Date***			
Date Stamp				***File Name***			
Frequency (MHz)	600.17	***Nucleus***	1H	***Number of Transients***	8	***Origin***	ECA 600
Original Points Count	32768	***Owner***	CKP	***Points Count***	32768	***Pulse Sequence***	single_pulse.ex2
Receiver Gain	20.00	***Solvent***	CHLOROFORM-d	***Spectrum Offset (Hz)***	5388.4248	***Sweep Width (Hz)***	16534.39

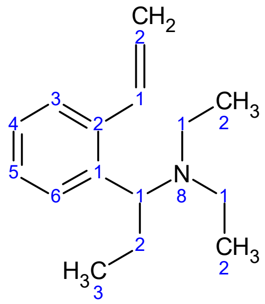

3c

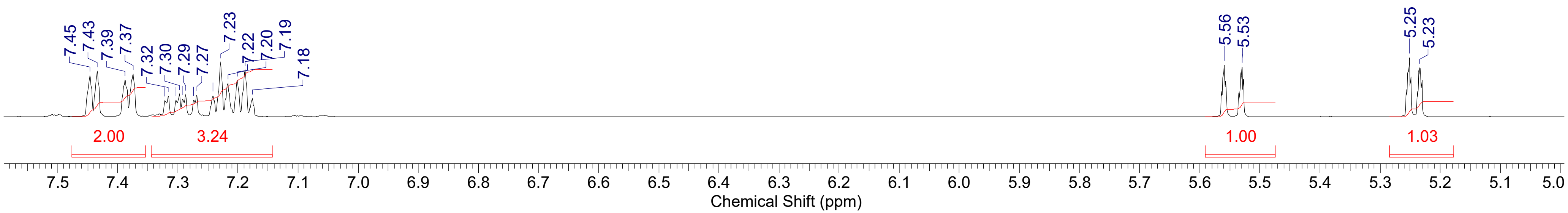

^{1}H NMR spectrum of compound **3c**

Acquisition Time (sec)	1.9818	***Comment***	single_pulse	***Date***			
Date Stamp				***File Name***			
Frequency (MHz)	600.17	***Nucleus***	1H	***Number of Transients***	8	***Origin***	ECA 600
Original Points Count	32768	***Owner***	CKP	***Points Count***	32768	***Pulse Sequence***	single_pulse.ex2
Receiver Gain	20.00	***Solvent***	CHLOROFORM-d	***Spectrum Offset (Hz)***	5388.4248	***Sweep Width (Hz)***	16534.39

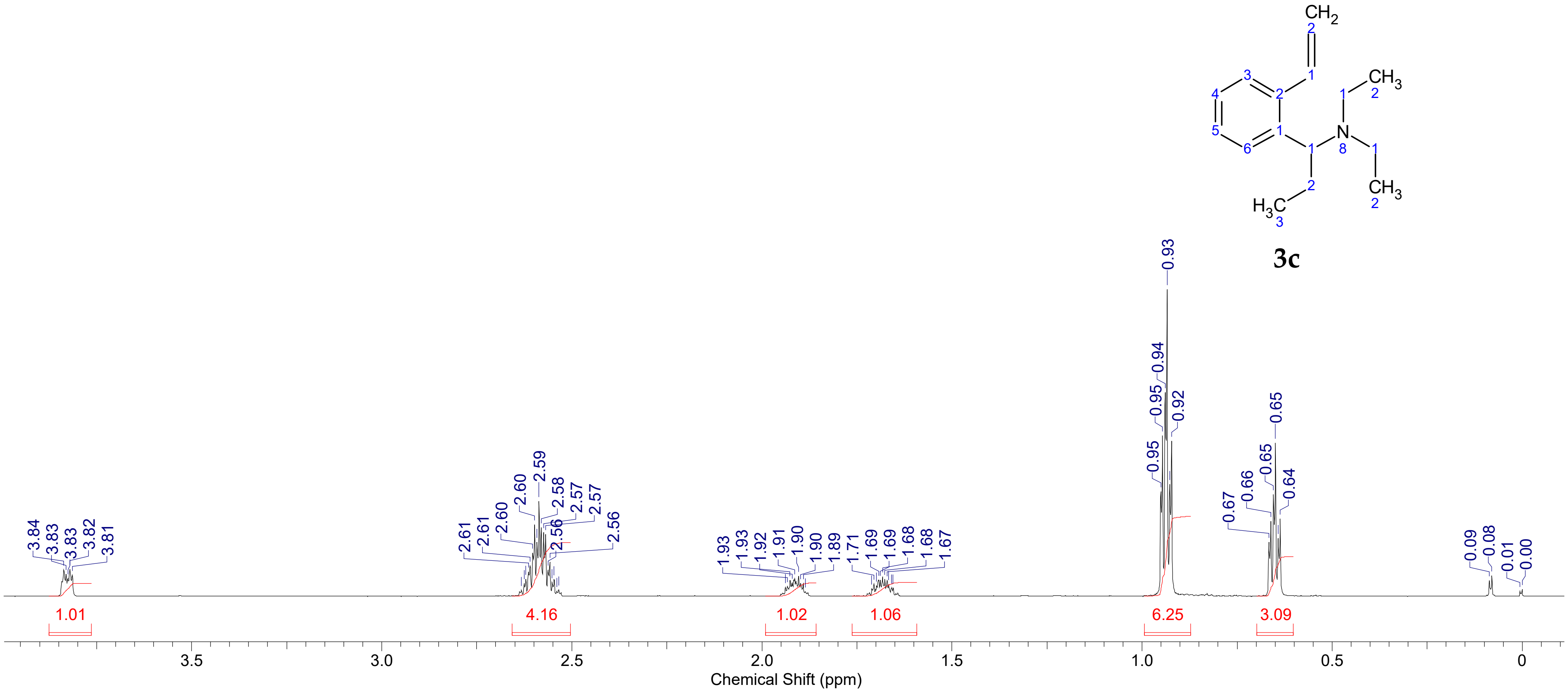

^{13}C NMR spectrum of compound **3c**

Acquisition Time (sec)	0.6921	Comment	single pulse decoupled gated NOE			Date	
Date Stamp							
File Name						Frequency (MHz)	150.91
Nucleus	13C	Number of Transients	1000	Origin	ECA 600	Original Points Count	32768
Owner	CKP	Points Count	32768	Pulse Sequence	single_pulse_dec	Receiver Gain	56.00
Solvent	CHLOROFORM-d	Spectrum Offset (Hz)	14918.9570	Sweep Width (Hz)	47348.49		

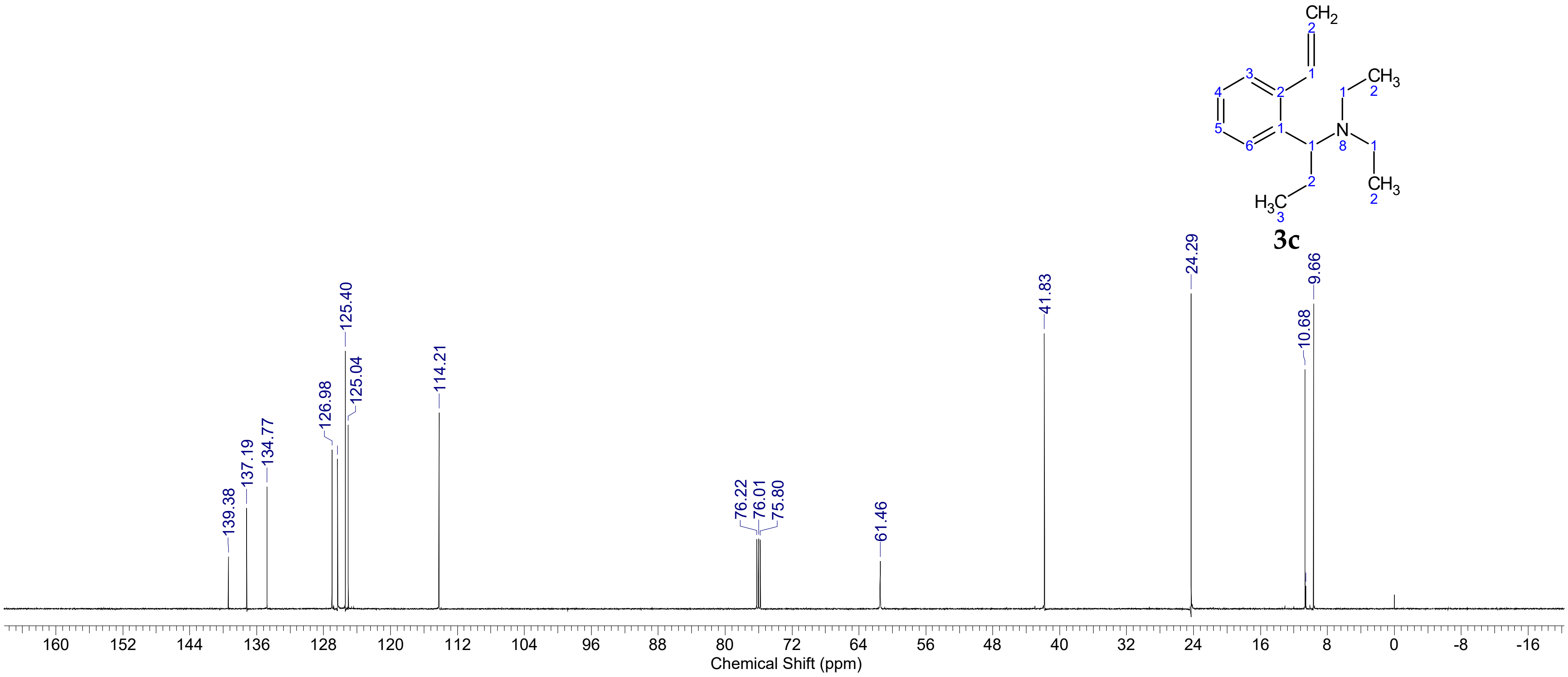

^{13}C NMR spectrum of compound **3c**

Acquisition Time (sec)	0.6921	***Comment***	single pulse decoupled gated NOE			***Date***	
Date Stamp							
File Name						***Frequency (MHz)***	150.91
Nucleus	13C	***Number of Transients***	1000	***Origin***	ECA 600	***Original Points Count***	32768
Owner	CKP	***Points Count***	32768	***Pulse Sequence***	single_pulse_dec	***Receiver Gain***	56.00
Solvent	CHLOROFORM-d	***Spectrum Offset (Hz)***	14918.9570	***Sweep Width (Hz)***	47348.49		

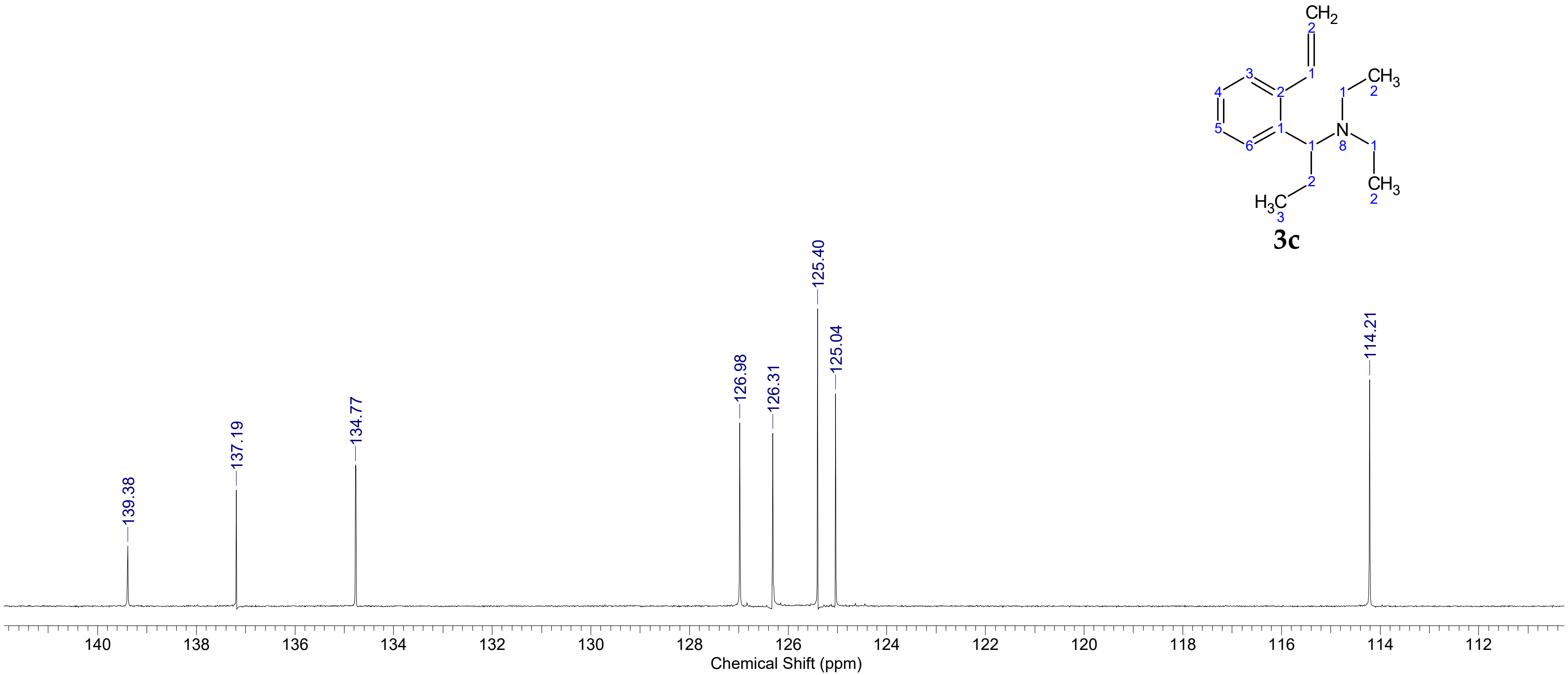

^{13}C NMR spectrum of compound **3c**

Acquisition Time (sec)	0.6921	***Comment***	single pulse decoupled gated NOE			***Date***	
Date Stamp							
File Name						***Frequency (MHz)***	150.91
Nucleus	13C	***Number of Transients***	1000	***Origin***	ECA 600	***Original Points Count***	32768
Owner	CKP	***Points Count***	32768	***Pulse Sequence***	single_pulse_dec	***Receiver Gain***	56.00
Solvent	CHLOROFORM-d	***Spectrum Offset (Hz)***	14918.9570	***Sweep Width (Hz)***	47348.49		

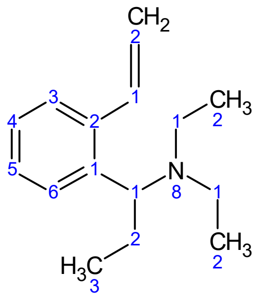

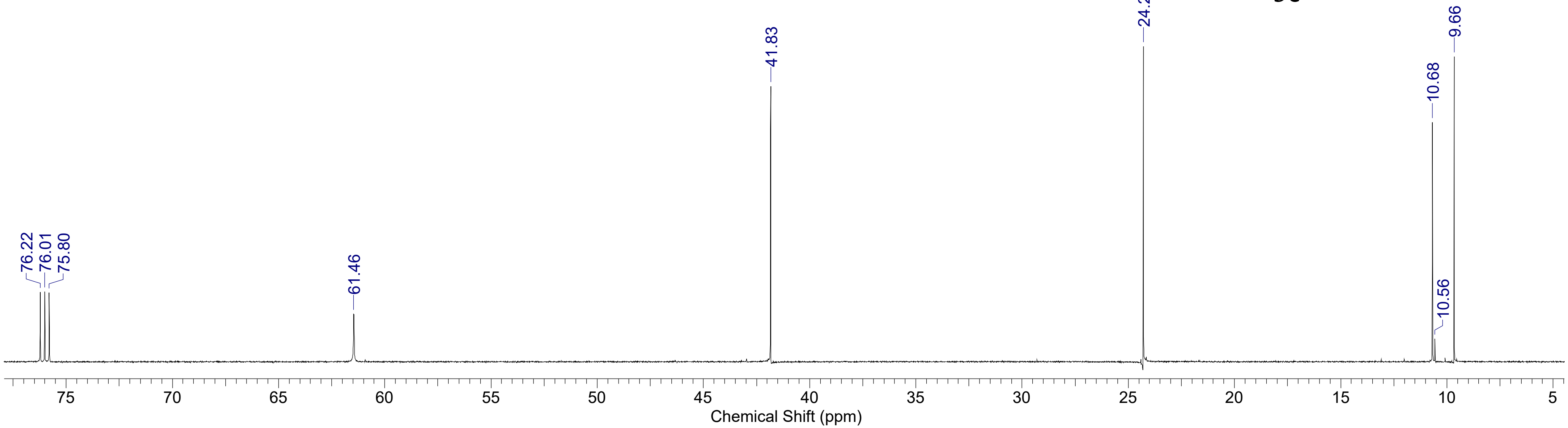

IR spectrum of compound **3c**

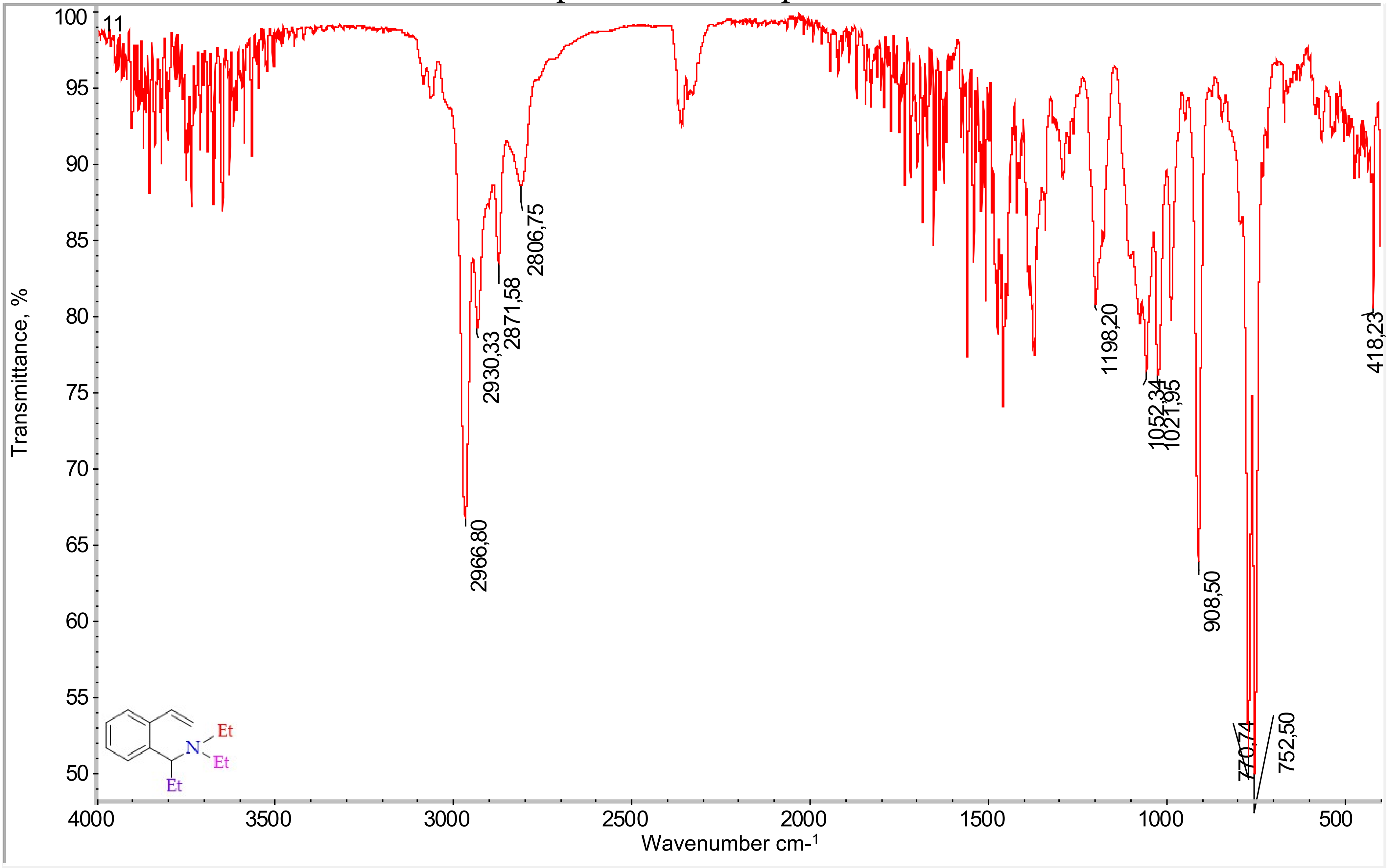

HRMS (MALDI-TOF) spectrum of compound **3c**

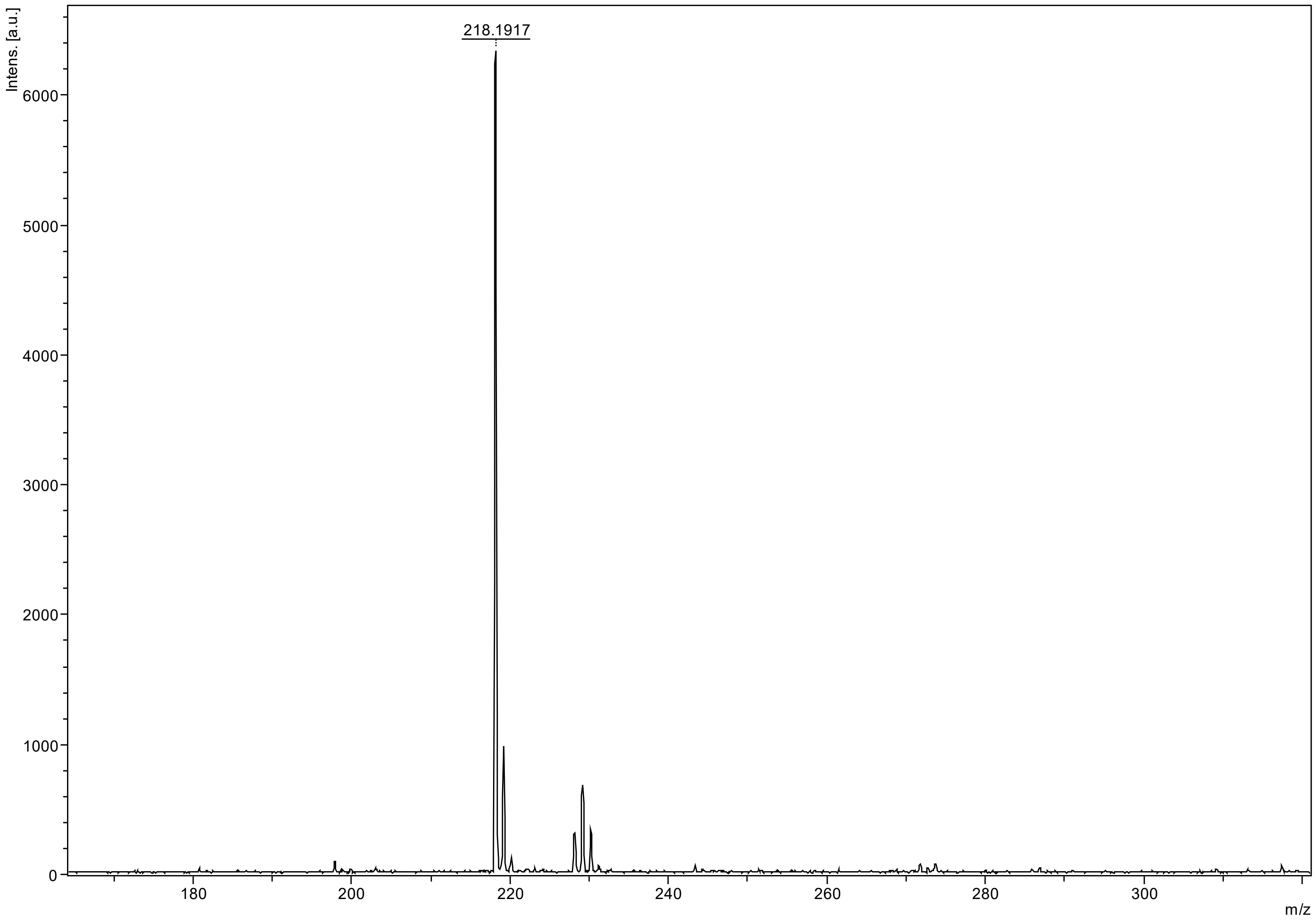

^{1}H NMR spectrum of compound **3d**

Acquisition Time (sec)	1.9818	***Comment***	single_pulse	***Date***			
Date Stamp							
File Name						***Frequency (MHz)***	600.17
Nucleus	1H	***Number of Transients***	8	***Origin***	ECA 600	***Original Points Count***	32768
Owner	CKP	***Points Count***	32768	***Pulse Sequence***	single_pulse.ex2	***Receiver Gain***	28.00
Solvent	CHLOROFORM-d	***Spectrum Offset (Hz)***	5401.5503	***Sweep Width (Hz)***	16534.39		

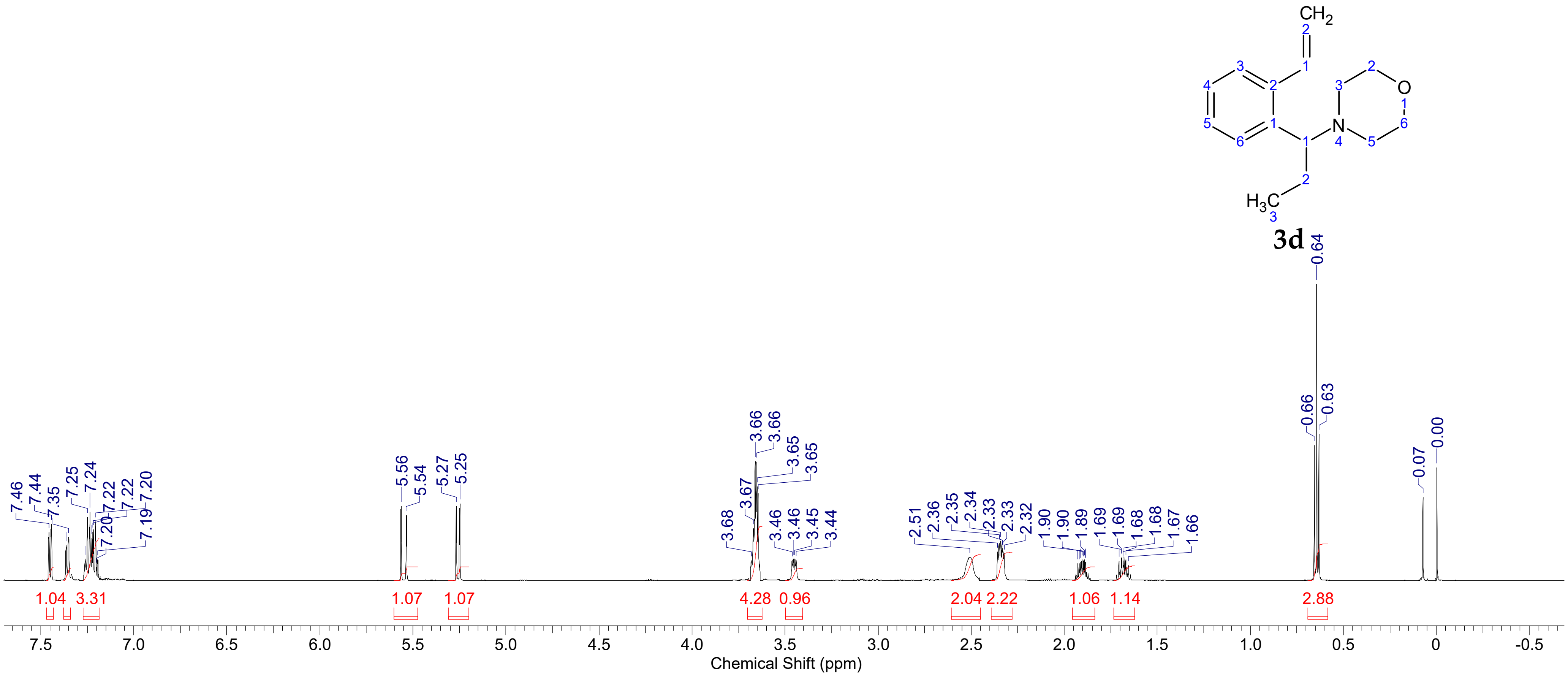

^1H NMR spectrum of compound **3d**

Acquisition Time (sec)	1.9818	***Comment***	single_pulse	***Date***			
Date Stamp							
File Name						***Frequency (MHz)***	600.17
Nucleus	1H	***Number of Transients***	8	***Origin***	ECA 600	***Original Points Count***	32768
Owner	CKP	***Points Count***	32768	***Pulse Sequence***	single_pulse.ex2	***Receiver Gain***	28.00
Solvent	CHLOROFORM-d	***Spectrum Offset (Hz)***	5401.5503	***Sweep Width (Hz)***	16534.39		

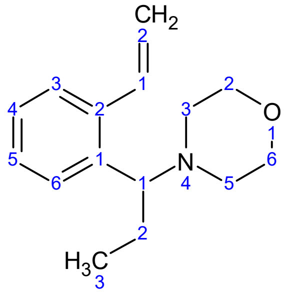

3d

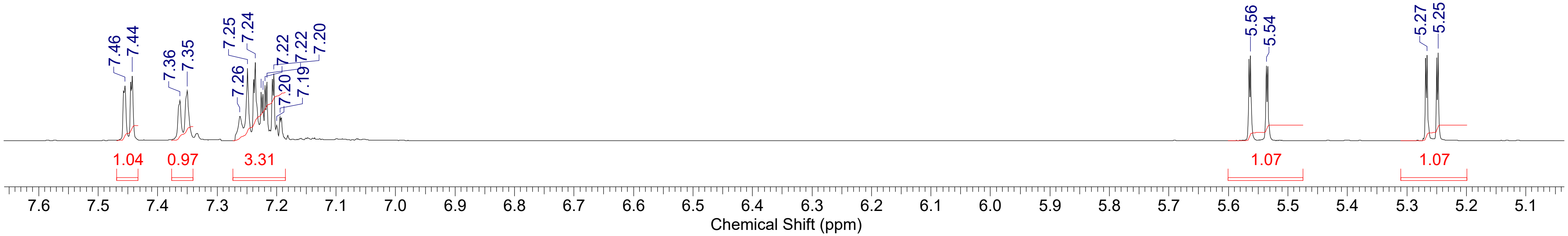

^{1}H NMR spectrum of compound **3d**

Acquisition Time (sec)	1.9818	***Comment***	single_pulse	***Date***			
Date Stamp							
File Name						***Frequency (MHz)***	600.17
Nucleus	1H	***Number of Transients***	8	***Origin***	ECA 600	***Original Points Count***	32768
Owner	CKP	***Points Count***	32768	***Pulse Sequence***	single_pulse.ex2	***Receiver Gain***	28.00
Solvent	CHLOROFORM-d	***Spectrum Offset (Hz)***	5401.5503	***Sweep Width (Hz)***	16534.39		

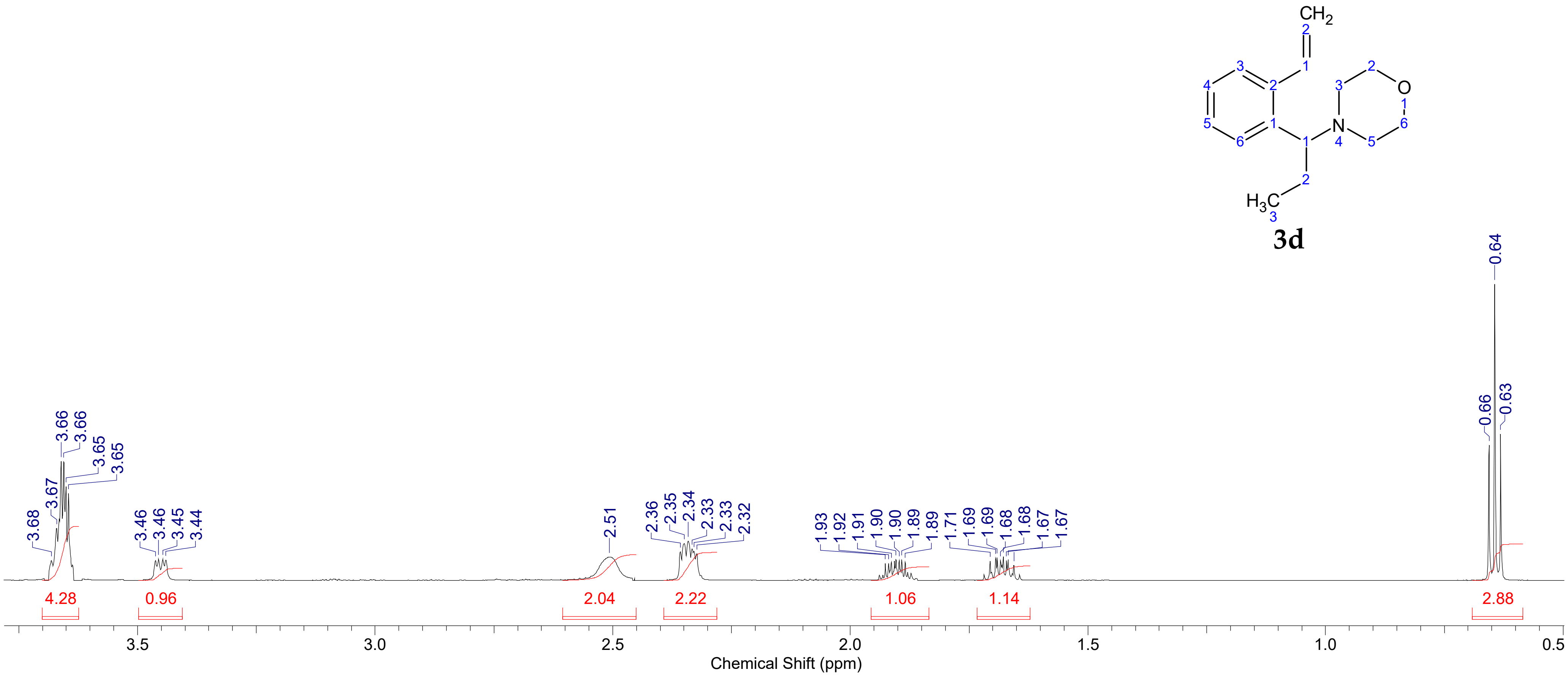

^{13}C NMR spectrum of compound **3d**

Acquisition Time (sec) 0.6921		***Comment***	single pulse decoupled gated NOE			***Date***	
Date Stamp							
File Name						***Frequency (MHz)***	150.91
Nucleus	13C	***Number of Transients***	1000	***Origin***	ECA 600	***Original Points Count***	32768
Owner	CKP	***Points Count***	32768	***Pulse Sequence***	single_pulse_dec	***Receiver Gain***	56.00
Solvent	CHLOROFORM-d	***Spectrum Offset (Hz)***	15076.4629	***Sweep Width (Hz)***	47348.49		

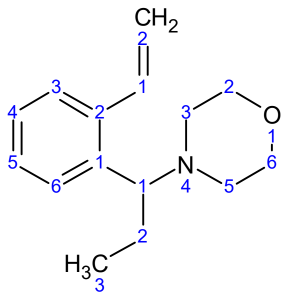

3d

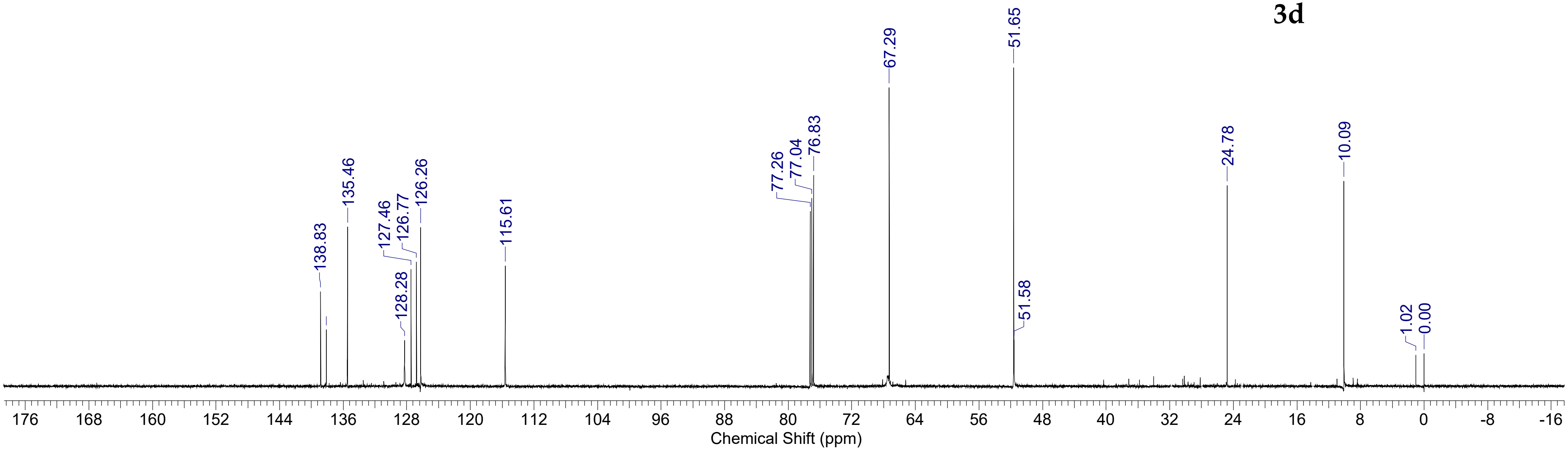

^{13}C NMR spectrum of compound **3d**

Acquisition Time (sec)	0.6921	***Comment***	single pulse decoupled gated NOE			***Date***	
Date Stamp							
File Name						***Frequency (MHz)***	150.91
Nucleus	13C	***Number of Transients***	1000	***Origin***	ECA 600	***Original Points Count***	32768
Owner	CKP	***Points Count***	32768	***Pulse Sequence***	single_pulse_dec	***Receiver Gain***	56.00
Solvent	CHLOROFORM-d	***Spectrum Offset (Hz)***	15076.4629	***Sweep Width (Hz)***	47348.49		

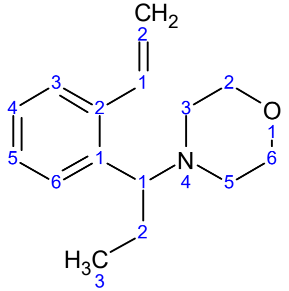

3d

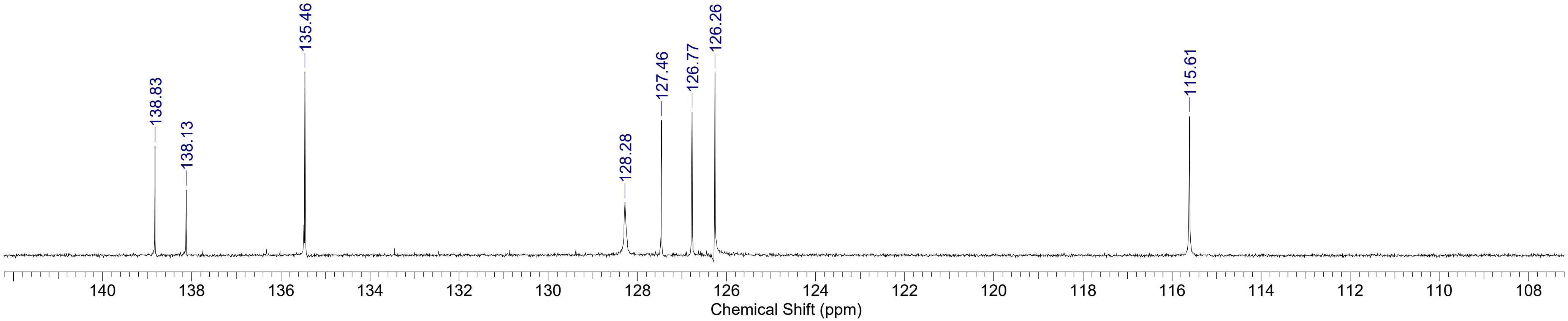

^{13}C NMR spectrum of compound **3d**

Acquisition Time (sec) 0.6921		***Comment***	single pulse decoupled gated NOE			***Date***	
Date Stamp							
File Name						***Frequency (MHz)***	150.91
Nucleus	13C	***Number of Transients***	1000	***Origin***	ECA 600	***Original Points Count***	32768
Owner	CKP	***Points Count***	32768	***Pulse Sequence***	single_pulse_dec	***Receiver Gain***	56.00
Solvent	CHLOROFORM-d	***Spectrum Offset (Hz)***	15076.4629	***Sweep Width (Hz)***	47348.49		

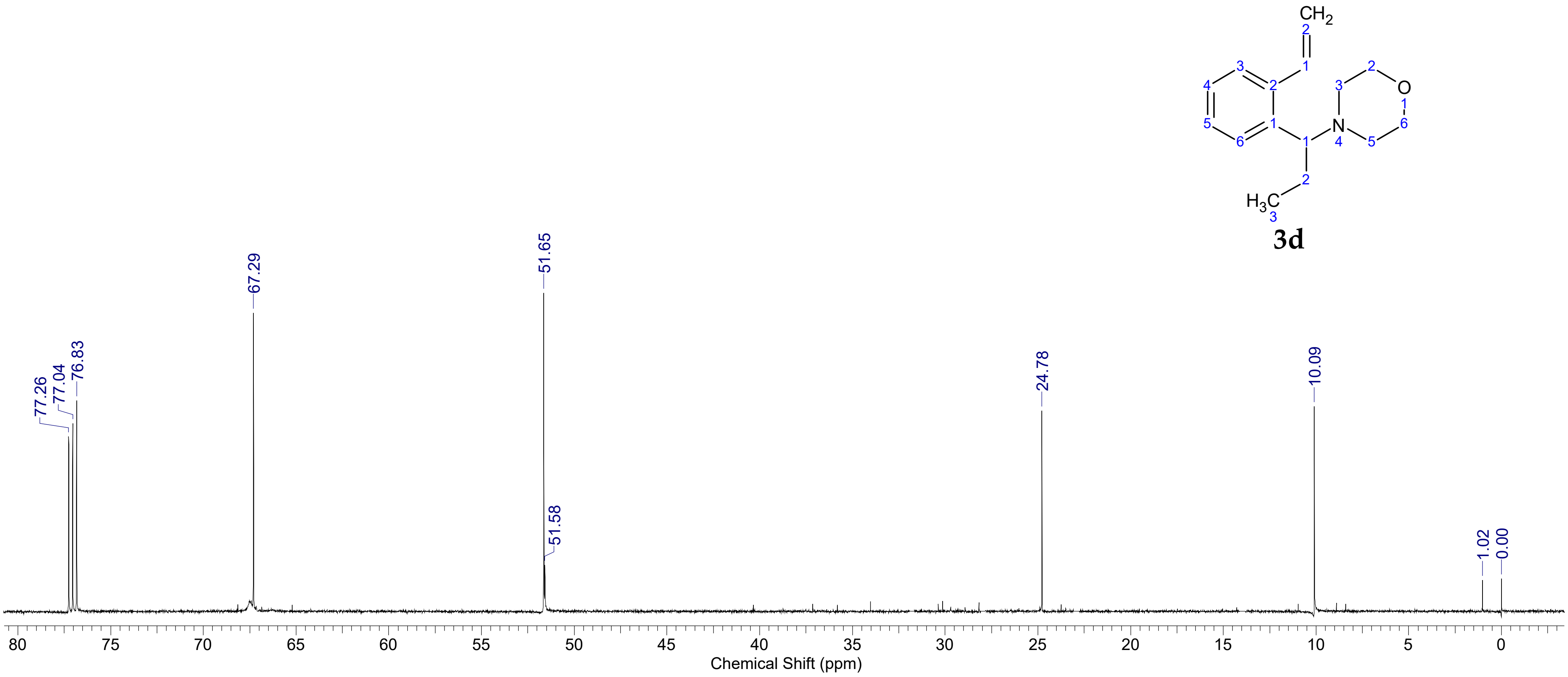

3d

IR spectrum of compound **3d**

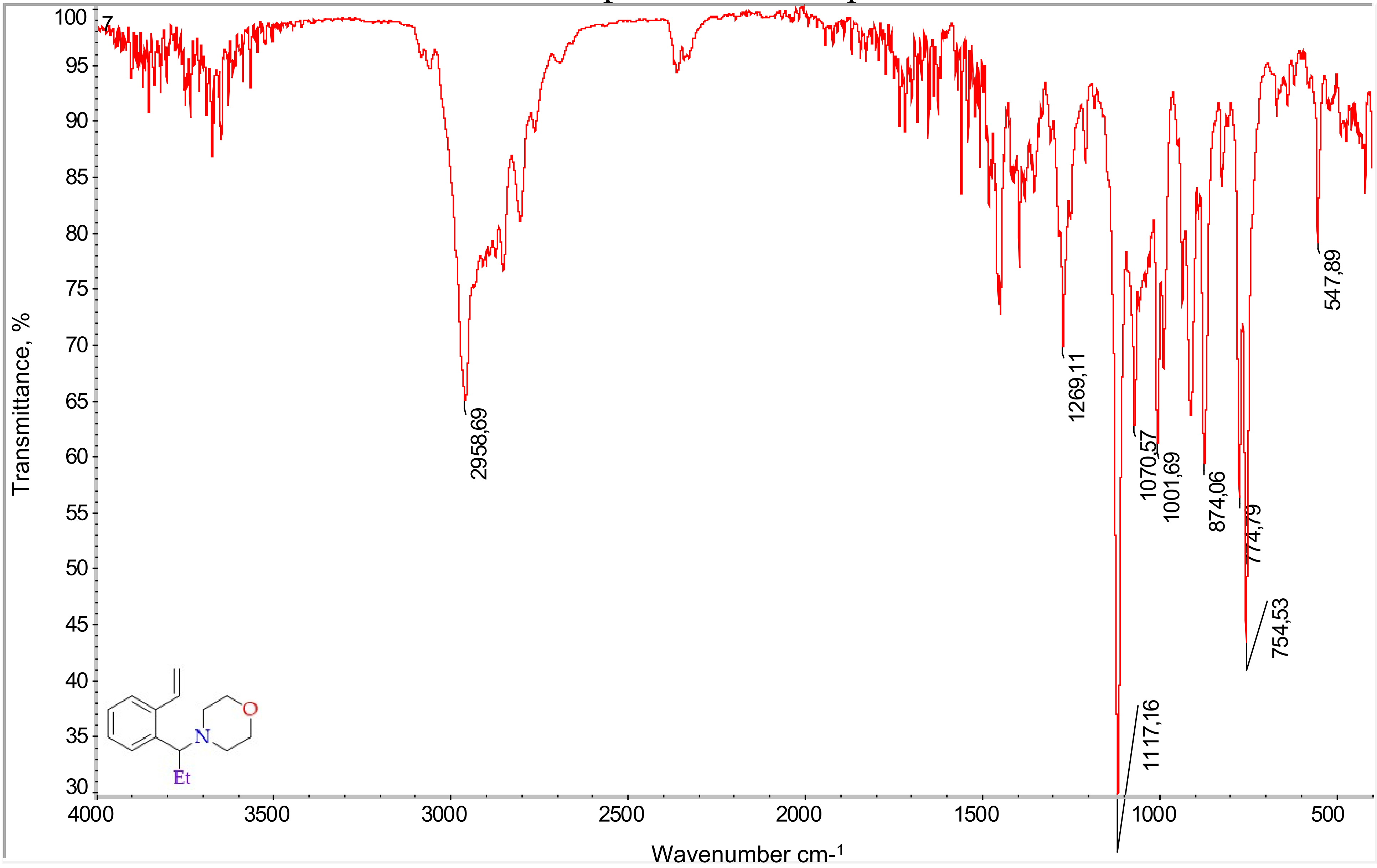

HRMS (MALDI-TOF) spectrum of compound **3d**

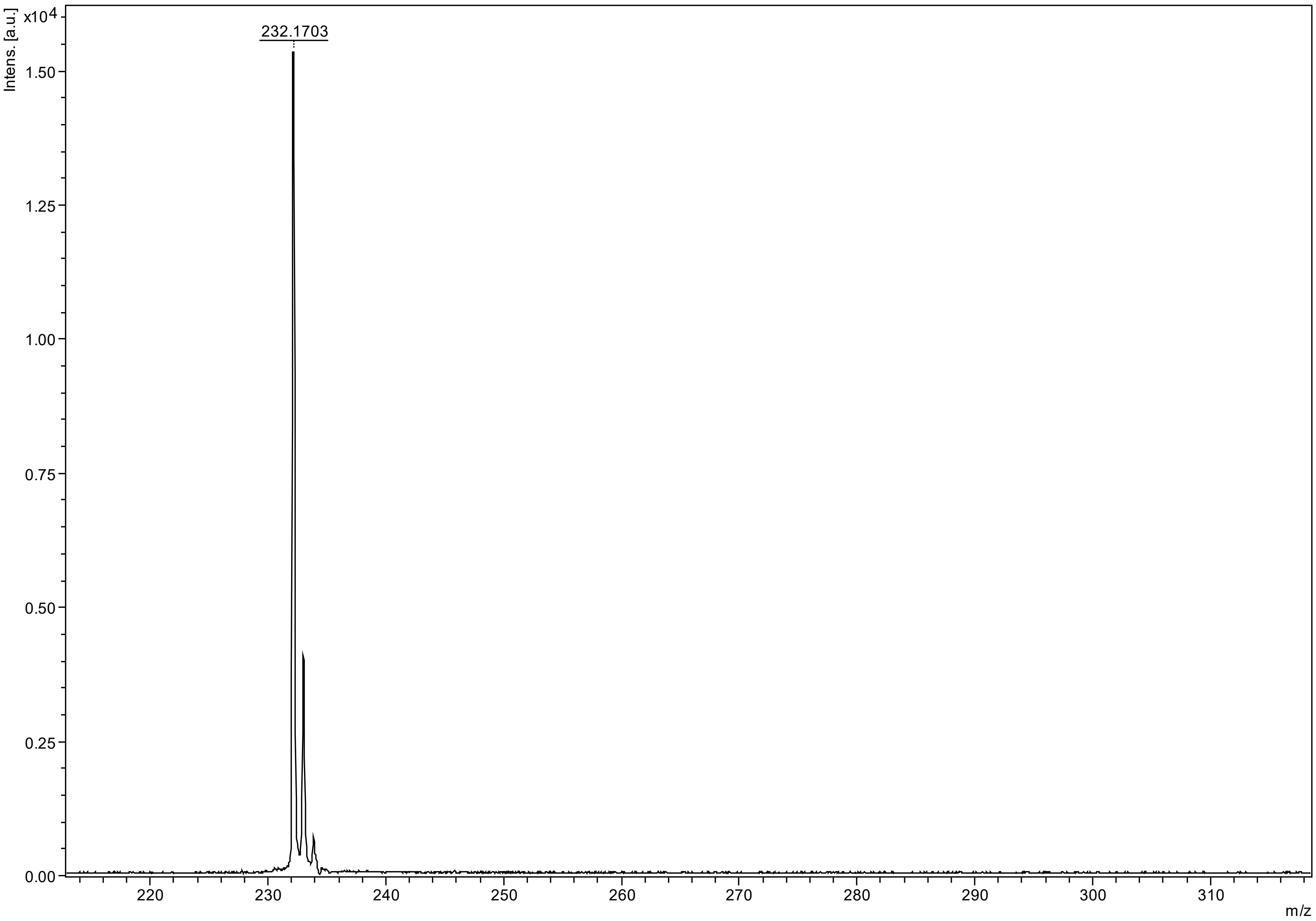

^1H spectrum of compound **4a**

Formula	$C_{32}H_{41}Cl_2N_3Ru$	FW	639.6629

Acquisition Time (sec)	2.5690	Comment	FZ_Grubbs_7-K8 in CD2Cl2	Date	30 Apr 2019 10:22:56
Date Stamp	30 Apr 2019 10:22:56			File Name	
Frequency (MHz)	300.13	Nucleus	1H	Number of Transients	64
Origin	spect	Original Points Count	65536	Owner	nmr
Points Count	262144	Pulse Sequence	zg	Receiver Gain	80.39
SW(cyclical) (Hz)	25510.20	Solvent	DICHLOROMETHANE-d2	Spectrum Offset (Hz)	1350.5919
Sweep Width (Hz)	25510.11	Temperature (degree C)	30.000		

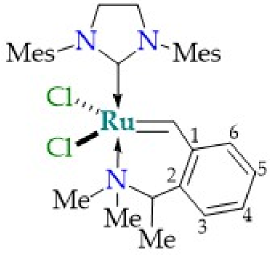

4a

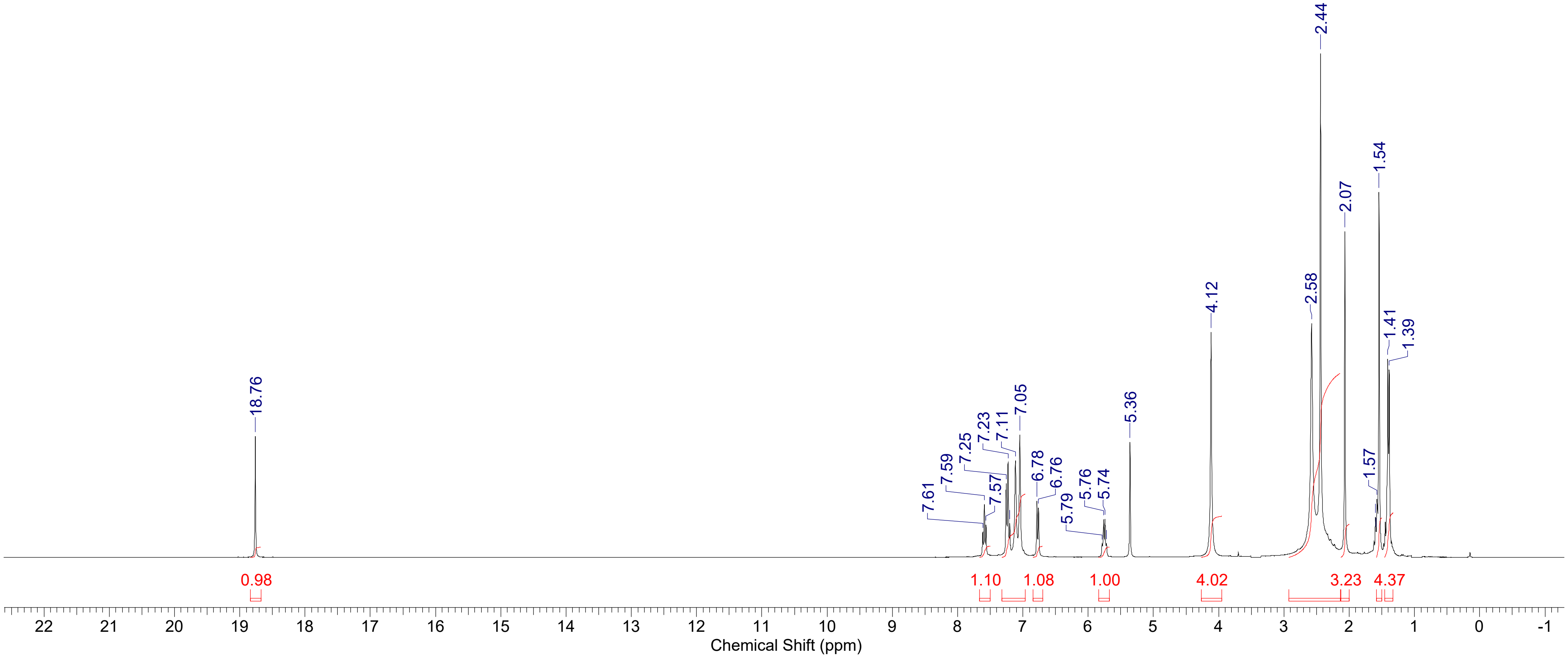

^{1}H spectrum of compound **4a**

Formula	$C_{32}H_{41}Cl_2N_3Ru$	FW	639.6629

Acquisition Time (sec)	2.5690	Comment	FZ_Grubbs_7-K8 in CD2Cl2			Date	30 Apr 2019 10:22:56
Date Stamp	30 Apr 2019 10:22:56			File Name			
Frequency (MHz)	300.13	Nucleus	1H	Number of Transients	64	Origin	spect
Original Points Count	65536	Owner	nmr	Points Count	262144	Pulse Sequence	zg
Receiver Gain	80.39	SW(cyclical) (Hz)	25510.20	Solvent	DICHLOROMETHANE-d2		
Spectrum Offset (Hz)	1350.5919	Sweep Width (Hz)	25510.11	Temperature (degree C)	30.000		

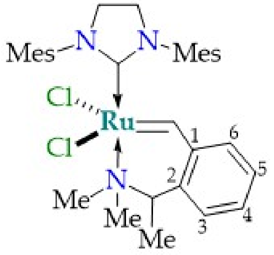

4a

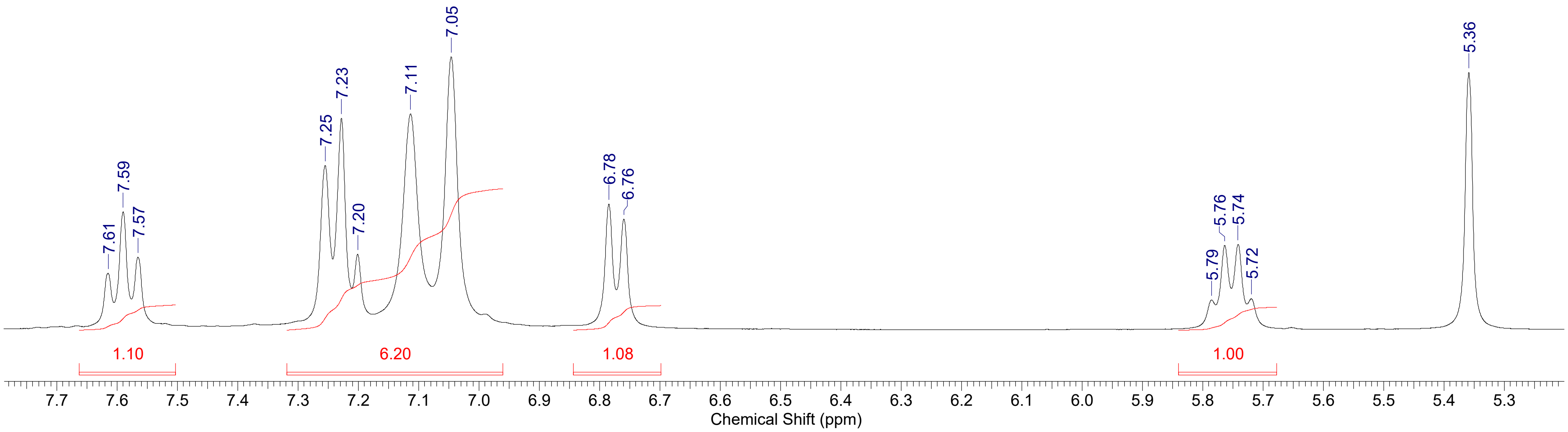

^{1}H spectrum of compound **4a**

Formula	$C_{32}H_{41}Cl_2N_3Ru$	FW	639.6629

Acquisition Time (sec)	2.5690	Comment	FZ_Grubbs_7-K8 in CD2Cl2	Date	30 Apr 2019 10:22:56
Date Stamp	30 Apr 2019 10:22:56	File Name			
Frequency (MHz)	300.13	Nucleus	1H	Number of Transients	64	Origin	spect
Original Points Count	65536	Owner	nmr	Points Count	262144	Pulse Sequence	zg
Receiver Gain	80.39	SW(cyclical) (Hz)	25510.20	Solvent	DICHLOROMETHANE-d2		
Spectrum Offset (Hz)	1350.5919	Sweep Width (Hz)	25510.11	Temperature (degree C)	30.000		

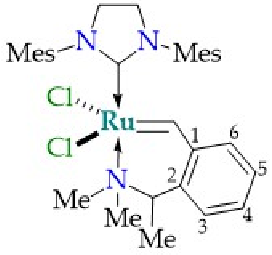

4a

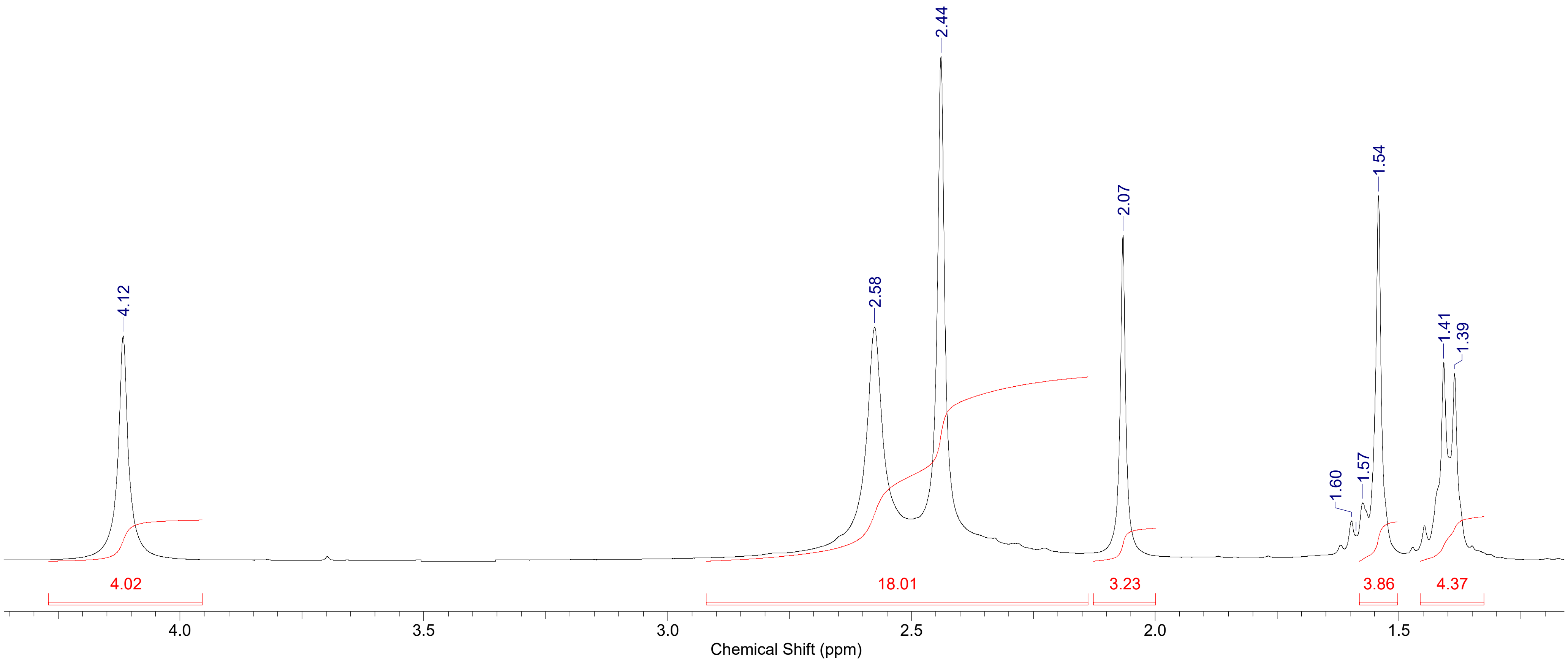

^{13}C spectrum of compound **4a**

Formula	$C_{32}H_{41}Cl_2N_3Ru$	***FW***	639.6629

Acquisition Time (sec)	1.1010	***Comment***	FZ_Grubbs_7-K8-C13dec [hp-dec]			***Date***	30 Apr 2019 12:39:28
Date Stamp	30 Apr 2019 12:39:28			***File Name***			
Frequency (MHz)	75.47	***Nucleus***	13C	***Number of Transients***	2224	***Origin***	spect
Original Points Count	65536	***Owner***	nmr	***Points Count***	262144	***Pulse Sequence***	zgpg
Receiver Gain	202.48	***SW(cyclical) (Hz)***	59523.81	***Solvent***	DICHLOROMETHANE-d2		
Spectrum Offset (Hz)	7546.7783	***Sweep Width (Hz)***	59523.58	***Temperature (degree C)***	29.987		

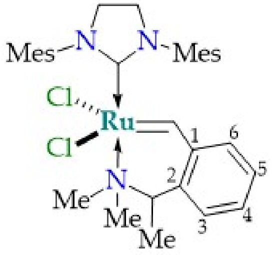

4a

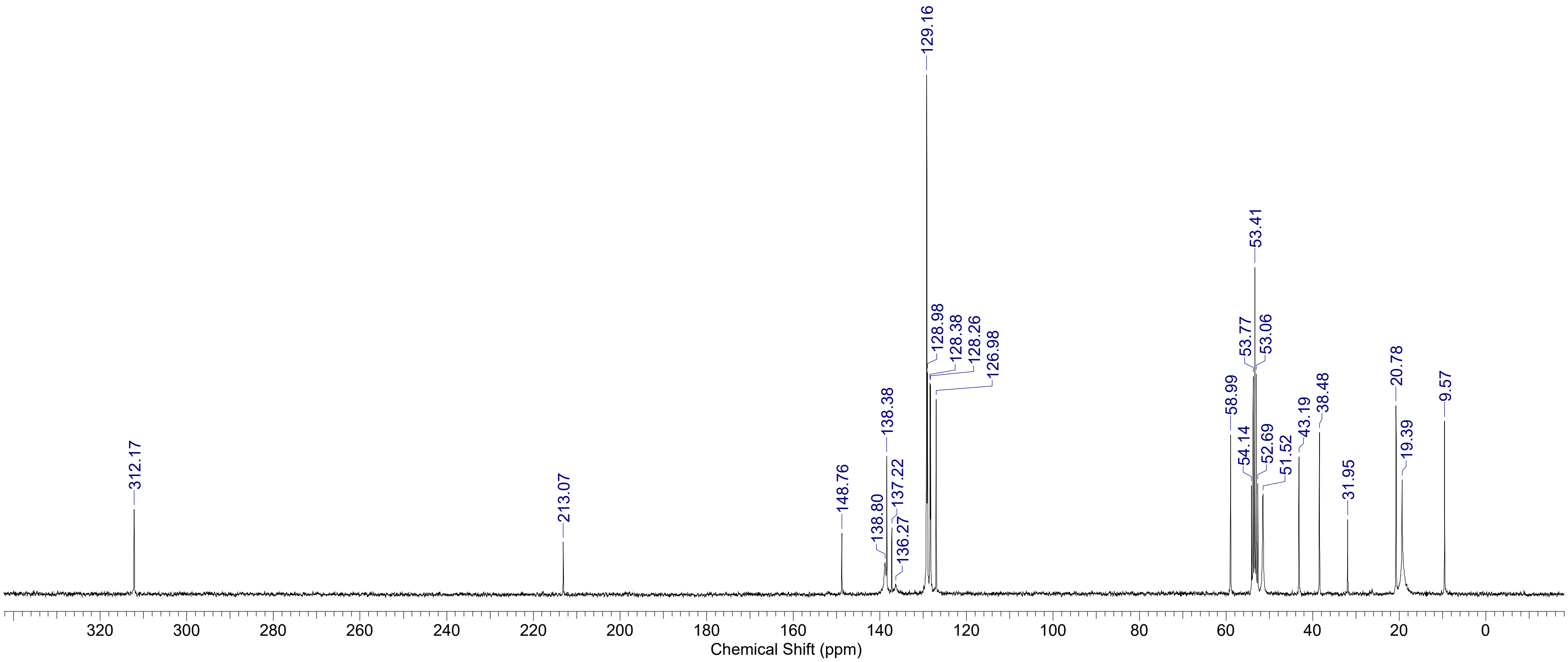

^{13}C spectrum of compound **4a**

**Formula**	$C_{32}H_{41}Cl_2N_3Ru$	**FW**	639.6629

**Acquisition Time (sec)**	1.1010	**Comment**	FZ_Grubbs_7-K8-C13dec [hp-dec]			**Date**	30 Apr 2019 12:39:28
**Date Stamp**	30 Apr 2019 12:39:28			**File Name**			
**Frequency (MHz)**	75.47	**Nucleus**	13C	**Number of Transients**	2224	**Origin**	spect
**Original Points Count**	65536	**Owner**	nmr	**Points Count**	262144	**Pulse Sequence**	zgpg
**Receiver Gain**	202.48	**SW(cyclical) (Hz)**	59523.81	**Solvent**	DICHLOROMETHANE-d2		
**Spectrum Offset (Hz)**	7546.7783	**Sweep Width (Hz)**	59523.58	**Temperature (degree C)**	29.987		

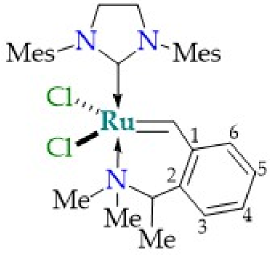

4a

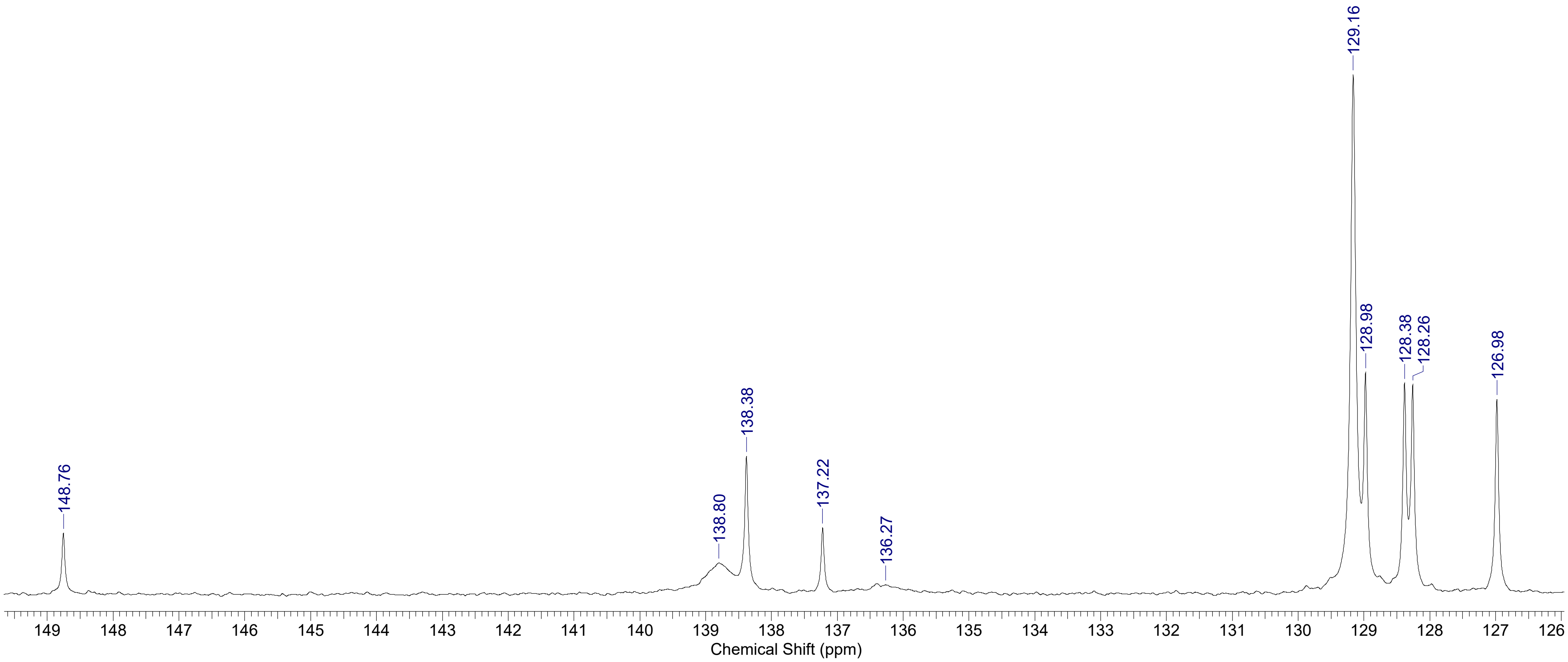

^{13}C spectrum of compound **4a**

Formula	$C_{32}H_{41}Cl_2N_3Ru$	***FW***	639.6629

Acquisition Time (sec)	1.1010	***Comment***	FZ_Grubbs_7-K8-C13dec [hp-dec]			***Date***	30 Apr 2019 12:39:28
Date Stamp	30 Apr 2019 12:39:28			***File Name***			
Frequency (MHz)	75.47	***Nucleus***	13C	***Number of Transients***	2224	***Origin***	spect
Original Points Count	65536	***Owner***	nmr	***Points Count***	262144	***Pulse Sequence***	zgpg
Receiver Gain	202.48	***SW(cyclical) (Hz)***	59523.81	***Solvent***	DICHLOROMETHANE-d2		
Spectrum Offset (Hz)	7546.7783	***Sweep Width (Hz)***	59523.58	***Temperature (degree C)***	29.987		

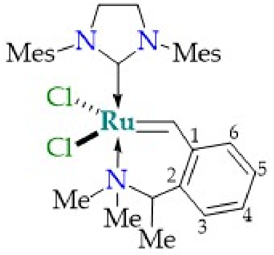

4a

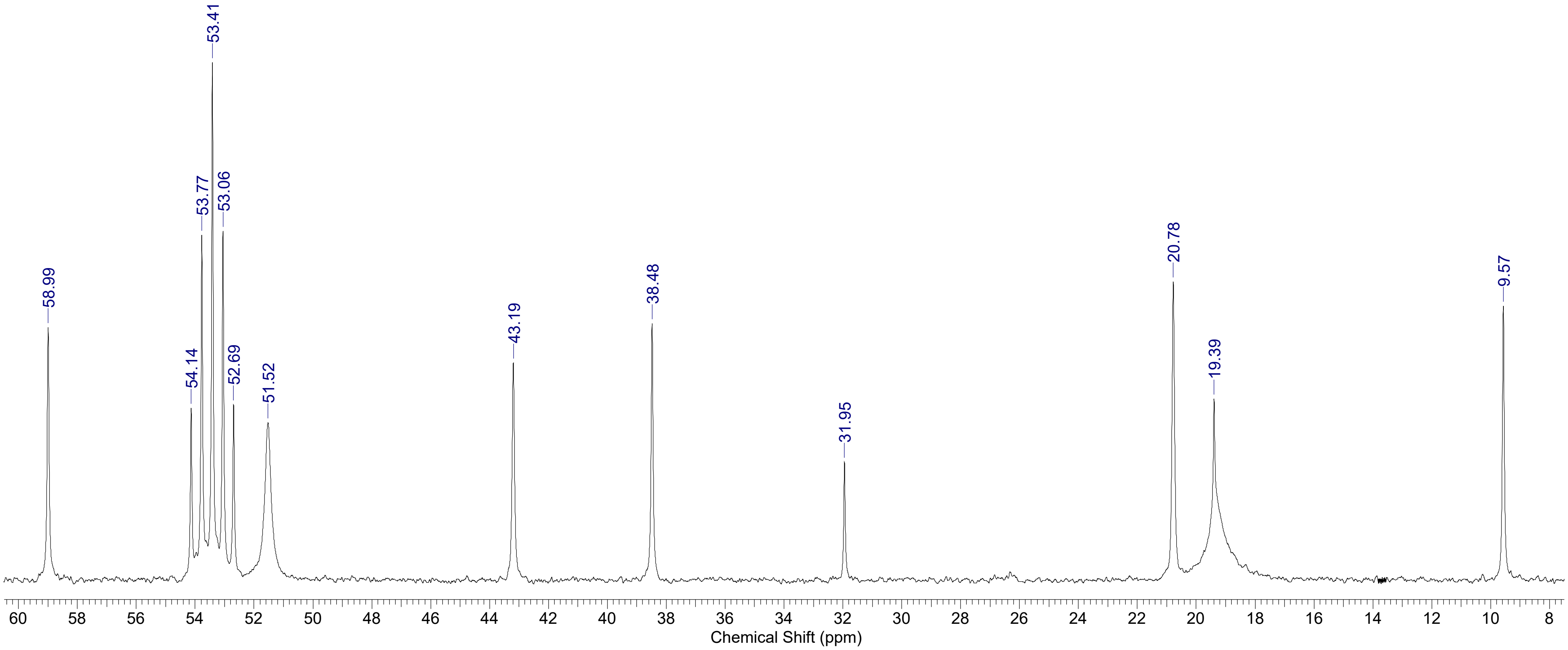

DEPT-135 spectrum of compound **4a**

Formula	$C_{32}H_{41}Cl_2N_3Ru$	***FW***	639.6629

Acquisition Time (sec)	1.1010	***Comment***	FZ_Grubbs_7-K8-dept135 [hp-dec]	***Date***	30 Apr 2019 10:40:00
Date Stamp	30 Apr 2019 10:40:00	***File Name***			
Frequency (MHz)	75.47	***Nucleus***	13C	***Number of Transients***	632
Origin	spect				
Original Points Count	65536	***Owner***	nmr	***Points Count***	262144
Pulse Sequence	dept135				
Receiver Gain	202.48	***SW(cyclical) (Hz)***	59523.81	***Solvent***	DICHLOROMETHANE-d2
Spectrum Offset (Hz)	7546.7783	***Sweep Width (Hz)***	59523.58	***Temperature (degree C)***	29.976

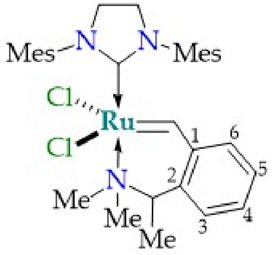

4a

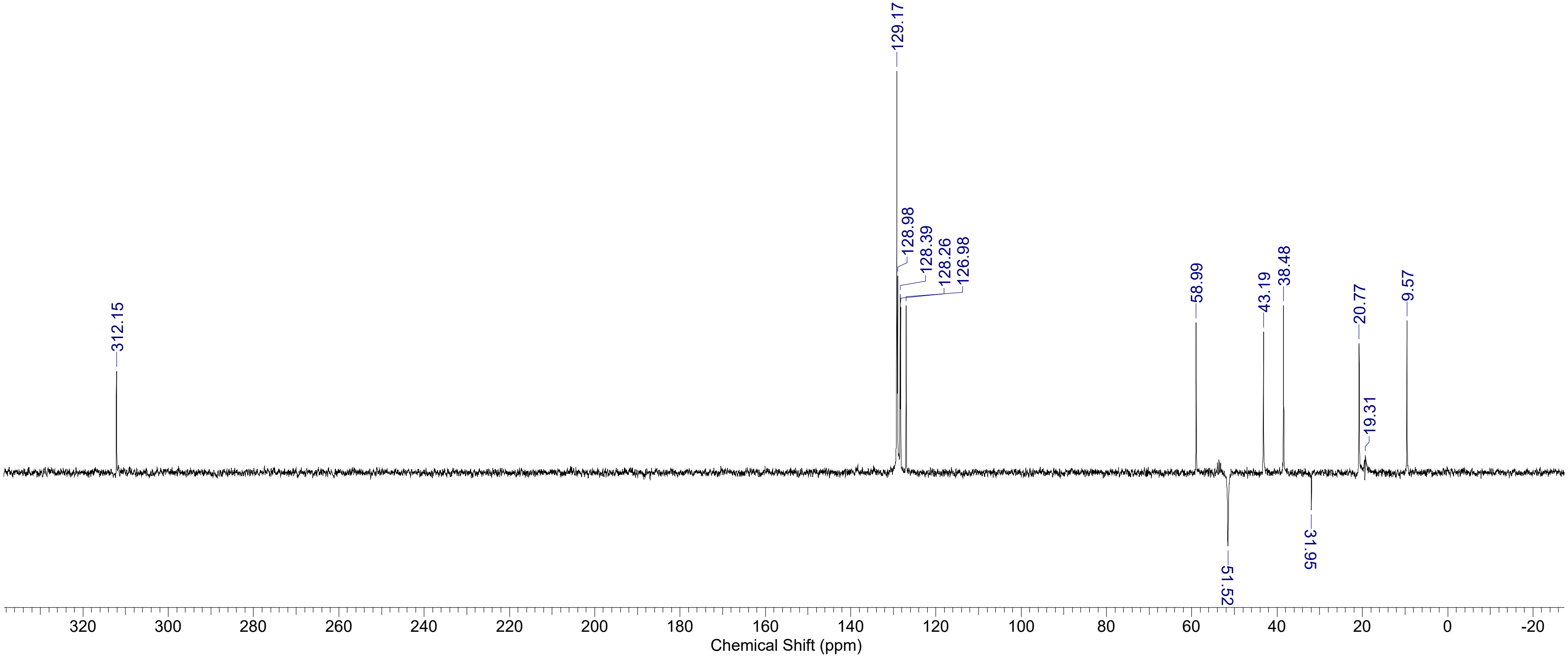

DEPT-135 spectrum of compound **4a**

Formula	$C_{32}H_{41}Cl_2N_3Ru$	***FW***	639.6629				
Acquisition Time (sec)	1.1010	***Comment***	FZ_Grubbs_7-K8-dept135 [hp-dec]			***Date***	30 Apr 2019 10:40:00
Date Stamp	30 Apr 2019 10:40:00			***File Name***			
Frequency (MHz)	75.47	***Nucleus***	13C	***Number of Transients***	632	***Origin***	spect
Original Points Count	65536	***Owner***	nmr	***Points Count***	262144	***Pulse Sequence***	dept135
Receiver Gain	202.48	***SW(cyclical) (Hz)***	59523.81	***Solvent***	DICHLOROMETHANE-d2		
Spectrum Offset (Hz)	7546.7783	***Sweep Width (Hz)***	59523.58	***Temperature (degree C)***	29.976		

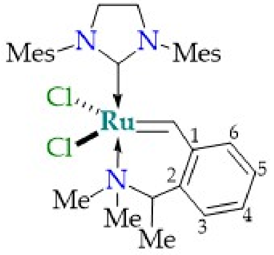

4a

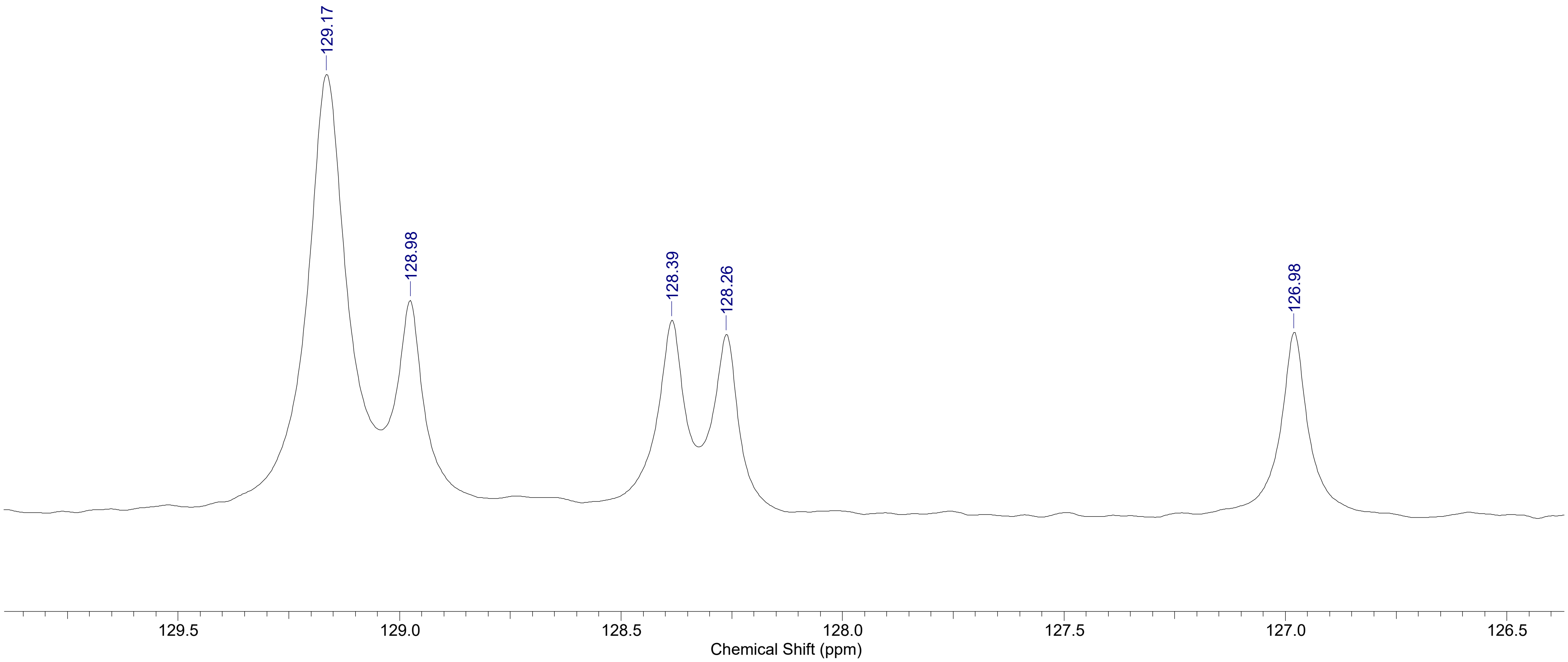

DEPT-135 spectrum of compound **4a**

Formula	$C_{32}H_{41}Cl_2N_3Ru$	FW	639.6629

Acquisition Time (sec)	1.1010	Comment	FZ_Grubbs_7-K8-dept135 [hp-dec]	Date	30 Apr 2019 10:40:00
Date Stamp	30 Apr 2019 10:40:00		File Name		
Frequency (MHz)	75.47	Nucleus	13C	Number of Transients	632	Origin	spect
Original Points Count	65536	Owner	nmr	Points Count	262144	Pulse Sequence	dept135
Receiver Gain	202.48	SW(cyclical) (Hz)	59523.81	Solvent	DICHLOROMETHANE-d2		
Spectrum Offset (Hz)	7546.7783	Sweep Width (Hz)	59523.58	Temperature (degree C)	29.976		

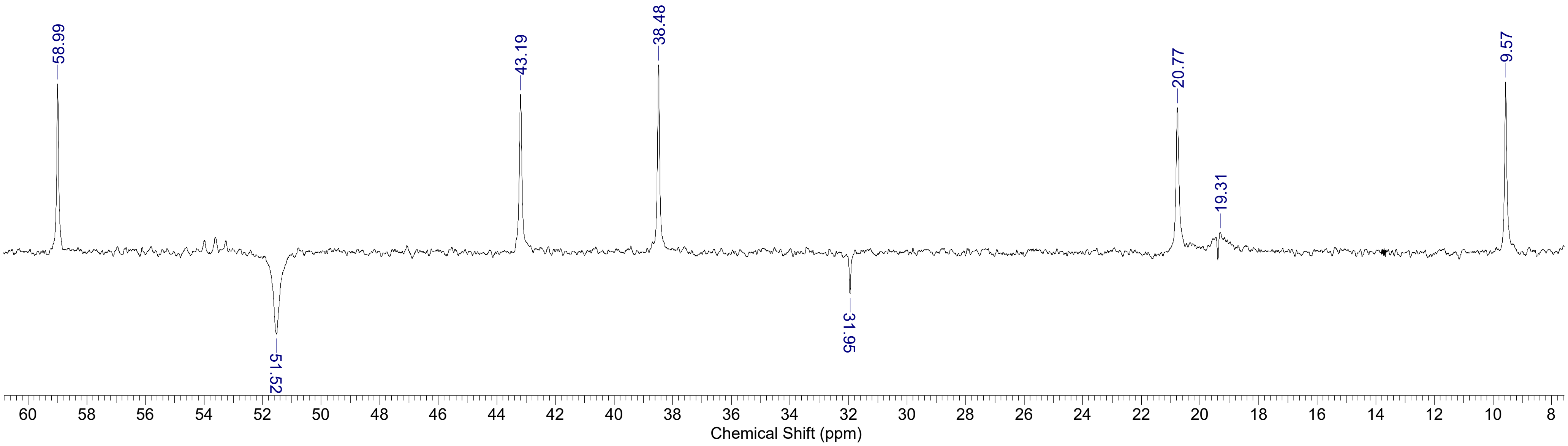

COSY spectrum of compound **4a**

Acquisition Time (sec)	(0.3408, 0.0426)	***Comment***	5 mm PABBO BB-1H/D Z-GRD Z104275/0345			***Date***	30 Apr 2019 17:10:26
File Name	C:\Users\Fedor\Desktop\Новые катализаторы от Ромы\FZ_Grubbs_7-K8\11\pdata\1\2rr					***Frequency (MHz)***	(300.13, 300.13)
Nucleus	(1H, 1H)	***Number of Transients***	1	***Origin***	spect	***Original Points Count***	(3072, 384)
Owner	nmr	***Points Count***	(8192, 1024)	***Pulse Sequence***	cosygpqf	***Solvent***	CD2Cl2
Sweep Width (Hz)	(9014.42, 9009.01)	***Temperature (degree C)***	30.007	***Title***	FZ_Grubbs_7-K8-COSY		

Formula	$C_{32}H_{41}Cl_2N_3Ru$	***FW***	639.6629

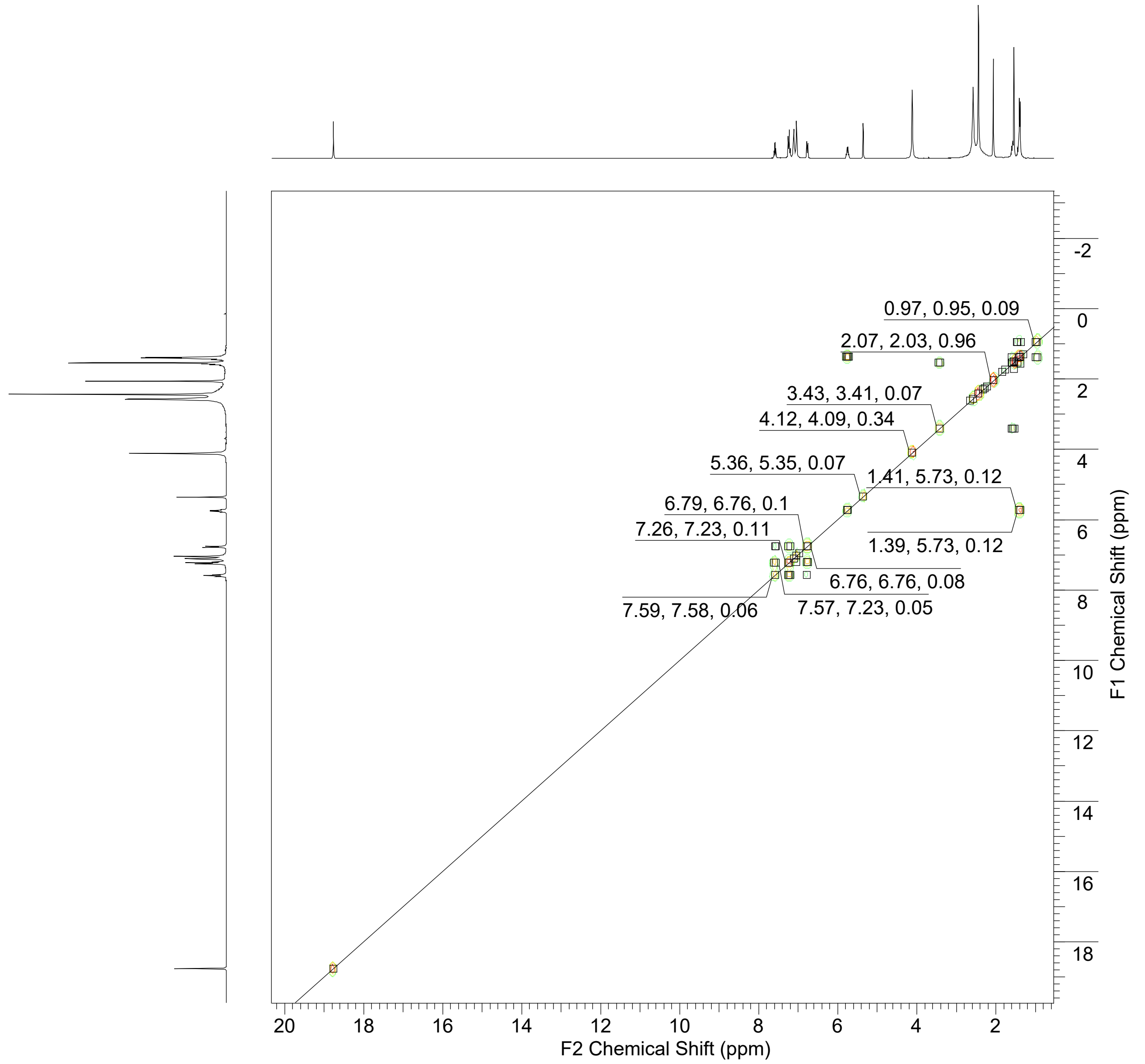

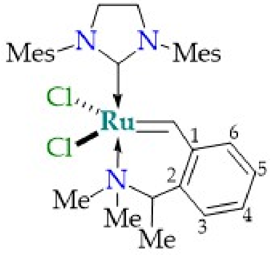

4a

COSY spectrum of compound **4a**

Acquisition Time (sec)	(0.3408, 0.0426)	***Comment***	5 mm PABBO BB-1H/D Z-GRD Z104275/0345			***Date***	30 Apr 2019 17:10:26
File Name	C:\Users\Fedor\Desktop\Новые катализаторы от Ромы\FZ_Grubbs_7-K8\11\pdata\1\2rr					***Frequency (MHz)***	(300.13, 300.13)
Nucleus	(1H, 1H)	***Number of Transients***	1	***Origin***	spect	***Original Points Count***	(3072, 384)
Owner	nmr	***Points Count***	(8192, 1024)	***Pulse Sequence***	cosygpqf	***Solvent***	CD2Cl2
Sweep Width (Hz)	(9014.42, 9009.01)	***Temperature (degree C)***	30.007	***Title***	FZ_Grubbs_7-K8-COSY		

Formula	$C_{32}H_{41}Cl_2N_3Ru$	***FW***	639.6629

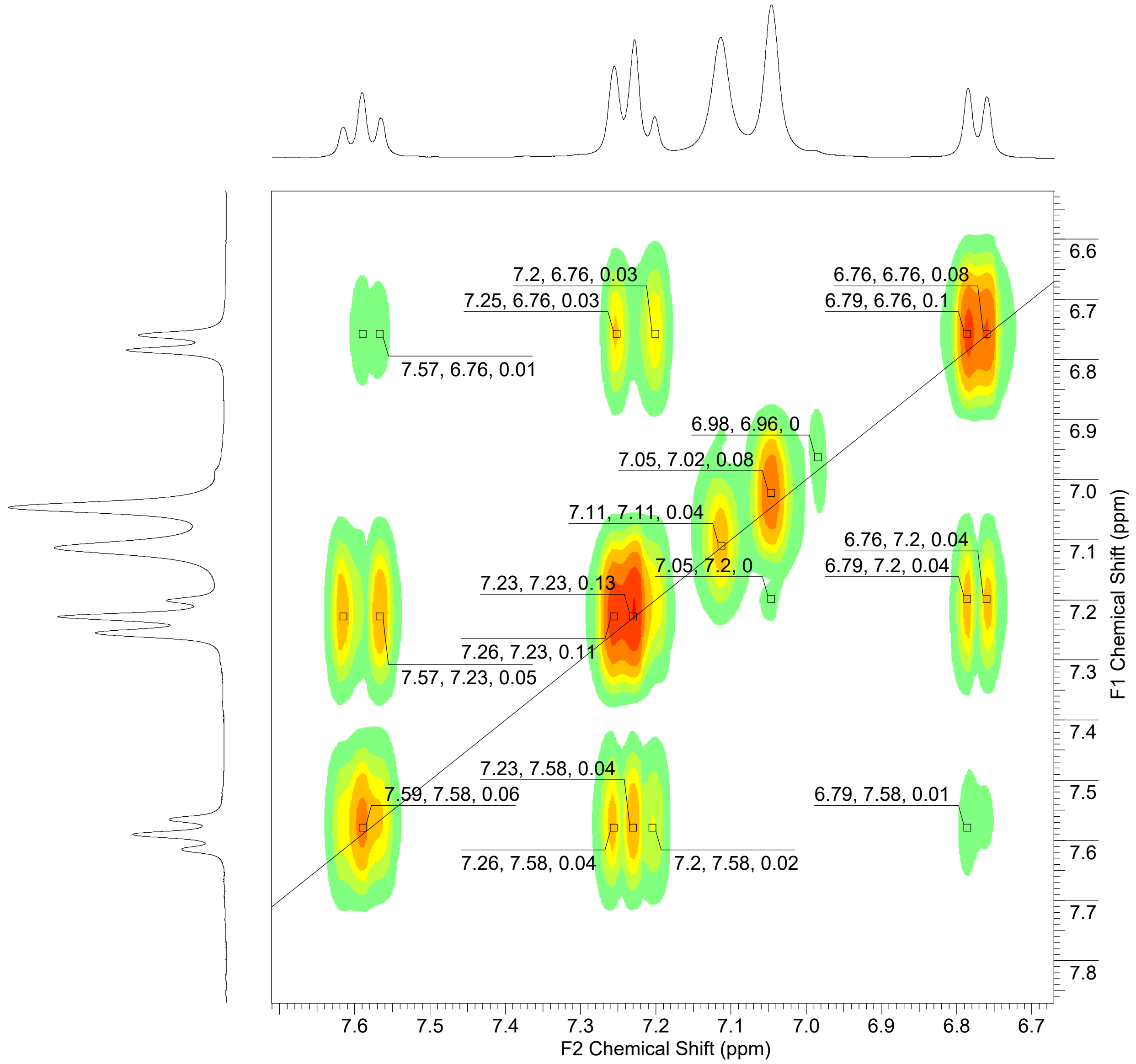

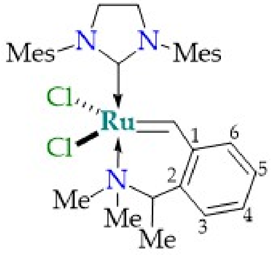

4a

COSY spectrum of compound **4a**

Acquisition Time (sec)	(0.3408, 0.0426)	***Comment***	5 mm PABBO BB-1H/D Z-GRD Z104275/0345			***Date***	30 Apr 2019 17:10:26
File Name	C:\Users\Fedor\Desktop\Новые катализаторы от Ромы\FZ_Grubbs_7-K8\11\pdata\1\2rr					***Frequency (MHz)***	(300.13, 300.13)
Nucleus	(1H, 1H)	***Number of Transients***	1	***Origin***	spect	***Original Points Count***	(3072, 384)
Owner	nmr	***Points Count***	(8192, 1024)	***Pulse Sequence***	cosygpqf	***Solvent***	CD2Cl2
Sweep Width (Hz)	(9014.42, 9009.01)	***Temperature (degree C)***	30.007	***Title***	FZ_Grubbs_7-K8-COSY		

Formula	$C_{32}H_{41}Cl_2N_3Ru$	***FW***	639.6629

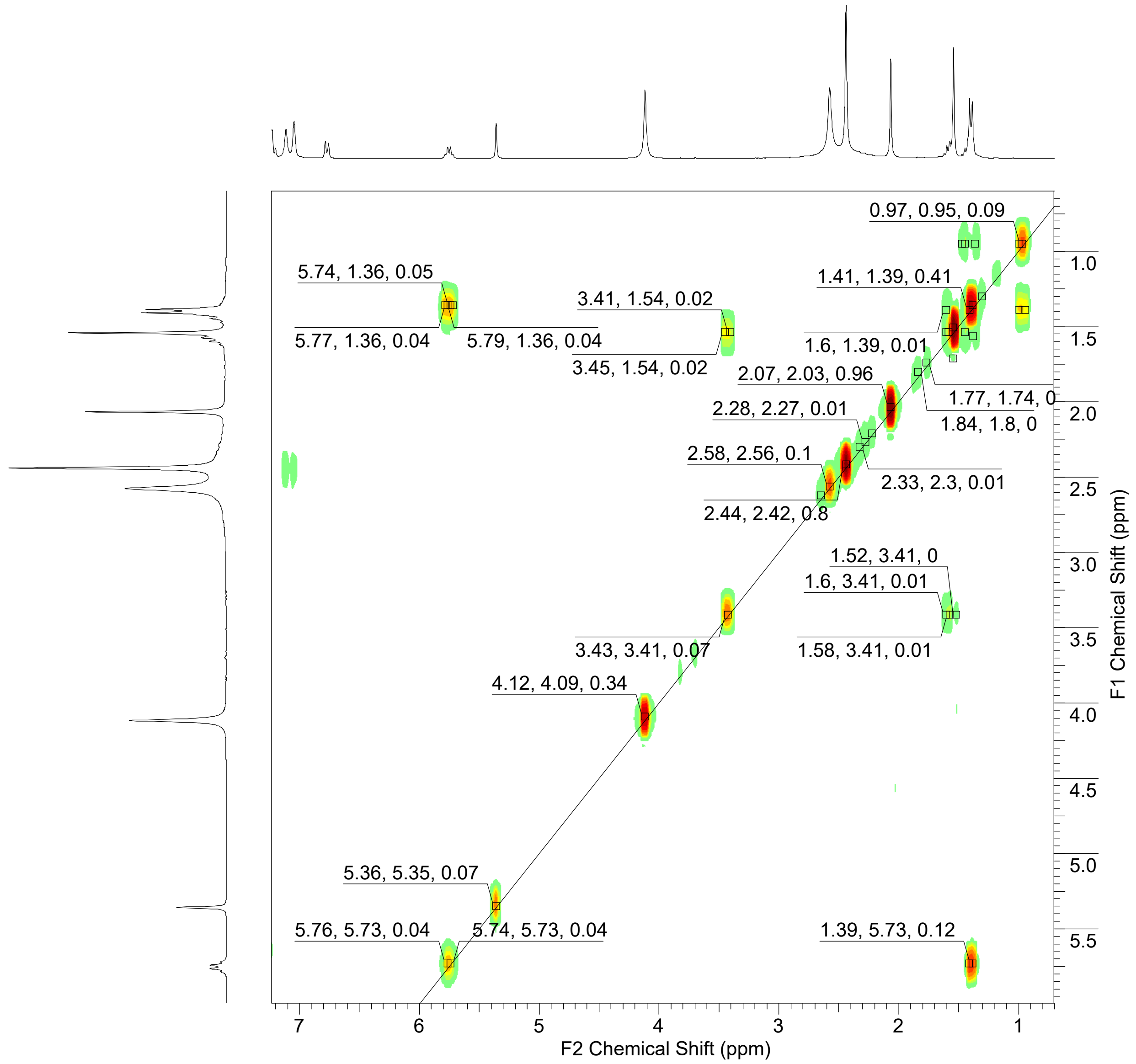

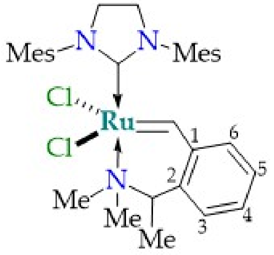

4a

HSQC spectrum of compound **4a**

Acquisition Time (sec)	(0.3408, 0.0089)	***Comment***	5 mm PABBO BB-1H/D Z-GRD Z104275/0345			***Date***	30 Apr 2019 17:07:58
File Name	C:\Users\Fedor\Desktop\Новые катализаторы от Ромы\FZ_Grubbs_7-K8\113\pdata\1\2rr					***Frequency (MHz)***	(300.13, 75.48)
Nucleus	(1H, 13C)	***Number of Transients***	2	***Origin***	spect	***Original Points Count***	(3072, 304)
Owner	nmr	***Points Count***	(8192, 1024)	***Pulse Sequence***	hsqcetgp	***Solvent***	CD2Cl2
Sweep Width (Hz)	(9014.42, 34013.61)	***Temperature (degree C)***	30.070	***Title***	FZ_Grubbs_7-K8-HSQC [hp-dec]		

Formula	$C_{32}H_{41}Cl_2N_3Ru$	***FW***	639.6629

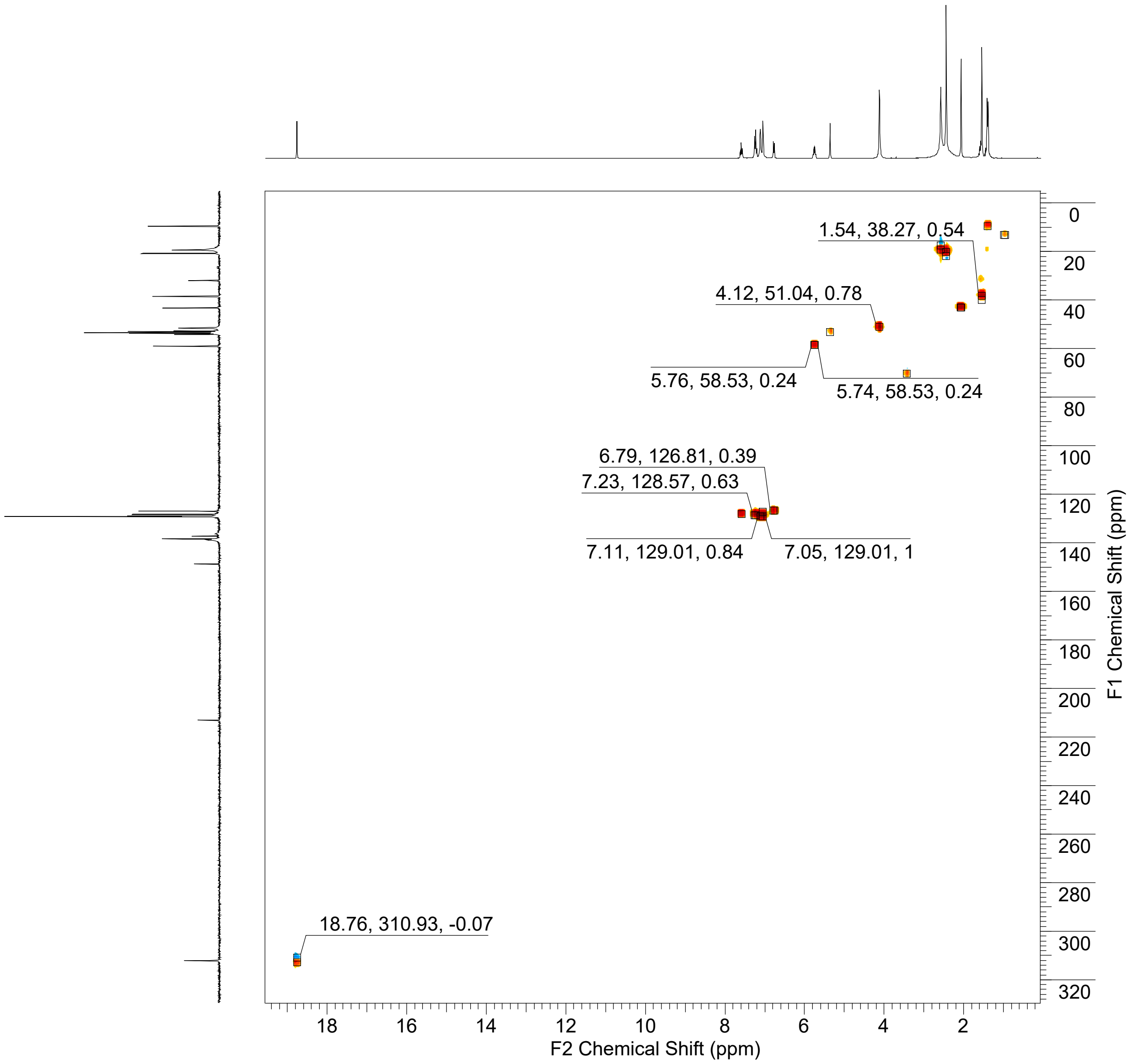

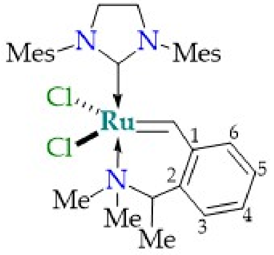

4a

HSQC spectrum of compound **4a**

Acquisition Time (sec)	(0.3408, 0.0089)	Comment	5 mm PABBO BB-1H/D Z-GRD Z104275/0345			Date	30 Apr 2019 17:07:58
File Name	C:\Users\Fedor\Desktop\Новые катализаторы от Ромы\FZ_Grubbs_7-K8\113\pdata\1\2rr					Frequency (MHz)	(300.13, 75.48)
Nucleus	(1H, 13C)	Number of Transients	2	Origin	spect	Original Points Count	(3072, 304)
Owner	nmr	Points Count	(8192, 1024)	Pulse Sequence	hsqcetgp	Solvent	CD2Cl2
Sweep Width (Hz)	(9014.42, 34013.61)	Temperature (degree C)	30.070	Title	FZ_Grubbs_7-K8-HSQC [hp-dec]		

Formula	$C_{32}H_{41}Cl_2N_3Ru$	FW	639.6629

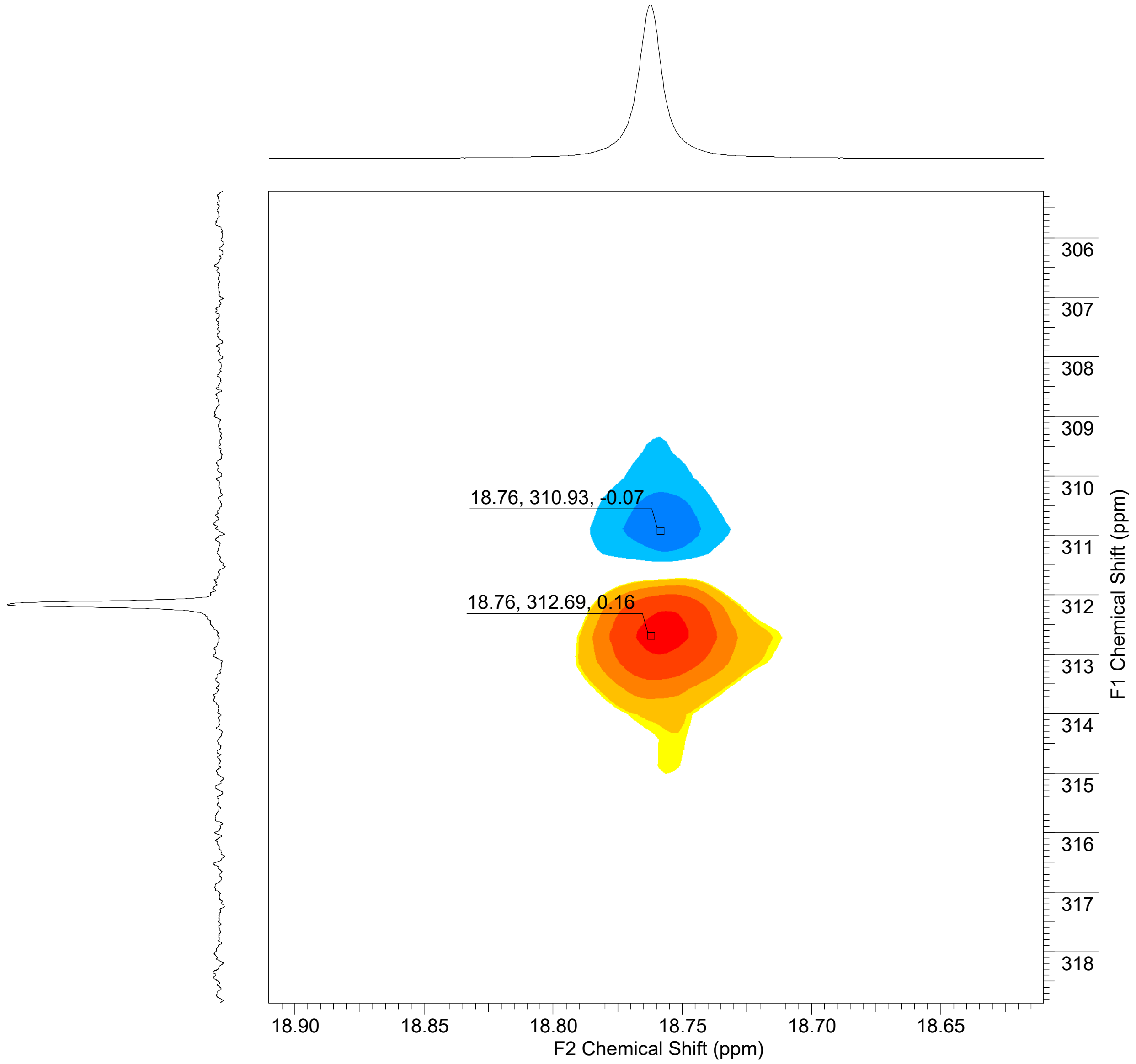

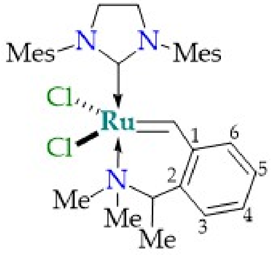

4a

HSQC spectrum of compound **4a**

Acquisition Time (sec)	(0.3408, 0.0089)	***Comment***	5 mm PABBO BB-1H/D Z-GRD Z104275/0345			***Date***	30 Apr 2019 17:07:58
File Name	C:\Users\Fedor\Desktop\Новые катализаторы от Ромы\FZ_Grubbs_7-K8\113\pdata\1\2rr					***Frequency (MHz)***	(300.13, 75.48)
Nucleus	(1H, 13C)	***Number of Transients***	2	***Origin***	spect	***Original Points Count***	(3072, 304)
Owner	nmr	***Points Count***	(8192, 1024)	***Pulse Sequence***	hsqcetgp	***Solvent***	CD2Cl2
Sweep Width (Hz)	(9014.42, 34013.61)	***Temperature (degree C)***	30.070	***Title***	FZ_Grubbs_7-K8-HSQC [hp-dec]		

Formula $C_{32}H_{41}Cl_2N_3Ru$	***FW*** 639.6629

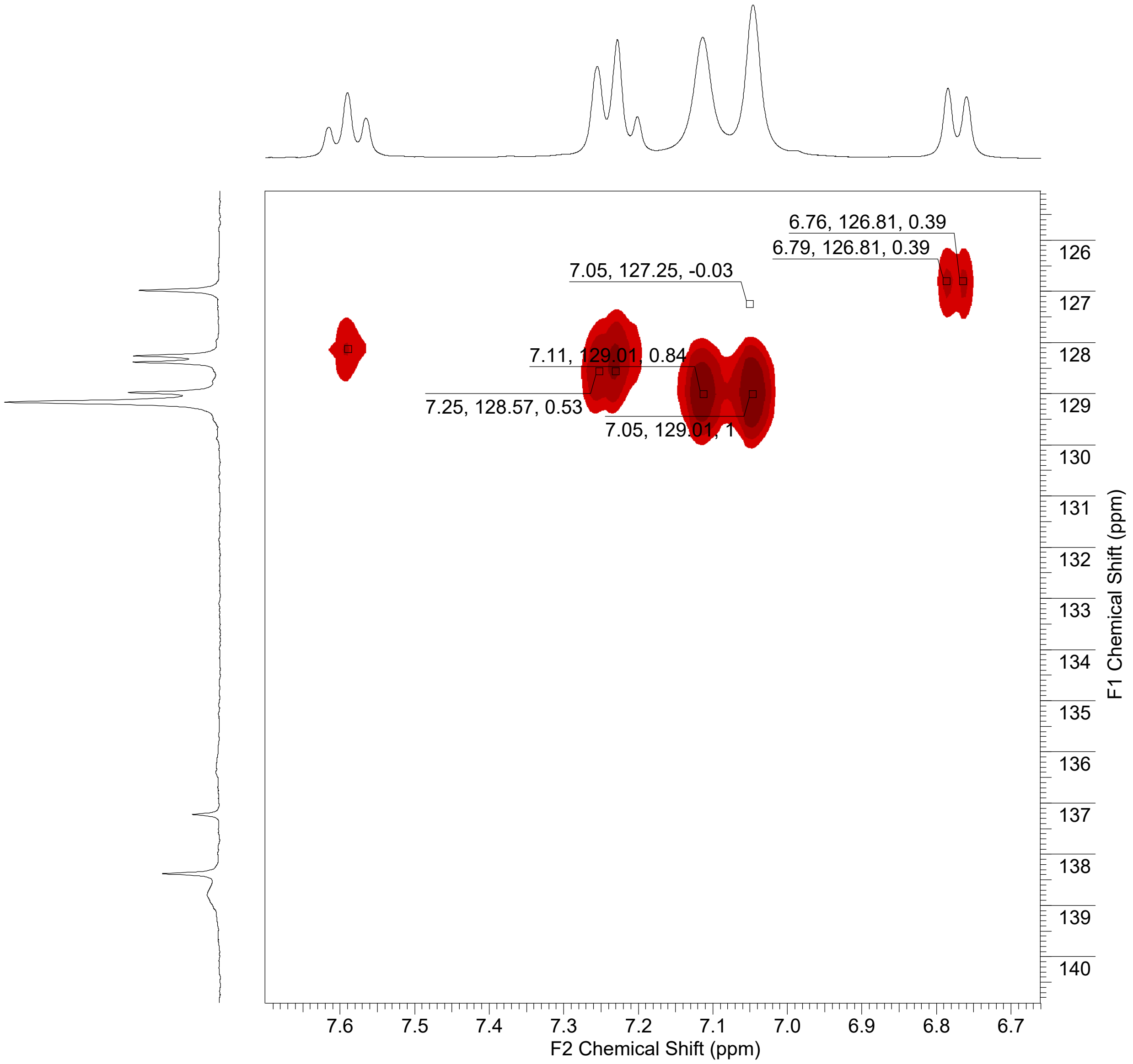

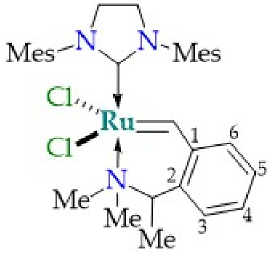

4a

HSQC spectrum of compound **4a**

Acquisition Time (sec)	(0.3408, 0.0089)	***Comment***	5 mm PABBO BB-1H/D Z-GRD Z104275/0345			***Date***	30 Apr 2019 17:07:58
File Name	C:\Users\Fedor\Desktop\Новые катализаторы от Ромы\FZ_Grubbs_7-K8\113\pdata\1\2rr					***Frequency (MHz)***	(300.13, 75.48)
Nucleus	(1H, 13C)	***Number of Transients***	2	***Origin***	spect	***Original Points Count***	(3072, 304)
Owner	nmr	***Points Count***	(8192, 1024)	***Pulse Sequence***	hsqcetgp	***Solvent***	CD2Cl2
Sweep Width (Hz)	(9014.42, 34013.61)	***Temperature (degree C)***	30.070	***Title***	FZ_Grubbs_7-K8-HSQC [hp-dec]		

Formula	$C_{32}H_{41}Cl_2N_3Ru$	***FW***	639.6629

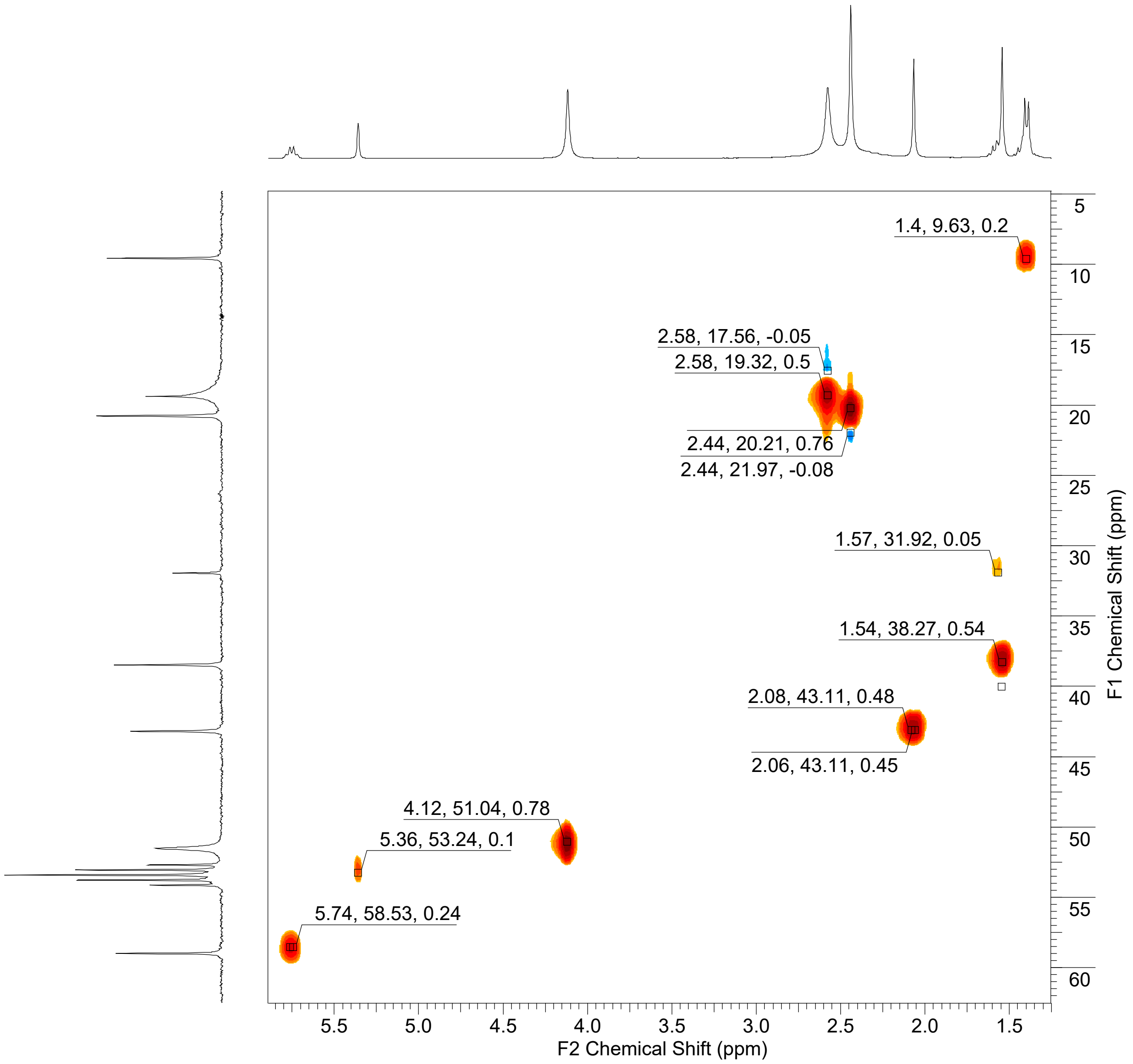

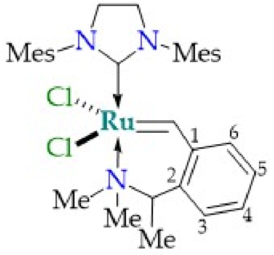

4a

HMBC spectrum of compound **4a**

Acquisition Time (sec)	(0.3408, 0.0089)	***Comment***	5 mm PABBO BB-1H/D Z-GRD Z104275/0345	***Date***	30 Apr 2019 17:09:48
File Name	C:\Users\Fedor\Desktop\Новые катализаторы от Ромы\FZ_Grubbs_7-K8\213\pdata\1\2rr			***Frequency (MHz)***	(300.13, 75.48)
Nucleus	(1H, 13C)	***Number of Transients***	4	***Origin***	spect
Original Points Count	(3072, 304)				
Owner	nmr	***Points Count***	(8192, 1024)	***Pulse Sequence***	hmbcgplpndqf
Solvent	CD2Cl2				
Sweep Width (Hz)	(9014.42, 34013.61)	***Temperature (degree C)***	29.520	***Title***	FZ_Grubbs_7-K8-HMBC

Formula $C_{32}H_{41}Cl_2N_3Ru$	***FW*** 639.6629

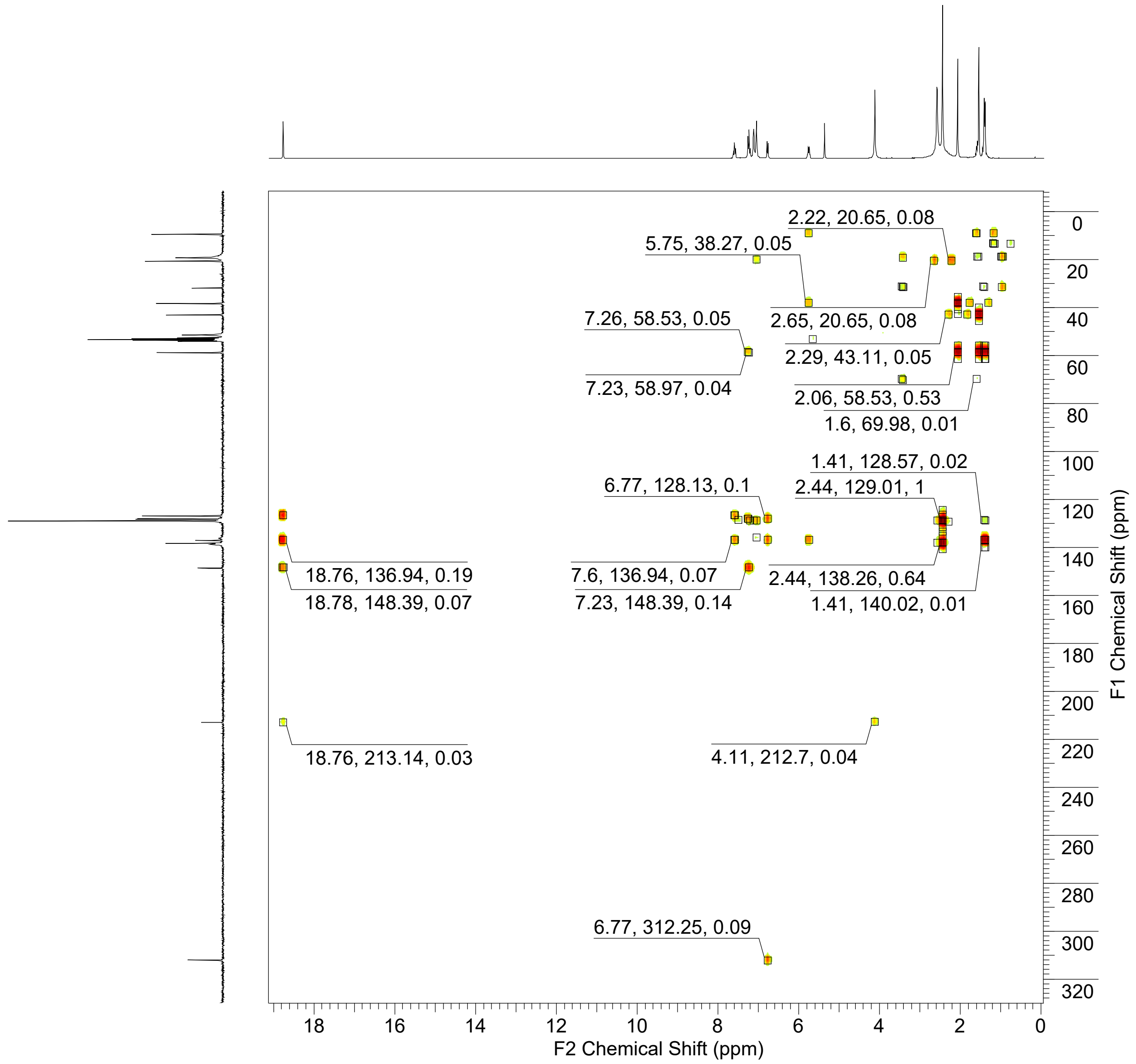

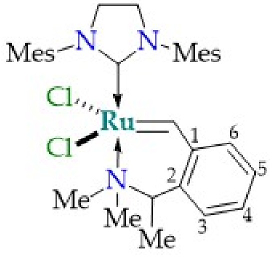

4a

HMBC spectrum of compound **4a**

Acquisition Time (sec)	(0.3408, 0.0089)	***Comment***	5 mm PABBO BB-1H/D Z-GRD Z104275/0345			***Date***	30 Apr 2019 17:09:48
File Name	C:\Users\Fedor\Desktop\Новые катализаторы от Ромы\FZ_Grubbs_7-K8\213\pdata\1\2rr					***Frequency (MHz)***	(300.13, 75.48)
Nucleus	(1H, 13C)	***Number of Transients***	4	***Origin***	spect	***Original Points Count***	(3072, 304)
Owner	nmr	***Points Count***	(8192, 1024)	***Pulse Sequence***	hmbcgplpndqf	***Solvent***	CD2Cl2
Sweep Width (Hz)	(9014.42, 34013.61)	***Temperature (degree C)***	29.520	***Title***	FZ_Grubbs_7-K8-HMBC		

Formula	$C_{32}H_{41}Cl_2N_3Ru$	***FW***	639.6629

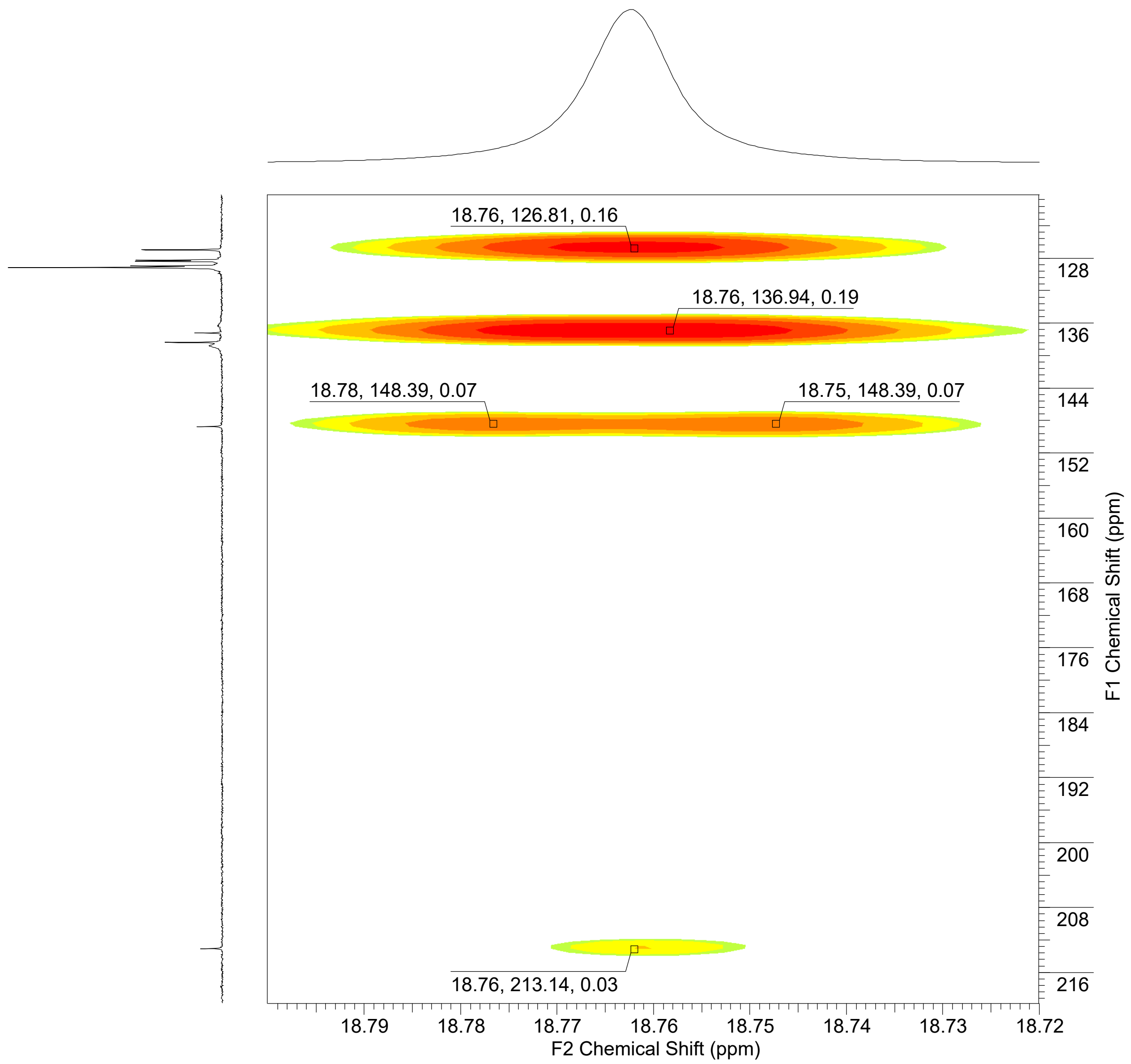

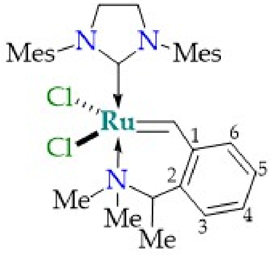

4a

HMBC spectrum of compound **4a**

Acquisition Time (sec)	(0.3408, 0.0089)	Comment	5 mm PABBO BB-1H/D Z-GRD Z104275/0345			Date	30 Apr 2019 17:09:48
File Name	C:\Users\Fedor\Desktop\Новые катализаторы от Ромы\FZ_Grubbs_7-K8\213\pdata\1\2rr					Frequency (MHz)	(300.13, 75.48)
Nucleus	(1H, 13C)	Number of Transients	4	Origin	spect	Original Points Count	(3072, 304)
Owner	nmr	Points Count	(8192, 1024)	Pulse Sequence	hmbcgplpndqf	Solvent	CD2Cl2
Sweep Width (Hz)	(9014.42, 34013.61)	Temperature (degree C)	29.520	Title	FZ_Grubbs_7-K8-HMBC		

Formula	$C_{32}H_{41}Cl_2N_3Ru$	FW	639.6629

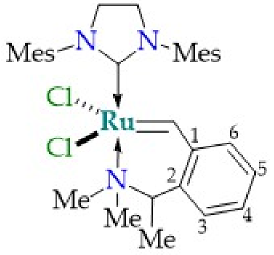

4a

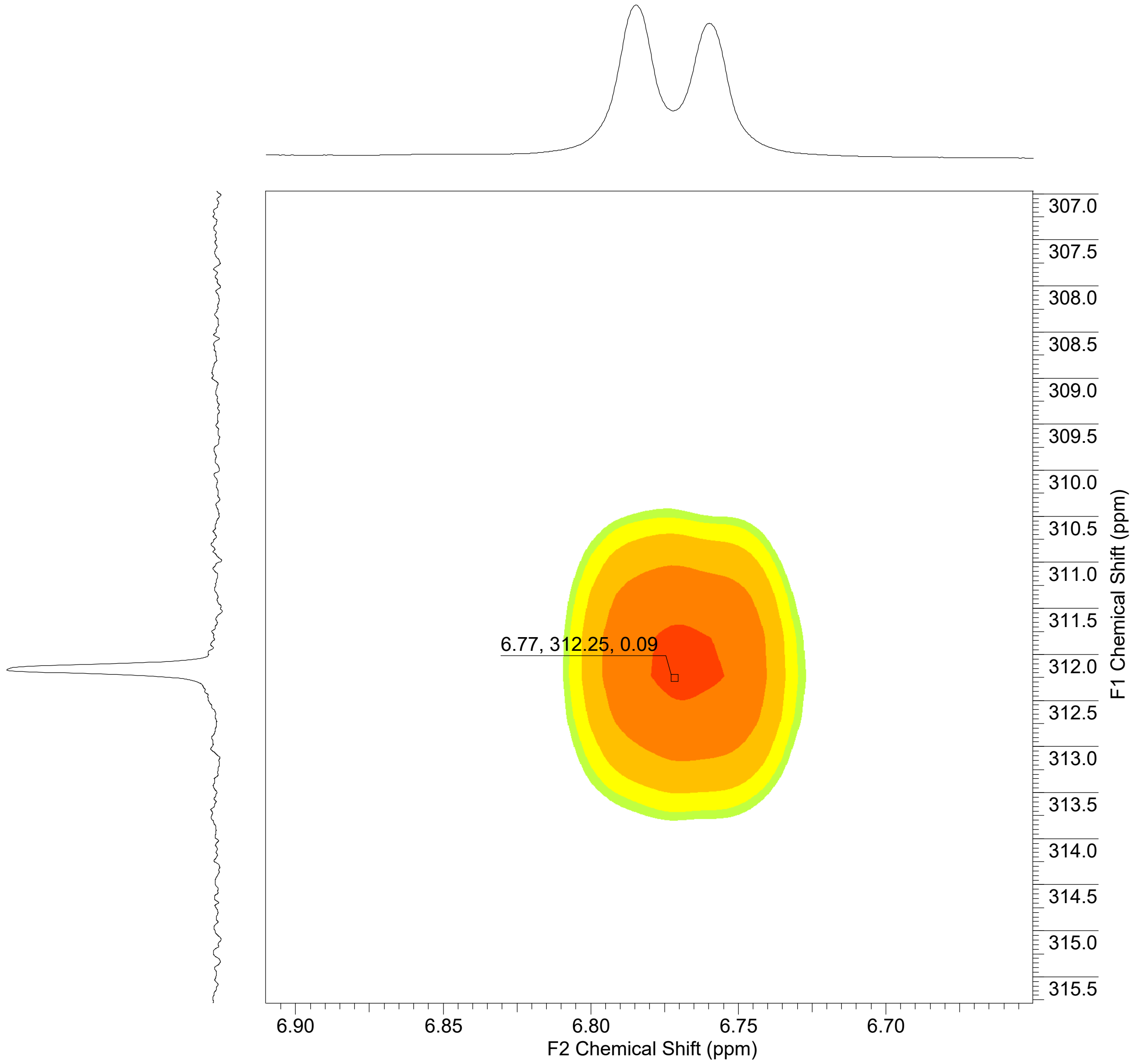

HMBC spectrum of compound **4a**

Acquisition Time (sec)	(0.3408, 0.0089)	***Comment***	5 mm PABBO BB-1H/D Z-GRD Z104275/0345			***Date***	30 Apr 2019 17:09:48
File Name	C:\Users\Fedor\Desktop\Новые катализаторы от Ромы\FZ_Grubbs_7-K8\213\pdata\1\2rr					***Frequency (MHz)***	(300.13, 75.48)
Nucleus	(1H, 13C)	***Number of Transients***	4	***Origin***	spect	***Original Points Count***	(3072, 304)
Owner	nmr	***Points Count***	(8192, 1024)	***Pulse Sequence***	hmbcgplpndqf	***Solvent***	CD2Cl2
Sweep Width (Hz)	(9014.42, 34013.61)	***Temperature (degree C)***	29.520	***Title***	FZ_Grubbs_7-K8-HMBC		

Formula	$C_{32}H_{41}Cl_2N_3Ru$	***FW***	639.6629

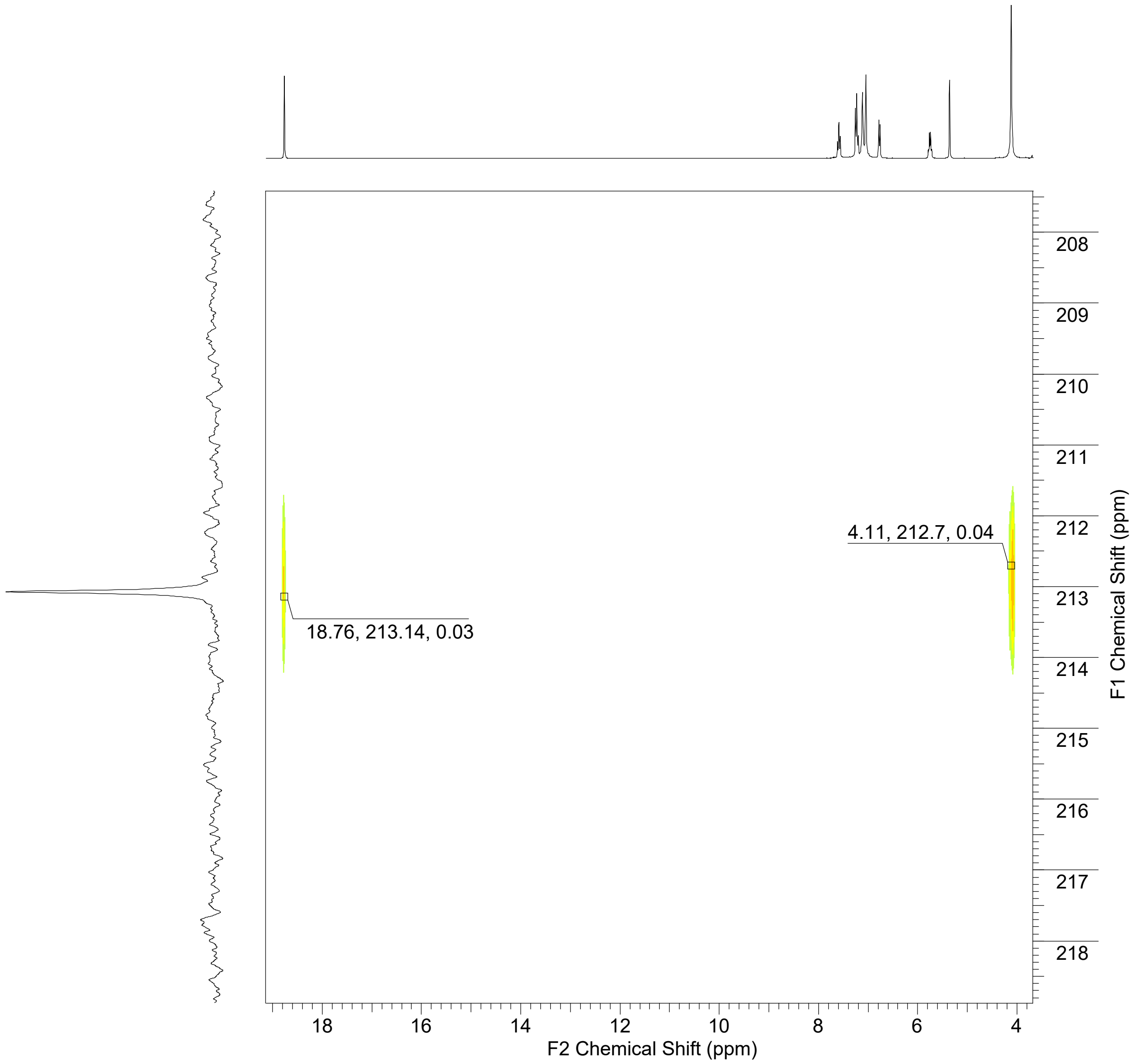

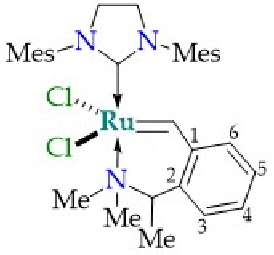

4a

HMBC spectrum of compound **4a**

Acquisition Time (sec)	(0.3408, 0.0089)	***Comment***	5 mm PABBO BB-1H/D Z-GRD Z104275/0345			***Date***	30 Apr 2019 17:09:48
File Name	C:\Users\Fedor\Desktop\Новые катализаторы от Ромы\FZ_Grubbs_7-K8\213\pdata\1\2rr					***Frequency (MHz)***	(300.13, 75.48)
Nucleus	(1H, 13C)	***Number of Transients***	4	***Origin***	spect	***Original Points Count***	(3072, 304)
Owner	nmr	***Points Count***	(8192, 1024)	***Pulse Sequence***	hmbcgplpndqf	***Solvent***	CD2Cl2
Sweep Width (Hz)	(9014.42, 34013.61)	***Temperature (degree C)***	29.520	***Title***	FZ_Grubbs_7-K8-HMBC		

Formula $C_{32}H_{41}Cl_2N_3Ru$	***FW*** 639.6629

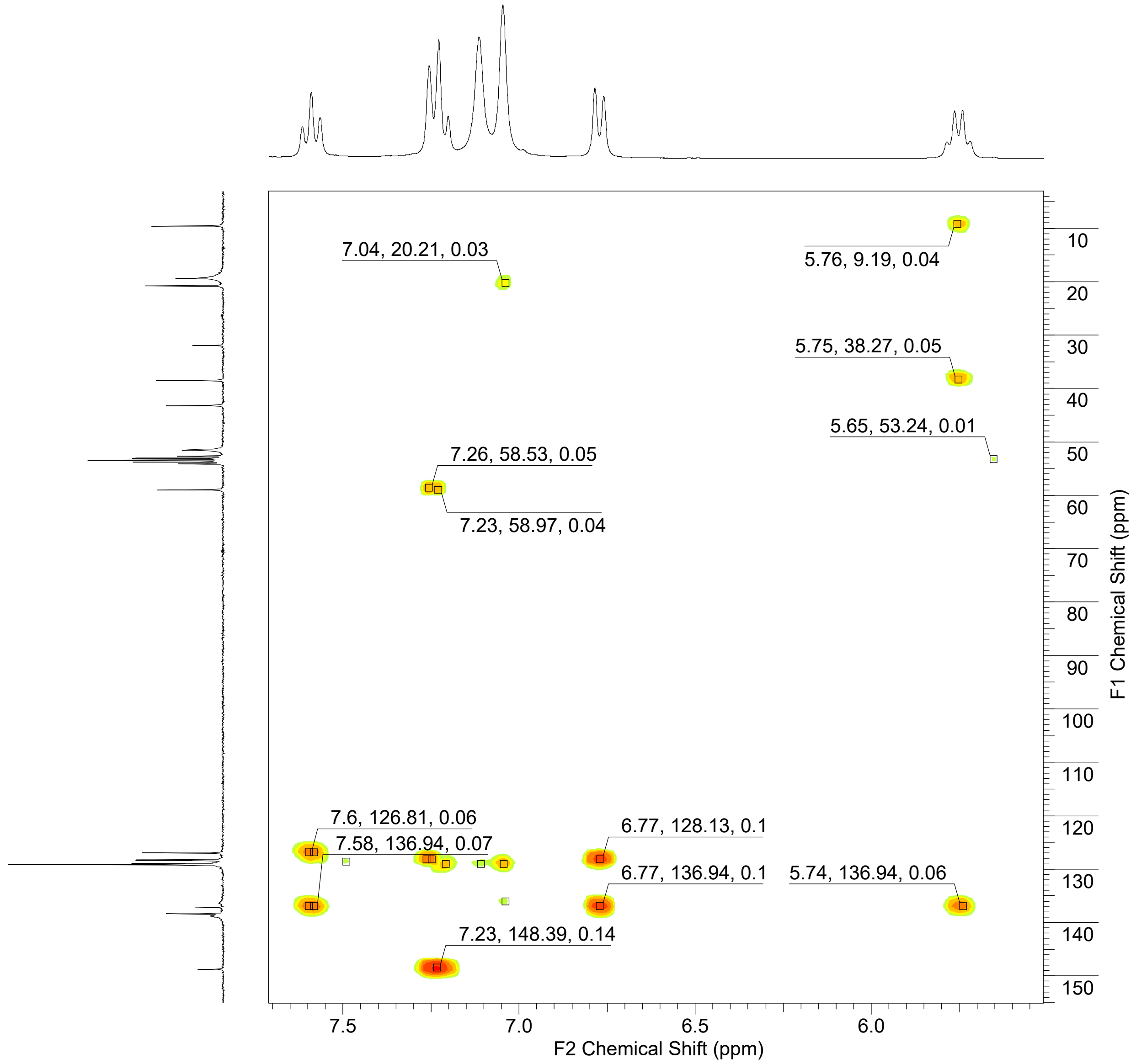

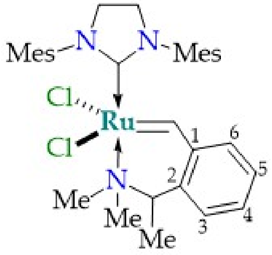

4a

HMBC spectrum of compound **4a**

Acquisition Time (sec)	(0.3408, 0.0089)	***Comment***	5 mm PABBO BB-1H/D Z-GRD Z104275/0345			***Date***	30 Apr 2019 17:09:48
File Name	C:\Users\Fedor\Desktop\Новые катализаторы от Ромы\FZ_Grubbs_7-K8\213\pdata\1\2rr					***Frequency (MHz)***	(300.13, 75.48)
Nucleus	(1H, 13C)	***Number of Transients***	4	***Origin***	spect	***Original Points Count***	(3072, 304)
Owner	nmr	***Points Count***	(8192, 1024)	***Pulse Sequence***	hmbcgplpndqf	***Solvent***	CD2Cl2
Sweep Width (Hz)	(9014.42, 34013.61)	***Temperature (degree C)***	29.520	***Title***	FZ_Grubbs_7-K8-HMBC		

Formula	$C_{32}H_{41}Cl_2N_3Ru$	***FW***	639.6629

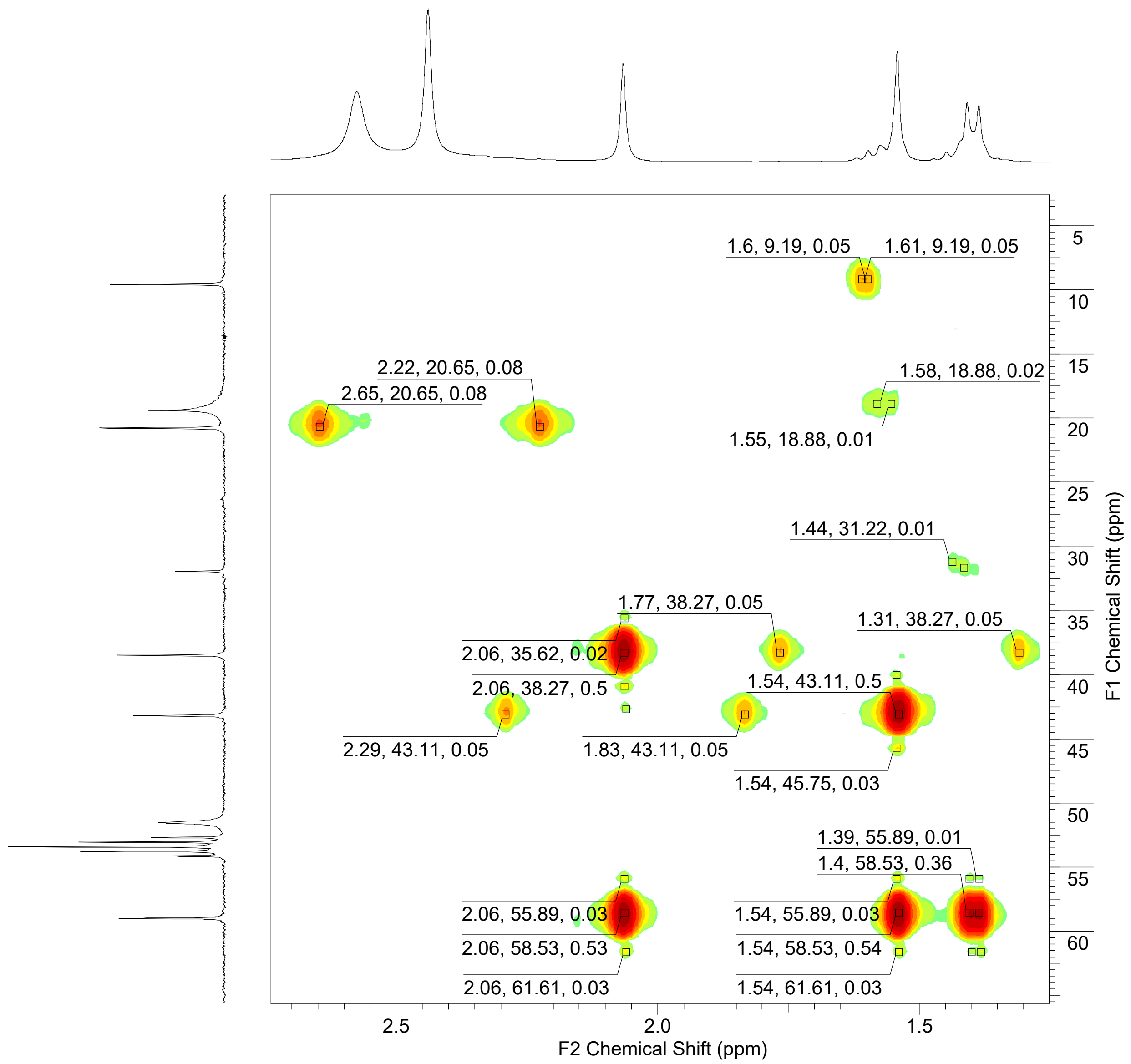

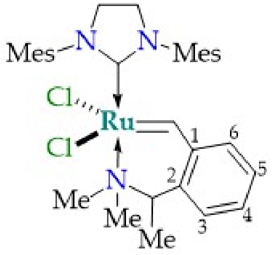

4a

IR spectrum of compound **4a**

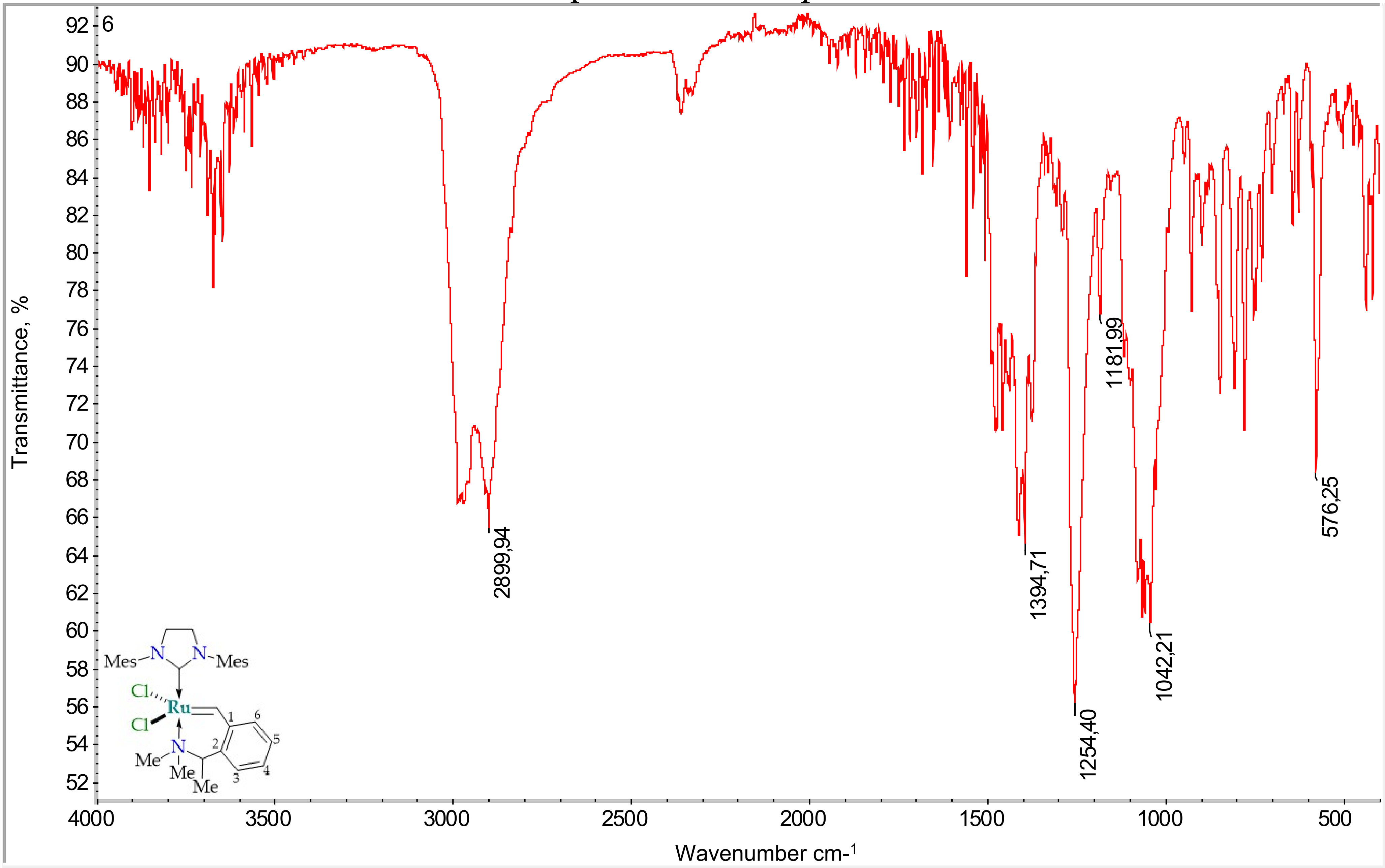

^{1}H spectrum of compound **4b**

Acquisition Time (sec)	1.9818	***Comment***	single_pulse	***Date***	14 Sep 2022 16:08:34		
Date Stamp	14 Sep 2022 15:53:41			***File Name***			
Frequency (MHz)	600.17	***Nucleus***	1H	***Number of Transients***	8	***Origin***	ECA 600
Original Points Count	32768	***Owner***	CKP	***Points Count***	32768	***Pulse Sequence***	single_pulse.ex2
Receiver Gain	44.00	***Solvent***	CHLOROFORM-d			***Spectrum Offset (Hz)***	5413.0830
Sweep Width (Hz)	16534.39						

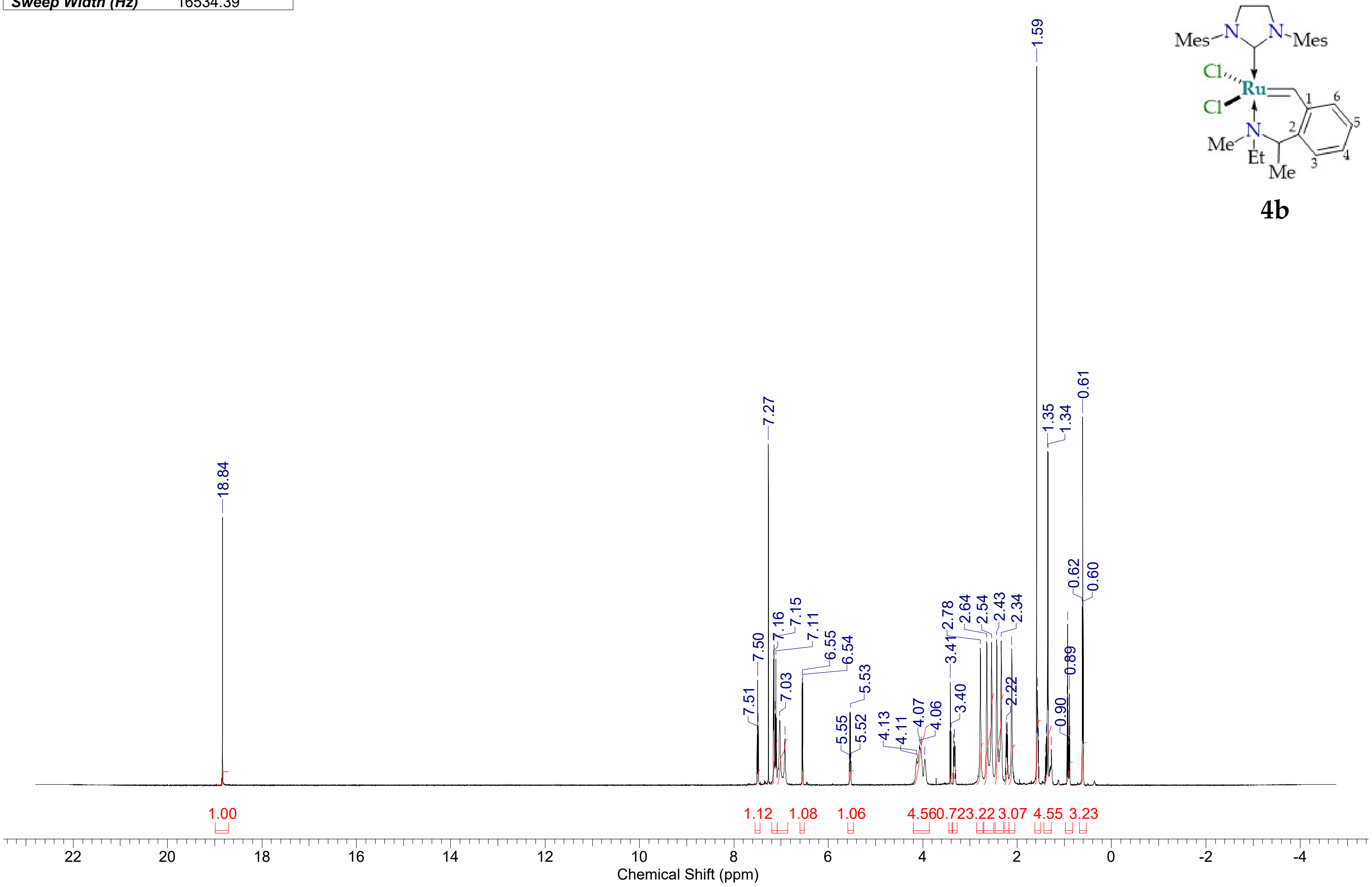

4b

^{1}H spectrum of compound **4b**

Acquisition Time (sec)	1.9818	***Comment***	single_pulse	***Date***	14 Sep 2022 16:08:34		
Date Stamp	14 Sep 2022 15:53:41			***File Name***			
Frequency (MHz)	600.17	***Nucleus***	1H	***Number of Transients***	8	***Origin***	ECA 600
Original Points Count	32768	***Owner***	CKP	***Points Count***	32768	***Pulse Sequence***	single_pulse.ex2
Receiver Gain	44.00	***Solvent***	CHLOROFORM-d			***Spectrum Offset (Hz)***	5413.0830
Sweep Width (Hz)	16534.39						

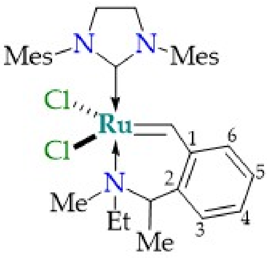

4b

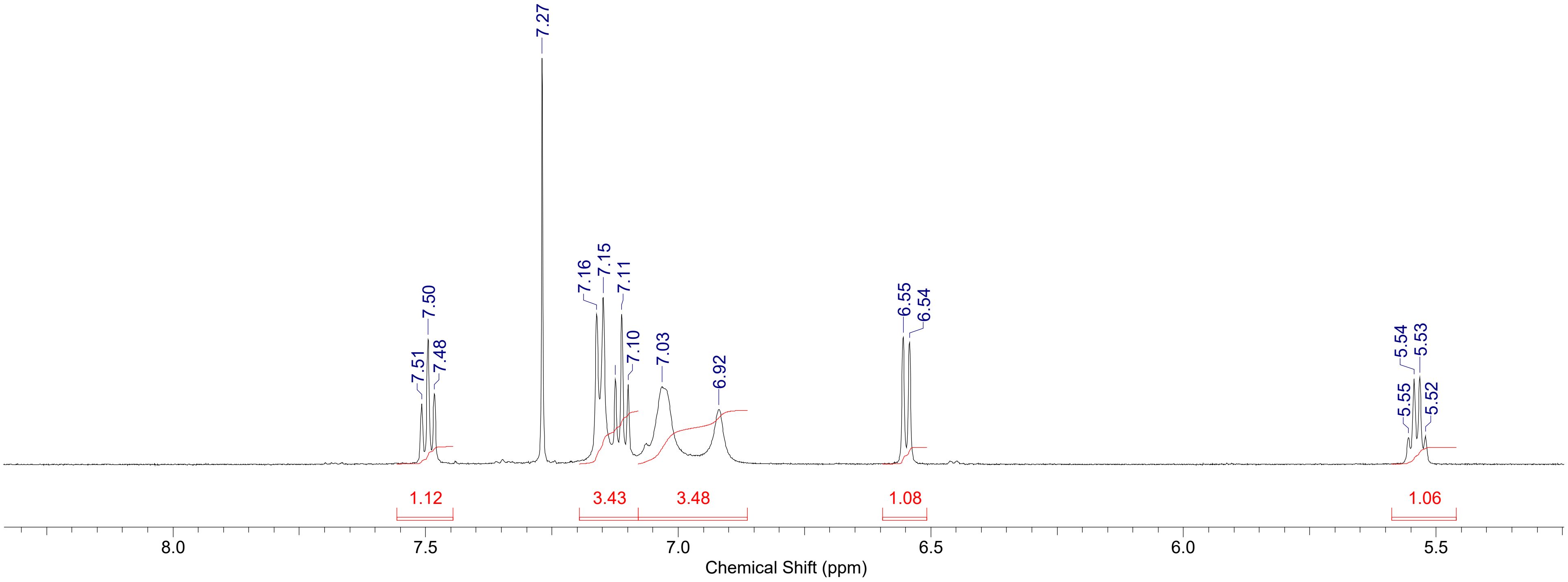

^{1}H spectrum of compound **4b**

Acquisition Time (sec)	1.9818	***Comment***	single_pulse	***Date***	14 Sep 2022 16:08:34		
Date Stamp	14 Sep 2022 15:53:41			***File Name***			
Frequency (MHz)	600.17	***Nucleus***	1H	***Number of Transients***	8	***Origin***	ECA 600
Original Points Count	32768	***Owner***	CKP	***Points Count***	32768	***Pulse Sequence***	single_pulse.ex2
Receiver Gain	44.00	***Solvent***	CHLOROFORM-d			***Spectrum Offset (Hz)***	5413.0830
Sweep Width (Hz)	16534.39						

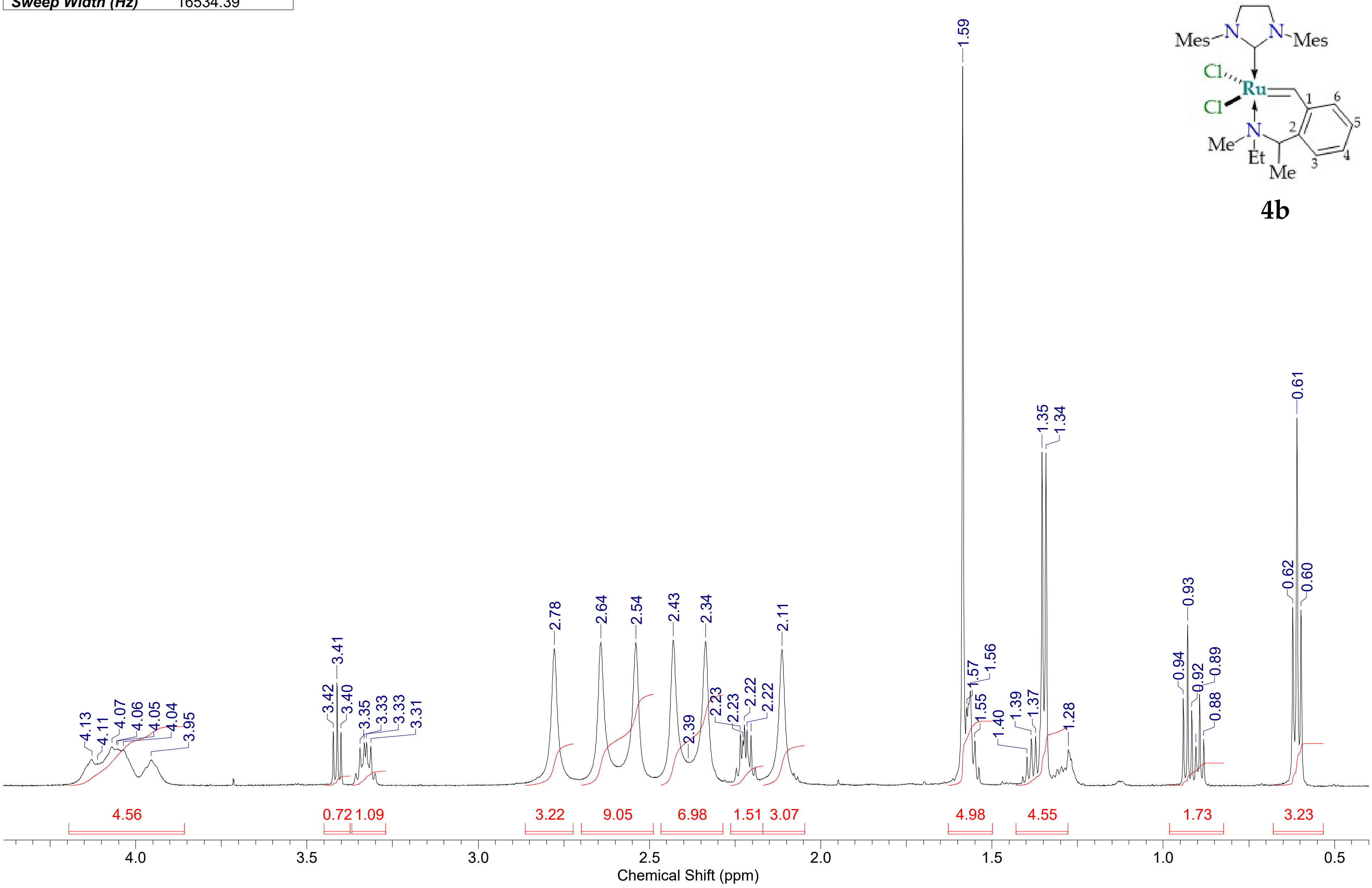

^{1}H spectrum of compound **4b**

Acquisition Time (sec)	2.1845	***Comment***	5 mm CPPBBO BB-1H/19F/D Z-GRD Z125869/0025			***Date***	07 Mar 2017 11:35:28
Date Stamp	07 Mar 2017 11:35:28						
File Name							
Frequency (MHz)	500.13	***Nucleus***	1H	***Number of Transients***	1	***Origin***	spect
Original Points Count	32768	***Owner***	nmrsu	***Points Count***	262144	***Pulse Sequence***	zg
Receiver Gain	31.21	***SW(cyclical) (Hz)***	15000.00	***Solvent***	DICHLOROMETHANE-d2		
Spectrum Offset (Hz)	3088.5078	***Sweep Width (Hz)***	14999.94	***Temperature (degree C)***	25.072		

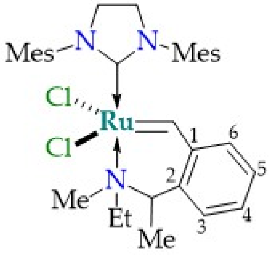

4b

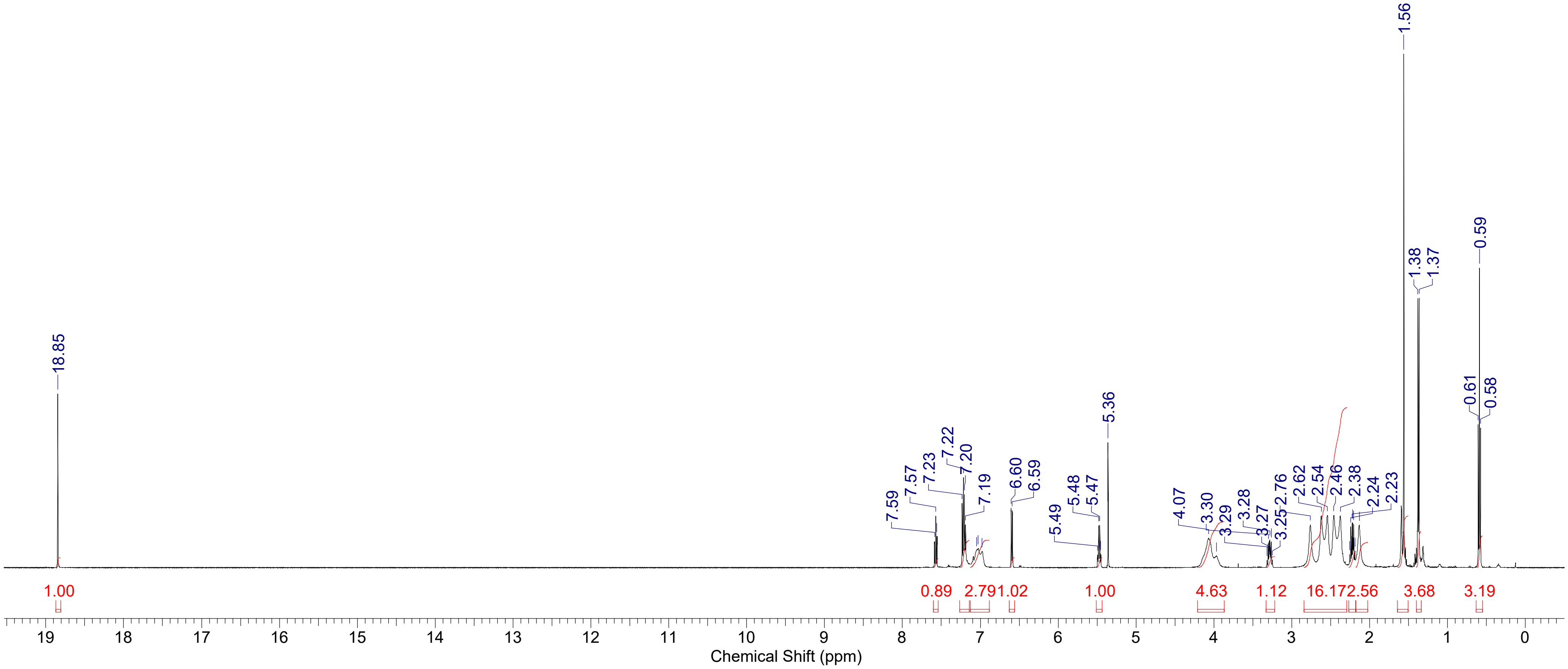

^{1}H spectrum of compound **4b**

Acquisition Time (sec)	2.1845	Comment	5 mm CPPBBO BB-1H/19F/D Z-GRD Z125869/0025			Date	07 Mar 2017 11:35:28
Date Stamp	07 Mar 2017 11:35:28						
File Name							
Frequency (MHz)	500.13	Nucleus	1H	Number of Transients	1	Origin	spect
Original Points Count	32768	Owner	nmrsu	Points Count	262144	Pulse Sequence	zg
Receiver Gain	31.21	SW(cyclical) (Hz)	15000.00	Solvent	DICHLOROMETHANE-d2		
Spectrum Offset (Hz)	3088.5078	Sweep Width (Hz)	14999.94	Temperature (degree C)	25.072		

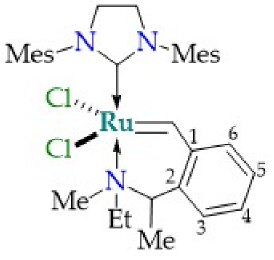

4b

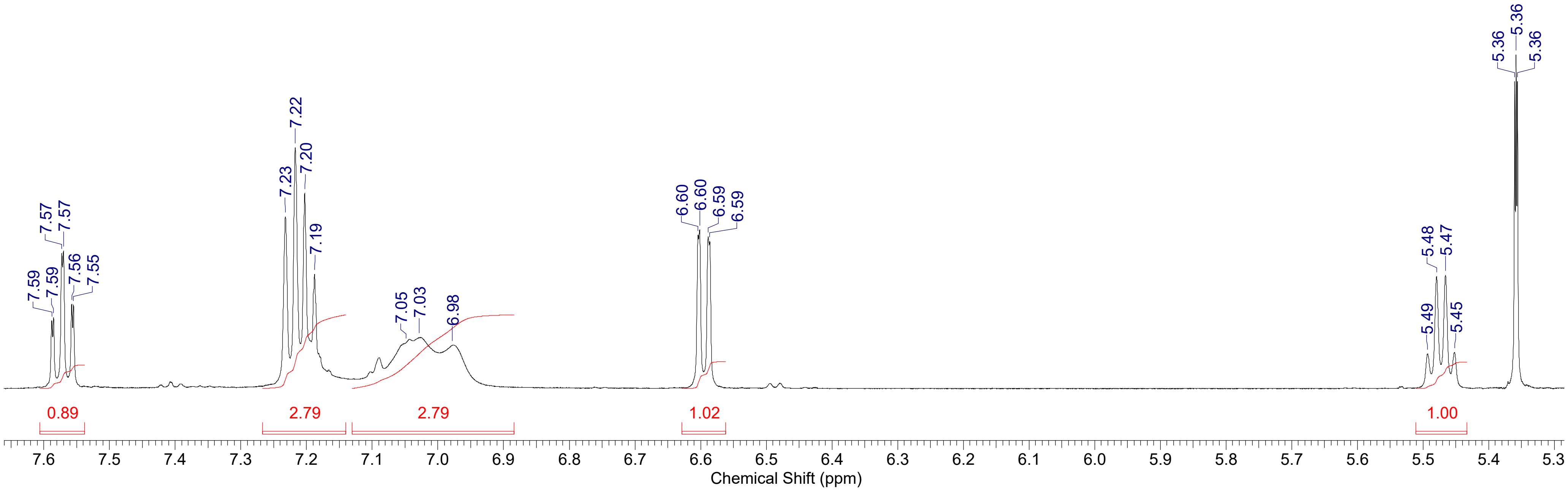

^{1}H spectrum of compound **4b**

Acquisition Time (sec)	2.1845	***Comment***	5 mm CPPBBO BB-1H/19F/D Z-GRD Z125869/0025			***Date***	07 Mar 2017 11:35:28
Date Stamp	07 Mar 2017 11:35:28						
File Name							
Frequency (MHz)	500.13	***Nucleus***	1H	***Number of Transients***	1	***Origin***	spect
Original Points Count	32768	***Owner***	nmrsu	***Points Count***	262144	***Pulse Sequence***	zg
Receiver Gain	31.21	***SW(cyclical) (Hz)***	15000.00	***Solvent***	DICHLOROMETHANE-d2		
Spectrum Offset (Hz)	3088.5078	***Sweep Width (Hz)***	14999.94	***Temperature (degree C)***	25.072		

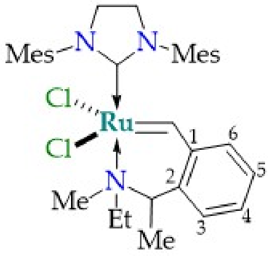

4b

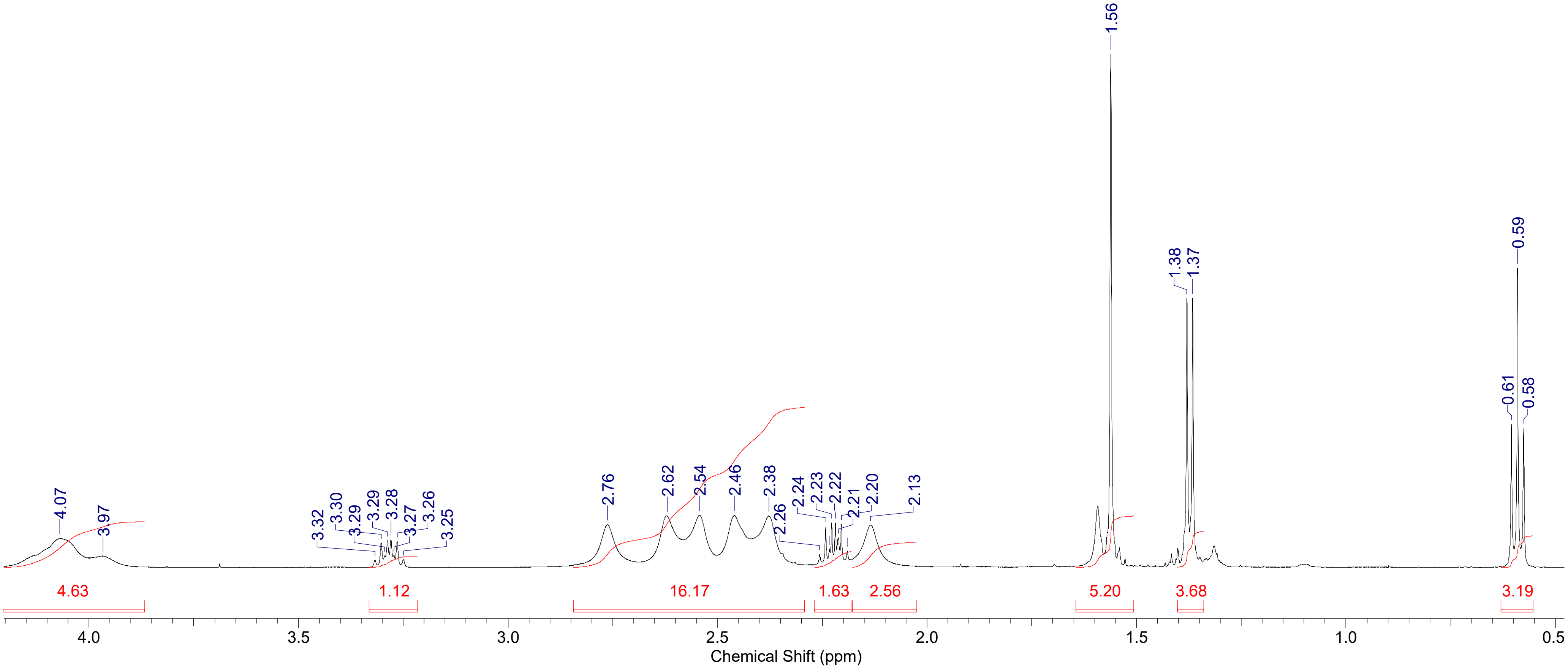

^{13}C spectrum of compound **4b**

Acquisition Time (sec)	1.7197	***Comment***	single pulse decoupled gated NOE			***Date***	19 Jan 2023 06:46:31
Date Stamp	19 Jan 2023 05:44:01			***File Name***			
Frequency (MHz)	150.91	***Nucleus***	13C	***Number of Transients***	4000	***Origin***	ECA 600
Original Points Count	131072	***Owner***	CKP	***Points Count***	131072	***Pulse Sequence***	single_pulse_dec
Receiver Gain	56.00	***Solvent***	CHLOROFORM-d			***Spectrum Offset (Hz)***	25629.8613
Sweep Width (Hz)	76219.52						

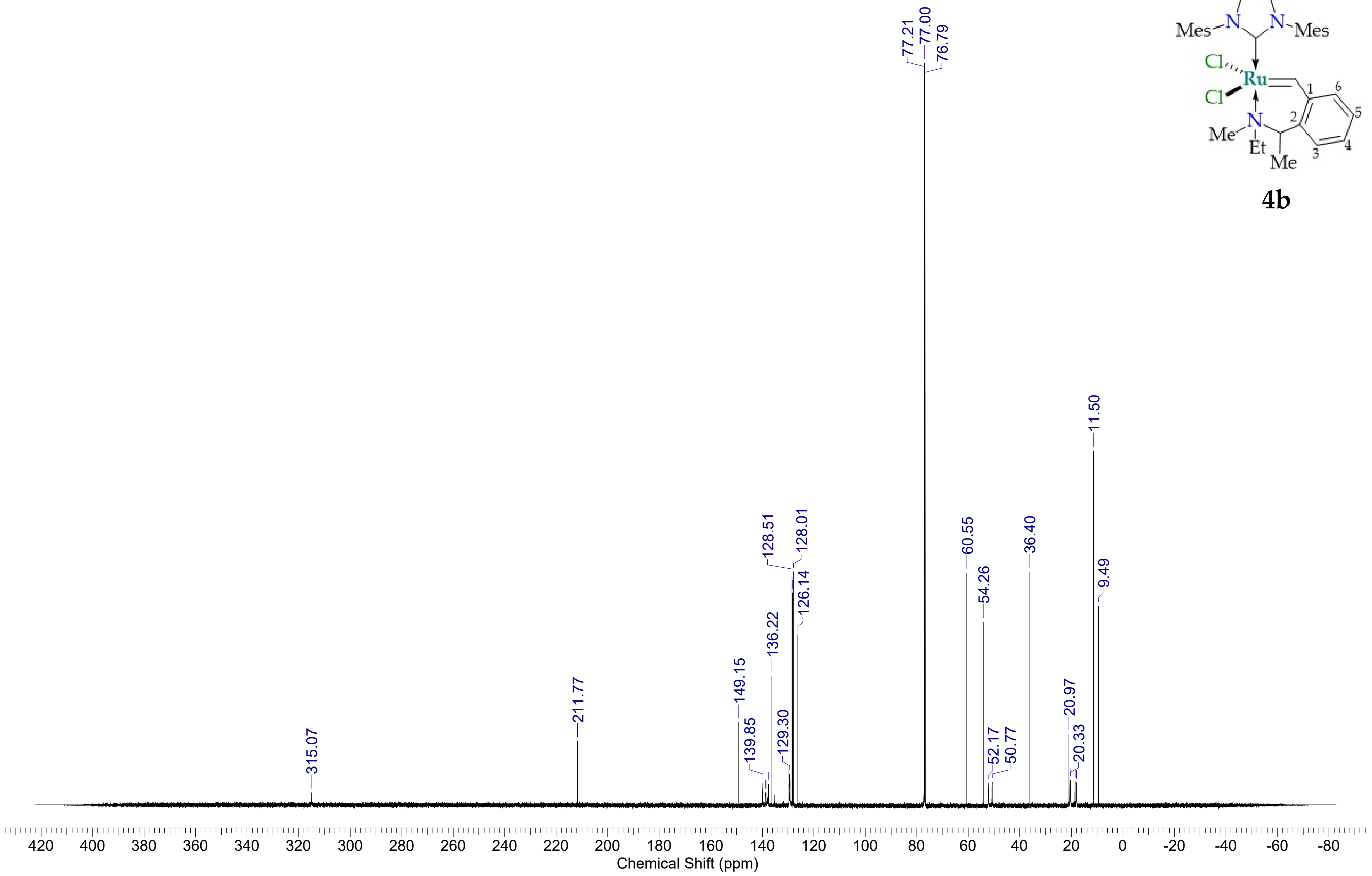

4b

^{13}C spectrum of compound **4b**

**Formula** $C_{15}H_{22}Cl_2N_3Ru$?	**FW** 416.3313+?

**Acquisition Time (sec)**	0.6921	**Comment**	single pulse decoupled gated NOE	**Date**	12 Jan 2023 09:51:21
**Date Stamp**	12 Jan 2023 08:48:40			**File Name**	C:\USERS\ALEXA\DESKTOP\4B 13CFZ1444-2 (2).JDF
**Frequency (MHz)**	150.91	**Nucleus**	13C	**Number of Transients**	3401
**Origin**	ECA 600				
**Original Points Count**	32768	**Owner**	CKP	**Points Count**	32768
**Pulse Sequence**	single_pulse_dec				
**Receiver Gain**	56.00	**Solvent**	CHLOROFORM-d	**Spectrum Offset (Hz)**	15065.9502
**Sweep Width (Hz)**	47348.49				

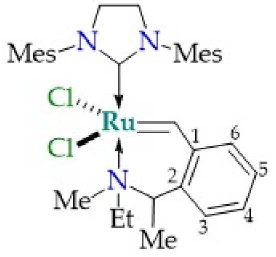

4b

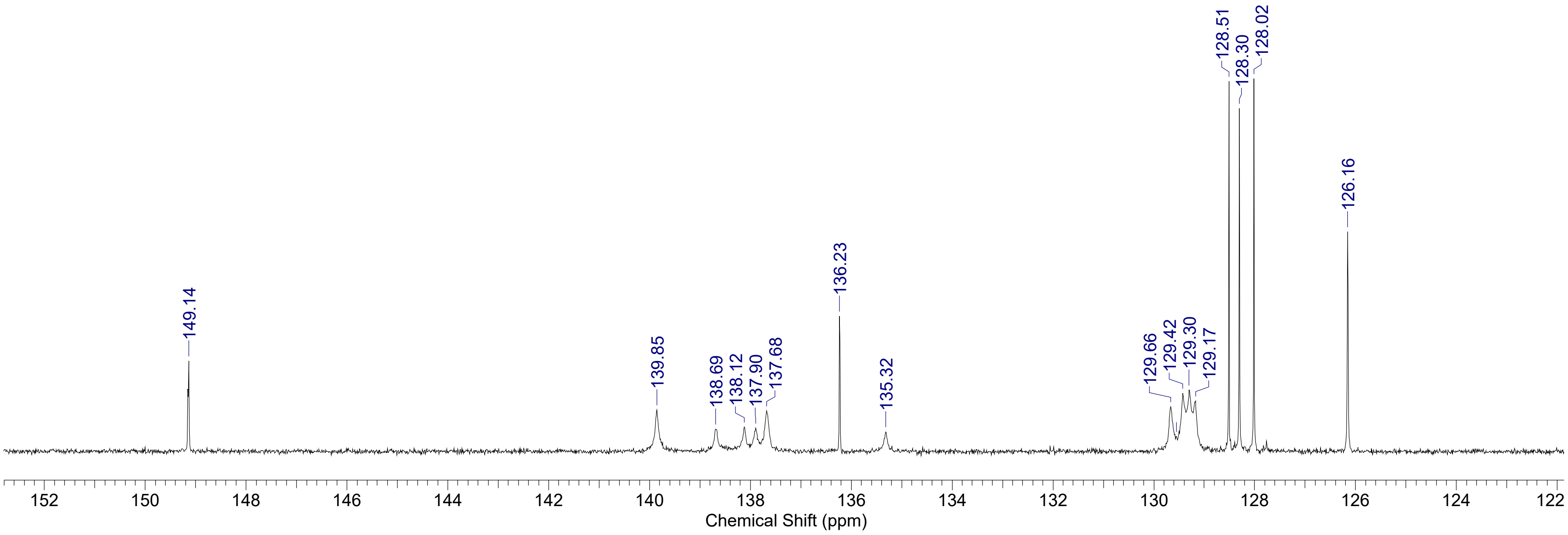

^{13}C spectrum of compound **4b**

**Formula** $C_{15}H_{22}Cl_2N_3Ru?$	**FW** 416.3313+?

**Acquisition Time (sec)**	0.6921	**Comment**	single pulse decoupled gated NOE	**Date**	12 Jan 2023 09:51:21
**Date Stamp**	12 Jan 2023 08:48:40	**File Name**	C:\USERS\ALEXA\DESKTOP\4B 13CFZ1444-2 (2).JDF		
**Frequency (MHz)**	150.91	**Nucleus**	13C	**Number of Transients**	3401
**Origin**	ECA 600				
**Original Points Count**	32768	**Owner**	CKP	**Points Count**	32768
**Pulse Sequence**	single_pulse_dec				
**Receiver Gain**	56.00	**Solvent**	CHLOROFORM-d	**Spectrum Offset (Hz)**	15065.9502
**Sweep Width (Hz)**	47348.49				

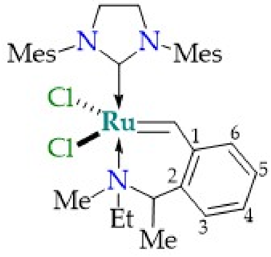

4b

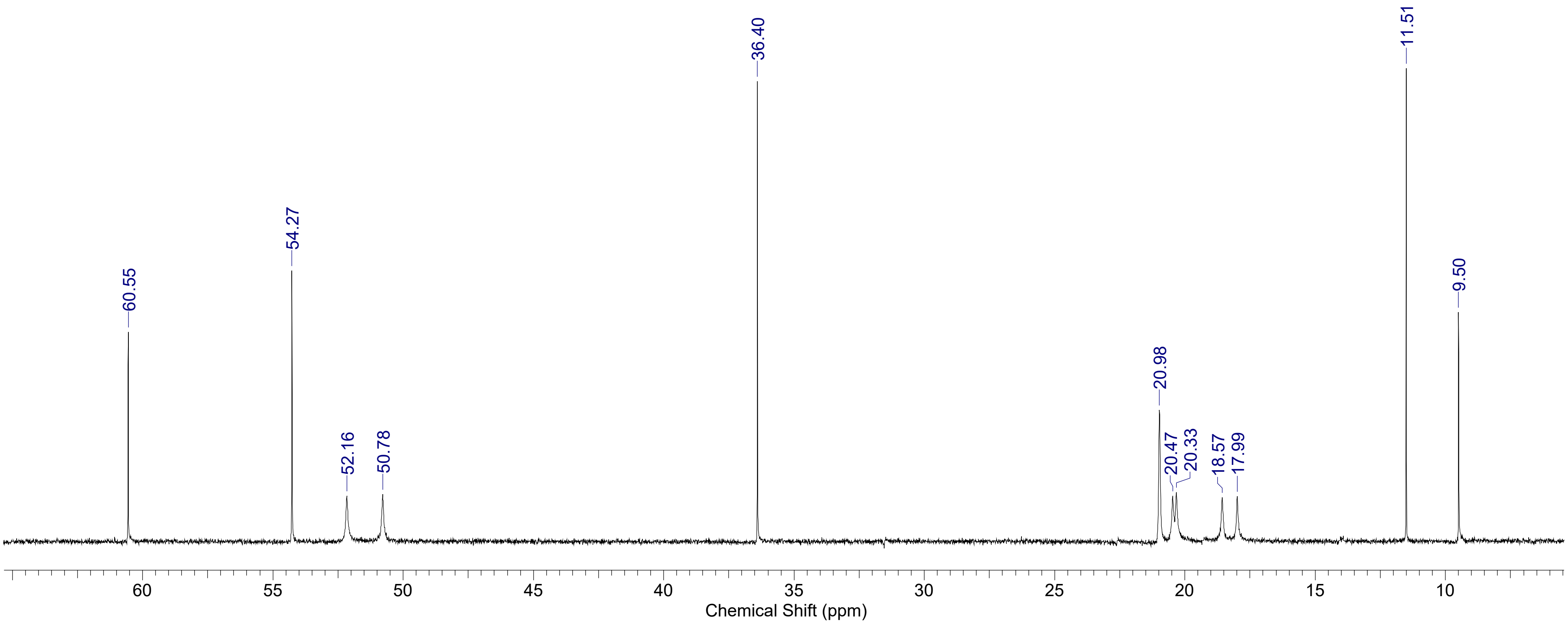

DEPT-135 spectrum of compound **4b**

Acquisition Time (sec)	0.8039	***Comment***	5 mm CPPBBO BB-1H/19F/D Z-GRD Z125869/0025			***Date***	07 Mar 2017 12:18:08
Date Stamp	07 Mar 2017 12:18:08						
File Name							
Frequency (MHz)	125.76	***Nucleus***	13C	***Number of Transients***	571	***Origin***	spect
Original Points Count	32768	***Owner***	nmrsu	***Points Count***	32768	***Pulse Sequence***	jmod
Receiver Gain	189.77	***SW(cyclical) (Hz)***	40760.87	***Solvent***	DICHLOROMETHANE-d2		
Spectrum Offset (Hz)	20121.2441	***Sweep Width (Hz)***	40759.63	***Temperature (degree C)***	25.087		

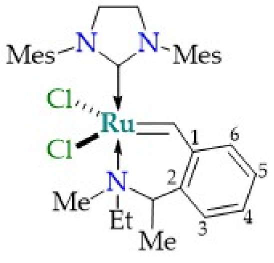

4b

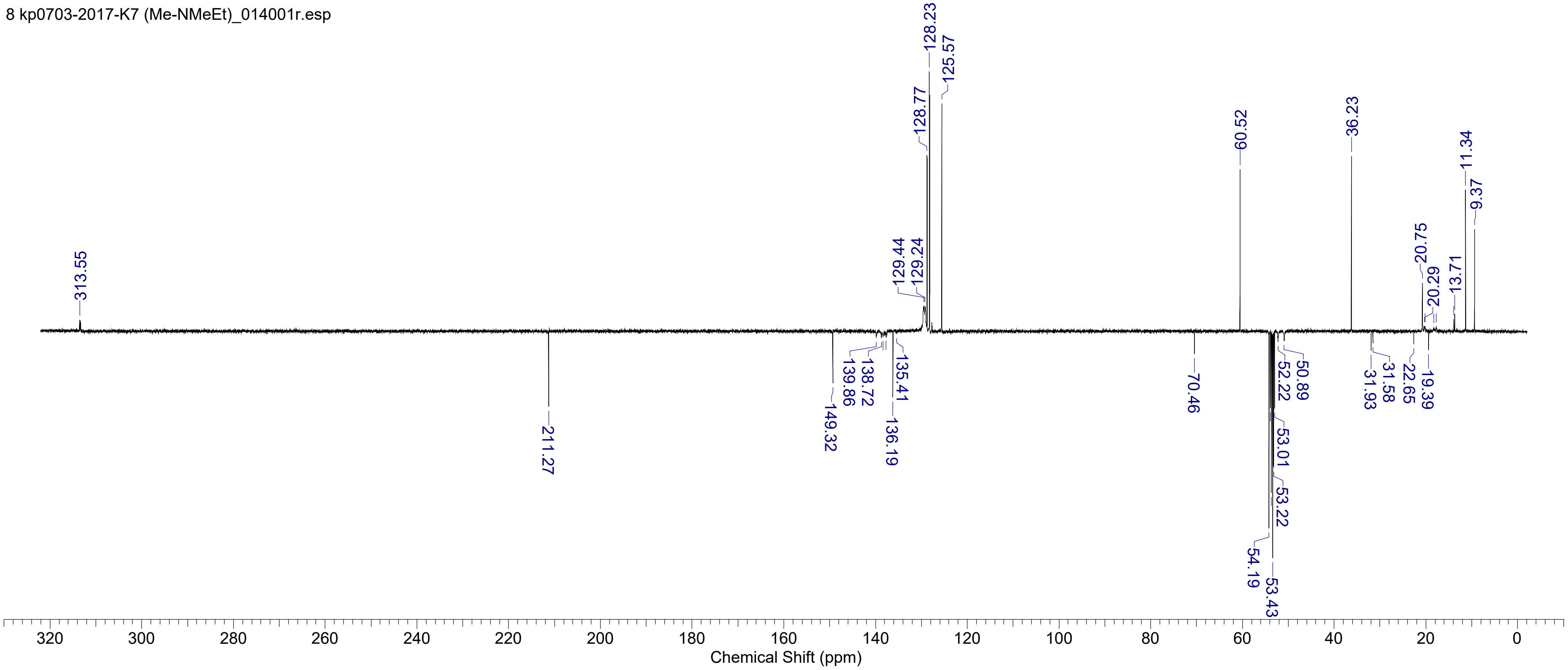

DEPT-135 spectrum of compound **4b**

Acquisition Time (sec)	0.8039	***Comment***	5 mm CPPBBO BB-1H/19F/D Z-GRD Z125869/0025			***Date***	07 Mar 2017 12:18:08
Date Stamp	07 Mar 2017 12:18:08						
File Name							
Frequency (MHz)	125.76	***Nucleus***	13C	***Number of Transients***	571	***Origin***	spect
Original Points Count	32768	***Owner***	nmrsu	***Points Count***	32768	***Pulse Sequence***	jmod
Receiver Gain	189.77	***SW(cyclical) (Hz)***	40760.87	***Solvent***	DICHLOROMETHANE-d2		
Spectrum Offset (Hz)	20121.2441	***Sweep Width (Hz)***	40759.63	***Temperature (degree C)***	25.087		

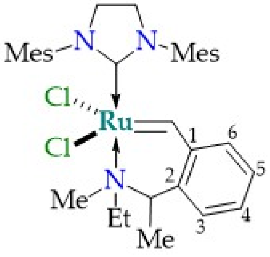

4b

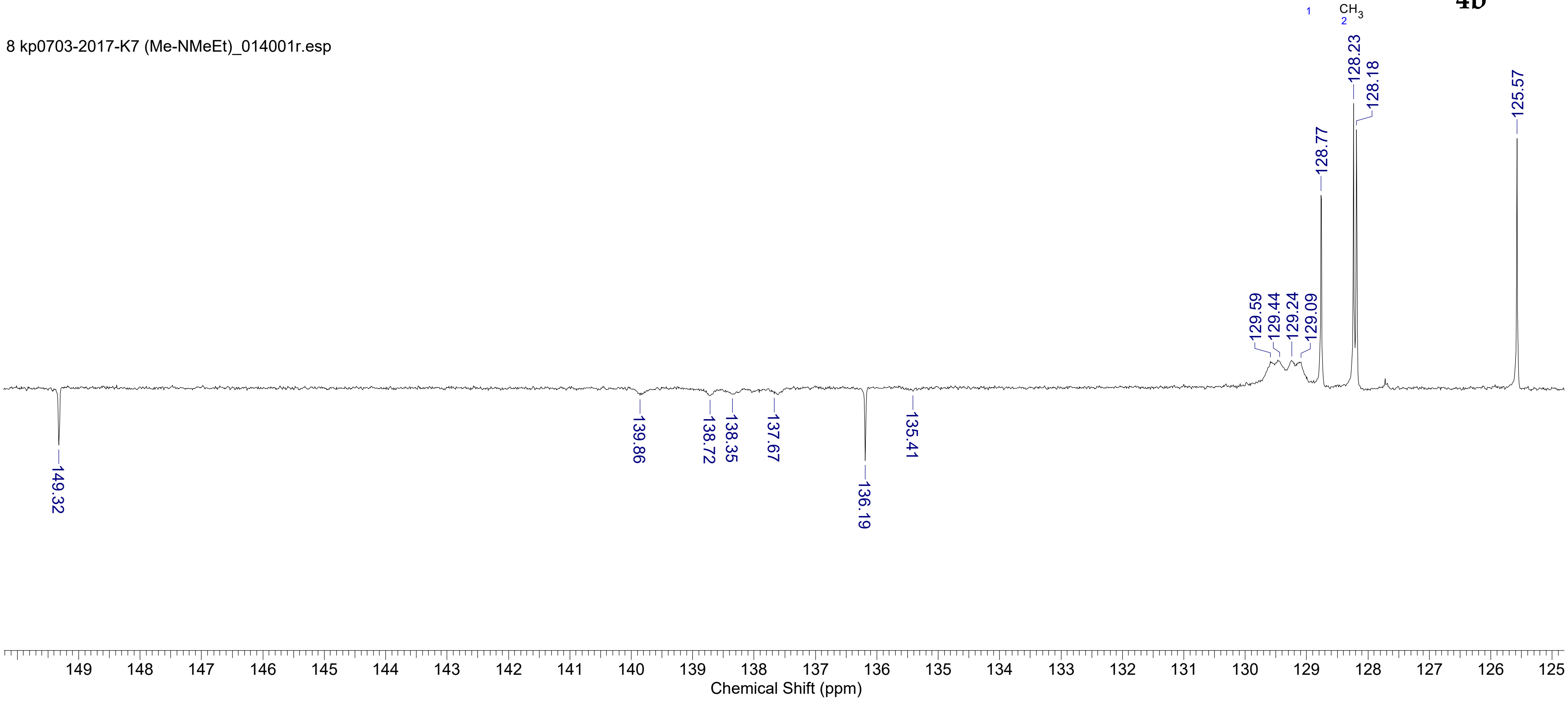

DEPT-135 spectrum of compound **4b**

Acquisition Time (sec)	0.8039	***Comment***	5 mm CPPBBO BB-1H/19F/D Z-GRD Z125869/0025			***Date***	07 Mar 2017 12:18:08
Date Stamp	07 Mar 2017 12:18:08						
File Name							
Frequency (MHz)	125.76	***Nucleus***	13C	***Number of Transients***	571	***Origin***	spect
Original Points Count	32768	***Owner***	nmrsu	***Points Count***	32768	***Pulse Sequence***	jmod
Receiver Gain	189.77	***SW(cyclical) (Hz)***	40760.87	***Solvent***	DICHLOROMETHANE-d2		
Spectrum Offset (Hz)	20121.2441	***Sweep Width (Hz)***	40759.63	***Temperature (degree C)***	25.087		

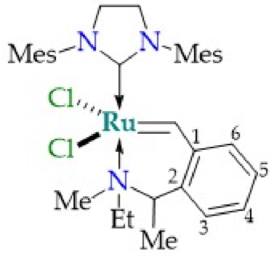

4b

8 kp0703-2017-K7 (Me-NMeEt)_014001r.esp

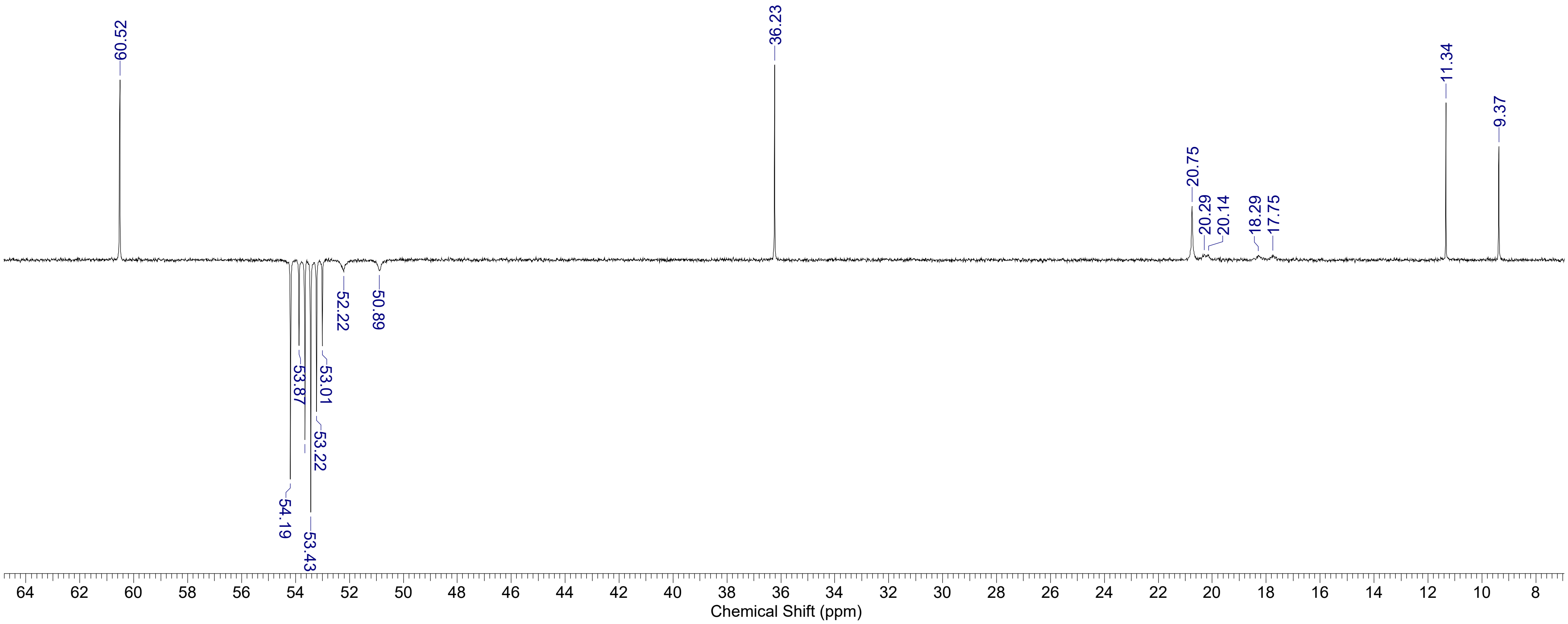

IR spectrum of compound **4b**

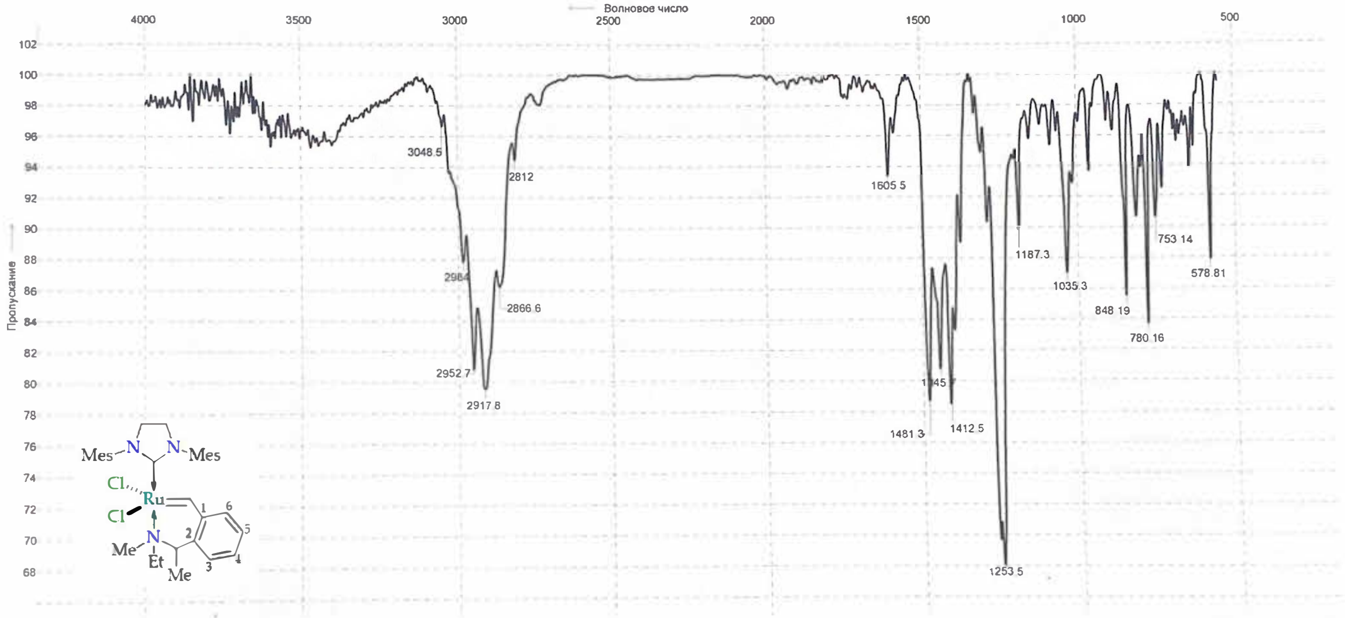

^{1}H spectrum of compound **4c**

Acquisition Time (sec)	1.9818	***Comment***	single_pulse	***Date***	14 Sep 2022 15:40:30		
Date Stamp	14 Sep 2022 15:25:37			***File Name***			
Frequency (MHz)	600.17	***Nucleus***	1H	***Number of Transients***	8	***Origin***	ECA 600
Original Points Count	32768	***Owner***	CKP	***Points Count***	32768	***Pulse Sequence***	single_pulse.ex2
Receiver Gain	42.00	***Solvent***	CHLOROFORM-d			***Spectrum Offset (Hz)***	5413.5879
Sweep Width (Hz)	16534.39						

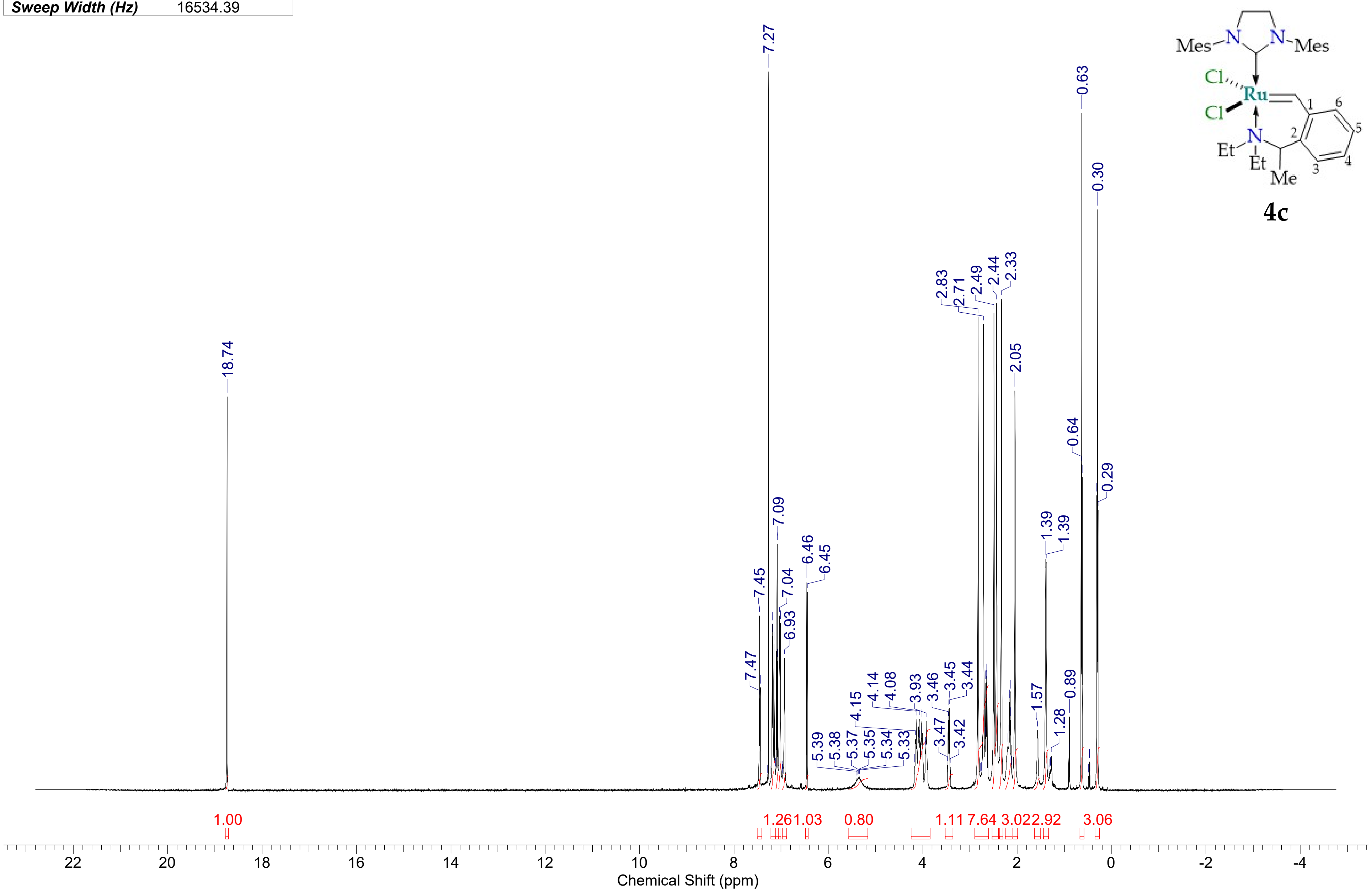

¹H spectrum of compound **4c**

Acquisition Time (sec)	1.9818	***Comment***	single_pulse	***Date***	14 Sep 2022 15:40:30		
Date Stamp	14 Sep 2022 15:25:37			***File Name***			
Frequency (MHz)	600.17	***Nucleus***	1H	***Number of Transients***	8	***Origin***	ECA 600
Original Points Count	32768	***Owner***	CKP	***Points Count***	32768	***Pulse Sequence***	single_pulse.ex2
Receiver Gain	42.00	***Solvent***	CHLOROFORM-d			***Spectrum Offset (Hz)***	5413.5879
Sweep Width (Hz)	16534.39						

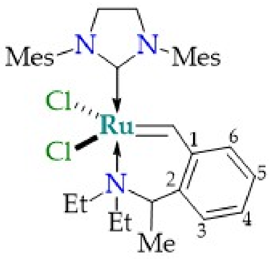

4c

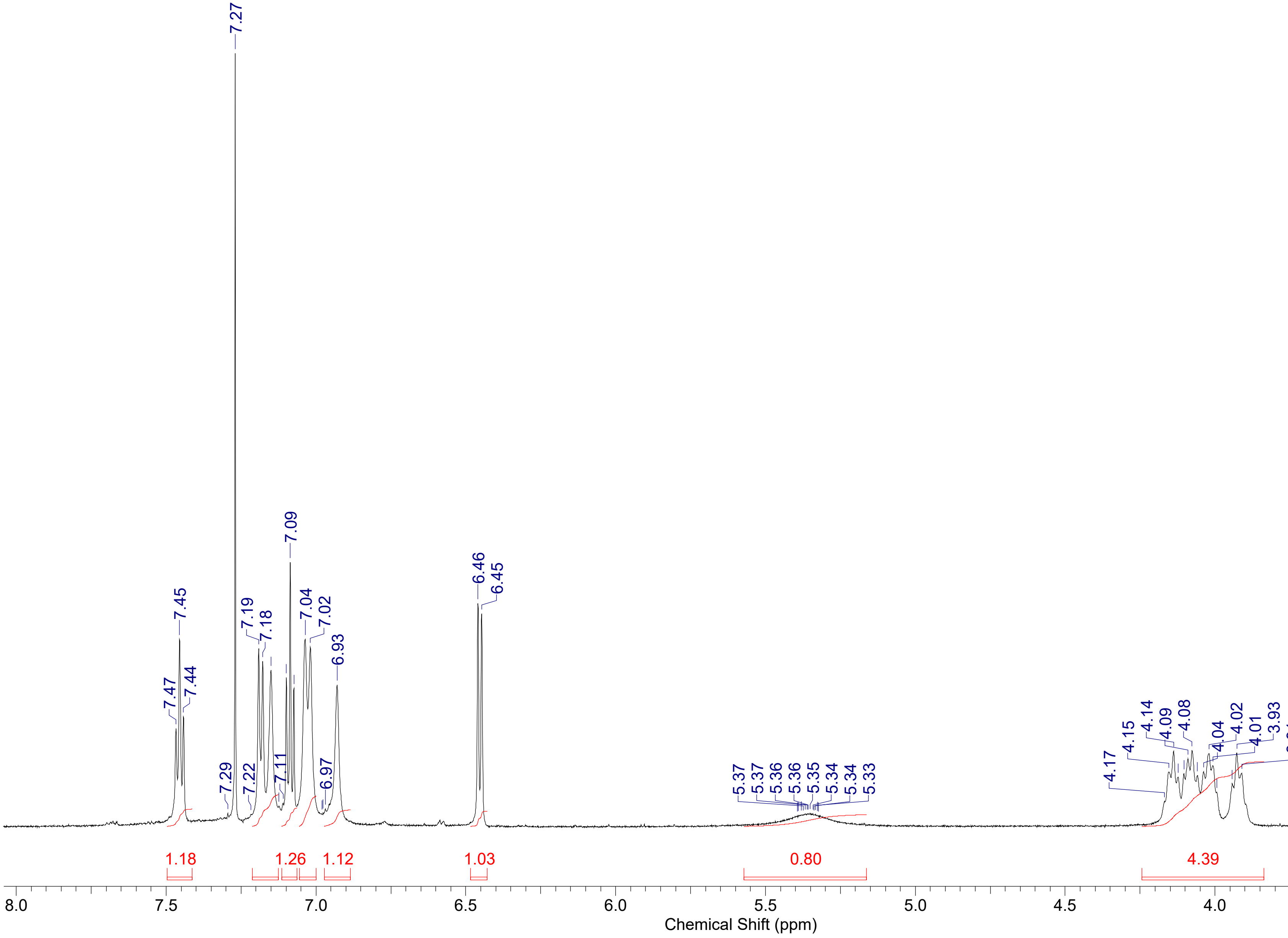

^{1}H spectrum of compound **4c**

Acquisition Time (sec)	1.9818	***Comment***	single_pulse	***Date***	14 Sep 2022 15:40:30		
Date Stamp	14 Sep 2022 15:25:37			***File Name***			
Frequency (MHz)	600.17	***Nucleus***	1H	***Number of Transients***	8	***Origin***	ECA 600
Original Points Count	32768	***Owner***	CKP	***Points Count***	32768	***Pulse Sequence***	single_pulse.ex2
Receiver Gain	42.00	***Solvent***	CHLOROFORM-d			***Spectrum Offset (Hz)***	5413.5879
Sweep Width (Hz)	16534.39						

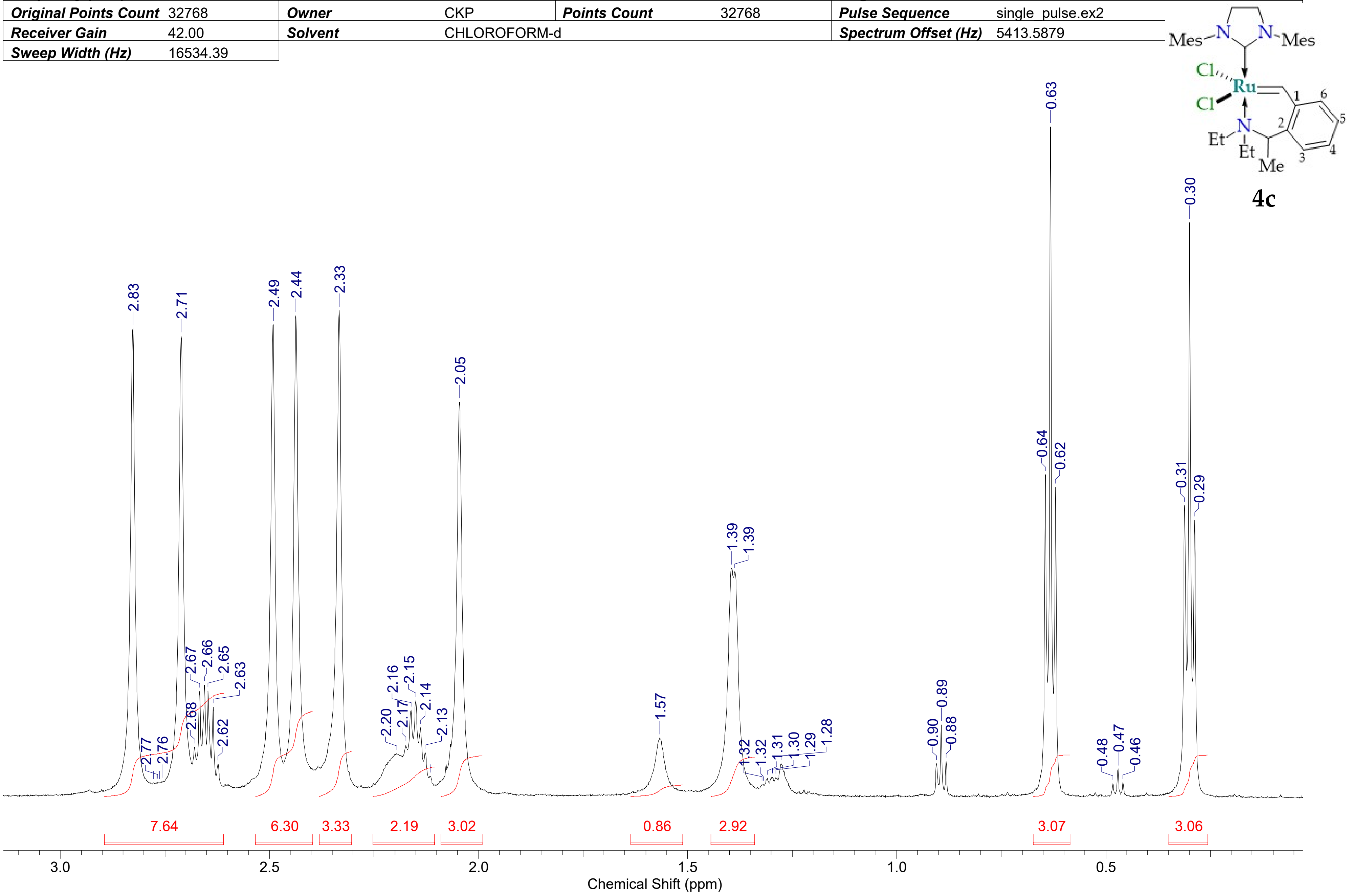

^{13}C spectrum of compound **4c**

Acquisition Time (sec)	1.7197	***Comment***	single pulse decoupled gated NOE			***Date***	19 Jan 2023 06:46:31
Date Stamp				***File Name***			
Frequency (MHz)	150.91	***Nucleus***	13C	***Number of Transients***	4000	***Origin***	ECA 600
Original Points Count	131072	***Owner***	CKP	***Points Count***	131072	***Pulse Sequence***	single_pulse_dec
Receiver Gain	56.00	***Solvent***	CHLOROFORM-d			***Spectrum Offset (Hz)***	25629.8613
Sweep Width (Hz)	76219.52						

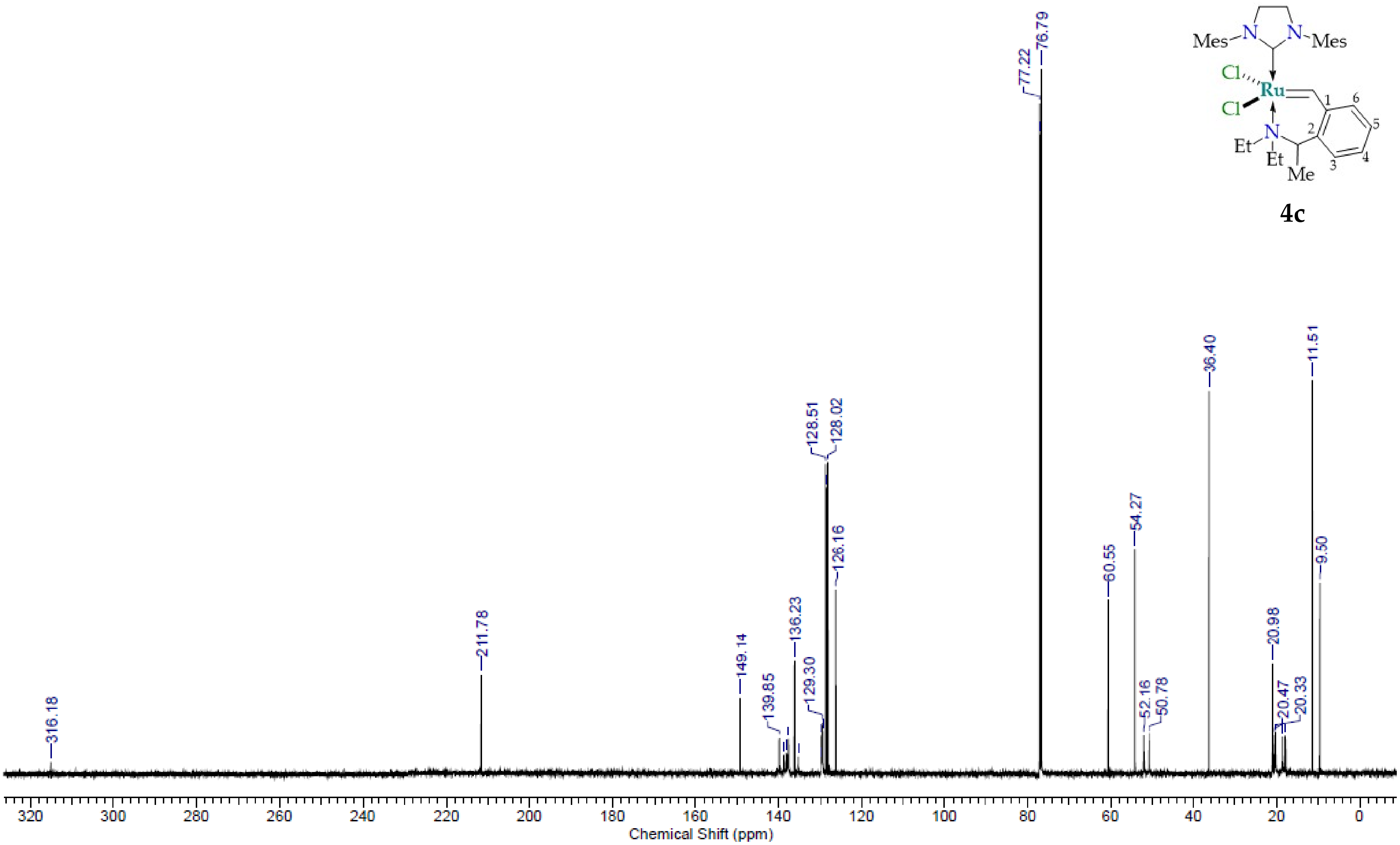

4c

^{13}C spectrum of compound **4c**

**Formula** $C_{15}H_{22}Cl_2N_3Ru$?	**FW** 416.3313+?

**Acquisition Time (sec)**	0.6921	**Comment**	single pulse decoupled gated NOE	**Date**	12 Jan 2023 09:51:21
**Date Stamp**	12 Jan 2023 08:48:40	**File Name**			
**Frequency (MHz)**	150.91	**Nucleus**	13C	**Number of Transients**	3401
**Origin**	ECA 600				
**Original Points Count**	32768	**Owner**	CKP	**Points Count**	32768
**Pulse Sequence**	single_pulse_dec				
**Receiver Gain**	56.00	**Solvent**	CHLOROFORM-d	**Spectrum Offset (Hz)**	15065.9502
**Sweep Width (Hz)**	47348.49				

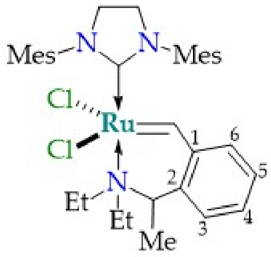

4c

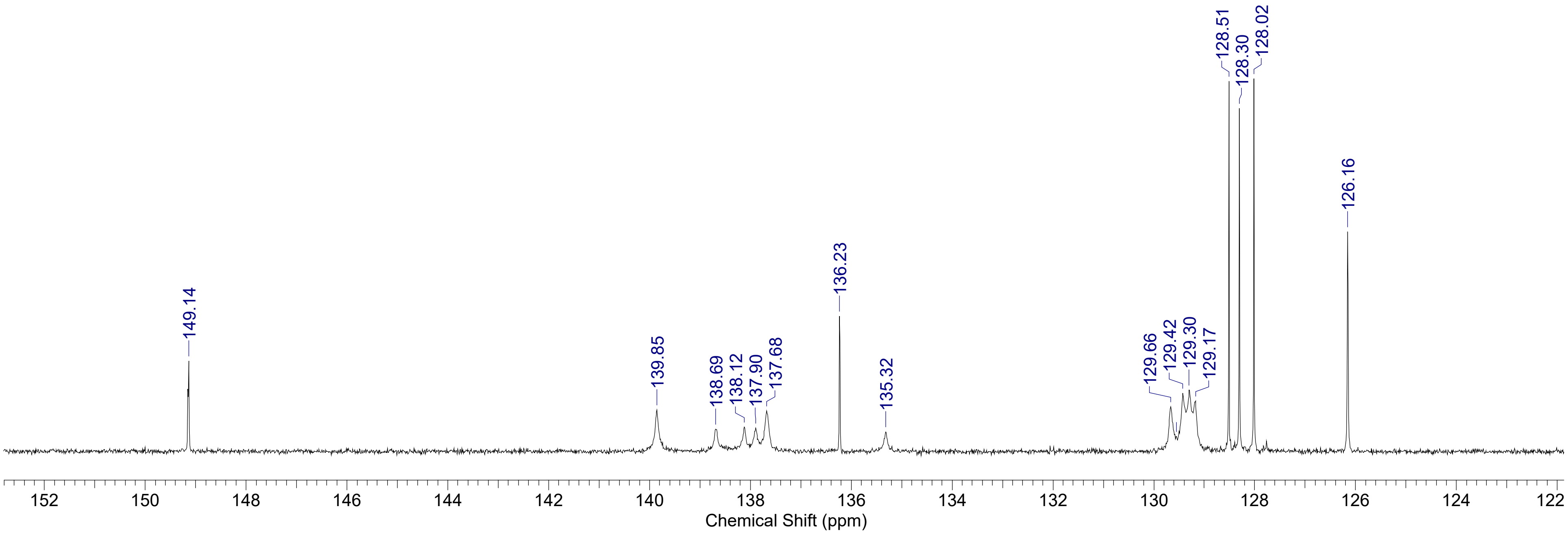

^{13}C spectrum of compound **4c**

**Formula** $C_{15}H_{22}Cl_2N_3Ru$?	**FW** 416.3313+?

Acquisition Time (sec)	0.6921	***Comment***	single pulse decoupled gated NOE			***Date***	12 Jan 2023 09:51:21
Date Stamp	12 Jan 2023 08:48:40			***File Name***			
Frequency (MHz)	150.91	***Nucleus***	13C	***Number of Transients***	3401	***Origin***	ECA 600
Original Points Count	32768	***Owner***	CKP	***Points Count***	32768	***Pulse Sequence***	single_pulse_dec
Receiver Gain	56.00	***Solvent***	CHLOROFORM-d			***Spectrum Offset (Hz)***	15065.9502
Sweep Width (Hz)	47348.49						

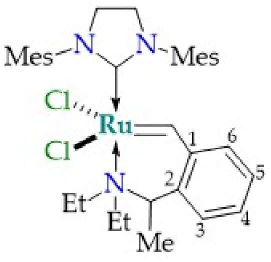

4c

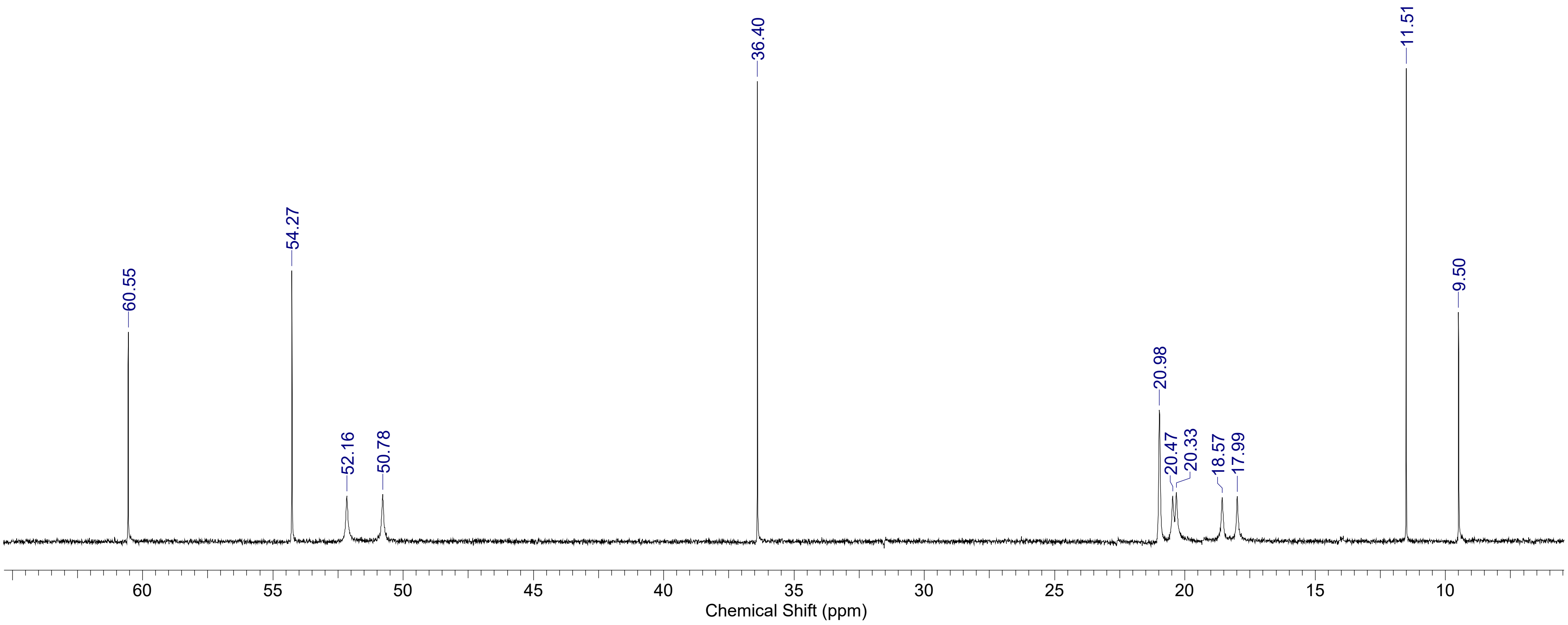

IR spectrum of compound **4c**

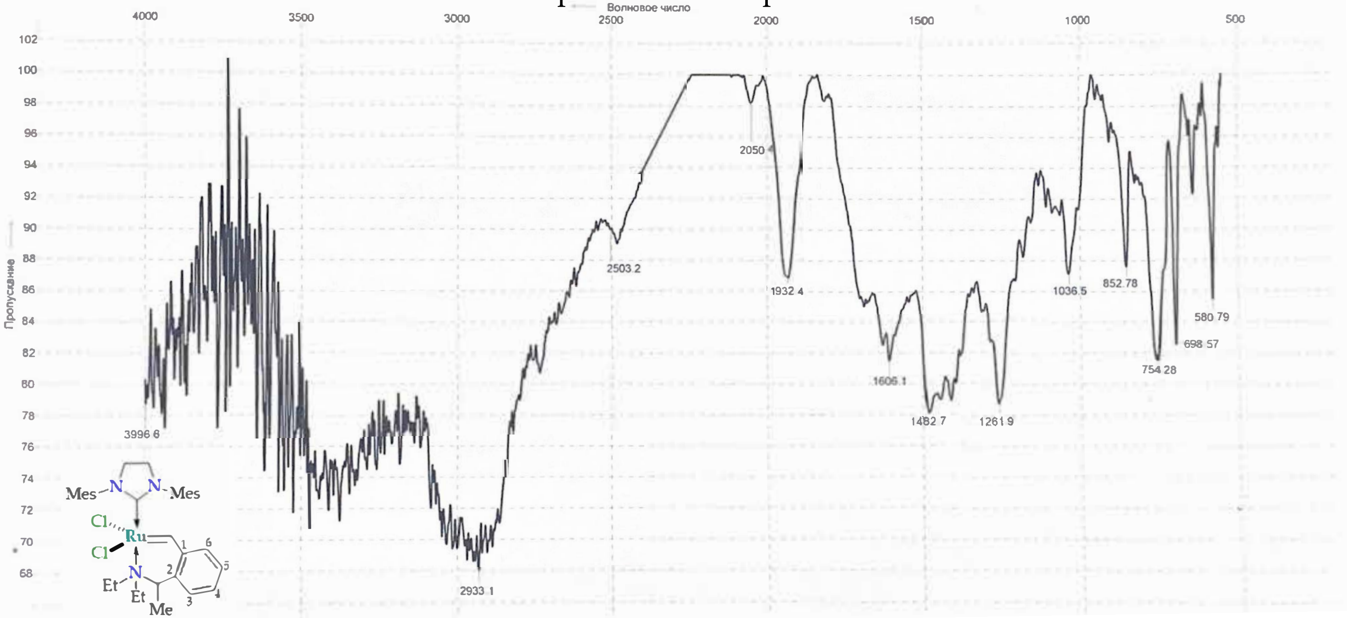

MALDI-ToF MS of compound **4c**

Display Report

Analysis Info

Analysis Name	D:\Data\Surzhikova\MVGA-4c.d	Acquisition Date	12.01.2023 13:17:40
Method	tune_100-1200.m	Operator	BDAL@DE
Sample Name		Instrument / Ser#	maXis 43
Comment			

Acquisition Parameter

Source Type	ESI	Ion Polarity	Positive	Set Nebulizer	1.0 Bar
Focus	Active			Set Dry Heater	200 °C
Scan Begin	50 m/z	Set Capillary	4500 V	Set Dry Gas	4.0 l/min
Scan End	1800 m/z	Set End Plate Offset	-500 V	Set Divert Valve	Waste

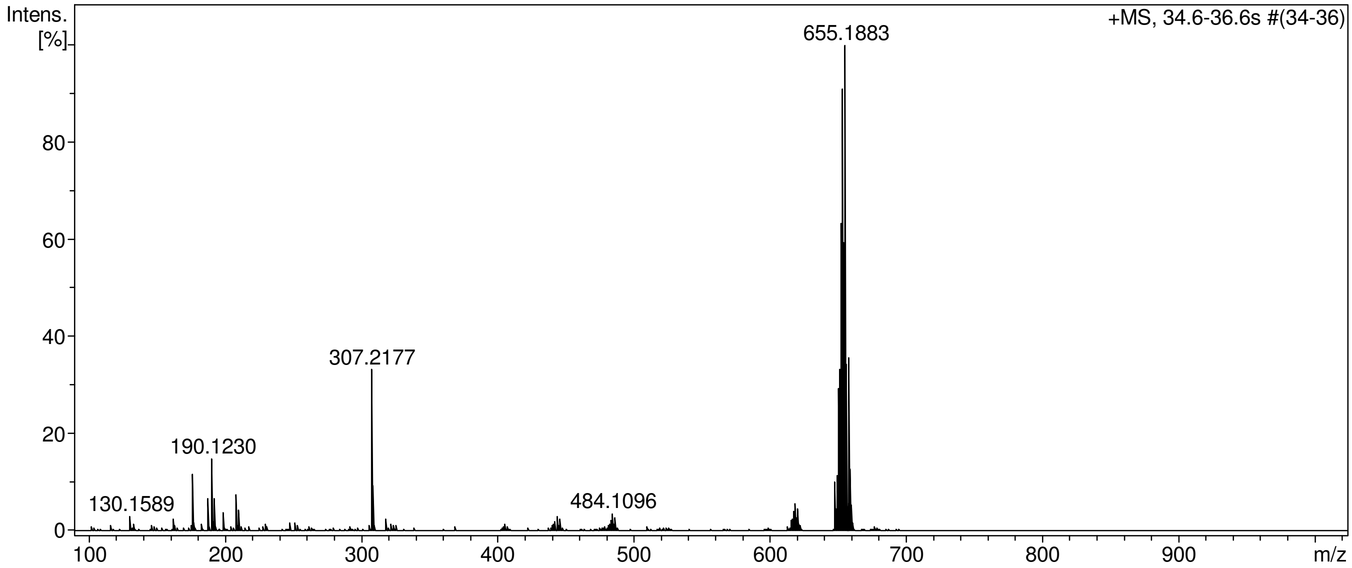

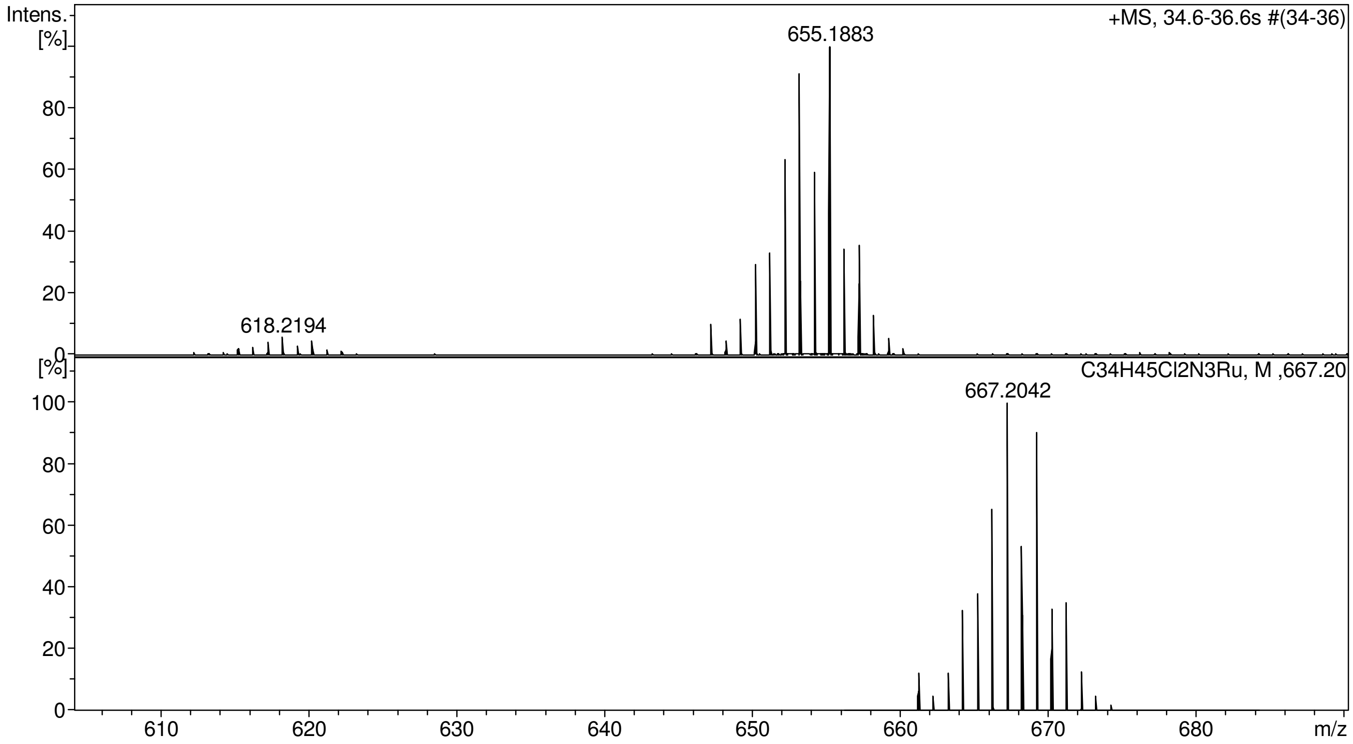

^{1}H spectrum of compound **4d**

Acquisition Time (sec)	1.9818	***Comment***	single_pulse	***Date***	27 Oct 2022 08:24:45		
Date Stamp	27 Oct 2022 08:10:30						
File Name						***Frequency (MHz)***	600.17
Nucleus	1H	***Number of Transients***	8	***Origin***	ECA 600	***Original Points Count***	32768
Owner	CKP	***Points Count***	32768	***Pulse Sequence***	single_pulse.ex2	***Receiver Gain***	40.00
Solvent	CHLOROFORM-d	***Spectrum Offset (Hz)***	5413.5884	***Sweep Width (Hz)***	16534.39		

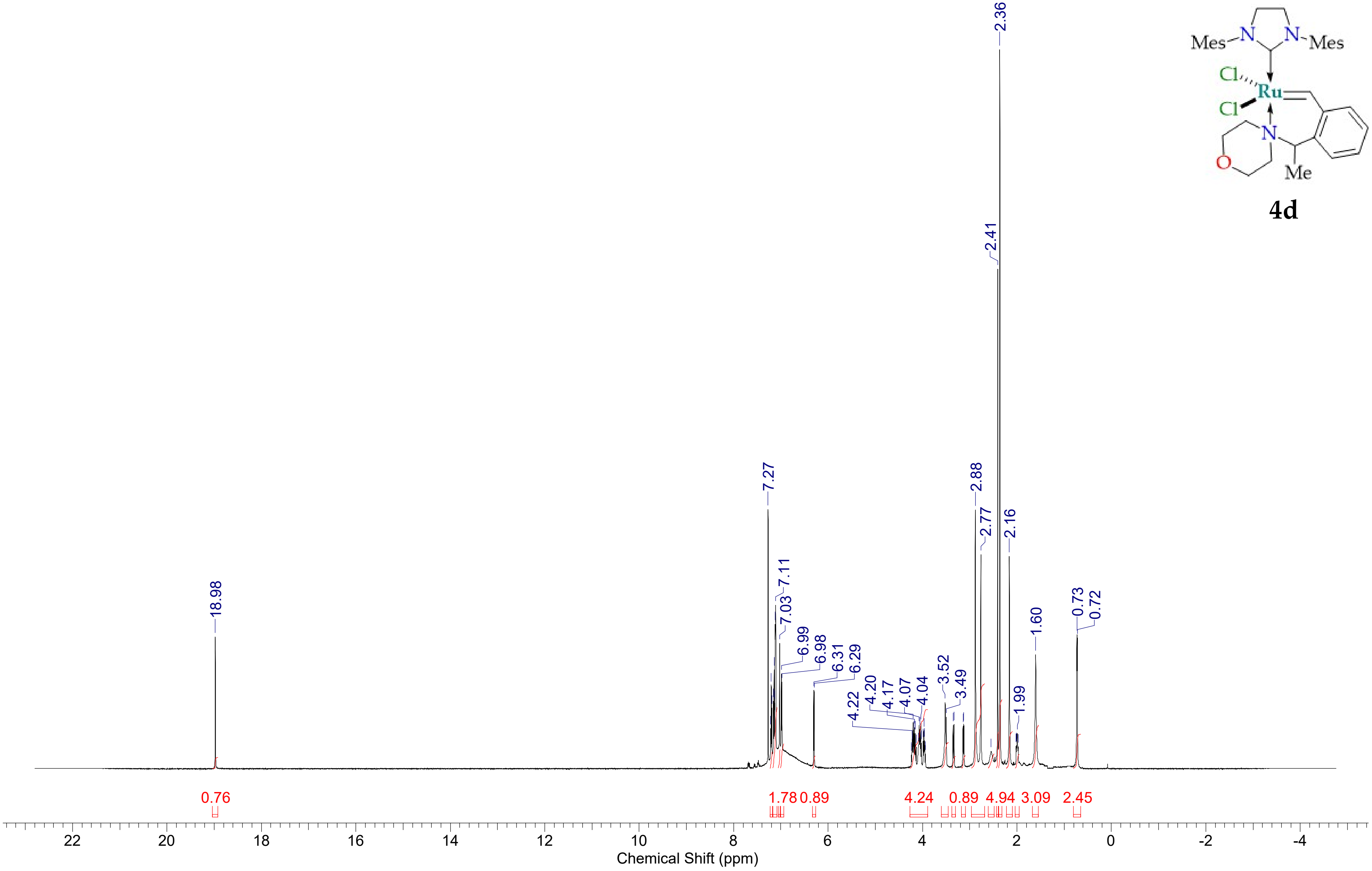

^{1}H spectrum of compound **4d**

Acquisition Time (sec)	1.9818	***Comment***	single_pulse	***Date***	27 Oct 2022 08:24:45		
Date Stamp	27 Oct 2022 08:10:30						
File Name						***Frequency (MHz)***	600.17
Nucleus	1H	***Number of Transients***	8	***Origin***	ECA 600	***Original Points Count***	32768
Owner	CKP	***Points Count***	32768	***Pulse Sequence***	single_pulse.ex2	***Receiver Gain***	40.00
Solvent	CHLOROFORM-d	***Spectrum Offset (Hz)***	5413.5884	***Sweep Width (Hz)***	16534.39		

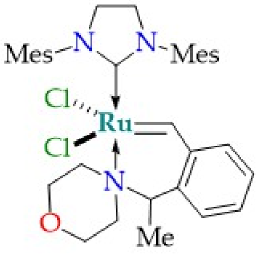

4d

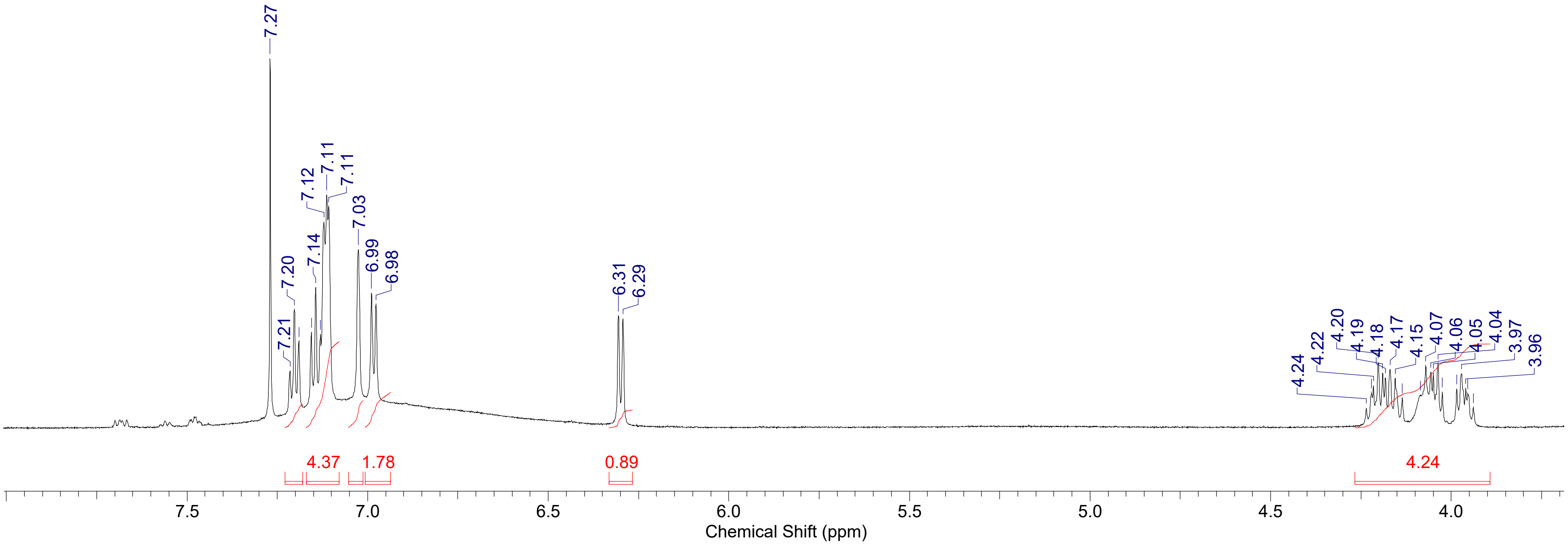

^{1}H spectrum of compound **4d**

Acquisition Time (sec)	1.9818	***Comment***	single_pulse	***Date***	27 Oct 2022 08:24:45		
Date Stamp	27 Oct 2022 08:10:30						
File Name						***Frequency (MHz)***	600.17
Nucleus	1H	***Number of Transients***	8	***Origin***	ECA 600	***Original Points Count***	32768
Owner	CKP	***Points Count***	32768	***Pulse Sequence***	single_pulse.ex2	***Receiver Gain***	40.00
Solvent	CHLOROFORM-d	***Spectrum Offset (Hz)***	5413.5884	***Sweep Width (Hz)***	16534.39		

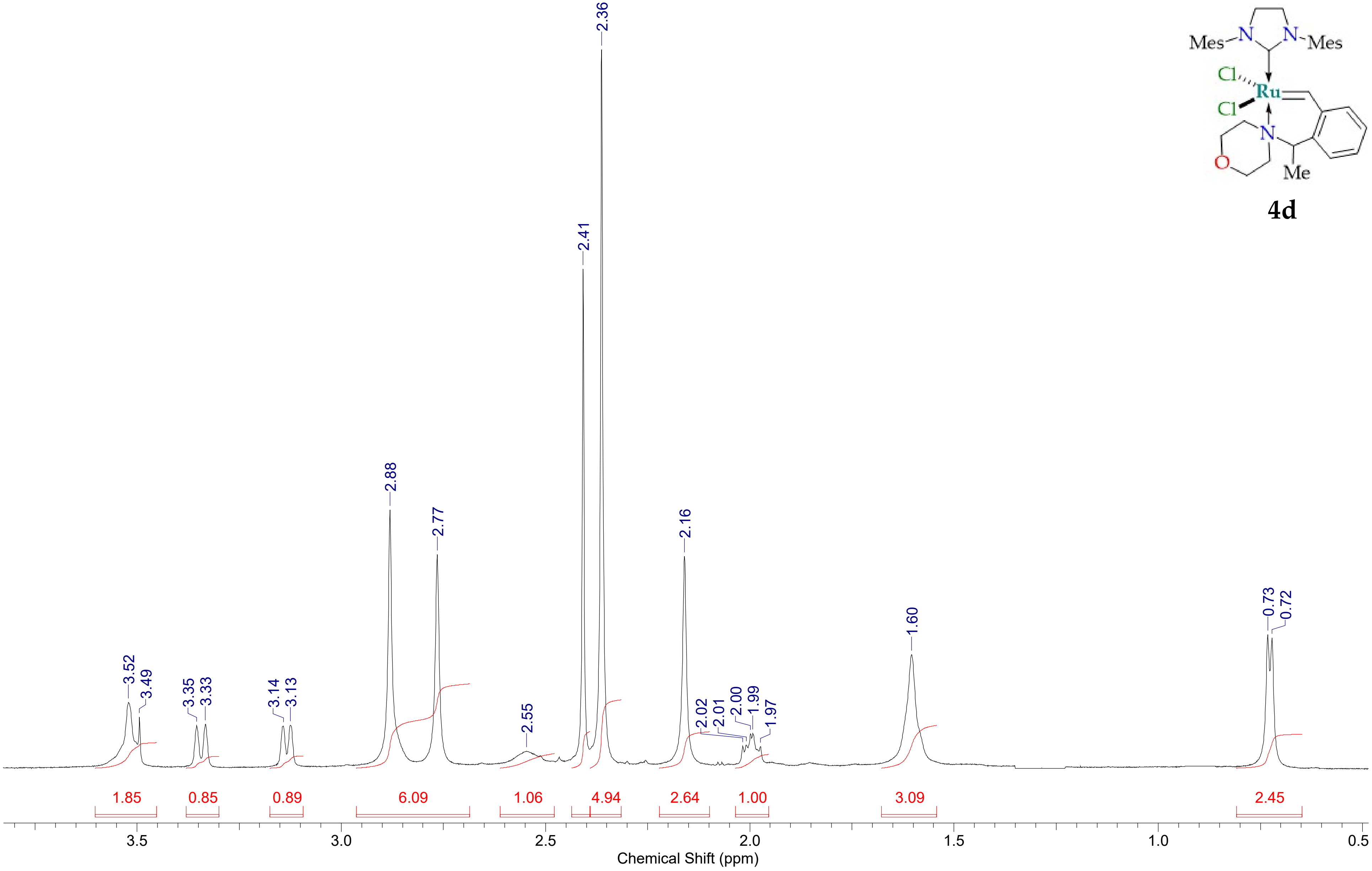

4d

^{13}C spectrum of compound **4d**

Acquisition Time (sec)	1.7197	***Comment***	single pulse decoupled gated NOE			***Date***	18 Jan 2023 17:46:18
Date Stamp	18 Jan 2023 16:43:47			***File Name***			
Frequency (MHz)	150.91	***Nucleus***	13C	***Number of Transients***	4000	***Origin***	ECA 600
Original Points Count	131072	***Owner***	CKP	***Points Count***	131072	***Pulse Sequence***	single_pulse_dec
Receiver Gain	54.00	***Solvent***	CHLOROFORM-d			***Spectrum Offset (Hz)***	25631.6035
Sweep Width (Hz)	76219.52						

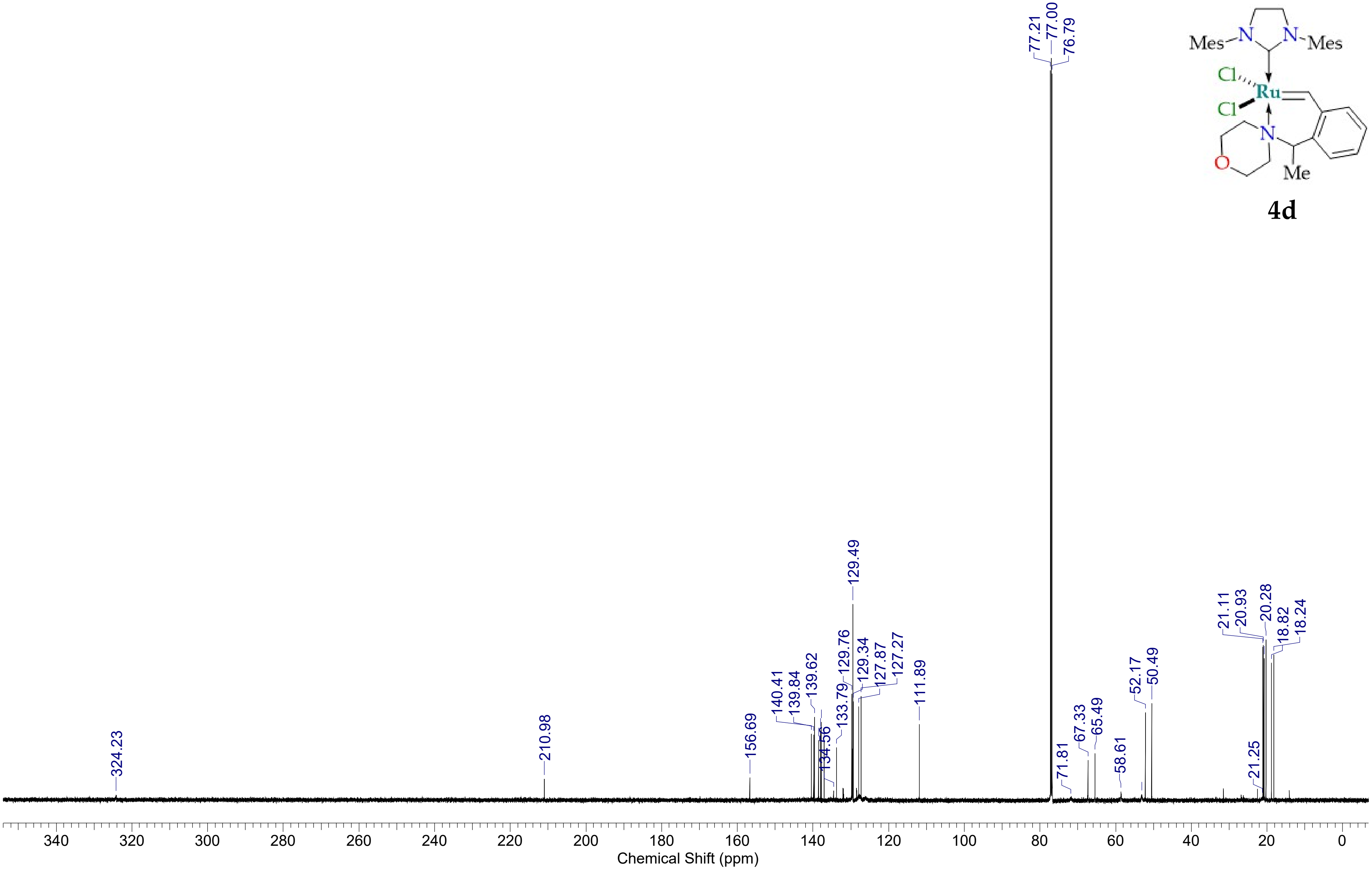

4d

^{13}C spectrum of compound **4d**

Acquisition Time (sec)	0.6921	***Comment***	single pulse decoupled gated NOE			***Date***	13 Jan 2023 00:48:39
Date Stamp	12 Jan 2023 17:58:14			***File Name***			
Frequency (MHz)	150.91	***Nucleus***	13C	***Number of Transients***	4000	***Origin***	ECA 600
Original Points Count	32768	***Owner***	CKP	***Points Count***	32768	***Pulse Sequence***	single_pulse_dec
Receiver Gain	58.00	***Solvent***	CHLOROFORM-d			***Spectrum Offset (Hz)***	15065.9502
Sweep Width (Hz)	47348.49						

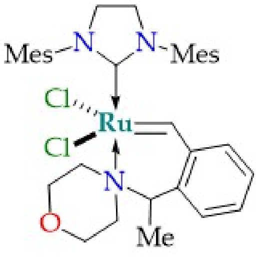

4d

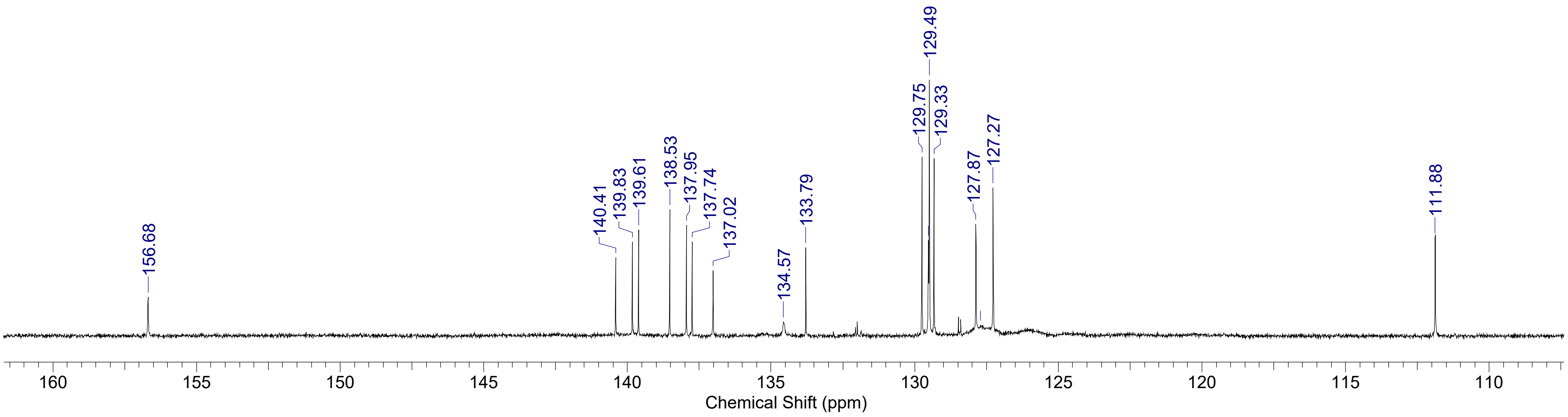

^{13}C spectrum of compound **4d**

Acquisition Time (sec)	0.6921	***Comment***	single pulse decoupled gated NOE			***Date***	13 Jan 2023 00:48:39
Date Stamp	12 Jan 2023 17:58:14			***File Name***			
Frequency (MHz)	150.91	***Nucleus***	13C	***Number of Transients***	4000	***Origin***	ECA 600
Original Points Count	32768	***Owner***	CKP	***Points Count***	32768	***Pulse Sequence***	single_pulse_dec
Receiver Gain	58.00	***Solvent***	CHLOROFORM-d			***Spectrum Offset (Hz)***	15065.9502
Sweep Width (Hz)	47348.49						

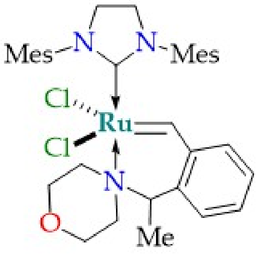

4d

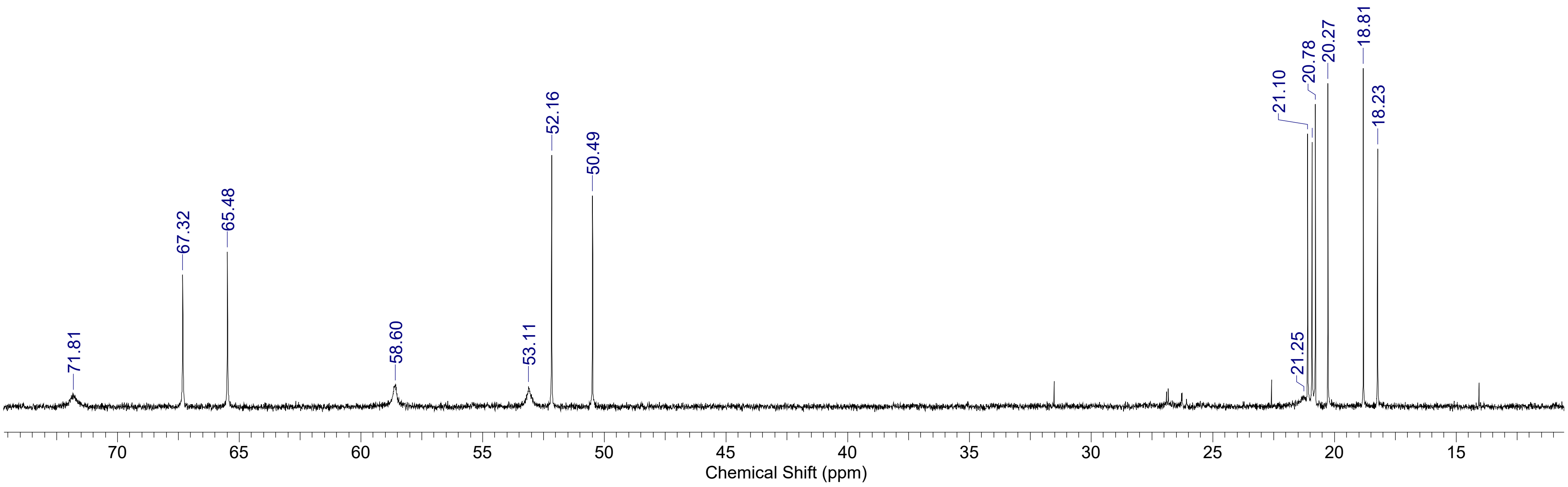

IR spectrum of compound **4d**

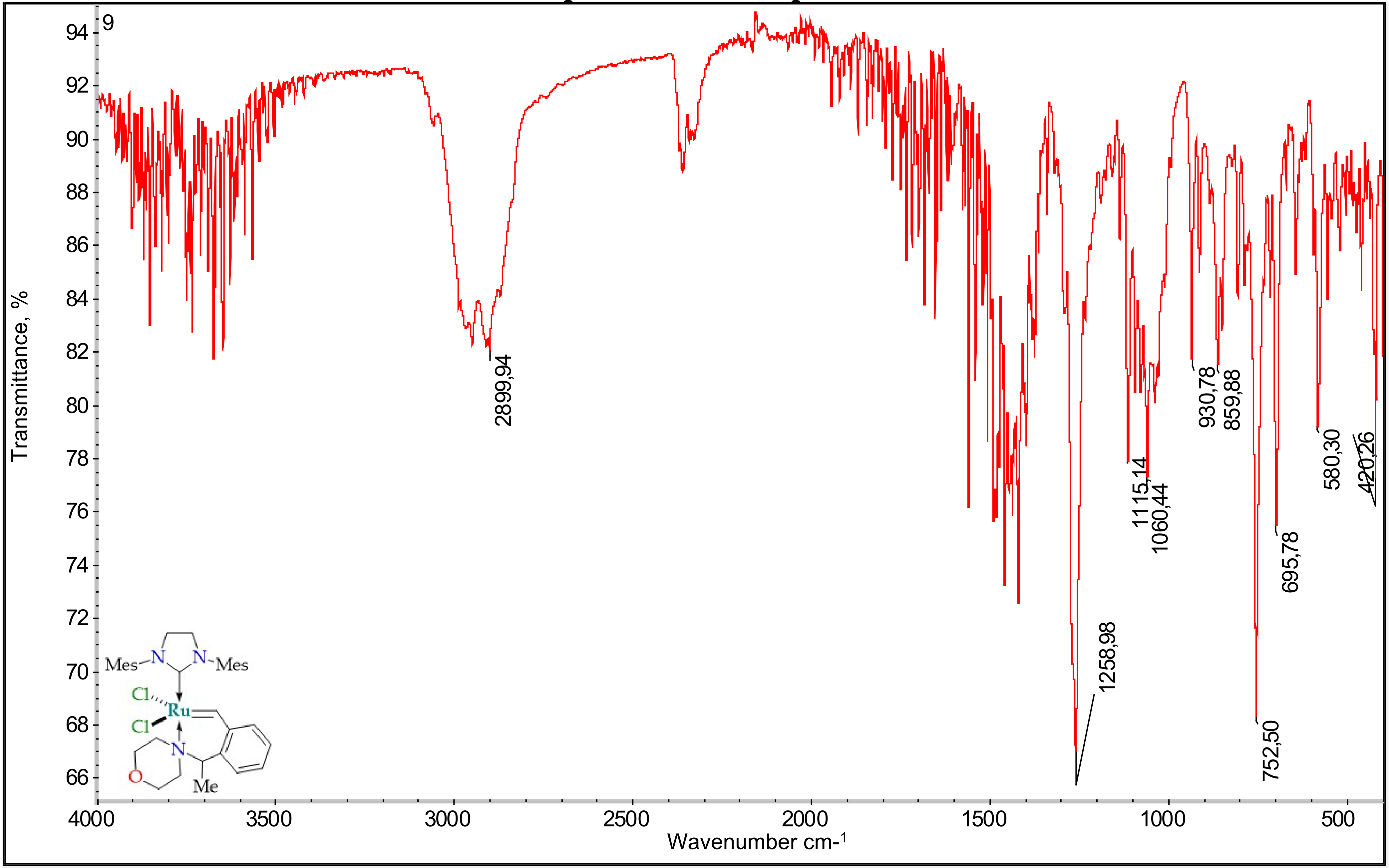

^{1}H spectrum of compound **5a**

Acquisition Time (sec)	1.7459	***Comment***	single_pulse	***Date***	23 Jan 2023 17:17:07		
Date Stamp	23 Jan 2023 16:14:45			***File Name***			
Frequency (MHz)	600.17	***Nucleus***	1H	***Number of Transients***	16	***Origin***	ECA 600
Original Points Count	32768	***Owner***	CKP	***Points Count***	32768	***Pulse Sequence***	single_pulse.ex2
Receiver Gain	38.00	***Solvent***	CHLOROFORM-d			***Spectrum Offset (Hz)***	6614.0498
Sweep Width (Hz)	18768.77						

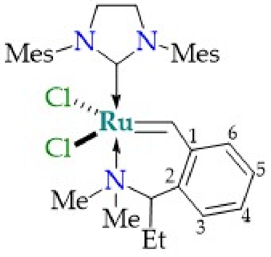

5a

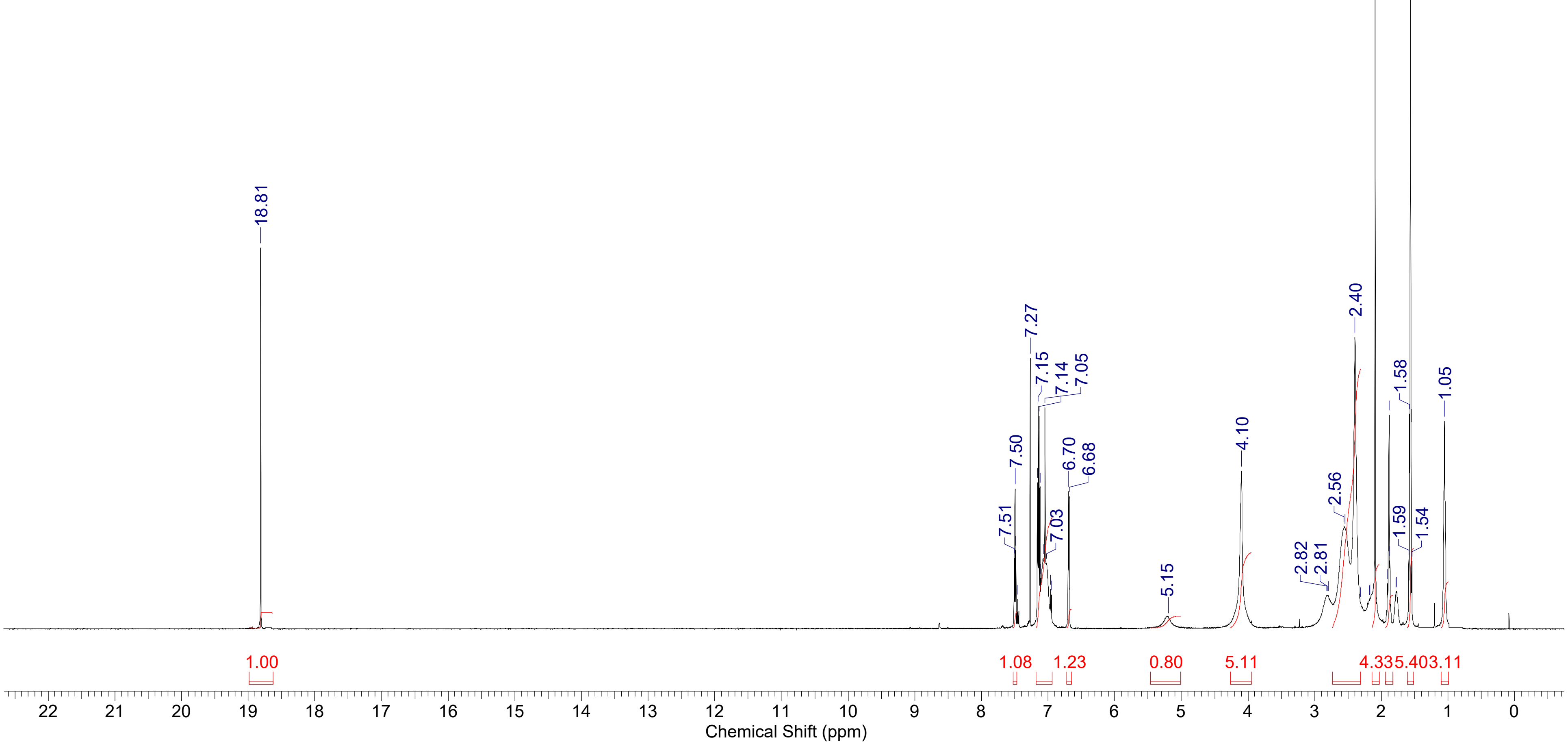

^{1}H spectrum of compound **5a**

Acquisition Time (sec)	1.7459	***Comment***	single_pulse	***Date***	23 Jan 2023 17:17:07		
Date Stamp	23 Jan 2023 16:14:45			***File Name***			
Frequency (MHz)	600.17	***Nucleus***	1H	***Number of Transients***	16	***Origin***	ECA 600
Original Points Count	32768	***Owner***	CKP	***Points Count***	32768	***Pulse Sequence***	single_pulse.ex2
Receiver Gain	38.00	***Solvent***	CHLOROFORM-d			***Spectrum Offset (Hz)***	6614.0498
Sweep Width (Hz)	18768.77						

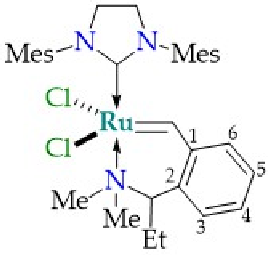

5a

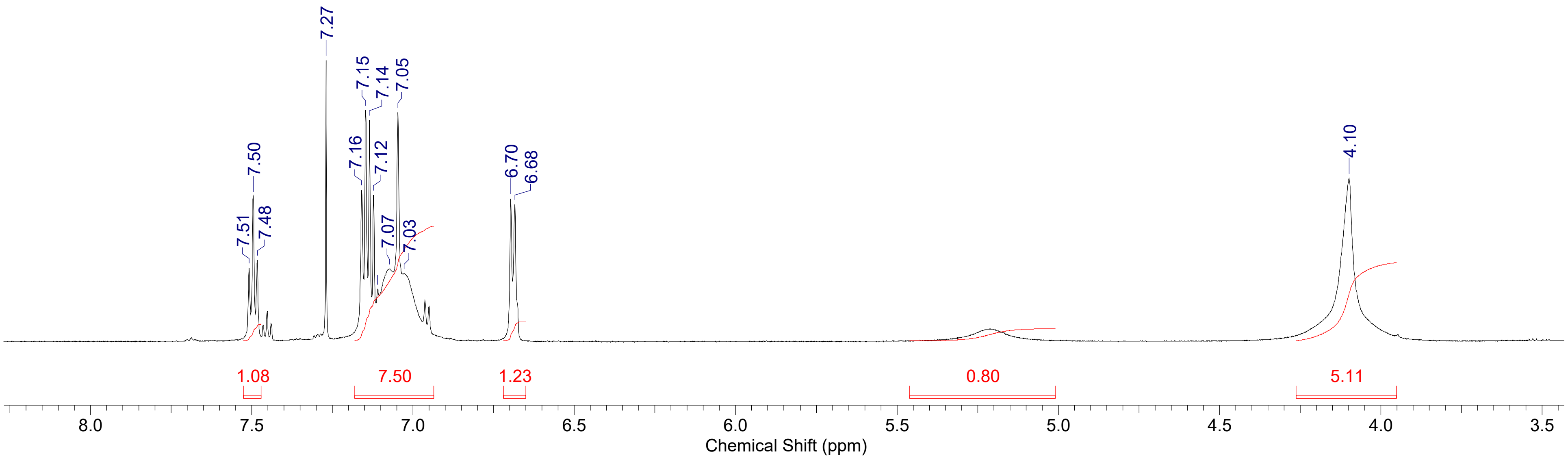

^{1}H spectrum of compound **5a**

Acquisition Time (sec)	1.7459	***Comment***	single_pulse	***Date***	23 Jan 2023 17:17:07		
Date Stamp	23 Jan 2023 16:14:45			***File Name***			
Frequency (MHz)	600.17	***Nucleus***	1H	***Number of Transients***	16	***Origin***	ECA 600
Original Points Count	32768	***Owner***	CKP	***Points Count***	32768	***Pulse Sequence***	single_pulse.ex2
Receiver Gain	38.00	***Solvent***	CHLOROFORM-d			***Spectrum Offset (Hz)***	6614.0498
Sweep Width (Hz)	18768.77						

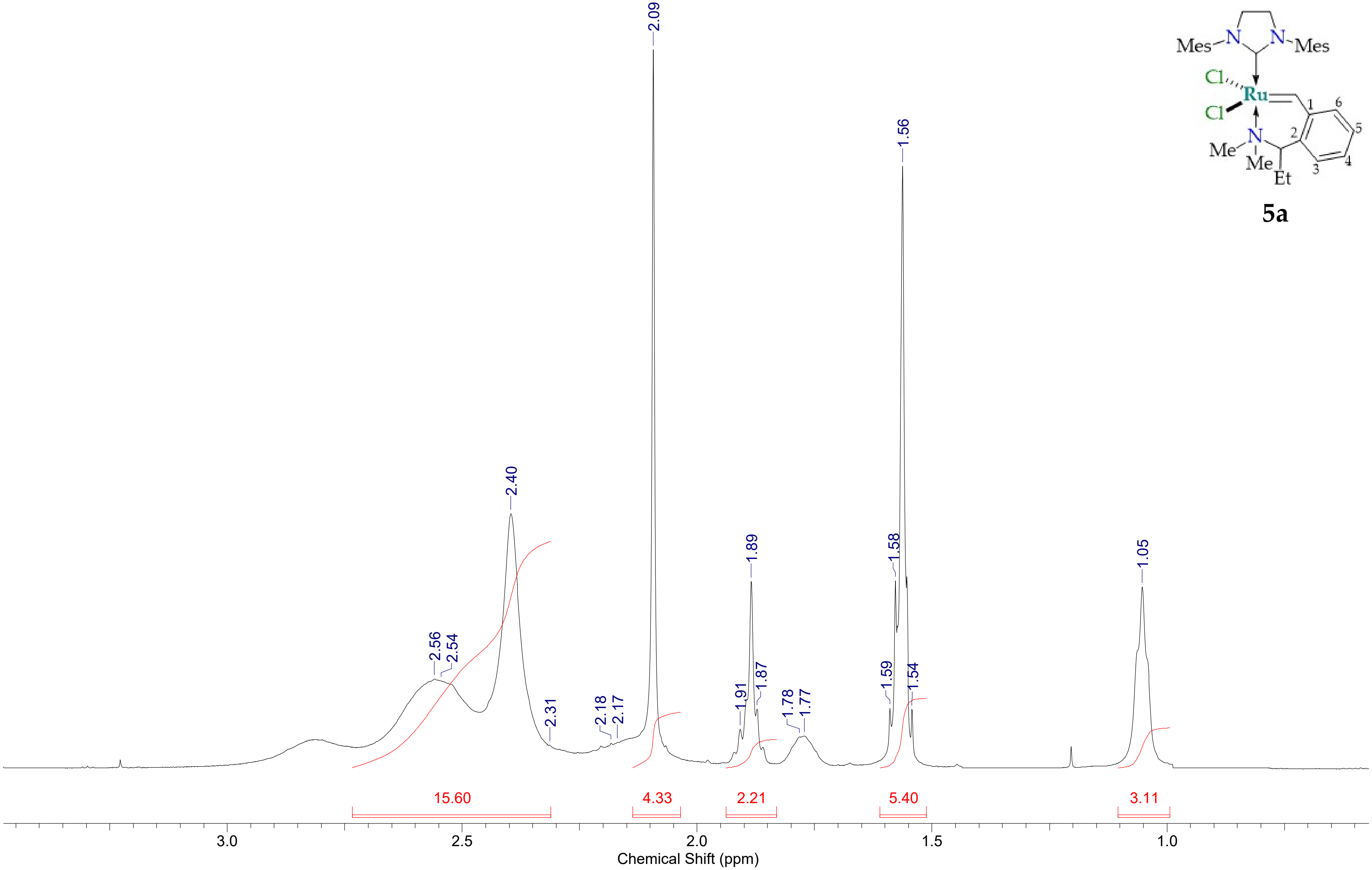

^{13}C spectrum of compound **5a**

Acquisition Time (sec)	0.9647	Comment	single pulse decoupled gated NOE	Date	24 Jan 2023 00:44:55
Date Stamp	23 Jan 2023 17:54:48	File Name			
Frequency (MHz)	150.91	Nucleus	13C	Number of Transients	4000
Origin	ECA 600				
Original Points Count	65536	Owner	CKP	Points Count	65536
Pulse Sequence	single_pulse_dec				
Receiver Gain	58.00	Solvent	CHLOROFORM-d	Spectrum Offset (Hz)	25655.2832
Sweep Width (Hz)	67934.78				

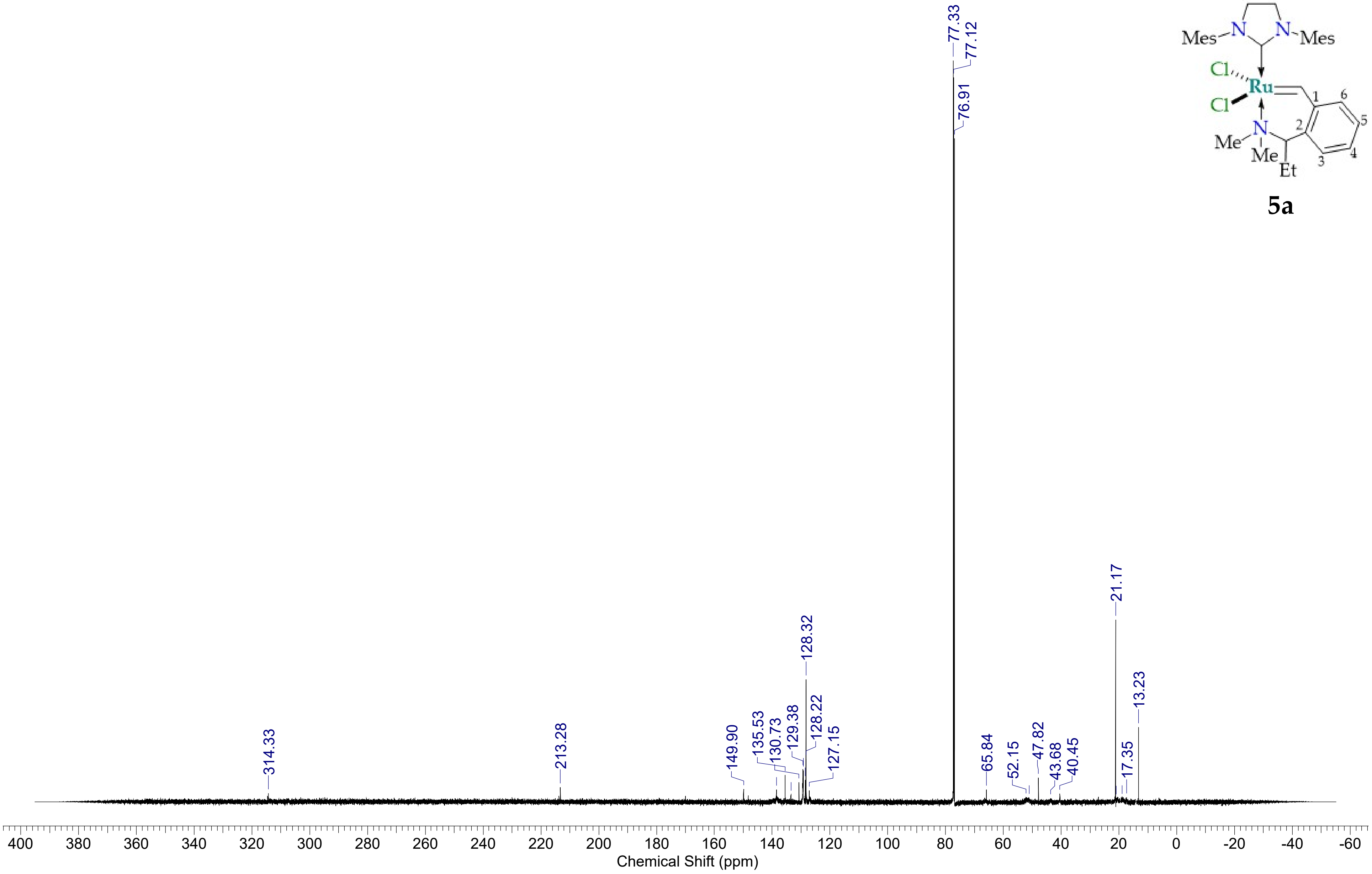

^{13}C spectrum of compound **5a**

Acquisition Time (sec)	0.9647	***Comment***	single pulse decoupled gated NOE			***Date***	24 Jan 2023 00:44:55
Date Stamp	23 Jan 2023 17:54:48			***File Name***			
Frequency (MHz)	150.91	***Nucleus***	13C	***Number of Transients***	4000	***Origin***	ECA 600
Original Points Count	65536	***Owner***	CKP	***Points Count***	65536	***Pulse Sequence***	single_pulse_dec
Receiver Gain	58.00	***Solvent***	CHLOROFORM-d			***Spectrum Offset (Hz)***	25655.2832
Sweep Width (Hz)	67934.78						

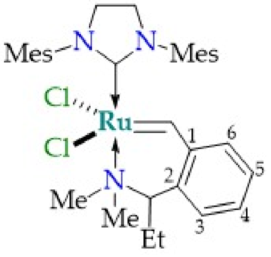

5a

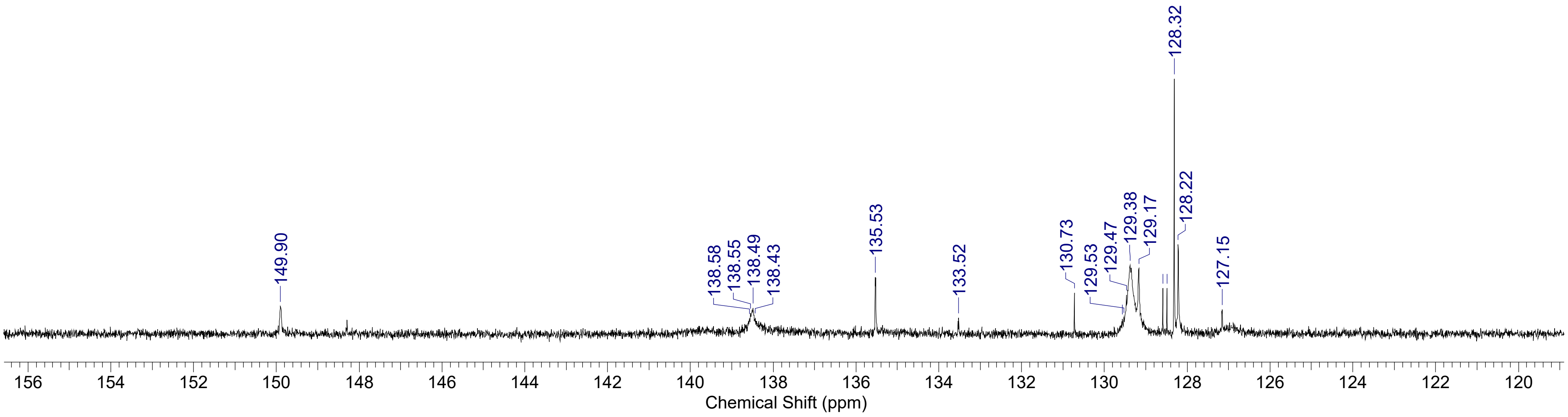

^{13}C spectrum of compound **5a**

Acquisition Time (sec)	0.9647	***Comment***	single pulse decoupled gated NOE			***Date***	24 Jan 2023 00:44:55
Date Stamp	23 Jan 2023 17:54:48			***File Name***			
Frequency (MHz)	150.91	***Nucleus***	13C	***Number of Transients***	4000	***Origin***	ECA 600
Original Points Count	65536	***Owner***	CKP	***Points Count***	65536	***Pulse Sequence***	single_pulse_dec
Receiver Gain	58.00	***Solvent***	CHLOROFORM-d			***Spectrum Offset (Hz)***	25655.2832
Sweep Width (Hz)	67934.78						

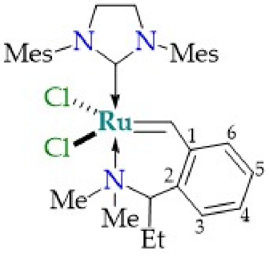

5a

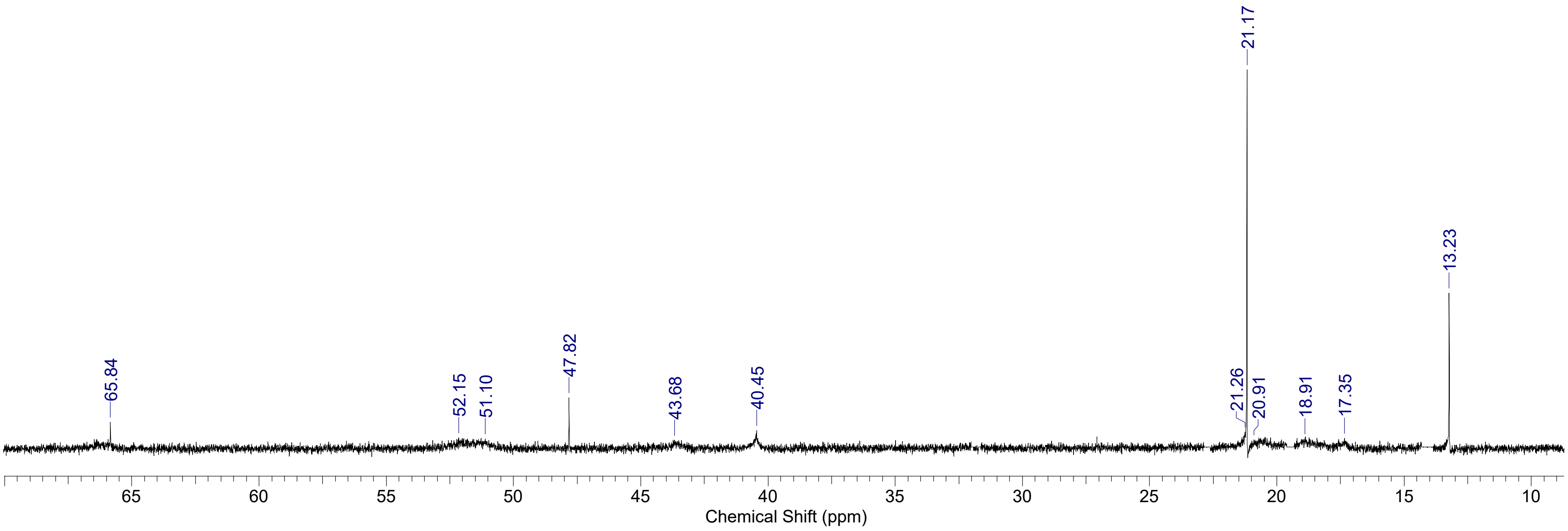

IR spectrum of compound **5a**

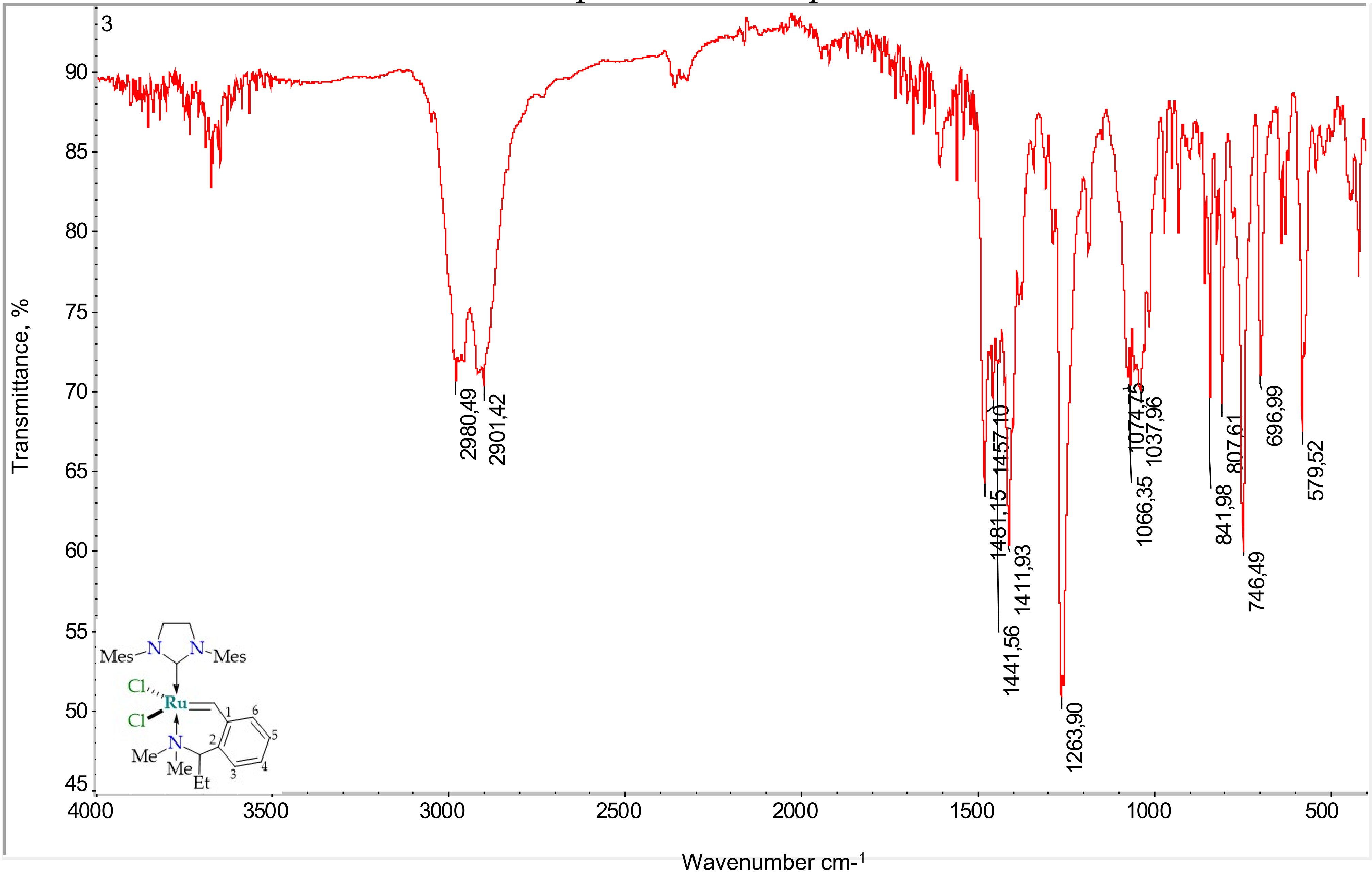

^{1}H spectrum of compound **5b**

Acquisition Time (sec)	1.7459	***Comment***	single_pulse	***Date***	23 Jan 2023 11:57:29		
Date Stamp	23 Jan 2023 10:55:06			***File Name***			
Frequency (MHz)	600.17	***Nucleus***	1H	***Number of Transients***	16	***Origin***	ECA 600
Original Points Count	32768	***Owner***	CKP	***Points Count***	32768	***Pulse Sequence***	single_pulse.ex2
Receiver Gain	34.00	***Solvent***	CHLOROFORM-d			***Spectrum Offset (Hz)***	6614.6226
Sweep Width (Hz)	18768.77						

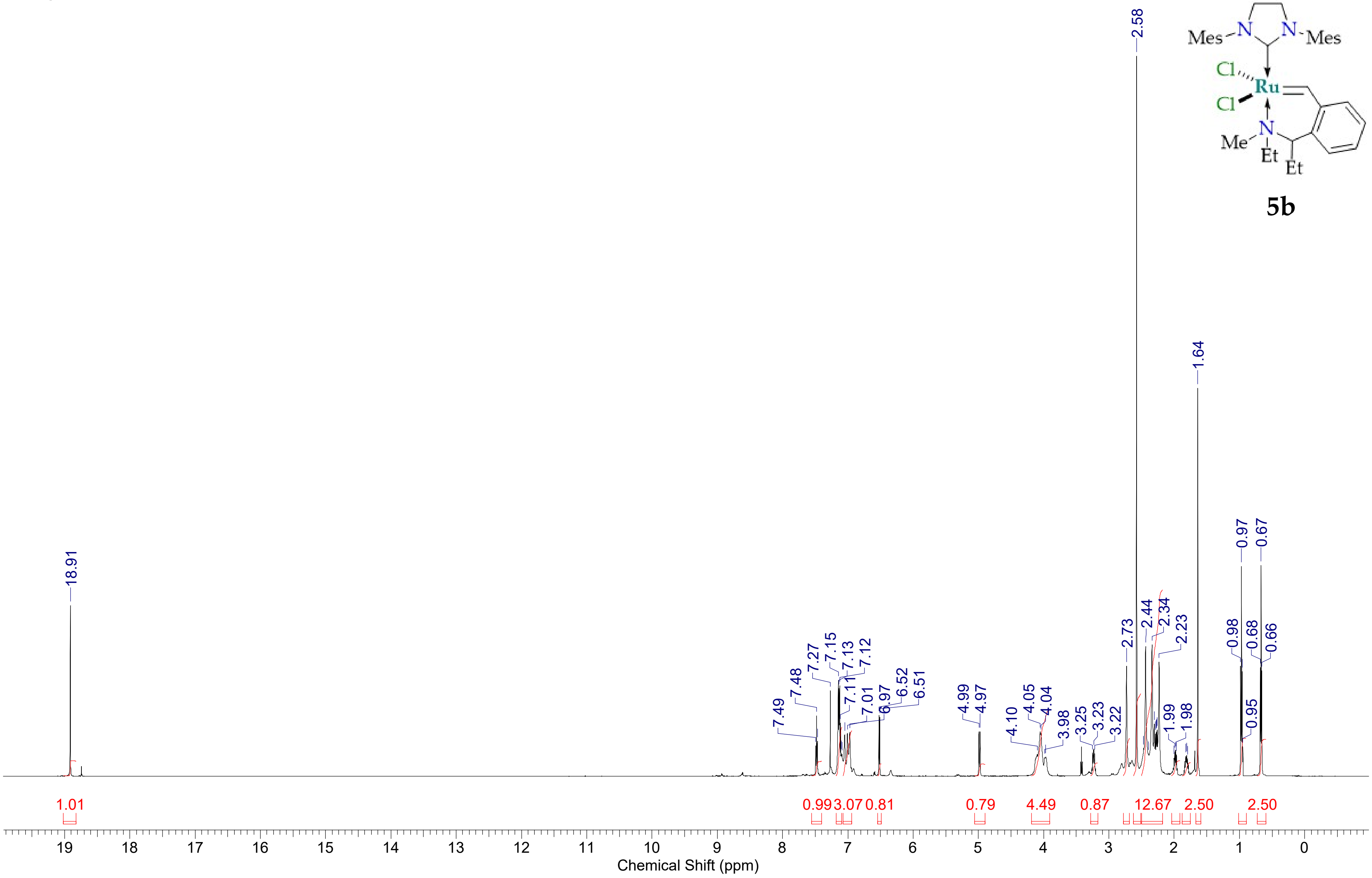

5b

^{1}H spectrum of compound **5b**

Acquisition Time (sec)	1.7459	***Comment***	single_pulse	***Date***	23 Jan 2023 11:57:29		
Date Stamp	23 Jan 2023 10:55:06			***File Name***			
Frequency (MHz)	600.17	***Nucleus***	1H	***Number of Transients***	16	***Origin***	ECA 600
Original Points Count	32768	***Owner***	CKP	***Points Count***	32768	***Pulse Sequence***	single_pulse.ex2
Receiver Gain	34.00	***Solvent***	CHLOROFORM-d			***Spectrum Offset (Hz)***	6614.6226
Sweep Width (Hz)	18768.77						

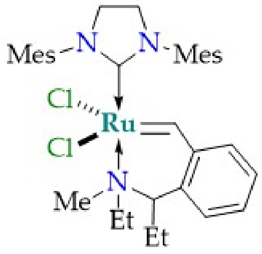

5b

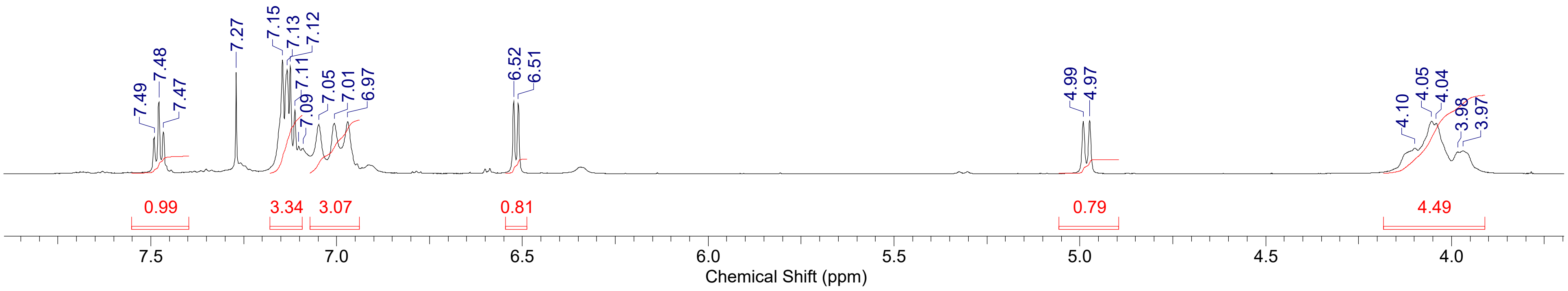

^{1}H spectrum of compound **5b**

Acquisition Time (sec)	1.7459	***Comment***	single_pulse	***Date***	23 Jan 2023 11:57:29		
Date Stamp	23 Jan 2023 10:55:06			***File Name***			
Frequency (MHz)	600.17	***Nucleus***	1H	***Number of Transients***	16	***Origin***	ECA 600
Original Points Count	32768	***Owner***	CKP	***Points Count***	32768	***Pulse Sequence***	single_pulse.ex2
Receiver Gain	34.00	***Solvent***	CHLOROFORM-d			***Spectrum Offset (Hz)***	6614.6226
Sweep Width (Hz)	18768.77						

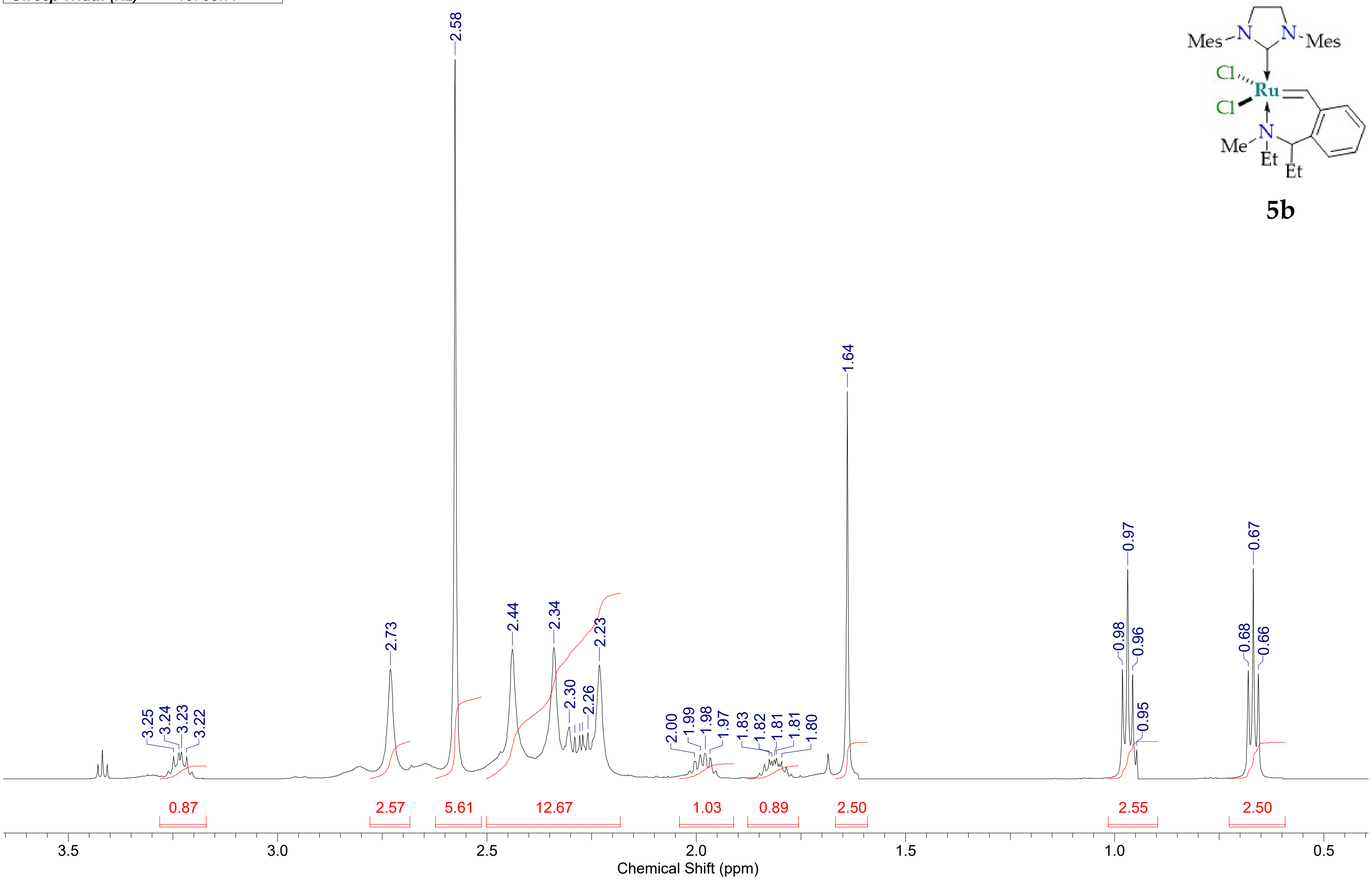

5b

^{13}C spectrum of compound **5b**

Acquisition Time (sec)	0.9647	Comment	single pulse decoupled gated NOE	Date	23 Jan 2023 13:37:35
Date Stamp	23 Jan 2023 12:35:13	File Name			
Frequency (MHz)	150.91	Nucleus	13C	Number of Transients	4000	Origin	ECA 600
Original Points Count	65536	Owner	CKP	Points Count	65536	Pulse Sequence	single_pulse_dec
Receiver Gain	56.00	Solvent	CHLOROFORM-d	Spectrum Offset (Hz)	25629.7129
Sweep Width (Hz)	67934.78				

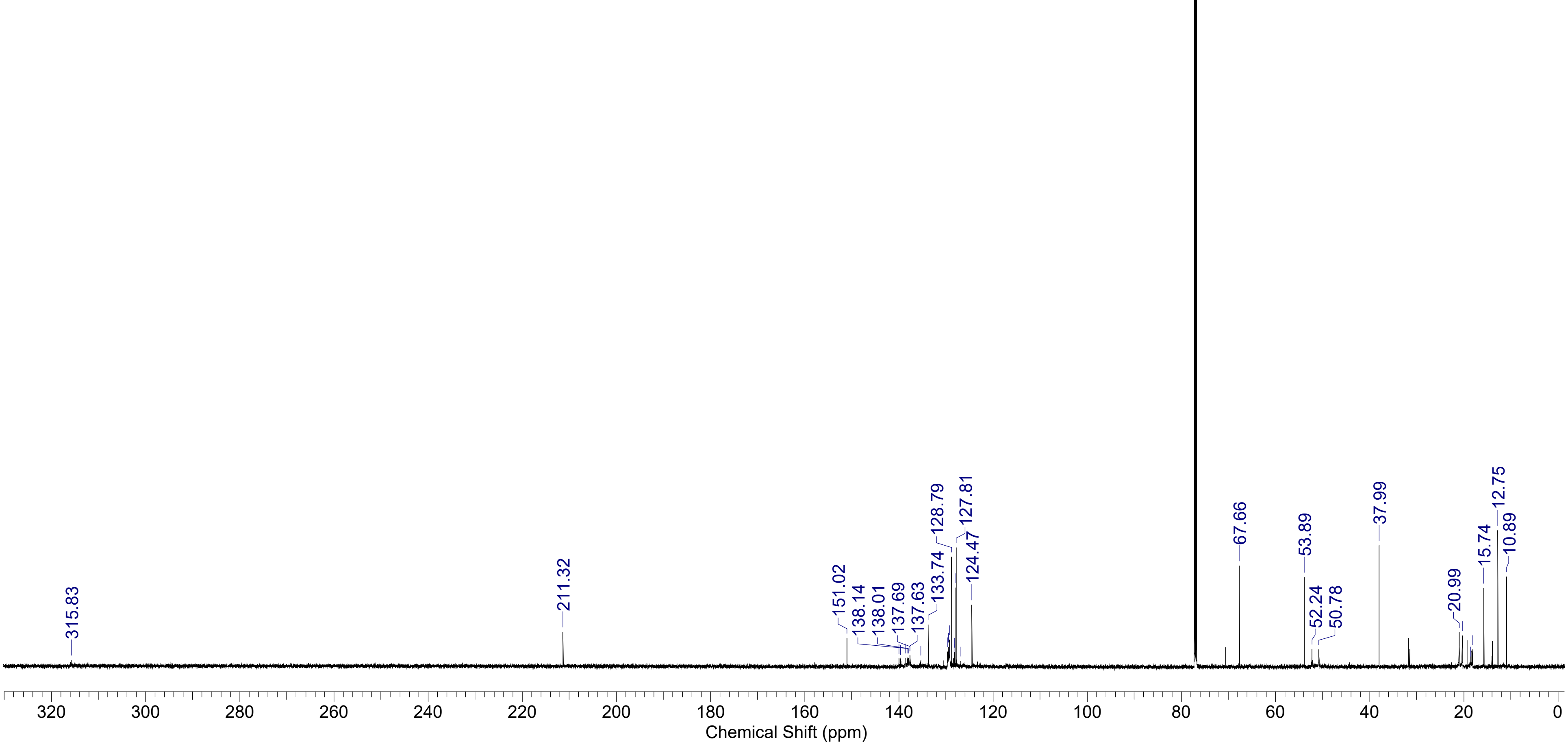

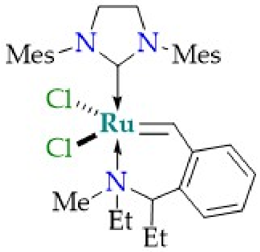

5b

^{13}C spectrum of compound **5b**

Acquisition Time (sec)	0.9647	***Comment***	single pulse decoupled gated NOE			***Date***	23 Jan 2023 13:37:35
Date Stamp	23 Jan 2023 12:35:13			***File Name***			
Frequency (MHz)	150.91	***Nucleus***	13C	***Number of Transients***	4000	***Origin***	ECA 600
Original Points Count	65536	***Owner***	CKP	***Points Count***	65536	***Pulse Sequence***	single_pulse_dec
Receiver Gain	56.00	***Solvent***	CHLOROFORM-d			***Spectrum Offset (Hz)***	25629.7129
Sweep Width (Hz)	67934.78						

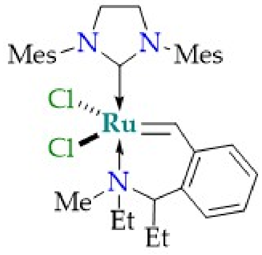

5b

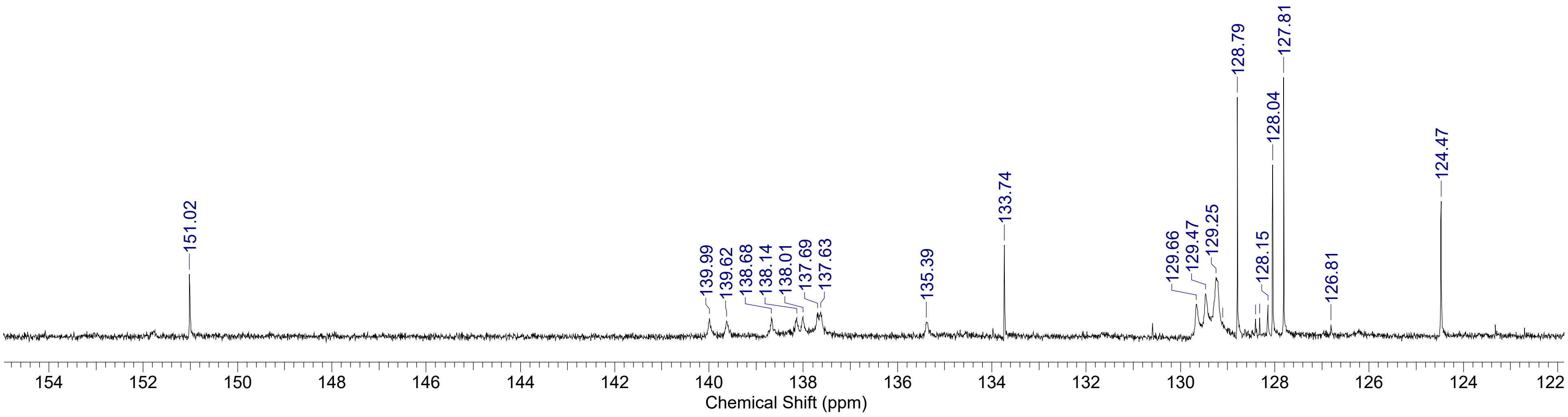

^{13}C spectrum of compound **5b**

Acquisition Time (sec)	0.9647	***Comment***	single pulse decoupled gated NOE			***Date***	23 Jan 2023 13:37:35
Date Stamp	23 Jan 2023 12:35:13			***File Name***			
Frequency (MHz)	150.91	***Nucleus***	13C	***Number of Transients***	4000	***Origin***	ECA 600
Original Points Count	65536	***Owner***	CKP	***Points Count***	65536	***Pulse Sequence***	single_pulse_dec
Receiver Gain	56.00	***Solvent***	CHLOROFORM-d			***Spectrum Offset (Hz)***	25629.7129
Sweep Width (Hz)	67934.78						

FZ1491-2.JDF

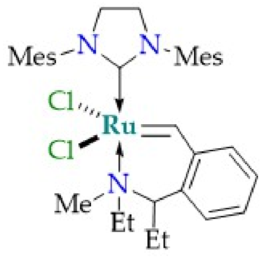

5b

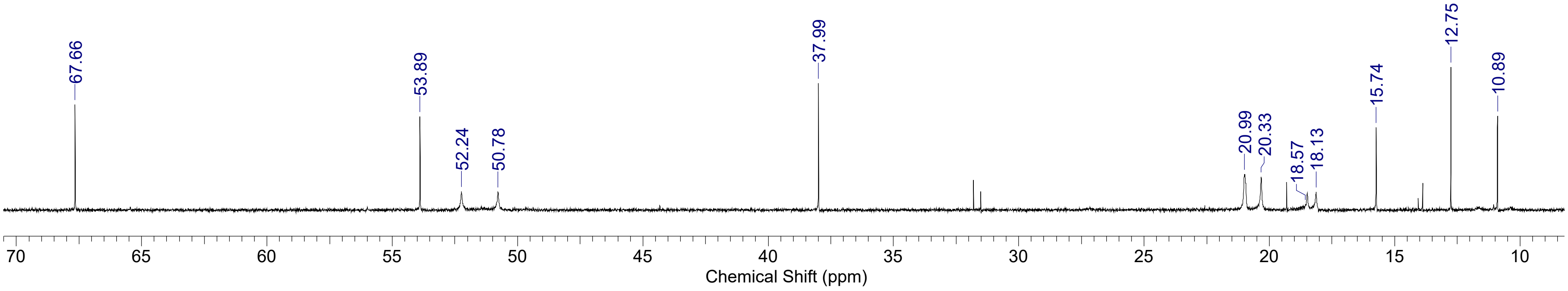

IR spectrum of compound **5b**

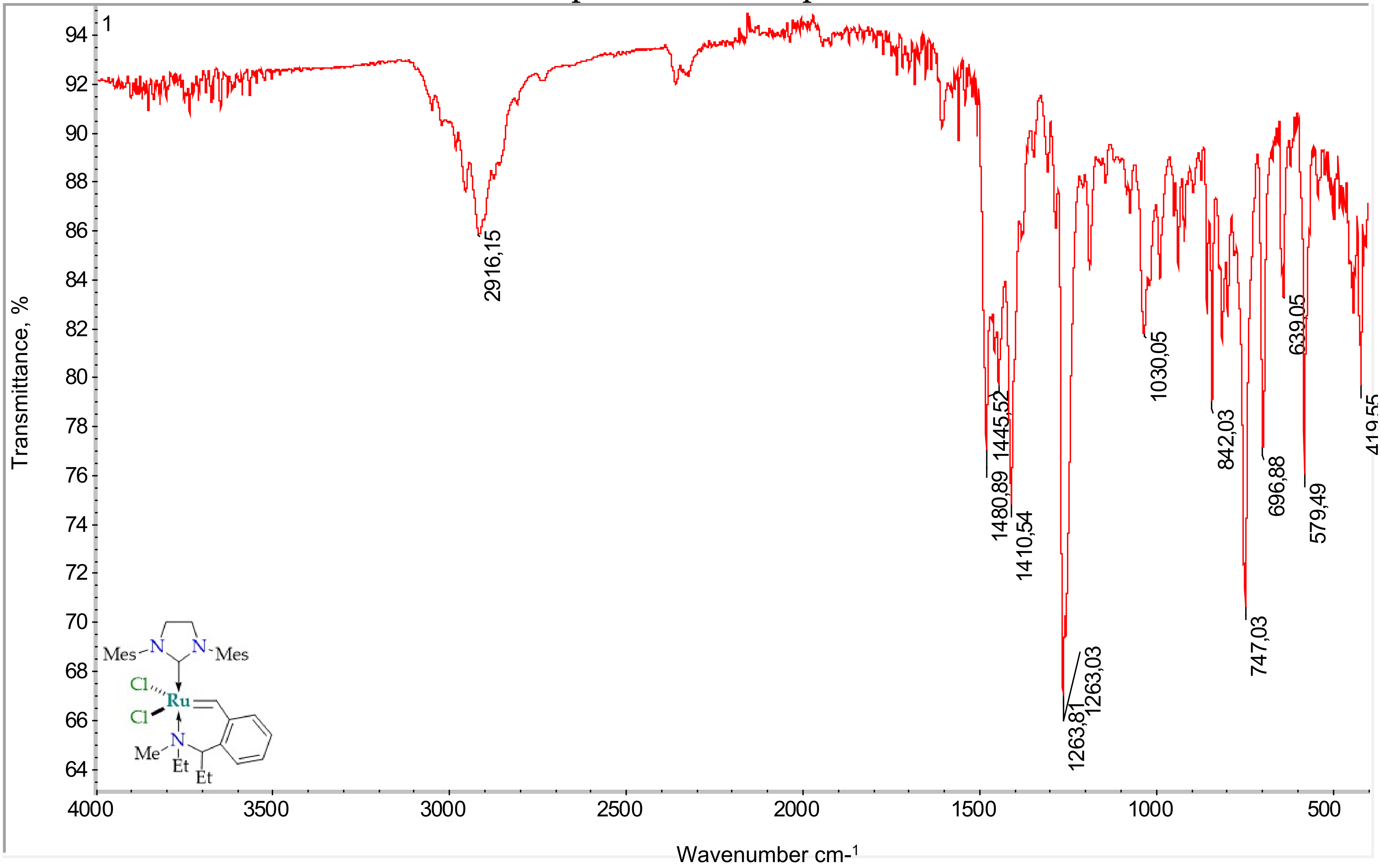

^{1}H spectrum of compound **5c**

Acquisition Time (sec)	1.9818	***Comment***	single_pulse	***Date***			
Date Stamp							
Frequency (MHz)	600.17	***Nucleus***	1H	***Number of Transients***	8	***Origin***	ECA 600
Original Points Count	32768	***Owner***	CKP	***Points Count***	32768	***Pulse Sequence***	single_pulse.ex2
Receiver Gain	42.00	***Solvent***	CHLOROFORM-d	***Spectrum Offset (Hz)***	5364.2036	***Sweep Width (Hz)***	16534.39

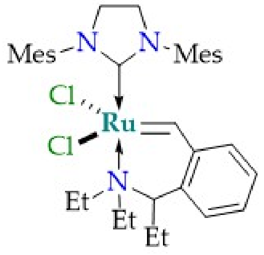

5c

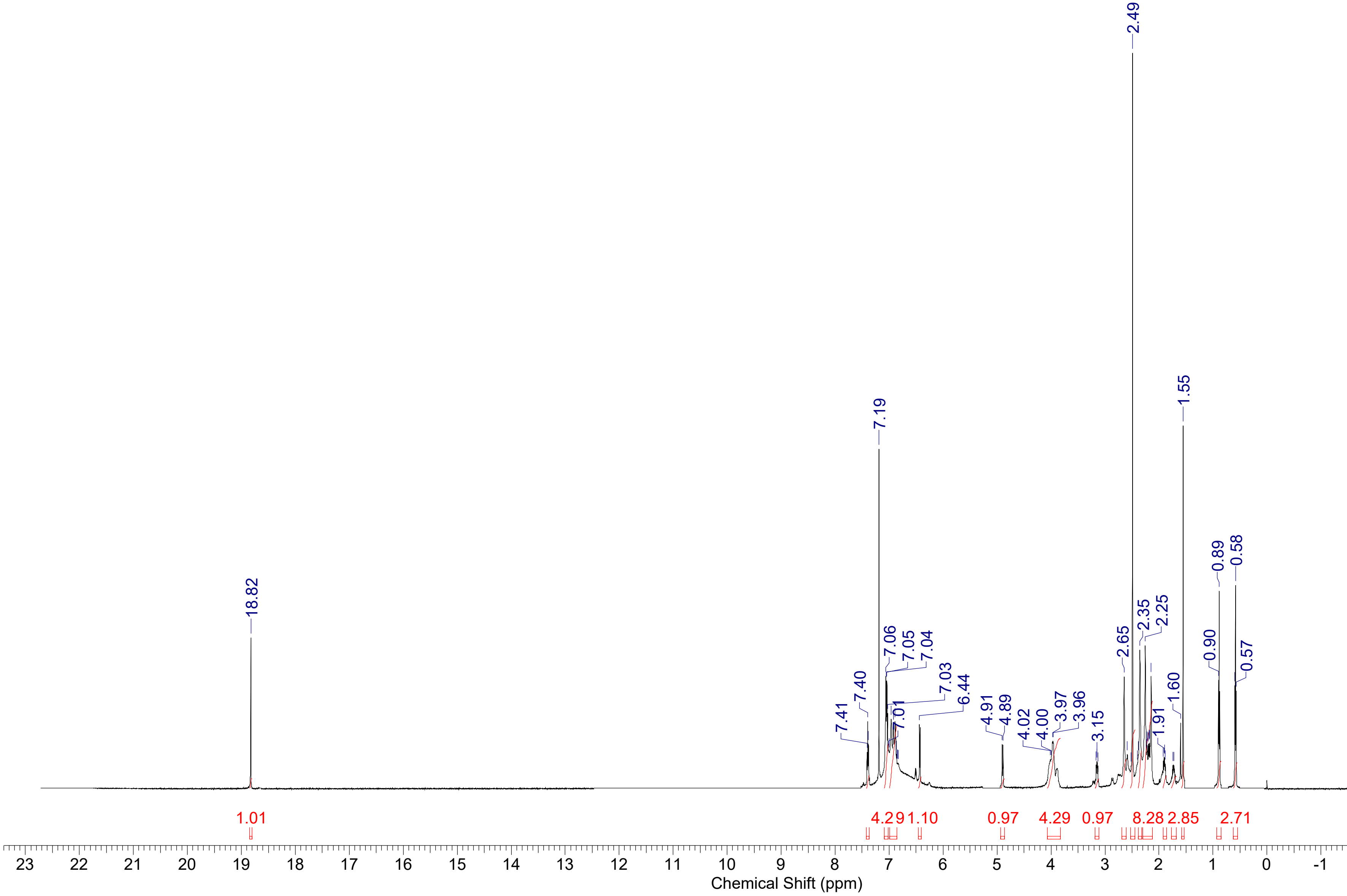

^{13}C spectrum of compound **5c**

Acquisition Time (sec)	0.6921	***Comment***	single pulse decoupled gated NOE			***Date***	
Date Stamp							
Frequency (MHz)	150.91	***Nucleus***	13C	***Number of Transients***	1000	***Origin***	ECA 600
Original Points Count	32768	***Owner***	CKP	***Points Count***	32768	***Pulse Sequence***	single_pulse_dec
Receiver Gain	54.00	***Solvent***	CHLOROFORM-d	***Spectrum Offset (Hz)***	15091.3428	***Sweep Width (Hz)***	47348.49

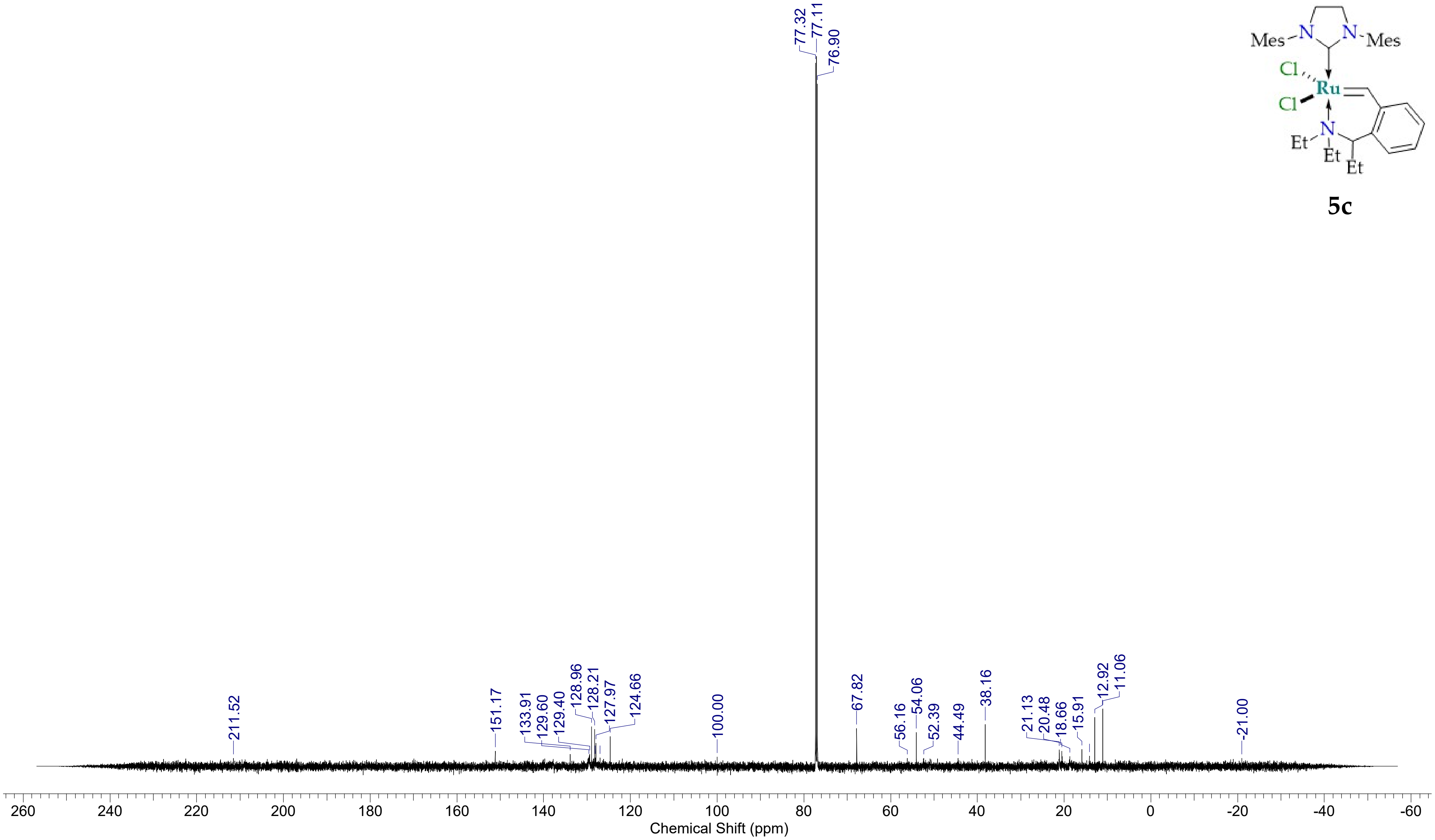

5c

^{1}H spectrum of compound **5d**

Acquisition Time (sec)	1.9818	*Comment*	single_pulse	*Date*	02 Nov 2022 13:18:46		
Date Stamp	02 Nov 2022 12:18:25			*File Name*			
Frequency (MHz)	600.17	*Nucleus*	1H	*Number of Transients*	8	*Origin*	ECA 600
Original Points Count	32768	*Owner*	CKP	*Points Count*	32768	*Pulse Sequence*	single_pulse.ex2
Receiver Gain	28.00	*Solvent*	CHLOROFORM-d			*Spectrum Offset (Hz)*	5498.8667
Sweep Width (Hz)	16534.39						

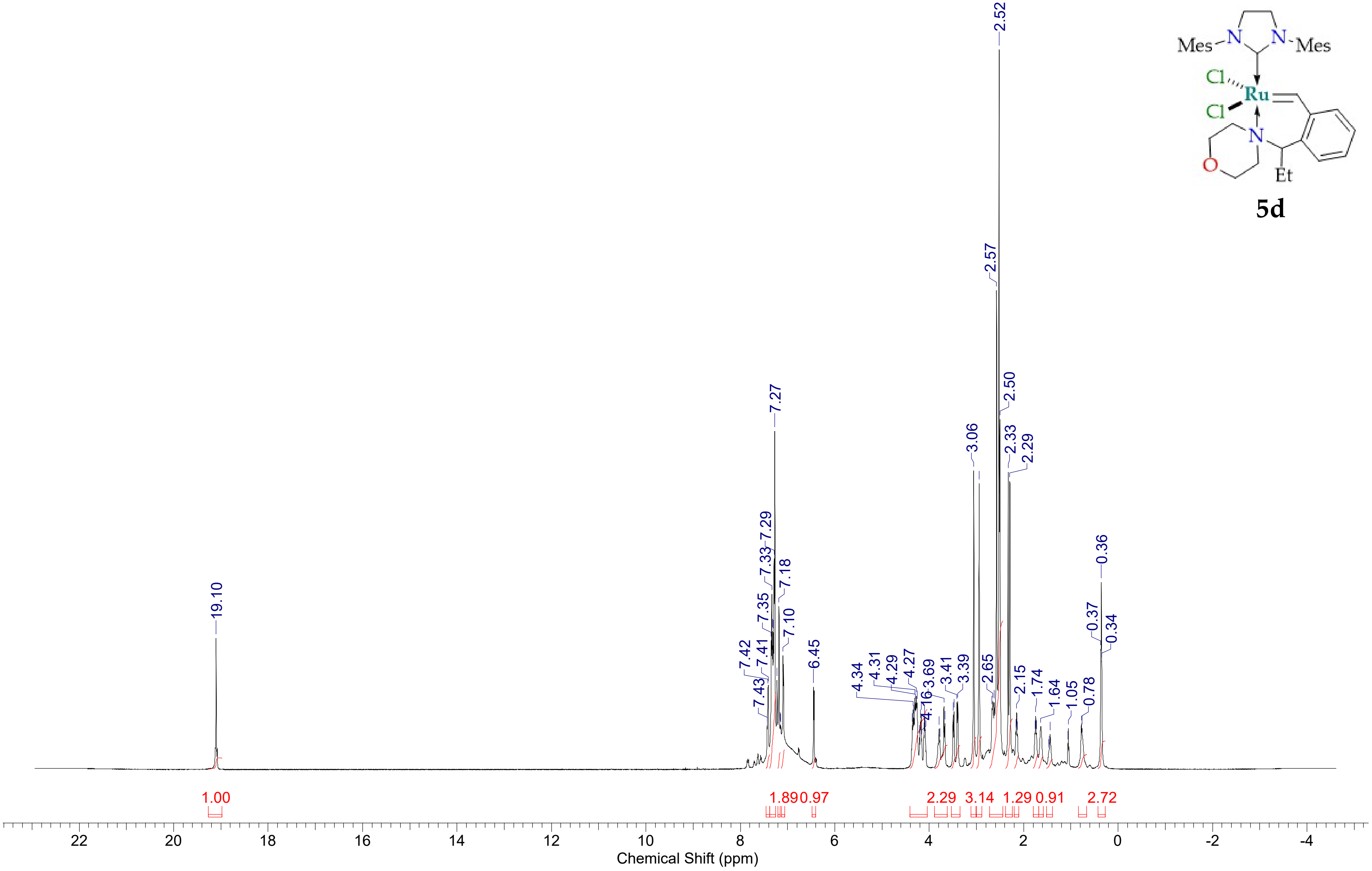

5d

^{1}H spectrum of compound **5d**

Acquisition Time (sec)	1.9818	***Comment***	single_pulse	***Date***	02 Nov 2022 13:18:46		
Date Stamp	02 Nov 2022 12:18:25			***File Name***			
Frequency (MHz)	600.17	***Nucleus***	1H	***Number of Transients***	8	***Origin***	ECA 600
Original Points Count	32768	***Owner***	CKP	***Points Count***	32768	***Pulse Sequence***	single_pulse.ex2
Receiver Gain	28.00	***Solvent***	CHLOROFORM-d			***Spectrum Offset (Hz)***	5498.8667
Sweep Width (Hz)	16534.39						

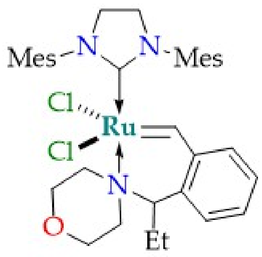

5d

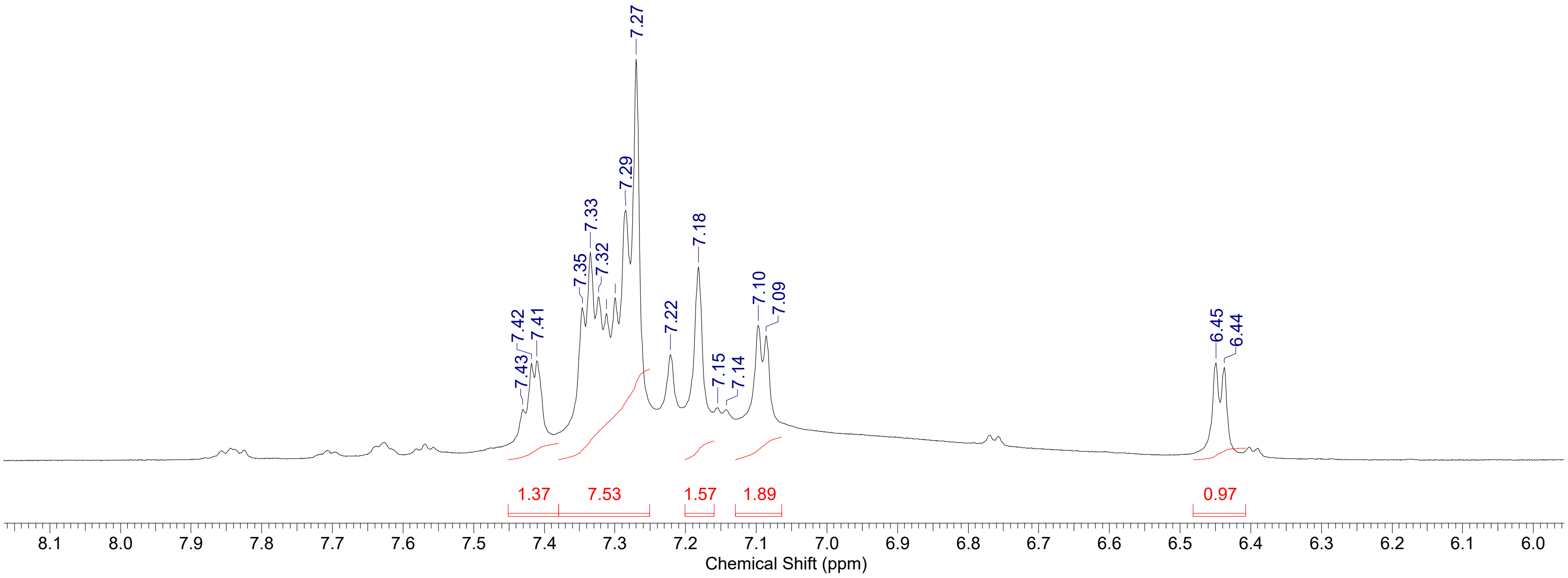

^{1}H spectrum of compound **5d**

Acquisition Time (sec)	1.9818	***Comment***	single_pulse	***Date***	02 Nov 2022 13:18:46		
Date Stamp	02 Nov 2022 12:18:25			***File Name***			
Frequency (MHz)	600.17	***Nucleus***	1H	***Number of Transients***	8	***Origin***	ECA 600
Original Points Count	32768	***Owner***	CKP	***Points Count***	32768	***Pulse Sequence***	single_pulse.ex2
Receiver Gain	28.00	***Solvent***	CHLOROFORM-d			***Spectrum Offset (Hz)***	5498.8667
Sweep Width (Hz)	16534.39						

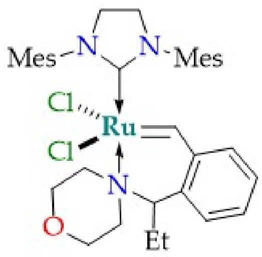

5d

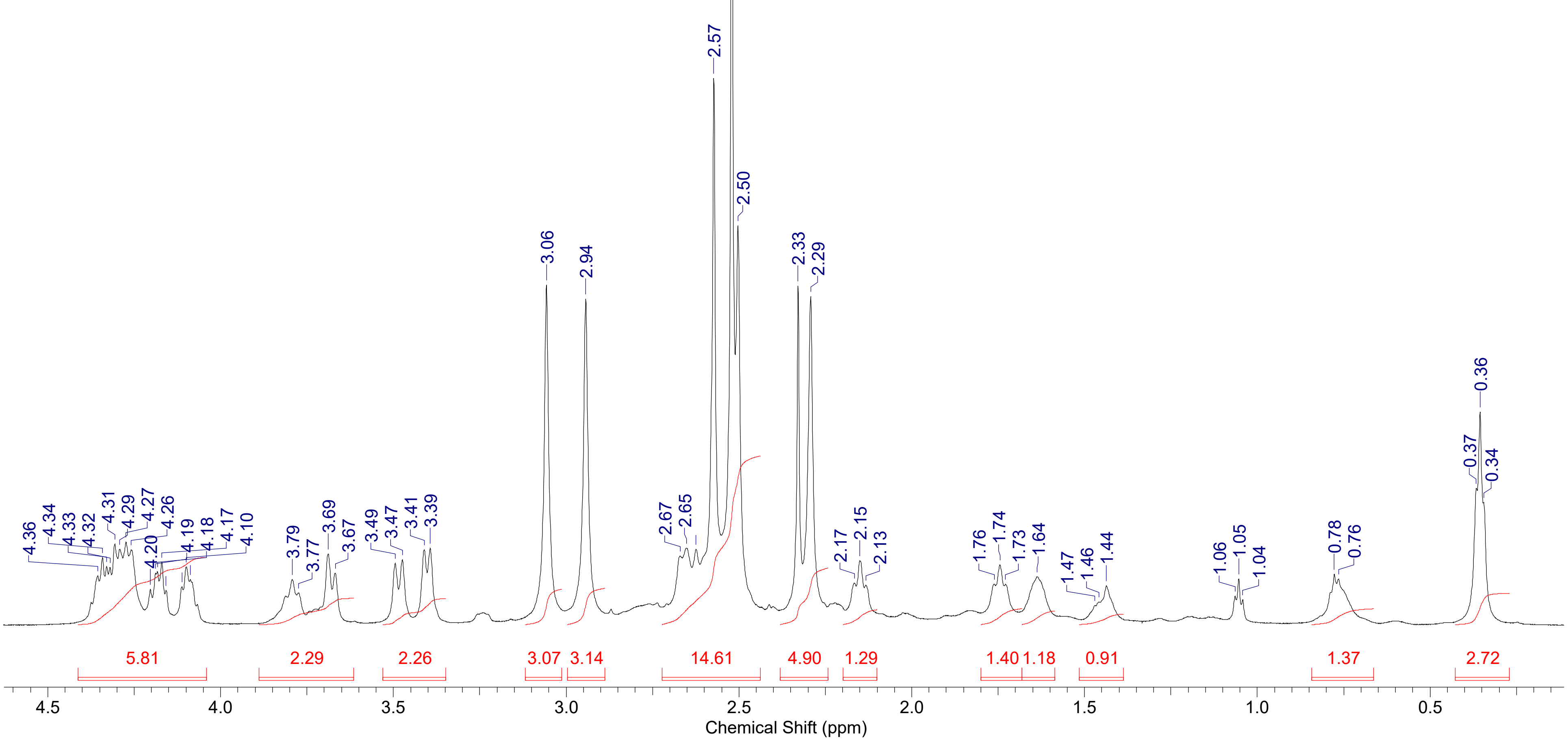

^{13}C spectrum of compound **5d**

Acquisition Time (sec)	1.7197	***Comment***	single pulse decoupled gated NOE			***Date***	19 Jan 2023 04:22:34
Date Stamp	19 Jan 2023 03:20:04			***File Name***			
Frequency (MHz)	150.91	***Nucleus***	13C	***Number of Transients***	4000	***Origin***	ECA 600
Original Points Count	131072	***Owner***	CKP	***Points Count***	131072	***Pulse Sequence***	single_pulse_dec
Receiver Gain	54.00	***Solvent***	CHLOROFORM-d			***Spectrum Offset (Hz)***	25630.4414
Sweep Width (Hz)	76219.52						

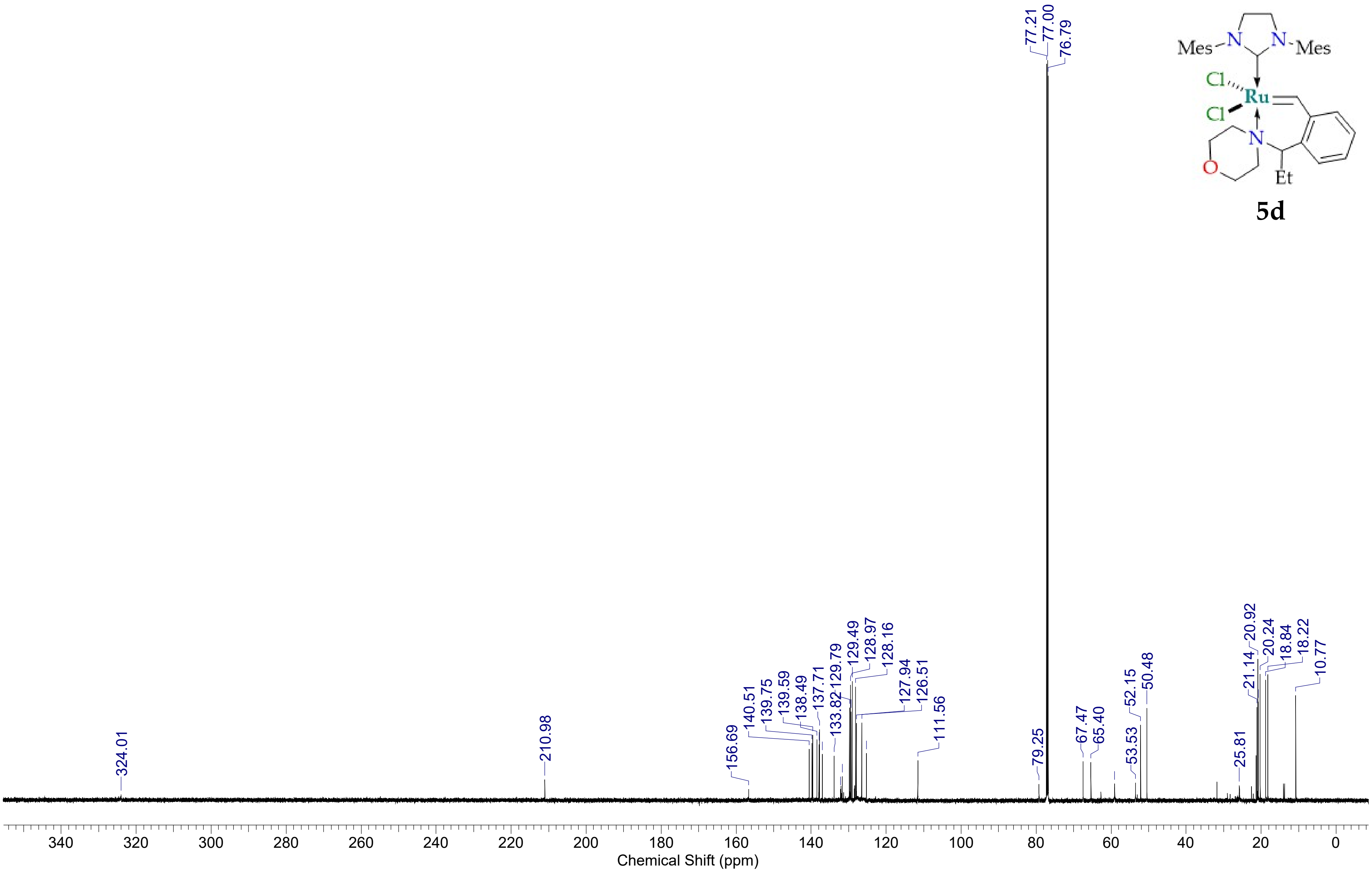

5d

^{13}C spectrum of compound **5d**

Acquisition Time (sec)	0.6921	***Comment***	single pulse decoupled gated NOE			***Date***	02 Nov 2022 13:40:49
Date Stamp	02 Nov 2022 12:40:28			***File Name***			
Frequency (MHz)	150.91	***Nucleus***	13C	***Number of Transients***	1000	***Origin***	ECA 600
Original Points Count	32768	***Owner***	CKP	***Points Count***	32768	***Pulse Sequence***	single_pulse_dec
Receiver Gain	58.00	***Solvent***	CHLOROFORM-d			***Spectrum Offset (Hz)***	15061.6152
Sweep Width (Hz)	47348.49						

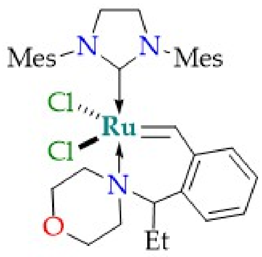

5d

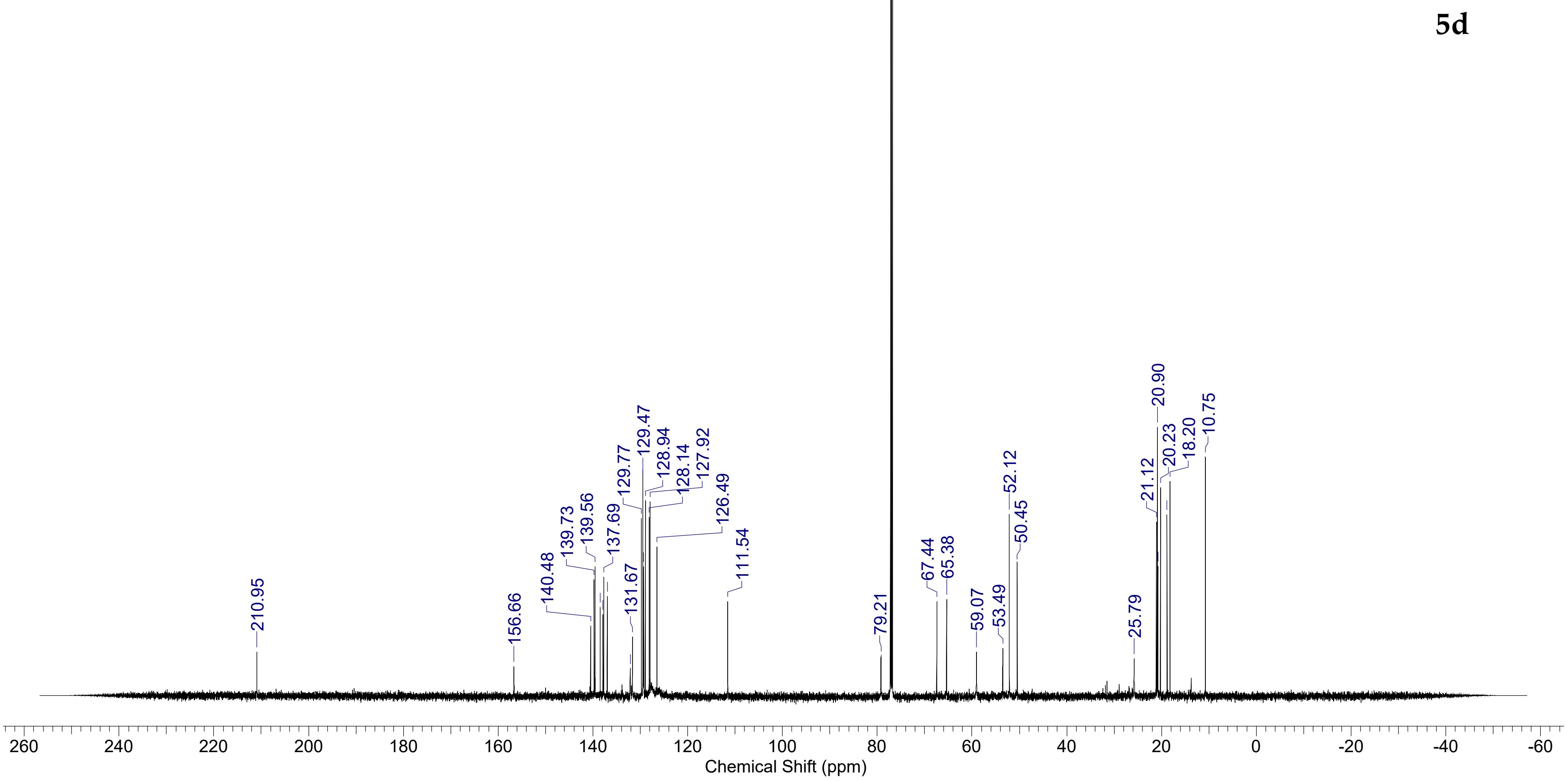

^{13}C spectrum of compound **5d**

Acquisition Time (sec)	0.6921	***Comment***	single pulse decoupled gated NOE			***Date***	02 Nov 2022 13:40:49
Date Stamp	02 Nov 2022 12:40:28			***File Name***			
Frequency (MHz)	150.91	***Nucleus***	13C	***Number of Transients***	1000	***Origin***	ECA 600
Original Points Count	32768	***Owner***	CKP	***Points Count***	32768	***Pulse Sequence***	single_pulse_dec
Receiver Gain	58.00	***Solvent***	CHLOROFORM-d			***Spectrum Offset (Hz)***	15061.6152
Sweep Width (Hz)	47348.49						

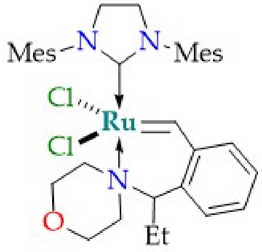

5d

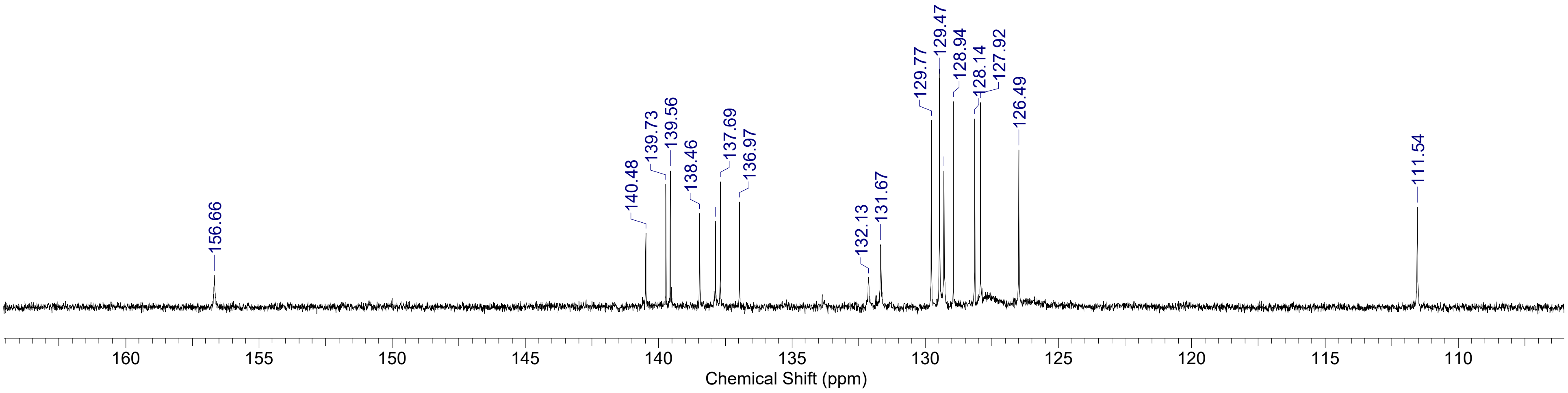

^{13}C spectrum of compound **5d**

Acquisition Time (sec)	0.6921	***Comment***	single pulse decoupled gated NOE			***Date***	02 Nov 2022 13:40:49
Date Stamp	02 Nov 2022 12:40:28			***File Name***			
Frequency (MHz)	150.91	***Nucleus***	13C	***Number of Transients***	1000	***Origin***	ECA 600
Original Points Count	32768	***Owner***	CKP	***Points Count***	32768	***Pulse Sequence***	single_pulse_dec
Receiver Gain	58.00	***Solvent***	CHLOROFORM-d			***Spectrum Offset (Hz)***	15061.6152
Sweep Width (Hz)	47348.49						

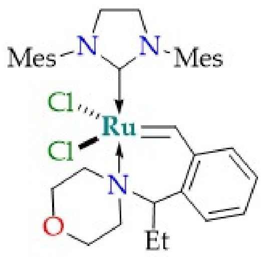

5d

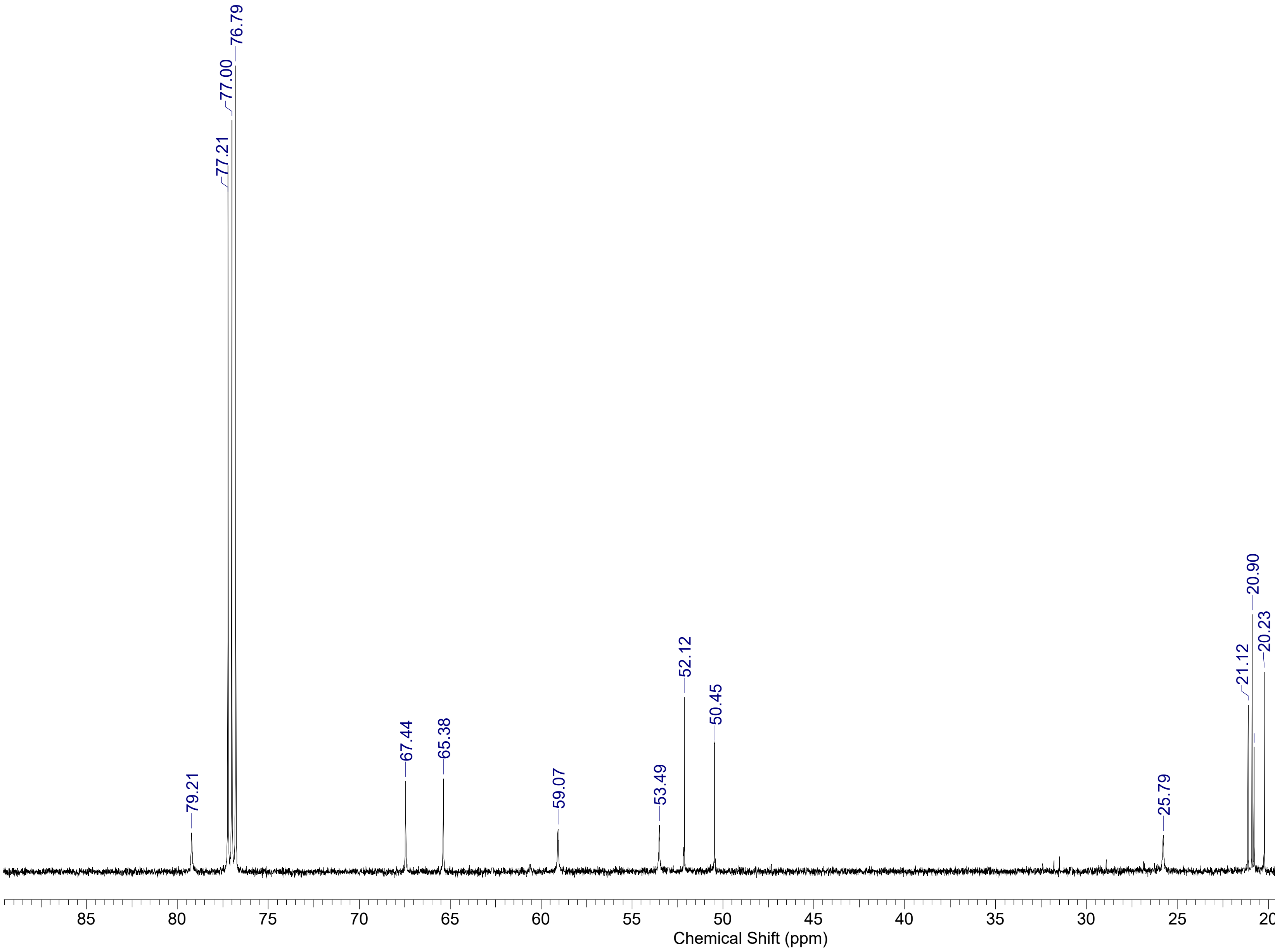

IR spectrum of compound **5d**

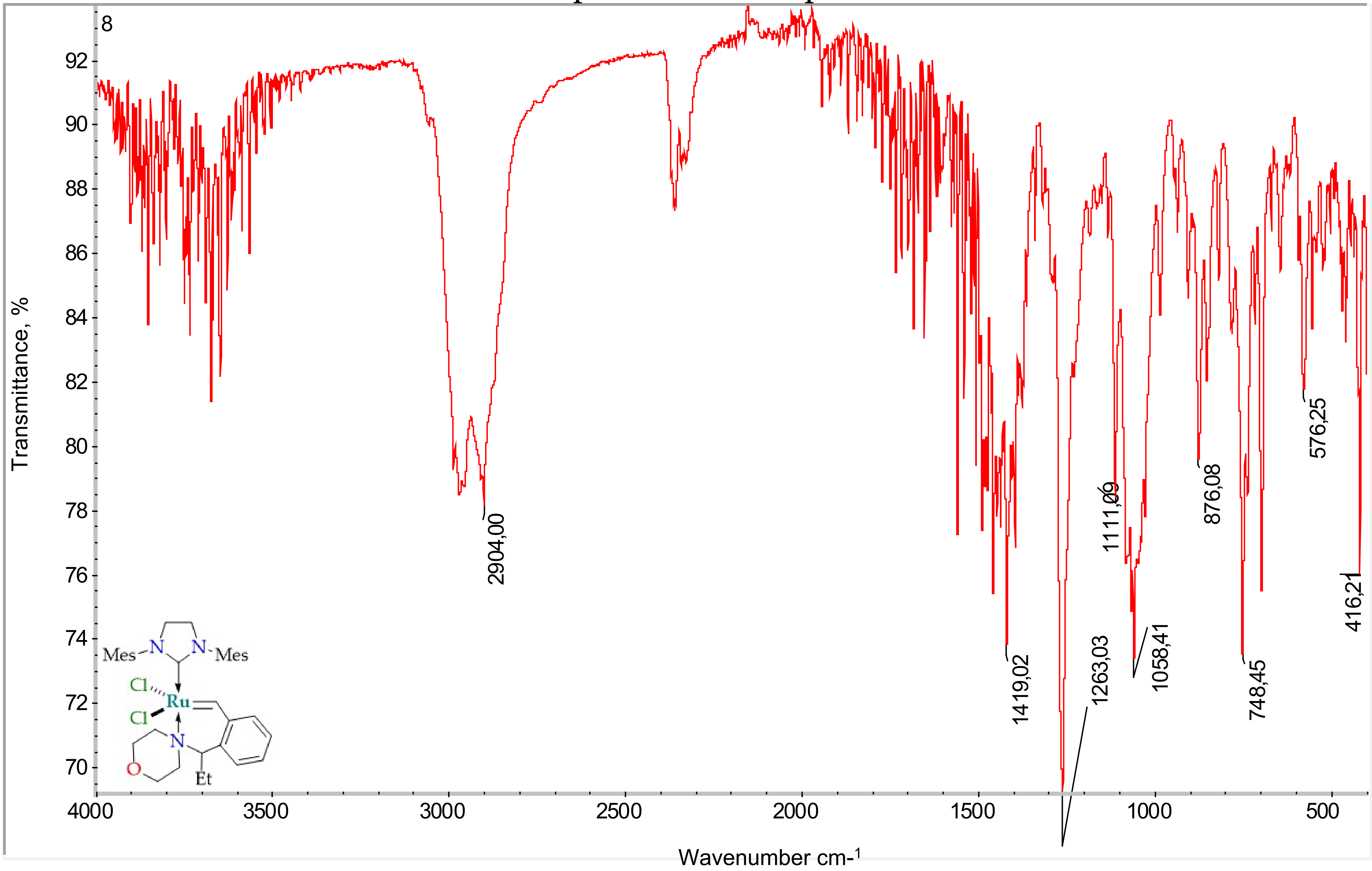

MALDI-ToF MS of compound **5d**

Display Report

Analysis Info

Analysis Name	D:\Data\Surzhikova\MVGA-5d.d	Acquisition Date	12.01.2023 13:30:04
Method	tune_100-1200.m	Operator	BDAL@DE
Sample Name		Instrument / Ser#	maXis 43
Comment			

Acquisition Parameter

Source Type	ESI	Ion Polarity	Positive	Set Nebulizer	1.0 Bar
Focus	Active			Set Dry Heater	200 °C
Scan Begin	50 m/z	Set Capillary	4500 V	Set Dry Gas	4.0 l/min
Scan End	1800 m/z	Set End Plate Offset	-500 V	Set Divert Valve	Waste

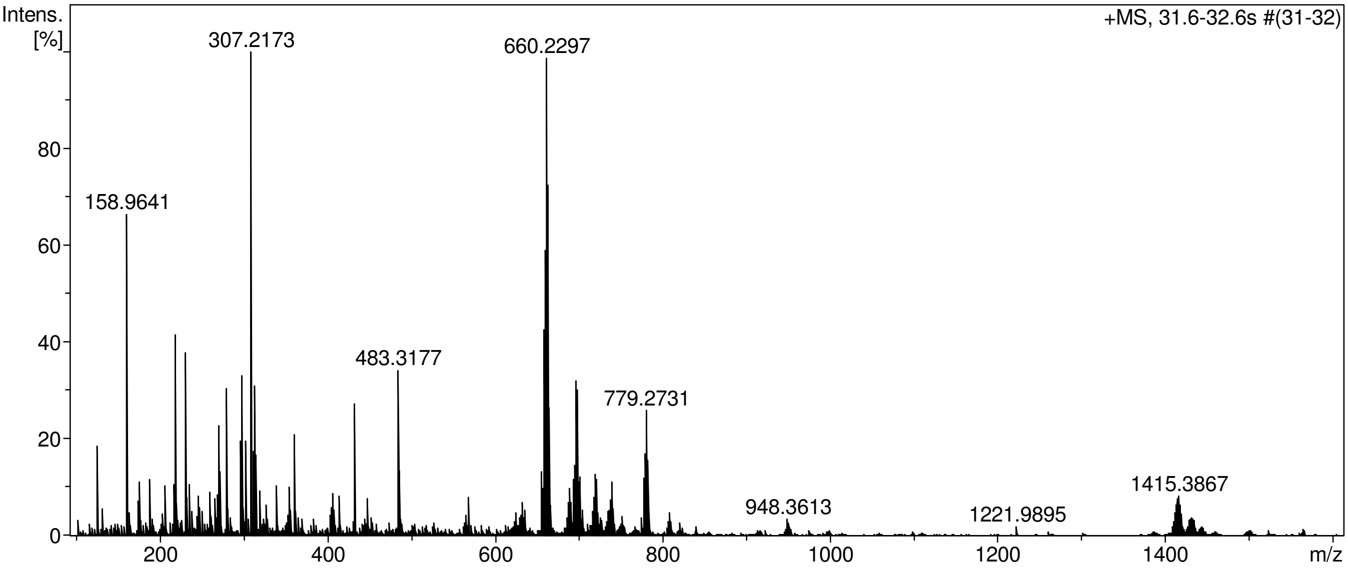

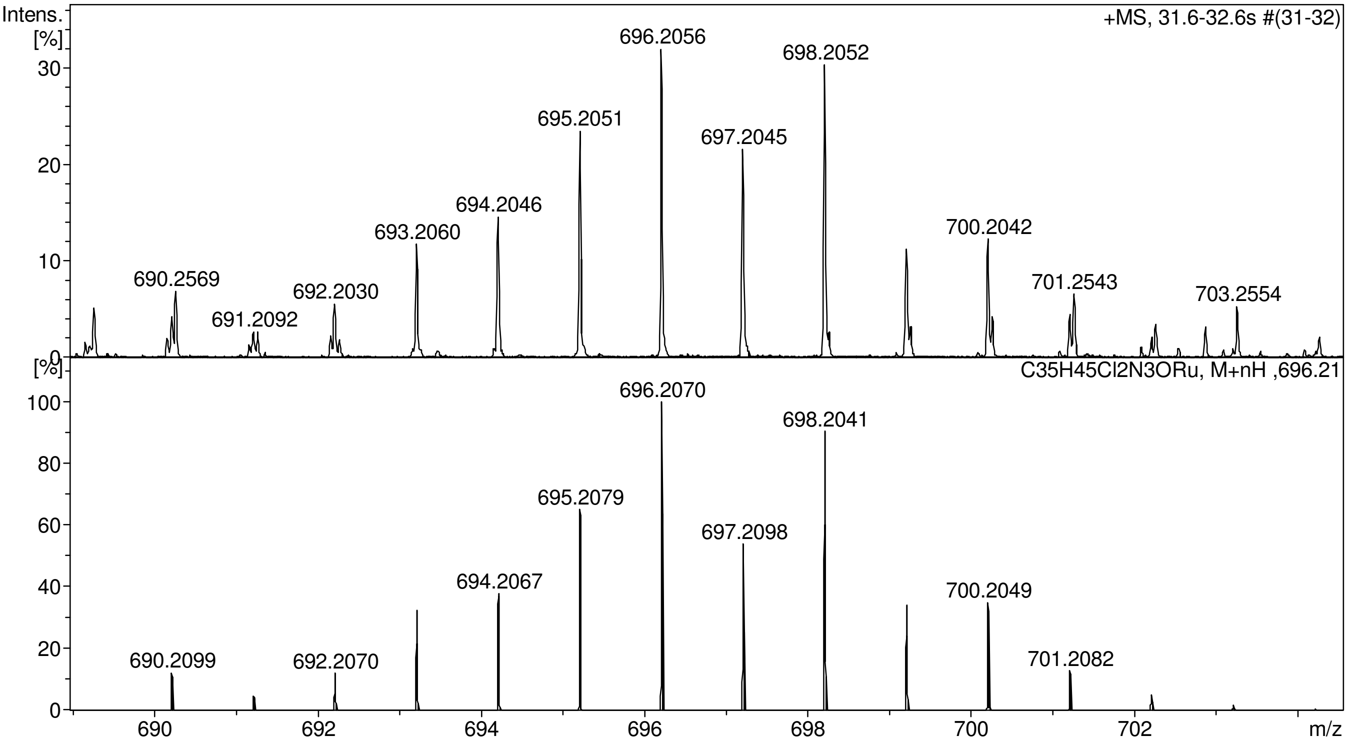

MALDI-ToF MS of compound **5d**

Display Report

Analysis Info

Analysis Name	D:\Data\Surzhikova\MVGA-5d.d	Acquisition Date	12.01.2023 13:30:04
Method	tune_100-1200.m	Operator	BDAL@DE
Sample Name		Instrument / Ser#	maXis 43
Comment			

Acquisition Parameter

Source Type	ESI	Ion Polarity	Positive	Set Nebulizer	1.0 Bar
Focus	Active			Set Dry Heater	200 ℃
Scan Begin	50 m/z	Set Capillary	4500 V	Set Dry Gas	4.0 l/min
Scan End	1800 m/z	Set End Plate Offset	-500 V	Set Divert Valve	Waste

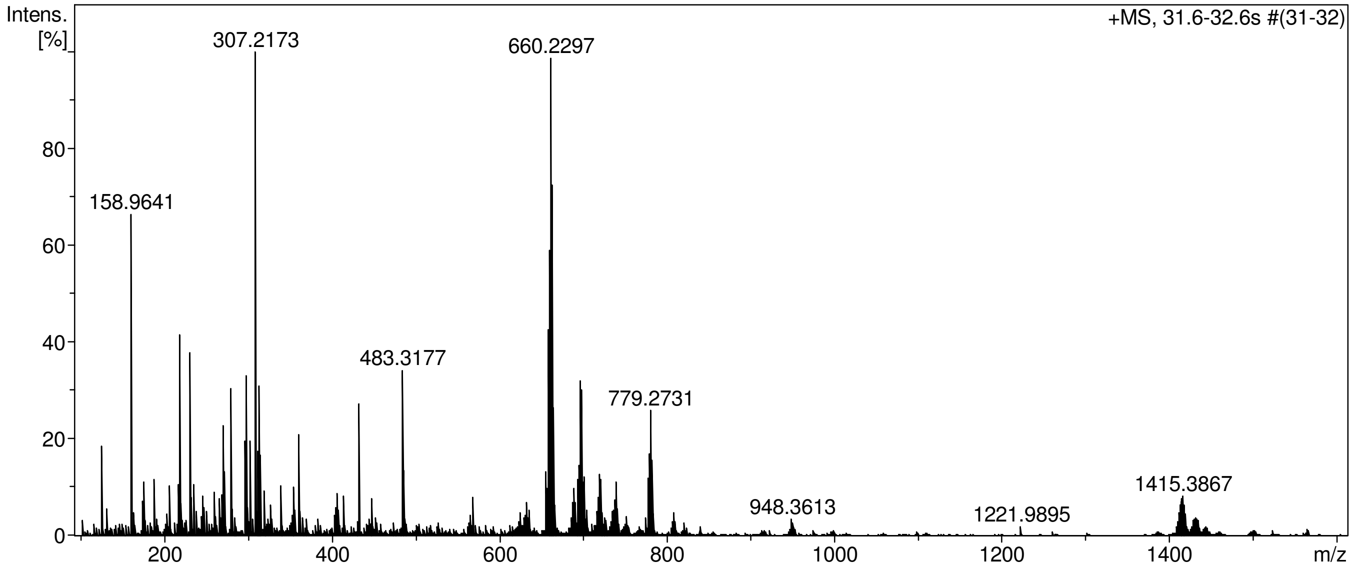

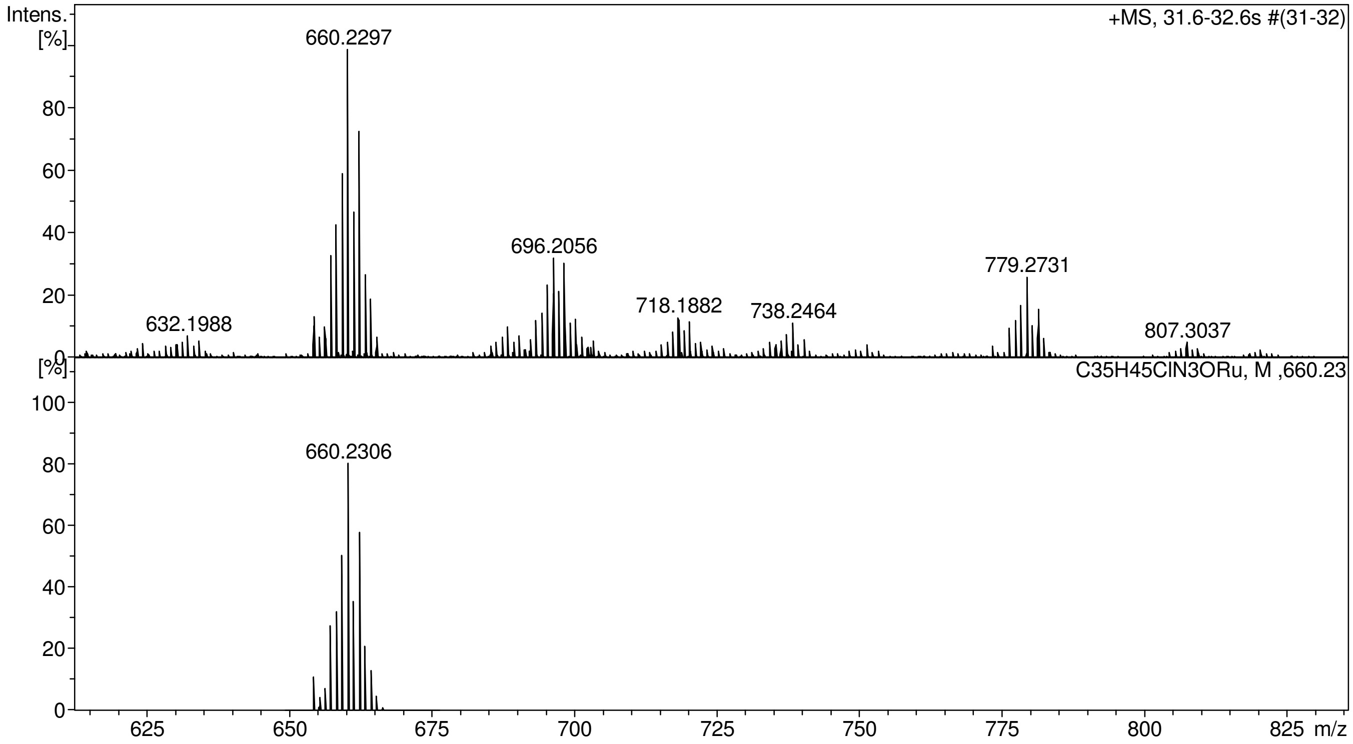

Supplement: Supplementary file 1 [file molecules-28-01188-s001.zip › molecules-2167903-NMR pics.pdf]
