# Supplementary material for: Influence of Substituents in a Six-Membered Chelate Ring of HG-Type Complexes Containing an N→Ru Bond on Their Stability and Catalytic Activity
Source: Molecules. 2023 Jan 25;28(3):1188. doi: 10.3390/molecules28031188 (PMC9921640; doi:10.3390/molecules28031188)
Supplement: Supplementary file 1 [file molecules-28-01188-s001.zip › molecules-2167903-Supplementary.pdf]

Electronic Supplementary Materials for the Article

Influence of Substituents in a Six-Membered Chelate Ring of HG-Type Complexes Containing an N→Ru Bond on Their Stability and Catalytic Activity

Kirill A. Vasilyev [1], Alexandra S. Antonova [1], Nikita S. Volchkov [1], Nikita A. Logvinenko [1], Eugeniya V. Nikitina [1], Mikhail S. Grigoriev [2], Anton P. Novikov [1,2], Vladimir V. Kouznetsov [3], Kirill B. Polyanskii [1,*] and Fedor I. Zubkov [1,*]

[1] Organic Chemistry Department, Faculty of Science, Peoples' Friendship University of Russia (RUDN University), Miklukho-Maklaya St., 6, Moscow 117198, Russia

[2] Frumkin Institute of Physical Chemistry and Electrochemistry, Russian Academy of Sciences, Leninsky pr. 31, bld. 4, Moscow 119071, Russia

[3] Laboratorio de Química Orgánica y Biomolecular, Escuela de Química, Universidad Industrial de Santander, Cl. 9 # Cra 27, Bucaramanga 680006, Colombia

* Correspondence: 1236tgp@mail.ru (K.B.P.); fzubkov@sci.pfu.edu.ru (F.I.Z.)

Content

General remarks 2

Experimental Procedures 2

1. *Synthesis of styrenes 1a–c and 2a–c* 3

2. *Synthesis of styrenes 3a–c* 5

3. *Synthesis of styrenes 1d and 3d* 7

4. *Optimization of conditions for complexes 4 and 5 preparation* 9

5. *General procedure for synthesis of complexes 4 and 5* 10

6. *Activity of 4 and 5 in RCM reactions with N,N-diallyltosylamide* 14

7. *Polymerization of norborn-5-ene-2,3-dicarboxylic acid dibutyl ester (DBNB) catalyzed by complexes 4 and 5* 16

8. *X-ray analysis* 17

9. *References* 42

10. *Copies of NMR, FTIR and mass spectra for compounds 1–5* 42

General remarks

All experiments with ruthenium derivatives were performed under an inert atmosphere (Ar), using standard Schlenk techniques, dry and oxygen-free solvents. Before experiments, toluene, THF and heptane were distilled over sodium under an argon atmosphere. Metathesis reactions required solvents (CH_2Cl_2) pre-dried over anhydrous P_2O_5 and an inert atmosphere (dry Ar). All other reagents were purchased from commercial suppliers (Acros Organics and Merck) and used without further purification. Thin layer chromatography (TLC) was carried out on aluminum backed silica pre-coated plates «Sorbfil» or «Alugram». The plates were visualized using a water solution of $KMnO_4$ or UV (254 nm). Eluents were *n*-hexane for styrenes and *n*-hexane : ethyl acetate = 3:1 for ruthenium complexes.

^1H and ^{13}C NMR spectra were recorded in $CDCl_3$ solutions on 700 MHz (700.2 MHz for ^1H and 176.1 MHz for ^{13}C), 600 MHz (600.1 MHz for ^1H and 150.9 MHz for ^{13}C), 500 MHz (500.1 MHz for ^1H and 125.8 MHz for ^{13}C) or 300 MHz (300.1 MHz for ^1H and 75.5 MHz for ^{13}C) spectrometers using residual solvent signals (δ 7.27/77.0 ppm for $^1H/^{13}C$ $CDCl_3$) or $SiMe_4$ as an internal standard. Chemical shifts are given relative to $SiMe_4$ or referenced to the residual solvent signals. In some cases, the obtained in $CDCl_3$ NMR spectra were strongly broadened due to the slow rotation on the Mes fragments around the C-N bonds. To overcome this difficulty, the spectra in dichloromethane (CD_2Cl_2, δ 5.36/53.4 ppm residual signals) were recorded. Assignments of ^1H, ^{13}C signals were made with the aid of 2D COSY, HSQC, and HMBC spectra.

IR spectra were obtained using an Infralum FT-801 (in KBr pellets or in thin films) or Nicolet 6700 FTIR spectrometers (for powders deposited on a diamond crystal) over the wavenumber ranges 4800–400 cm^{-1}. HR-MALDI-ToF mass spectra for styrene ligands were recorded using a ToF mass spectrometer Bruker autoflex speed, equipped with a solid-state UV-laser of 355 nm and operated in positive reflectron mode. Solutions of the analytes (2 mg/mL) in CH_2Cl_2 were deposited on steel targets (MTP 384 ground steel; Bruker Daltonics Inc., Germany) and air-dried. High-resolution mass spectra (ESI-MS TOF) of the Grubbs catalysts were recorded on a Bruker maXis Q-TOF instrument (Bruker Daltonik) equipped with an electrospray ionization (ESI) source. The measurements were performed in positive (+) MS ion mode (HV capillary: 4.5 kV; spray shield: –0.5 kV) with a scan range of m/z 50–1800. External calibration of the mass spectrometer was performed using a low-concentration using mix solution (Agilent Technologies). Direct syringe injection was applied to the solution in MeCN. Nitrogen was used as the nebulizing (1.0 bar) and drying gas (200 °C, 4.0 L×min^{-1}). All spectra were recorded at 1 Hz frequency and processed using the Bruker Data Analysis 5.1 software package.

Microanalyses were performed for C, H, N on an Eurovector EA 3000 (CHNS) elemental analysis system and were within ±0.5% of theoretical values.

Experimental Procedures

1-Alkyl-3,4-dihydroisoquinolines were used as precursors for preparation of **1a**–**c** and **2a**–**c**. Synthesis of 1-substituted-3,4-dihydroquinolines includes acylation of phenethylamine and a further cyclization of the resulting amide by the Bischler-Napieralski reaction according to previously described procedures [1].

1. *Synthesis of styrenes 1a–c and 2a–c*

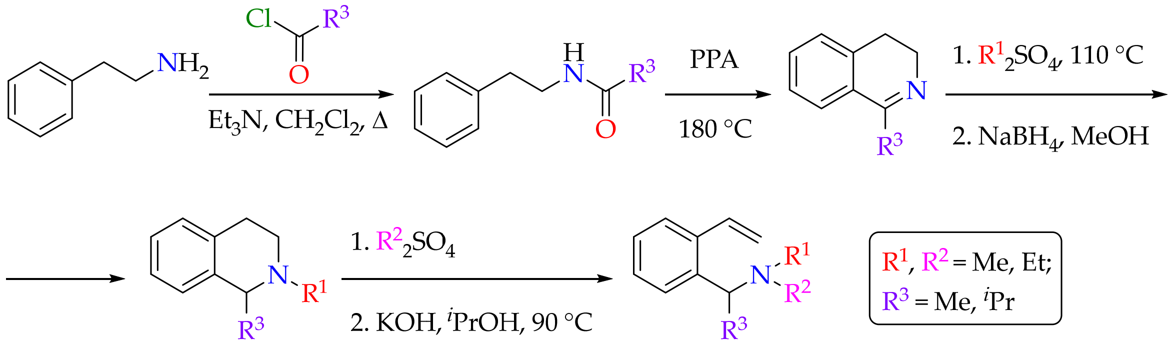

1a–c, 2a–c

Scheme S1. Synthesis of 2-vinylbenzylamines **1a–c** and **2a–c**

1-Alkyl-3,4-dihydroisoquinoline (0.068 mol, 1 eqv.) was stirred with an equimolar amount of dialkyl sulfate (0.068 mol, 1 eqv.) in a two-necked flask (250 mL). The reaction vessel was heated at 110 °C for 1 h. After cooling, methanol (100 mL) was added into the flask. Then, $NaBH_4$ (0.68 mol, 10 eqv.) was portionwise added at 0 °C with vigorous stirring. The resulting reaction mixture was stirred at r.t. for 2 h. Then it was poured into ice-cold water (150 mL) and organic products were extracted with Et_2O (2 × 150 mL). The combined organic layers were dried over anhydrous sodium sulfate. After filtration, the solvent was evaporated, and resulting viscous oil (THQ) was used at the next stage without additional purification.

An equimolar amount of dialkyl sulfate was added (0.067 mol, 1 eqv.) to 1,2-dialkyl-1,2,3,4-tetrahydroisoquinoline (0.067 mol, 1 eqv.) and the resulting mixture was stirred under reflux for 1 h. After cooling to r.t., the reaction mass solidified, isopropanol (50 mL) was added and a reaction vessel was heated at 90 °C for 20 min. At that temperature, pellets of KOH were portionwise added (0.13 mol, 2 eqv.) and the reaction mixture was stirred for another 1.5 h at 90 °C. After that, isopropanol was evaporated under reduced pressure, and the obtained slurry was diluted with water (100 mL) and extracted with Et_2O (2 x 100 mL). The combined organic layers were dried over anhydrous sodium sulfate. After filtration, the solvent was evaporated and the resulting viscous oil was further purified by flash chromatography (*n*-hexane, SiO_2).

1.1 *N,N-Dimethyl-1-(2-vinylphenyl)ethan-1-amine (1a)*

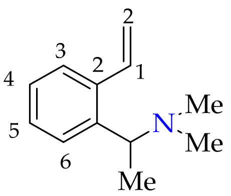

Pale-yellow oil, 3.90 g, 0.018 mol, yield 84%.

^{1}H NMR (600 MHz, $CDCl_3$) δ 7.44–7.43 (m, 2H, H-3,6-Ph), 7.25–7.19 (m, 3H, H-4,5-Ph, C<u>H</u>=CH_2), 5.56 (dd, J = 1.5 and J = 17.2 Hz, 1H, H-2-*trans*), 5.27 (dd, J = 1.5 and J = 11.1 Hz, 1H, H-2-*cis*), 3.51 (q, J = 6.6 Hz, 1H, C<u>H</u>Me), 2.20 (s, 6H, 2 × Me_2N), 1.30 (d, J = 6.6, 3H, <u>Me</u>CH) ppm. ^{13}C NMR (150 MHz, $CDCl_3$) δ 142.5, 136.8, 135.2, 127.9, 127.0, 126.7, 126.3, 115.7, 62.0, 43.8 (2C), 20.00 ppm. IR spectrum: ν (C=C) 1640 cm^{-1}. HRMS (MALDI-TOF) $[M + H]^+$ calcd for $C_{12}H_{17}N$: 176.1395, found: 176.1437.

1.2 *N-Ethyl-N-methyl-1-(2-vinylphenyl)ethan-1-amine (1b)*

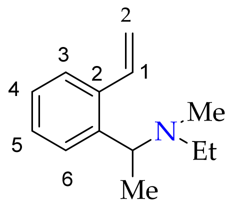

Pale-yellow oil, 3.24 g, 0.012 mol, yield 72%.

^{1}H NMR (600 MHz, $CDCl_3$) δ 7.45–7.43 (m, 2H, H-3,6-Ph), 7.27–7.18 (m, 3H, H-4,5-Ph, C$\underline{H}$=CH_2), 5.56 (dd, *J* = 1.5 and *J* = 17.7 Hz, 1H, H-2-*trans*), 5.26 (dd, *J* = 1.5 and *J* = 11.1 Hz, 1H, H-2-*cis*), 3.76 (q, *J* = 6.6 Hz, 1H, CHMe), 2.54–2.49 (m, 1H, C$\underline{H}_2$Me), 2.38–2.32 (m, 1H, CH_2Me), 2.20 (s, 3H, MeN), 1.29 (d, *J* = 6.6 Hz, 3H, $\underline{Me}$CH), 0.99 (t, *J* = 7.1 Hz, 3H, $\underline{Me}CH_2$) ppm. ^{13}C NMR (150 MHz, $CDCl_3$) δ 142.7, 137.0, 135.4, 127.8, 127.0, 126.7, 126.3, 115.6, 59.7, 48.7, 38.2, 18.9, 12.1 ppm. IR spectrum: ν (C=C) 1642 cm^{-1}. HRMS (ESI-TOF) [M + H]$^+$ calcd for $C_{13}H_{19}N$: 190.1551, found: 190.1579.

1.3 N,N-Diethyl-1-(2-vinylphenyl)ethan-1-amine (1c)

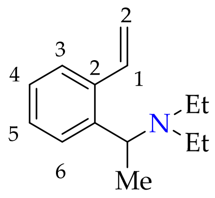

Pale-yellow oil, 3.55 g, 0.013 mol, yield 78%.

^{1}H NMR (700 MHz, $CDCl_3$) δ 7.47 (br.d, *J* = 7.6, 1H, H-3-Ph), 7.44 (br.d, *J* = 7.6, 1H, H-6-Ph), 7.29 (dd, *J* = 11.0 and *J* = 17.4 Hz, 1H, C$\underline{H}$=CH_2), 7.24 (br.t, *J* = 7.6 Hz, 1H, H-5-Ph), 7.20 (br.t, *J* = 7.6 Hz, 1H, H-4-Ph), 5.57 (dd, *J* = 1.3 and *J* = 17.4 Hz, 1H, H-2-*trans*), 5.26 (dd, *J* = 1.3 and *J* = 11.0 Hz, 1H, H-2-*cis*), 4.03 (q, *J* = 6.7 Hz, 1H, C$\underline{H}$Me), 2.64–2.54 (m, 4H, 2 × C$\underline{H}_2$Me), 1.28 (d, *J* = 6.7 Hz, 3H, $\underline{Me}$CH), 0.95 (t, *J* = 7.2 Hz, 6H, 2 × $\underline{Me}CH_2$) ppm. ^{13}C NMR (150 MHz, $CDCl_3$) δ 143.1, 137.0, 135.4, 127.6, 127.1, 126.4, 126.1, 115.2, 56.2, 42.7 (2C), 18.8, 11.5 (2C) ppm. IR spectrum: ν (C=C) 1638 cm^{-1}. HRMS (ESI-TOF) [M + H]$^+$ calcd for $C_{14}H_{21}N$: 204.1708, found: 204.1751.

1.4 N,N-Dimethyl-1-(2-vinylphenyl)propan-1-amine (2a)

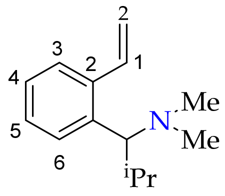

Pale-yellow oil, 2.12 g, 0.0055 mol, yield 53%.

^{1}H NMR (600 MHz, $CDCl_3$) δ 7.45 (dd, *J* = 1.2 and 7.4 Hz, 1H, H-3-Ph), 7.28–7.20 (m, 3H, H-4,5,6-Ph), 7.16 (dd, *J* = 11.1 and *J* = 17.1 Hz, 1H, C$\underline{H}$=CH_2), 5.52 (dd, *J* = 1.5 and *J* = 17.1 Hz, 1H, H-2-*trans*), 5.27 (dd, *J* = 1.5 and *J* = 11.1 Hz, 1H, H-2-*cis*), 3.48 (d, *J* = 8.1 Hz, 1H, C$\underline{H}$^{i}Pr), 2.26–2.20 (m, 1H, C$\underline{H}$(Me)$_2$), 2.13 (s, 6H, 2 × Me_2N), 0.93 (d, *J* = 6.6 Hz, 3H, A-$\underline{Me}$CH), 0.75 (d, *J* = 7.1 Hz, 3H, B-$\underline{Me}$CH) ppm. ^{13}C NMR (150 MHz, $CDCl_3$) δ = 139.1, 136.2, 136.0, 128.1, 126.8, 126.5, 126.4, 115.9, 69.4, 42.4 (2C), 29.9, 20.6, 18.6 ppm. IR spectrum: ν (C=C) 1643 cm^{-1}. HRMS (MALDI-TOF) [M + H]$^+$ calcd for $C_{14}H_{21}N$: 204.1708, found: 204.1738.

1.5 *N-Ethyl-N-methyl -1-(2-vinylphenyl)propan-1-amine (2b)*

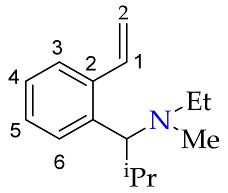

Pale-yellow oil, 2.31 g, 0.0045 mol, yield 42%.

^{1}H NMR ($CDCl_3$, 600 MHz): δ 7.45 (br.d, J = 7.6, 1H, H-3-Ph), 7.27 (m, 1H, H-6-Ph,), 7.24–7.14 (m, 3H, H-4,5-Ph, C<u>H</u>=CH_2), 5.52 (dd, J = 17.2 and 1.5 Hz, 1H, H-2-*trans*), 5.26 (dd, J = 10.6 and 1.5 Hz, 1H, H-2-*cis*), 3.62 (d, J = 8.6 Hz, 1H, C<u>H</u>^{i}Pr), 2.48–2.43 (m, 1H, C<u>H</u>$(Me)_2$), 2.26–2.20 (m, 2H, C<u>H</u>$_2$Me), 2.12 (s, 3H, N-Me), 0.98 (t, J = 7.2 Hz, 3H, <u>Me</u>CH_2), 0.96 (d, J = 6.6 Hz, 3H, A-<u>Me</u>CH), 0.71 (d, J = 7.1 Hz, 3H, B-<u>Me</u>CH) ppm. ^{13}C NMR ($CDCl_3$, 176 MHz): δ = 139.0, 136.8, 136.3, 128.0, 126.8, 126.40, 126.38, 115.9, 68.1, 47.2, 37.8, 29.8, 20.7, 19.0, 12.7 ppm. IR spectrum: ν (C=C) 1645 cm^{-1}. HRMS (MALDI-TOF) [M + H]$^+$ calcd for $C_{16}H_{25}N$: 218.1864, found: 218.1914.

1.6 *N,N-Diethyl-1-(2-vinylphenyl)propan-1-amine (2c)*

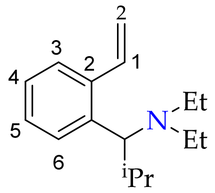

Pale-yellow oil, 2.27 g, 0.0049 mol, yield 50%.

^{1}H NMR ($CDCl_3$, 600 MHz): δ 7.43 (br.d, J = 7.6 Hz, 1H, H-3-Ph), 7.30 (br.d, J = 7.6 Hz, 1H, H-6-Ph,), 7.24–7.18 (m, 2H, H-4,5-Ph), 7.14 (dd, J = 10.8 and J = 17.1 Hz, 1H, C<u>H</u>=CH_2), 5.52 (dd, J = 17.4 and 1.4 Hz, 1H, H-2-*trans*), 5.26 (dd, J = 10.8 and 1.4 Hz, 1H, H-2-*cis*), 3.72 (d, J = 8.8 Hz, 1H, C<u>H</u>^{i}Pr), 2.58 (dq, J = 7.2 and 13.4 Hz, 2H, C<u>H</u>$_2$Me), 2.26 (dq, J = 7.2 and 13.4 Hz, 2H, C<u>H</u>$_2$Me), 2.23–2.19 (m, 1H, C<u>H</u>$(Me)_2$), 0.96 (m, 9H, 2 × <u>Me</u>CH_2N, A-<u>Me</u>CH), 0.70 (d, J = 6.7 Hz, 3H, B-<u>Me</u>CH) ppm. ^{13}C NMR ($CDCl_3$, 176 MHz): δ = 139.0, 138.2, 136.4, 128.0, 126.8, 126.30, 126.28, 115.9, 64.5, 42.6 (2C), 29.9, 20.9, 19.4, 13.3 (2C) ppm. IR spectrum: ν (C=C) 1646 cm^{-1}. HRMS (MALDI-TOF) [M + H]$^+$ calcd for $C_{16}H_{25}N$: 232.2021, found: 232.2045.

2. *Synthesis of styrenes 3a−c*

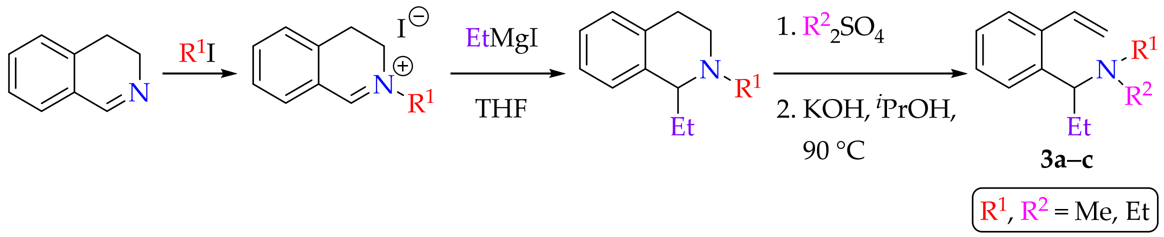

Scheme S2. Synthesis of *α*-ethyl substituted styrenes **3a−c**

3,4-Dihydroisoquinoline (5.00 g, 0.038 mol, 1 eqv.) was added dropwise to alkyl iodide (0.38 mol, 10 eqv.) pre-cooled to 0 °C. Then the reaction mixture was heated at 110 °C for 16 h. Obtained solid was filtered, washed with hexane (3 × 50 mL), diethyl ether (3 × 50 mL) and air-dried. The resulting

salt (0.0087 mol, 1 eqv.) was used in the next step (the Grignard synthesis) without further purification.

Grignard reagent (0.01 mol, 1.2 eqv.) was added to the salt dissolved in abs. THF (50 mL) at 0 °C under an argon atmosphere, stirred for an hour while cooling and further left stirred for two days at r.t. Then it was gently poured into cold water (100 mL) and extracted with Et_2O (2 × 100 mL). The combined organic layers were dried over anhydrous sodium sulfate. After filtration, the solvent was evaporated and the resulting viscous oil (THQ) was used at the next stage without further purification.

1-Ethyl-2-alkyl-1,2,3,4-tetrahydroisoquinoline (0.0086 mol, 1 eqv.) was stirred with the corresponding dialkyl sulfate (0.0086 mol, 1 eqv.) for 1 h at 110 °C. Then the mixture was cooled to r.t., isopropanol (50 mL) and KOH (0.019 mol, 2.2 eqv.) were added and the reaction mixture was heated at 90 °C for 1.5 h. Then isopropanol was evaporated under reduced pressure to give a thick slurry. It was diluted with water (2 × 100 mL) and extracted with Et_2O (3 × 100 mL). After that, the combined organic layers were dried over anhydrous sodium sulfate. After filtration, the solvent was evaporated to give light-yellow oil, which was purified by flash chromatography on silica gel (EtOAc : hexane - 1:10).

2.1 N,N-Dimethyl-1-(2-vinylphenyl)propan-1-amine (3a)

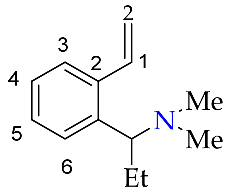

Pale-yellow oil, 3.02 g, 0.012 mol, yield 75%.

^{1}H NMR (600 MHz, $CDCl_3$) *δ* 7.45 (br.d, *J* = 7.6 Hz, 1H, H-3-Ph), 7.36 (br.d, *J* = 8.1 Hz, 1H, H-6-Ph), 7.25–7.20 (m, 3H, H-4,5-Ph, C<u>H</u>=CH_2), 5.56 (dd, *J* = 1.5 and *J* = 17.7 Hz, 1H, H-2-*trans*), 5.26 (dd, *J* = 1.5 and *J* = 11.1 Hz, 1H, H-2-*cis*), 3.37 (dd, *J* = 4.0 and *J* = 9.6 Hz, 1H, C<u>H</u>Et), 2.20 (s, 6H, Me_2N), 1.94–1.90 (m, 1H, A-C<u>H</u>$_2$Me), 1.73–1.68 (m, 1H, B-C<u>H</u>$_2$Me), 0.65 (t, *J* = 7.1 Hz, 3H, <u>Me</u>CH_2) ppm. ^{13}C NMR (150 MHz, $CDCl_3$) *δ* 139.4, 138.0, 135.5, 127.7, 127.5, 126.7, 126.2, 115.6, 67.8, 43.7 (2C), 25.9, 10.3 ppm. IR spectrum: ν (C=C) 1639 cm^{-1}. HRMS (MALDI-TOF) $[M + H]^+$ calcd for $C_{13}H_{19}N$: 190.1551, found: 190.1553.

2.2 N-Ethyl-N-methyl-1-(2-vinylphenyl)propan-1-amine (3b)

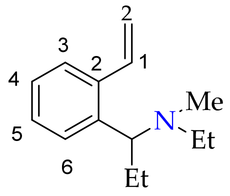

Pale-yellow oil, 3.59 g, 0.012 mol, yield 70%.

^{1}H NMR (600 MHz, $CDCl_3$) *δ* 7.45 (d, *J* = 7.6 Hz, 1H, H-3-Ph), 7.37 (d, *J* = 8.1 Hz, 1H, H-6-Ph), 7.26–7.18 (m, 3H, H-4,5-Ph, C<u>H</u>=CH_2), 5.56 (dd, *J* = 1.5 and *J* = 17.2 Hz, 1H, H-2-*trans*), 5.25 (dd, *J* = 1.5 and *J* = 10.6 Hz, 1H, H-2-*cis*), 3.60 (dd, *J* = 4.0 and *J* = 9.6 Hz, 1H, C<u>H</u>Et), 2.53–2.48 (m, 1H, A-NC<u>H</u>$_2$Me), 2.38–2.33 (m, 1H, B-NC<u>H</u>$_2$Me), 2.20 (s, 3H, MeN), 1.93–1.89 (m, 1H, CHC<u>H</u>$_2$Me), 1.74–1.70 (m, 1H, CHC<u>H</u>$_2$Me), 0.98 (t, *J* = 7.1 Hz, 3H, <u>Me</u>CH_2N), 0.66 (t, *J* = 7.6 Hz, 3H, <u>Me</u>CH_2CH) ppm. ^{13}C NMR (150 MHz, $CDCl_3$) *δ* 139.8, 135.7, 127.9, 127.5, 126.7, 126.3, 125.3, 115.7, 64.6, 47.4, 37.3, 24.2, 10.9, 9.4 ppm.

IR spectrum: ν (C=C) 1640 cm^{-1}. HRMS (MALDI-TOF) $[M + H]^+$ calcd for $C_{14}H_{21}N$: 204.1708, found: 204.1741.

2.3 *N,N-Diethyl-1-(2-vinylphenyl)propan-1-amine (3c)*

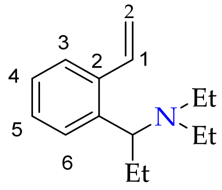

Pale-yellow oil, 3.32 g, 0.010 mol, yield 68%.

^{1}H NMR (600 MHz, $CDCl_3$) δ 7.45 (br.d, *J* = 7.6 Hz, 1H, H-3-Ph), 7.37 (br.d, *J* = 8.1 Hz, 1H, H-6-Ph), 7.32–7.18 (m, 3H, H-4,5-Ph, C<u>H</u>=CH_2), 5.56 (dd, *J* = 1.5 and *J* = 17.7 Hz, 1H, H-2-*trans*), 5.25 (dd, *J* = 1.5 and *J* = 10.6 Hz, 1H, H-2-*cis*), 3.84 (dd, *J* = 4.0 and *J* = 9.6 Hz, 1H, C<u>H</u>Et), 2.64–2.51 (m, 4H, 2 × NC<u>H</u>$_2$Me), 1.93–1.89 (m, 1H, A-CHC<u>H</u>$_2$Me), 1.72–1.66 (m, 1H, B-CHC<u>H</u>$_2$Me), 0.95 (t, *J* = 7.1 Hz, 6H, 2 × <u>Me</u>CH_2N), 0.65 (t, *J* = 7.1 Hz, 3H, <u>Me</u>CH_2CH) ppm. ^{13}C NMR (150 MHz, $CDCl_3$) δ 139.4, 137.2, 134.8, 127.0, 126.3, 125.4, 125.0, 114.2, 61.5, 41.8 (2C), 24.3, 10.7 (2C), 9.7 ppm. IR spectrum: ν (C=C) 1640 cm^{-1}. HRMS (MALDI-TOF) $[M + H]^+$ calcd for $C_{15}H_{23}N$: 218.1864, found: 218.1917.

3. *Synthesis of styrenes 1d and 3d*

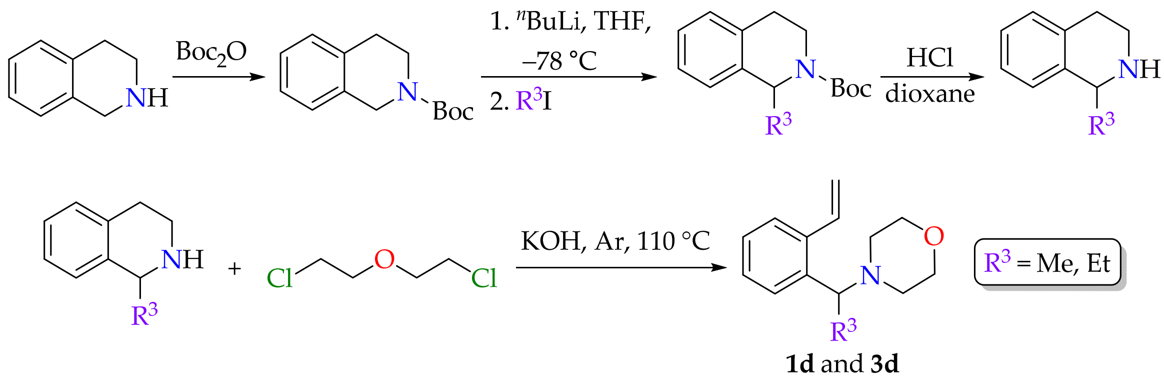

Scheme S3. Synthesis of styrenes **1d** and **3d**

A solution of 1,2,3,4-tetrahydroisoquinoline (13.32 g, 0.1 mol, 1 eqv.) in DCM (40 mL) was added dropwise for a period of 5 min to a solution of Boc_2O (24 g, 0.11 mol, 1.1 eqv.) in DCM (80 mL). The reaction mixture was stirred at r.t. for 2 h, then trifluoroacetic acid (2 mL) was added and the flask was placed in an ultrasonic bath for 10 min to remove an excess of Boc_2O. The solvent was evaporated under reduced pressure.

In a three-necked flask (250 mL), N-Boc-1,2,3,4-tetrahydroisoquinoline (10 g, 0.043 mol, 1 eqv.) was dissolved in abs. THF (100 mL) under an argon atmosphere. The reaction vessel was cooled to –60 °C and a solution of *n*-butyllithium in hexane (26 mL, 2.5 M) was slowly added dropwise with vigorous stirring at the mentioned temperature. Then, the reaction was stirred at –60 °C for 1 h. Alkyl iodide (0.13 mol, 3 eqv.) was added dropwise at –60 °C and stirred at this temperature for additional 30 min. The reaction mixture was left to heat up to r.t. and then was stirred overnight at r.t. Methanol

(100 mL) and water (200 mL) were added and the resulting mixture was extracted with Et_2O (2 x 200 mL). After that, the combined organic layers were dried over anhydrous sodium sulfate. After filtration, the solvent was evaporated under reduced pressure.

The obtained N-Boc-1-alkyl-1,2,3,4-tetrahydroisoquinoline (0.017 mol, 1 eqv.) was dissolved in dioxane (100 mL), then 20% HCl in dioxane (31 mL, 0.17 mol, 10 eqv.) was added. The reaction mixture was left to stir overnight. The solution was basified with an aqueous solution of NaOH to pH 12 and was extracted with Et_2O (2 x 100 mL). The organic layers were dried over anhydrous sodium sulfate. After filtration, the solvent was evaporated under reduced pressure.

Without further purification a mixture of 1-alkyl-1,2,3,4-tetrahydroisoquinoline (0.02 mol, 1 eqv.), *bis*(chloroethyl) ether (3.29 g, 0.023 mol, 1.1 eqv.) and KOH (2.55 g, 0.045 mol, 2.3 eqv.) was heated without solvent under an inert atmosphere for 7 h at 110 °C. Then water (200 mL) was added, and the resulting solution was extracted with ethyl acetate (2 x 200 mL). The combined organic layers were dried over anhydrous sodium sulfate. After filtration, the solvent was evaporated under reduced pressure. The resulting light-yellow styrene (**1d** and **3d**) was purified by flash chromatography on silica gel (ethyl acetate : hexane - 1:10).

3.1 4-(1-(2-Vinylphenyl)ethyl)morpholine (1d)

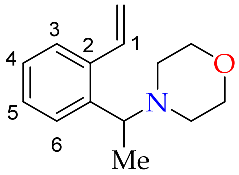

Pale-yellow oil, 1.72 g, 0.0067 mol, yield 85%.

^{1}H NMR (600 MHz, $CDCl_3$) δ 7.45 (m, 2H, H-3,6-Ph), 7.27 (dd, J = 11.1 and 17.2 Hz, 1H, C<u>H</u>=CH_2), 7.25 and 7.21 (dt and dt, 1H and 1H, J = 1.5 and 7.6 Hz, H-4-Ph and H-5-Ph), 5.56 (dd, J = 1.5 and J = 17.2 Hz, 1H, H-2-*trans*), 5.27 (dd, J = 1.5 and J = 11.1 Hz, 1H, H-2-*cis*), 3.70–3.63 (m, 4H, CH_2OCH_2), 3.61 (q, 1H, J = 6.6 Hz, C<u>H</u>Me), 2.50 (br.m, 2H, NCH_2), 2.37–2.34 (m, 2H, NCH_2), 1.29 (d, J = 6.6 Hz, 3H, CH<u>Me</u>) ppm. ^{13}C NMR (150 MHz, $CDCl_3$) δ 141.6, 137.0, 135.1, 127.8, 127.2, 126.8, 126.3, 115.6, 67.2 (2C), 61.4, 51.4 (2C), 19.1 ppm. IR spectrum: ν (C=C) 1630 cm^{-1}. HRMS (MALDI-TOF) $[M + H]^+$ calcd for $C_{14}H_{19}NO$: 218.1500, found: 218.1553.

3.2 4-(1-(2-Vinylphenyl)propyl)morpholine (3d)

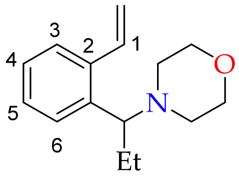

Pale-yellow oil, 1.59 g, 0.0056 mol, yield 81%.

^{1}H NMR (600 MHz, $CDCl_3$) δ 7.45 (dd, J = 1.5 and J = 7.6 Hz, 1H, H-3-Ph), 7.36 (br.d, J = 7.6 Hz, 1H, H-6-Ph), 7.28–7.19 (m, 3H, H-4,5-Ph, C<u>H</u>=CH_2), 5.55 (dd, J = 1.5 and J = 17.7 Hz, 1H, H-2-*trans*), 5.26 (dd, J = 1.5 and J = 11.1 Hz, 1H, H-2-*cis*), 3.69–3.63 (m, 4H, CH_2OCH_2), 3.46 (dd, J = 4.0 and J = 9.1 Hz, 1H, C<u>H</u>Et), 2.51 (m, 2H, A-NCH_2), 2.37–2.32 (m, 2H, B-NCH_2), 1.93–1.89 (m, 1H, A-CHC<u>H</u>$_2$Me), 1.71–1.67 (m, 1H, B-CHC<u>H</u>$_2$Me), 0.64 (t, J = 7.6 Hz, 3H, CH_2<u>Me</u>) ppm. ^{13}C NMR (150 MHz, $CDCl_3$) δ 138.8, 138.1, 135.5, 128.3, 127.5, 126.8, 126.3, 115.6, 67.3 (2C), 51.7 (2C), 51.6, 24.8, 10.1 ppm. IR spectrum: ν (C=C) 1628 cm^{-1}. HRMS (MALDI-TOF) $[M + H]^+$ calcd for $C_{15}H_{21}NO$: 232.1657, found: 232.1703.

4. *Optimization of conditions for complexes 4 and 5 preparation*

The initial conditions were chosen on the basis of the previous experiments [1] and proved to be suitable for obtaining chelates **4a–c**. However, α-ethyl or α-isopropyl substituted catalysts (**2a** and **3a**) could not be isolated by this way.

In this regard, we changed the solvent system. It is known that **Ind II** is highly soluble in toluene and poorly soluble in nonpolar solvents, while target catalysts precipitate much more efficiently from nonpolar solvents such as heptane. Initially, we carried out the reaction in pure heptane at reflux. However, **Ind II** did not dissolve entirely, so these conditions turned out to be unsuitable. Subsequently, the ratios of toluene and heptane were selected such at which **Ind II** was dissolved and the target catalysts were precipitated after cooling. Thus, the ratio of 1:5 (toluene - heptane, respectively) at 110 °C turned out to be the optimal. It should be noted, that catalysts **6** with an isopropyl group in the alpha-position could not be isolated, although they existed in solutions, which was confirmed by TLC. These complexes are precipitated reluctantly on cooling and dissolve even in ice-cold pentane when trying to separate them from the starting materials.

Table S1. Screening of conditions for synthesis of catalysts **4**, **5** and **6**.

Conditions	Styrene	Catalyst	Time, min	Yield, %
PhMe, 110 °C	**1a–c**	**4a–c**	90	> 62
	**2a**	**6a**	90	-*
	**2a**	**6a**	120	-*
	**3a**	**5a**	90	traces
	**3a**	**5a**	120	traces
heptane, 90 °C	**2a**	**6a**	90	0
	**3a**	**5a**	90	0
PhMe:heptane (1:1), 90 °C	**2a**	**6a**	90	-*
	**3a**	**5a**	90	15
	**3a**	**5a**	120	18
PhMe:heptane (1:3), 90 °C	**2a**	**6a**	90	-*
	**3a**	**5a**	90	27
PhMe:heptane (1:5), 90 °C	**2a**	**6a**	90	-*
	**3a**	**5a**	90	30
PhMe:heptane (1:5), 110 °C	**1a–c**	**4a–c**	90	> 70
	**2a**	**6a**	90	-*
	**3a**	**5a**	90	74
PhMe:heptane (1:7), 110 °C	**2a**	**6a**	90	-*
	**3a**	**5a**	90	20

* We were unable to isolated complex **6a**, but its formation was proved by TLC

5.   *General procedure for synthesis of complexes 4 and 5*

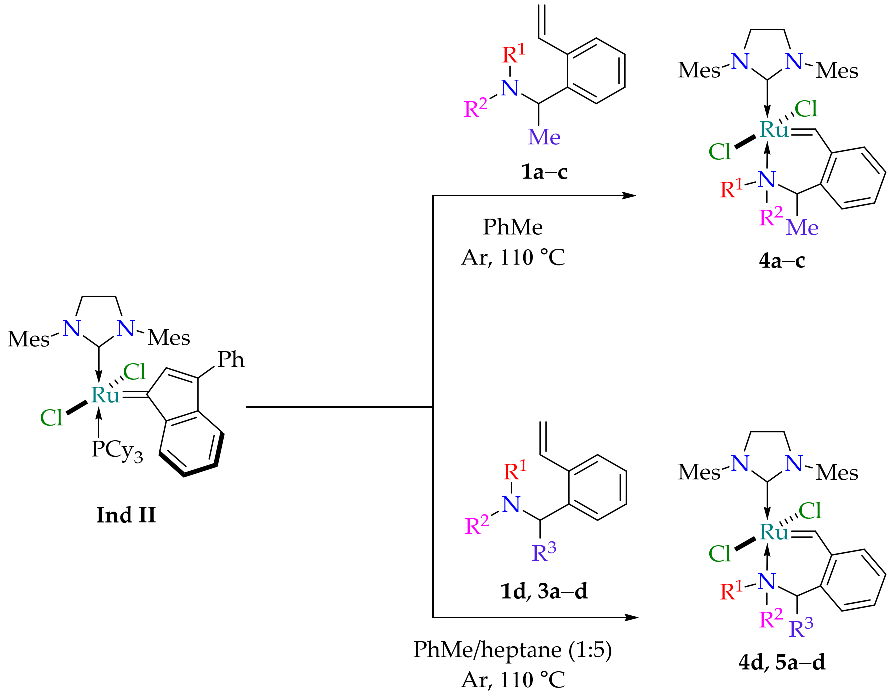

Scheme S4. Synthesis of ruthenium complexes **4a–d** and **5a–d**

In a Schlenk flask, the precursor of ruthenium complexes **IndII** (500 mg, 0.53 mmol, 1.0 eqv.) was suspended in abs. toluene (in the case of **1a–c**) or in a mixture of absolute heptane and toluene (V:V, 5:1) (in the case of **4d**, **5a–d**) (15 mL) under an argon atmosphere. Then, the reaction mixture was heated to 110 °C with continuous stirring and styrene **1**, **2** or **3** (0.58 mmol, 1.1 eqv.) was added in an argon stream. After that, the flask was sealed with a screw cap and heated at 110 °C for 1 h (40 min for **1d**, **3d**). After cooling to r.t., solvents were removed under reduced pressure, and 10 mL of cool pentane (−20 °C) were added to the obtained solid. Participate was filtered off and washed with hexane (3 × 5 mL) and with methanol (3 × 3 mL), both cooled to −20 °C to give the target catalyst (**4a–d**, **5a–d**) as a green powder after drying under vacuum.

5.1   [1,3-Bis(2,4,6-trimethylphenyl)imidazolin-2-ylidene](dichloro){2-[(dimethylamino)-1-ethyl]benzylidene}ruthenium (4a)

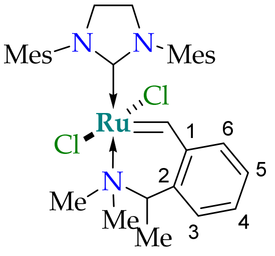

Green powder, 264 mg, 0.41 mmol, 78%, m.p. 203.1–203.5 °C.

^{1}H NMR (300.1 MHz, CD_2Cl_2, 30 °C) δ 18.76 (s, 1H, CH=Ru), 7.59 (br.t, *J* = 7.4 Hz, 1H, H-4-C_6H_4), 7.25–7.20 (m, 2H, H-3-C_6H_4 and H-5-C_6H_4), 7.11 and 7.05 (br.s and br.s, 2H and 2H, CH-3,5-Mes), 6.77 (d, *J* = 7.4 Hz, 1H, H-6-C_6H_4), 5.74 (q, *J* = 7.1 Hz, 1H, NC<u>H</u>Me), 4.12 (s, 4H, NCH_2CH_2N), 2.58 and 2.44 (br.s and br.s, 9H and 9H, Me-Mes), 2.07 (s, 3H, A-MeN), 1.54 (s, 3H, B-MeN), 1.40 (d, *J* = 7.1 Hz, 3H,

MeCH). ^{13}C NMR (150.9 MHz, $CDCl_3$, 25 °C) δ 312.2, 213.1, 148.8, 138.8 (br.s), 138.4, 137.2, 136.3 (very br.s), 129.2, 129.0, 128.4, 128.3, 127.0, 59.0, 51.5 (br.s), 43.2, 38.5, 20.8, 19.4 (very br.s), 9.6.

IR spectrum : 2953, 1620, 1593, 1417, 1310, 1264, 1190, 1031, 874, 815, 788.

HRMS (ESI-TOF) $[M]^+$ calcd. for $C_{32}H_{41}Cl_2N_3Ru$ 639.1716, found 639.1727.

5.2 [1,3-Bis(2,4,6-trimethylphenyl)imidazolin-2-ylidene](dichloro){2-[(methylethylamino)-1-ethyl]benzylidene}ruthenium (4b)

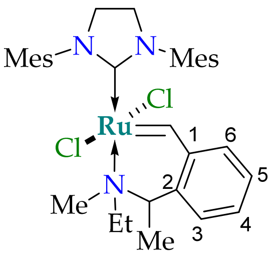

Green powder, 274 mg, 0.42 mmol, 79%, m.p. 202.8–203.2 °C.

^1H NMR (600.2 MHz, $CDCl_3$, 23 °C) δ 18.83 (s, 1H, CH=Ru), 7.48 (t, *J* = 7.6 Hz, 1H, H-4-C_6H_4), 7.15 (d, *J* = 7.6 Hz, 1H, H-5-C_6H_4), 7.11 (br.s, 1H, H-Mes), 7.10 (t, *J* = 7.6 Hz, 1H, H-3-C_6H_4), 7.02 (very br.s, 2H, CH-3,5-Mes), 6.91 (br.s, 1H, CH-Mes), 6.54 (d, *J* = 7.6 Hz, 1H, H-6-C_6H_4), 5.51 (q, *J* = 6.9 Hz, 1H, NCHMe), 4.12–3.94 (m, 4H, NCH_2CH_2N), 3.33–3.30 (m, 1H, A-CH_2Me), 2.77 (br.s, 3H, Me-Mes), 2.63 (br.s, 3H, Me-Mes), 2.53 (br.s, 3H, Me-Mes), 2.42 (br.s, 3H, Me-Mes), 2.33 (br.s, 3H, Me-Mes), 2.24–2.18 (m, 1H, B-CH_2Me), 2.10 (br.s, 3H, Me-Mes), 1.57 (s, 3H, MeN), 1.34 (d, *J* = 6.9 Hz, 3H, MeCH), 0.60 (t, *J* = 7.1 Hz, 3H, MeCH_2N). ^{13}C NMR (150.9 MHz, $CDCl_3$, 23 °C) δ 315.1, 211.8, 149.1, 139.9, 138.7, 138.1, 137.9, 137.7, 136.2, 135.3, 129.7, 129.4, 129.3, 129.2, 128.5, 128.3, 128.0, 126.2, 60.6, 54.3, 52.2 (br.s), 50.8 (br.s), 36.4, 21.1 (br.s, 2C), 20.5 (br.s), 20.5 (br.s), 18.5 (br.s), 18.0 (br.s), 11.7, 9.7.

^1H NMR (500.1 MHz, CD_2Cl_2, 25 °C) δ 18.85 (s, 1H, CH=Ru), 7.59 (t, *J* = 7.6 Hz, 1H, H-4-C_6H_4), 7.23–7.19 (m, 3H, H-3,5-C_6H_4 and CH-Mes), 7.05 (very br.s, 3H, CH-Mes), 6.60 (d, *J* = 7.6 Hz, 1H, H-6-C_6H_4), 5.49 (q, *J* = 6.9 Hz, 1H, NCHMe), 4.10–3.97 (m, 4H, NCH_2CH_2N), 3.32–3.25 (m, 1H, A-CH_2Me), 2.76 (br.s, 3H, Me-Mes), 2.62 (br.s, 3H, Me-Mes), 2.54 (br.s, 3H, Me-Mes), 2.45 (br.s, 3H, Me-Mes), 2.38 (br.s, 3H, Me-Mes), 2.26–2.20 (m, 1H, B-CH_2Me), 2.13 (br.s, 3H, Me-Mes), 1.56 (s, 3H, MeN), 1.38 (d, *J* = 6.9 Hz, 3H, MeCH), 0.61 (t, *J* = 7.1 Hz, 3H, MeCH_2N). ^{13}C NMR (125.8 MHz, CD_2Cl_2, 25 °C) δ 313.6, 211.3, 149.3, 139.9, 138.7, 138.4, 137.7, 136.2, 135.4, 129.6, 129.4, 129.2, 129.1, 128.8, 128.2, 128.2, 125.6, 60.5, 54.2, 52.2, 50.9, 36.2, 31.9, 31.6, 22.6, 20.8, 20.3, 20.1, 19.4, 18.3, 17.8, 11.3, 9.37.

IR spectrum: 2945, 1610, 1598, 1444, 1306, 1257, 1181, 1026, 872, 816 cm^{-1}.

HRMS (ESI-TOF) $[M]^+$ calcd. for $C_{33}H_{43}Cl_2N_3Ru$ 667.2029, found 667.2042.

5.3 [1,3-Bis(2,4,6-trimethylphenyl)imidazolin-2-ylidene](dichloro){2-[(diethylamino)-1-ethyl]benzylidene}ruthenium (4c)

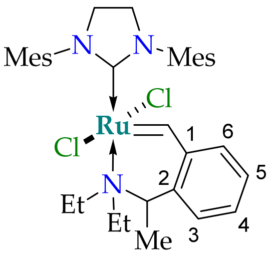

Green powder, 251 mg, 0.38 mmol, 71%, m.p. 203.7–204.1 °C.

^1H NMR (600.2 MHz, $CDCl_3$, 22 °C) δ 18.74 (s, 1H, CH=Ru), 7.46 (t, *J* = 7.8 Hz, 1H, H-4-C_6H_4), 7.19 (d, *J* = 7.8 Hz, 1H, H-3-C_6H_4), 7.15 (br.s, 1H, CH-Mes), 7.09 (t, *J* = 7.8 Hz, 1H, H-5-C_6H_4), 7.04 (br.s, 1H,

CH-Mes), 7.02 (br.s, 1H, CH-Mes), 6.93 (br.s, 1H, CH-Mes), 6.46 (d, *J* = 7.8 Hz, 1H, H-6-C_6H_4), 5.36 (very br.s, 1H, NC<u>H</u>Me), 4.15–3.93 (m, 4H, NCH_2CH_2N), 3.46–3.43 (m, 1H, A-C<u>H</u>$_2$Me), 2.83 (br.s, 3H, Me-Mes), 2.71 (br.s, 3H, Me-Mes), 2.70–2.65 (m, 1H, B-C<u>H</u>$_2$Me), 2.49 (br.s, 3H, Me-Mes), 2.43 (br.s, 3H, Me-Mes), 2.33 (br.s, 3H, Me-Mes), 2.22–2.12 (m, 2H, C<u>H</u>$_2$Me), 2.05 (br.s, 3H, Me-Mes), 1.40 (d, *J* = 6.8 Hz, 3H, <u>Me</u>CH), 0.63 (t, *J* = 7.6 Hz, 3H, <u>Me</u>CH_2N), 0.32 (t, *J* = 7.6 Hz, 3H, <u>Me</u>CH_2N). ^{13}C NMR (150.9 MHz, $CDCl_3$, 22 °C) δ 316.2, 211.9, 149.7, 140.0, 139.7, 138.7, 138.1 (2C), 137.8, 137.73, 137.68, 135.3, 129.7, 129.4, 129.3, 129.2, 128.5, 127.6, 127.3, 124.9 (very br.s), 60.9, 52.1, 51.1, 50.8, 44.6, 21.0, 20.9, 20.6, 20.3, 18.7, 18.0, 13.6 (very br.s), 12.5, 10.6 (br.s).

IR spectrum: 2957, 1610, 1595, 1436, 1306, 1258, 1186, 1027, 862, 810 cm^{-1}.

HRMS (ESI-TOF) $[M-Cl]^+$ calcd. for $C_{34}H_{45}Cl_2N_3Ru$ 631.7569, found 631.7566.

5.4 *[1,3-Bis(2,4,6-trimethylphenyl)imidazolidin-2-ylidene](dichloro)[2-(morpholin-4-ylethyl)benzylidene]ruthenium (4d)*

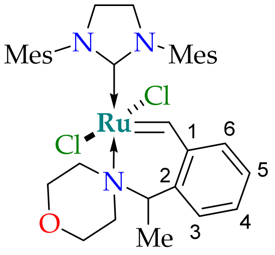

Green powder, 289 mg, 0.42 mmol, 80%, m.p. 204.6–204.9 °C.

^{1}H NMR (600.2 MHz, $CDCl_3$, 25 °C) δ 18.98 (s, 1H, CH=Ru), 7.20 (t, *J* = 7.6 Hz, 1H, H-4-C_6H_4), 7.14 (t, *J* = 7.6 Hz, 1H, H-5-C_6H_4), 7.12–7.11 (m, 4H, CH-3,5-Mes and H-3-C_6H_4), 7.03 (br.s, 1H, CH-Mes), 6.98 (d, *J* = 7.6 Hz, 1H, H-6-C_6H_4), 6.30 (d, *J* = 7.1 Hz, 1H, NC<u>H</u>Me), 4.24–3.94 (m, 4H, NCH_2CH_2N), 3.52 (m, 2H, H-Morph), 3.34 (br.d, *J* ~ 12.6 Hz, 1H, H-Morph), 3.13 (br.d, *J* ~ 10.1 Hz, 1H, H-Morph), 2.68 (br.s, 3H, Me-Mes), 2.77 (br.s, 3H, Me-Mes), 2.55 (m, 1H, H-Morph), 2.41 (s, 3H, Me-Mes), 2.36 (br.s, 6H, 2 × Me-Mes), 2.18 (br.s, 3H, Me-Mes), 2.03–1.97 (m, 1H, H-Morph), 1.60 (br.m, 3H, H-Morph), 0.72 (d, *J* = 7.1 Hz, 3H, NCH<u>Me</u>). ^{13}C NMR (150.9 MHz, $CDCl_3$, 25 °C) δ 324.4, 211.0, 156.7, 140.4, 139.8, 139.6, 138.5, 138.0, 137.7, 137.0, 134.6 (br.s), 133.8, 129.8, 129.52, 129.49, 129.3, 127.9, 127.7 (very br.s), 127.3, 111.0, 71.8 (br.s), 67.3, 65.5, 58.6 (br.s), 53.1 (br.s), 52.2, 50.5, 31.7, 21.3 (br.s), 21.1, 20.9, 21.8, 20.3, 18.8, 18.2.

IR spectrum: 2947, 1615, 1590, 1420, 1305, 1257, 1187, 1033, 869, 817 cm^{-1}.

HRMS (ESI-TOF) $[M-Cl]^+$ calcd. for $C_{34}H_{43}Cl_2N_3ORu$ 645.7361, found 645.7358.

5.5 *[1,3-Bis(2,4,6-trimethylphenyl)imidazolin-2-ylidene](dichloro){2-[(dimethylamino)-1-propyl]benzylidene}ruthenium (5a)*

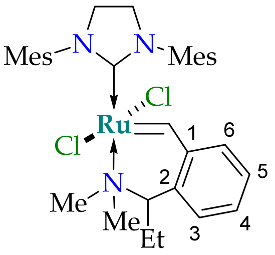

Green powder, 256 mg, 0.39 mmol, 74%, m.p. 202.3–202.7 °C.

^{1}H NMR (600.2 MHz, $CDCl_3$, 25 °C) δ 18.91 (s, 1H, CH=Ru), 7.50 (t, *J* = 7.6 Hz, 1H, H-4-C_6H_4), 7.16–7.03 (m, 6H, H-3-C_6H_4, H-5-C_6H_4, H-Mes), 6.70 (d, *J* = 7.1 Hz, 1H, H-6-C_6H_4), 5.15–5.10 (br.s, 1H, NC<u>H</u>Et), 4.10 (br.s, 4H, NCH_2CH_2N), 2.80–2.30 (m, 15H, Me-Mes), 2.01 (s, 3H, Me-Mes), 1.91-1.87 (m, 2H,

CH_2Me), 1.59-154 (m, 6H, Me_2N), 1.05 (m, 3H, $MeCH_2CH$). ^{13}C NMR (150.9 MHz, CD_2Cl_2, 25 °C) δ 314.2. 213.3, 149.5, 139.7, 138.5, 135.6, 129.4, 129.4, 129.4, 129.3, 129.3, 129.3, 129.2, 128.6, 128.6, 128.3, 128.2, 128.2, 127.7, 126.9, 66.8, 52.2, 51.2, 43.7, 40.5, 21.2, 21.0, 20.9, 18.9, 18.9, 17.2, 17.2, 13.2.

IR spectrum: 2924, 1929, 1606, 1478, 1261, 748, 591, 582 cm^{-1}.

HRMS (ESI-TOF) $[M]^+$ calcd. for $C_{33}H_{43}Cl_2N_3Ru$ 667.2029, found 667.2035.

5.6 [1,3-Bis(2,4,6-trimethylphenyl)imidazolin-2-ylidene](dichloro){2-[(methylethylamino)-1-propyl]benzylidene}ruthenium (5b)

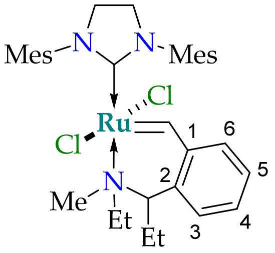

Green powder, 205 mg, 0.31 mmol, 58%, m.p. 202.1–204.5 °C.

^{1}H NMR (600.2 MHz, $CDCl_3$, 25 °C) δ 18.91 (s, 1H, CH=Ru), 7.49 (t, J = 7.6 Hz, 1H, H-4-C_6H_4), 7.15-7.12 (m, 3H, H-3-C_6H_4, H-5-C_6H_4 and H-Mes), 7.05 (br.s, 1H, H-Mes), 7.01 (br.s, 1H, H-Mes), 6.97 (br.s, 1H, H-Mes), 6.52 (d, J = 7.1 Hz, 1H, H-6-C_6H_4), 4.99 (d, J = 10.1 Hz, 1H, NCHEt), 4.10–3.97 (m, 4H, NCH_2CH_2N), 3.25–3.22 (m, 1H, N-A-CH_2Me), 2.73 (br. s, 3H, Me-Mes), 2.58 (br. s, 6H, Me-Mes), 2.44 (br. s, 3H, Me-Mes), 2.34 (br. s, 3H, Me-Mes), 2.23 (br.s, 3H, Me-Mes), 2.30-2.26 (m, 1H, N-B-CH_2Me), 2.00-1.97 (m, 1H, CH-A-CH_2Me), 1.83-1.80 (m, 1H, CH-B-CH_2Me), 1.64 (m, 3H, MeN), 0.97 (m, 3H, $MeCH_2N$), 0.67 (m, 3H, $MeCH_2CH$). ^{13}C NMR (150.9 MHz, CD_2Cl_2, 25 °C) δ 315.8, 211.3, 151.0, 140.0, 139.6, 138.6, 138.1, 137.7, 135.4, 133.7, 129.6, 129.7, 129.4, 129.2, 129.2, 128.8, 128.1, 128.0, 127.8, 126.4, 124.5, 67.6, 54.0, 52.2, 50.8, 38.0, 21.0, 20.3, 18.5, 18.1, 15.7, 12.7, 10.9.

IR spectrum: 2952, 1600, 1590, 1428, 1305, 1255, 1183, 1028, 871, 812 cm^{-1}.

HRMS (ESI-TOF) $[M]^+$ calcd. for $C_{34}H_{45}Cl_2N_3Ru$ 631.7569, found 631.7565.

5.7 [1,3-Bis(2,4,6-trimethylphenyl)imidazolin-2-ylidene](dichloro){2-[(diethylamino)-1-propyl]benzylidene}ruthenium (5c)

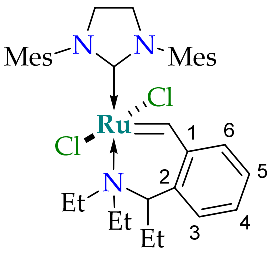

Green powder, 18 mg, 0.03 mmol, 5%.

^{1}H NMR (600.2 MHz, $CDCl_3$, 25 °C) δ 18.82 (s, 1H, CH=Ru), 7.40 (t, J = 7.6 Hz, 1H, H-4-C_6H_4), 7.06 (m, 1H, H-3-C_6H_4), 7.04 (m, 1H, H-5-C_6H_4), 7.02–6.82 (m, 4H, H-3,5-Mes), 6.44 (d, J = 7.1 Hz, H-6-C_6H_4), 4.91 (d, J = 10.1 Hz, 1H, α-CHN), 4.05–3.70 (m, 4H, NCH_2CH_2N), 3.17–3.13 (m, 1H, N-A-CH_2Me), 2.65 (br. s, 3H, Me-4-Mes), 2.51 (m, 1H, N-B-CH_2Me) 2.49 (br. s, 6H, Me-2,4-Mes), 2.35 (br. s, 3H, Me-2-Mes), 2.25 (br. s, 3H, Me-6-Mes), 2.19 (m, 2H, NCH_2Me), 2.15 (br. s, 3H, Me-6-Mes), 1.90 (m, 1H, CH-A-CH_2Me), 1.73 (m, 1H, CH-B-CH_2Me), 1.55 (s, 3H, $MeCH_2N$), 0.89 (t, J = 7.6 Hz, 3H, $MeCH_2N$), 0.58 (t, J = 7.6 Hz, 3H, $MeCH_2CH$). ^{13}C NMR (150.9 MHz, CD_2Cl_2, 25 °C) δ 314.4, 211.5, 151.2, 133.9, 129.8, 129.7, 129.6, 129.6, 129.4, 129.4, 129.4, 129.0, 128.2, 128.2, 128.1, 128.1, 128.0, 127.0, 124.7, 67.8, 67.8, 56.2, 54.1, 52.4, 44.5, 38.2, 21.1, 20.5, 18.7, 18.5, 15.9, 14.2, 12.9, 11.1.

IR spectrum: 2959, 1606,1586, 1408, 1314, 1259, 1190, 1028, 872, 813 cm^{-1}.

HRMS (ESI-TOF) $[M]^+$ calcd. for $C_{35}H_{47}Cl_2N_3Ru$ 681.2185, found 681.2193.

5.8 [1,3-Bis(2,4,6-trimethylphenyl)imidazolidin-2-ylidene](dichloro)[2-(morpholin-4-ylpropyl)benzylidene]ruthenium (5d)

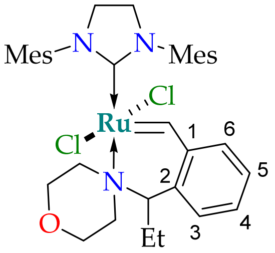

Green powder, 284 mg, 0.41 mmol, 77%, m.p. 204.2–204.6 °C.

^{1}H NMR (600.2 MHz, $CDCl_3$, 25 °C) δ 19.10 (s, 1H, CH=Ru), 7.41 (m, 1H, H-4-C_6H_4), 7.27–7.08 (m, 6H, H-C_6H_4 and CH-Mes), 6.44 (d, *J* = 7.1 Hz, 1H, H-C_6H_4), 4.36–4.09 (m, 5H, NC<u>H</u>Et and NCH_2CH_2N), 3.80–3.66 (m, 2H, H-Morph), 3.46 (d, *J* = 12.6 Hz, 1H, H-Morph), 3.40 (d, *J* = 10.6 Hz, 1H, H-Morph), 3.05 (br.s, 3H, Me-Mes), 2.94 (br.s, 3H, Me-Mes), 2.66–2.55 (m, 1H, H-Morph), 2.56 (s, 3H, Me-Mes), 2.51 (s, 3H, Me-Mes), 2.49 (s, 3H, Me-Mes), 2.32 (s, 3H, Me-Mes), 2.16–2.13 (m, 1H, C<u>H</u>$_2$Me), 1.79–1.72 (m, 1H, H-Morph), 1.64–1.61 (m, 1H, H-Morph), 1.47–1.44 (m, 1H, C<u>H</u>$_2$Me), 0.77–0.74 (m, 1H, H-Morph), 0.34 (t, *J* = 7.3 Hz, 3H, <u>Me</u>CH_2). ^{13}C NMR (150.9 MHz, $CDCl_3$, 25 °C) δ 324.2, 211.0, 156.7, 140.5, 139.7, 139.6, 138.5, 137.9, 137.7, 137.0, 132.1, 131.7, 130.0, 129.5, 129.3, 128.9, 128.1, 127.9, 126.5, 111.5, 79.2 (br.s), 67.5, 65,4, 59.1 (br.s), 53.5 (br.s), 52.1, 50.5, 25.8 (br.s), 21.1, 20.9, 20.8, 20.2, 18.8, 18.2, 10.8.

IR spectrum : 2951, 1601, 1586, 1423, 1304, 1262, 1183, 1025, 871, 820 cm^{-1}.

HRMS (ESI-TOF) $[M\text{-}Cl]^+$ calcd. for $C_{35}H_{45}Cl_2N_3ORu$: 660.2289, found: 620.2297.

6. Activity of 4 and 5 in RCM reactions with N,N-diallyltosylamide

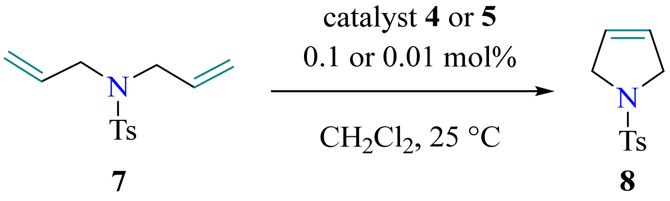

Scheme S5. Ring-closing metathesis reaction (RCM) with N,N-diallyltosylamide

Under an argon atmosphere, the solution of complex **4a**–**d** or **5a**–**d** in dry DCM (5 mL, 0.0008 mmol, 0.1 mol%) was added to a solution of N,N-diallyltosylamide **7** (0.20 g, 0.8 mmol, 1 equiv.) in dry DCM (15 mL). The reaction mixture was stirred at 25 °C for 30 min. During this time, an aliquot (1 mL) was sampled every 2 min and a 10% solution of ethyl vinyl ether in THF (0.2 mL) was added to this aliquot. Solvents were removed under reduced pressure and the **7**/**8** ratio was examined by ^{1}H NMR.

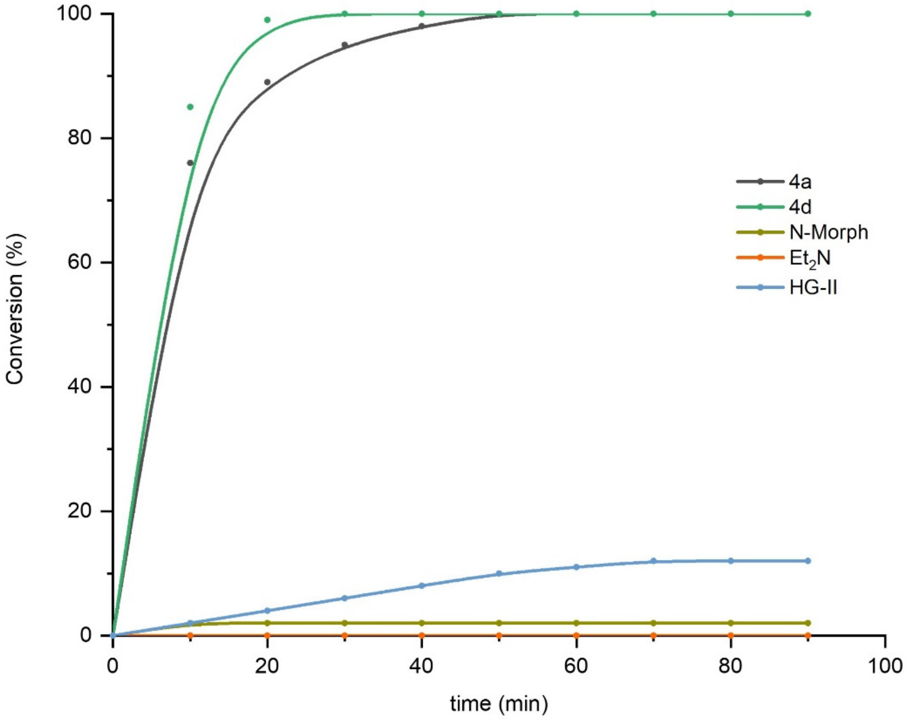

Figure S1. Time/conversion curves for RCM transformations of **7** (0.8 mmol, 200 mg) to **8** under the action of catalysts **4a**, **4d** compared to the previously reported **N-Morph** [1], **NEt2** [1] and **HG-II** [2]. Conditions: catalyst loading is 0.1% mol (0.0008 mmol), 25 °C, Ar, dry CH_2Cl_2 (20 mL).

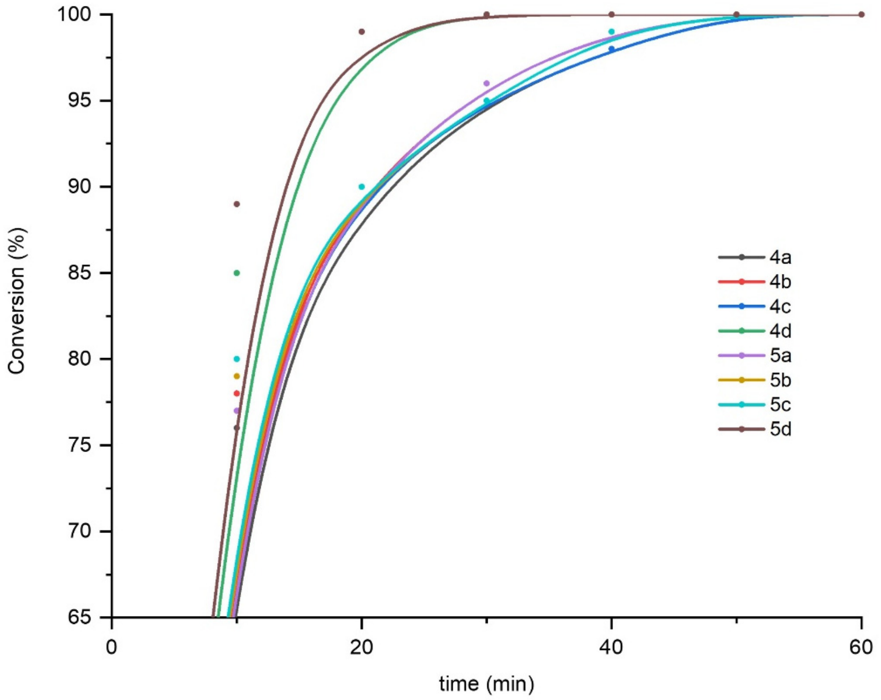

Figure S2. Time/conversion curves for RCM transformations of **7** (0.8 mmol, 200 mg) to **8** under the action of catalysts **4a–d** and **5a–d**. Conditions: catalyst loading is 0.1% mol (0.0008 mmol), 25 °C, Ar, dry CH_2Cl_2 (20 mL). A fragment of the kinetic curve is presented to better reflect the course of reactions with various catalysts.

To study the efficiency of the catalysts, their concentration was reduced to 0.01 mol%. A solution of complex **4a–d** or **5a–d** in dry DCM (0.00008 mmol, 0.01 mol%, 5 mL) was added to a solution of N,N-diallyltosylamide **7** (0.20 g, 0.8 mmol, 1 equiv.) in dry DCM (15 mL) under an argon atmosphere. The reaction mixture was stirred at 25 °C for 30 min. During this time, an aliquot (1 mL) was sampled every 2 min and a 10% solution of ethyl vinyl ether in THF (0.2 mL) was added to this aliquot. Solvents were removed under reduced pressure and the **7**/**8** ratio was examined by ^{1}H NMR.

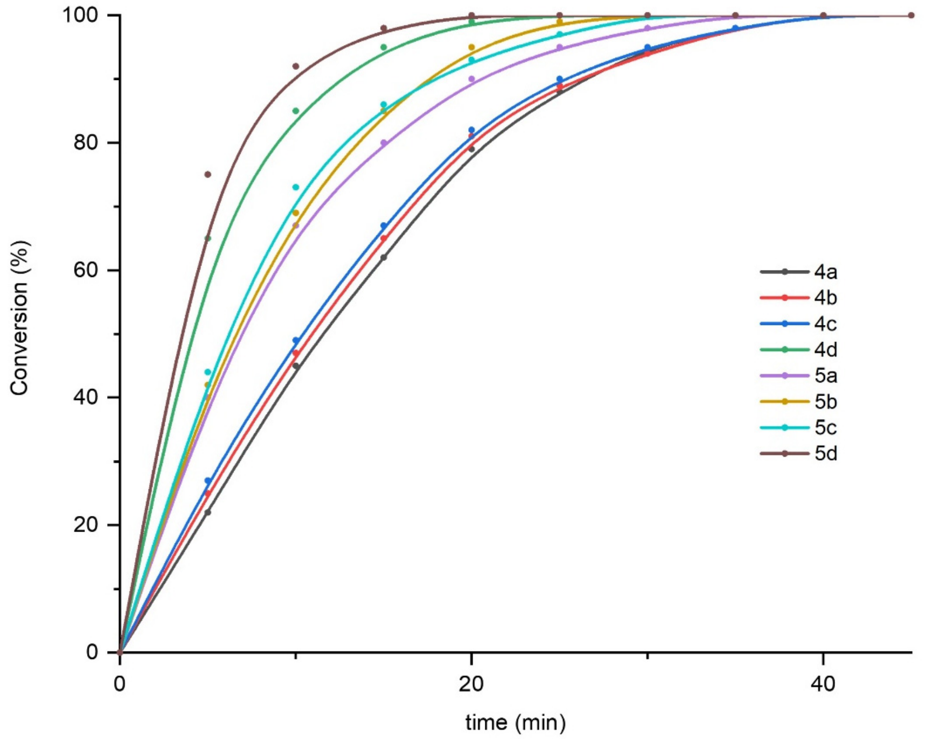

Figure S3. Time/conversion curves for RCM transformations of **7** (0.8 mmol, 200 mg) to **8** under the action of catalysts **4a–d** and **5a–d**. Conditions: catalyst loading is 0.01% mol (0.00008 mmol), 25 °C, Ar, dry CH_2Cl_2 (20 mL).

7. *Polymerization of norborn-5-ene-2,3-dicarboxylic acid dibutyl ester (DBNB) catalyzed by complexes 4 and 5*

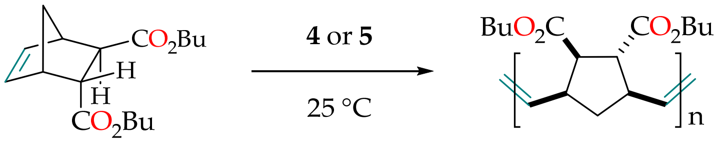

9 (DBNB) **10 (polyDBNB)**

Scheme S6. ROMP polymerization of DBNB promoted by **4a**, **4d**, **5a**, **5d** catalysts.

A solution of complex **4** or **5** in dry DCM (2 mL, 0.008 mmol, 0.1 mol%) was added to thermostated (25 °C) and stirred (1000 rpm) **DBNB** (10 g, 0.05 mol, 1 equiv.). The temperature changes were measured and fixed every 5 seconds until a plateau was reached. As a result, solidification of the liquid substance was observed.

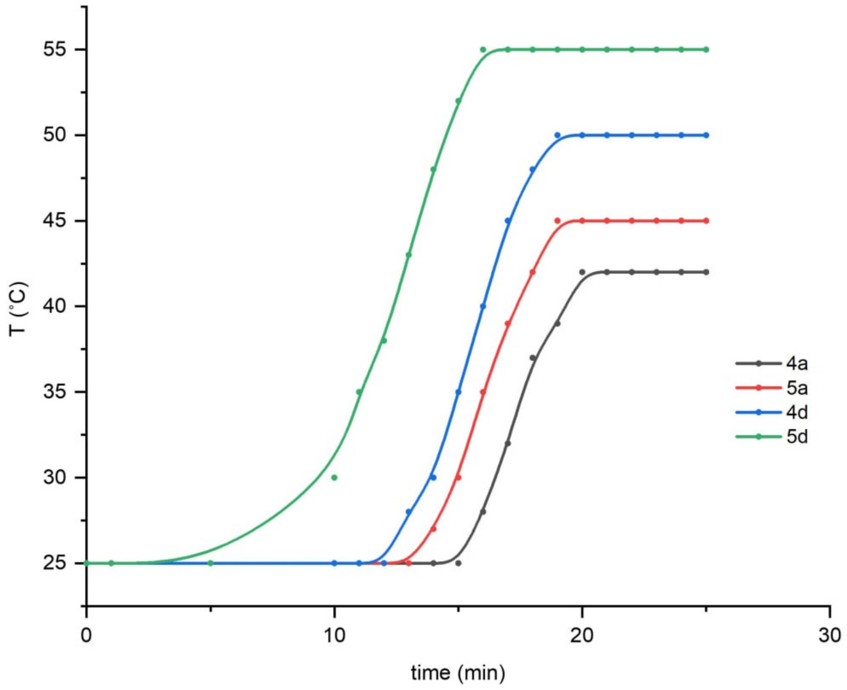

Figure S4. Isotherms of ROMP reactions of **DBNB** (10 g, 0.05 mol) under the action of catalysts **4a**, **4d**, **5a**, **5d**. Reaction conditions: catalyst loading is 0.1 % mol (0.008 mmol), starting temperature is 25 °C, neat.

8. *X-ray analysis*

The crystal structure of all synthesized substances was determined by X-ray structural analysis using an automatic four-circle area-detector diffractometer Bruker KAPPA APEX II with MoKα radiation. The cell parameters were refined over the entire data set, together with data reduction using SAINT-Plus software [3]. Absorption corrections were introduced using the SADABS program [4]. The structures were solved using the SHELXT-2018/2 program [5] and refined by full-matrix least squares on F^2 in the anisotropic approximation for all non-hydrogen atoms [6]. The H atoms were placed in geometrically calculated positions with isotropic temperature factors equal to 1.2U_{eq}(C) for CH_2 and CH-groups, and 1.5U_{eq}(C) for CH_3-groups, the orientation of CH_3-groups was refined. In structures **4a**, **4b**, **5a** solvent molecules were strongly disordered and therefore it was removed using the SQUEEZE routine of PLATON [7], and the structure was then refined again using the data generated. Structure **5b** was refined as an inversion twin. Tables and figures for the structures were generated using Olex2 [8].

Table S2. Crystal data and structure refinement for structures **4a–4c**, **5a**, **5b**, **5d**

Identification Code	4a	4b	4c	5a	5b	5d
CCDC number	2232047	2232048	2232049	2232050	2232051	2232052
Empirical formula	$C_{32}H_{41}Cl_2N_3Ru$	$C_{36}H_{50}N_3Cl_2Ru$	$C_{37}H_{52}N_3Cl_2Ru$	$C_{33}H_{43}Cl_2N_3Ru$	$C_{34}H_{45}N_3Cl_2Ru$	$C_{35}H_{45}Cl_2N_3ORu$
Formula weight	689.74	696.76	710.78	703.77	667.70	695.71
Temperature/K	296(2)	120(2)	120(2)	100(2)	100(2)	100(2)
Crystal system	monoclinic	monoclinic	monoclinic	monoclinic	orthorhombic	triclinic
Space group	C2/c	*C*2/*c*	*C*2/*c*	C2/c	*Pna*2_1	P-1
a/Å	36.4041(11)	35.864(4)	35.9118(15)	36.3345(13)	12.6799(9)	10.800(3)
b/Å	10.5974(3)	10.6637(11)	10.8652(5)	10.4776(4)	23.5558(17)	14.366(4)
c/Å	24.3725(7)	24.284(3)	24.4406(10)	24.3610(9)	10.6250(8)	22.335(5)

α/°	90	90	90	90	90	106.923(11)
β/°	131.974(1)	132.371(3)	132.480(1)	131.192(1)	90	91.005(10)
γ/°	90	90	90	90	90	90.807(11)
Volume/Å^3	6990.4(4)	6861.4(14)	7033.3(5)	6978.9(5)	3173.5(4)	3314.1(15)
Z	8	8	8	8	4	4
ρ_{calc}g/cm^3	1.311	1.349	1.343	1.340	1.397	1.394
μ/mm^{-1}	0.629	0.641	0.627	0.631	0.690	0.666
F(000)	2888.0	2920.0	2984.0	2952.0	1392.0	1448.0
Crystal size/mm^3	0.4 × 0.34 × 0.28	0.33 × 0.24 × 0.17	0.28 × 0.24 × 0.18	0.22 × 0.18 × 0.05	0.28 × 0.18 × 0.12	0.18 × 0.1 × 0.04
Radiation	MoKα (λ = 0.71073)	MoKα (λ = 0.71073)	MoKα (λ = 0.71073)	MoKα (λ = 0.71073)	MoKα (λ = 0.71073)	MoKα (λ = 0.71073)
2Θ range for data collection/°	8.26 to 60	3.074 to 54.996	3.076 to 59.998	8.332 to 59.998	8.248 to 59.998	8.168 to 54.998
Index ranges	-51 ≤ h ≤ 51, -14 ≤ k ≤ 14, -34 ≤ l ≤ 34	-46 ≤ h ≤ 46, -13 ≤ k ≤ 13, -31 ≤ l ≤ 31	-50 ≤ h ≤ 50, -15 ≤ k ≤ 15, -34 ≤ l ≤ 34	-50 ≤ h ≤ 50, -14 ≤ k ≤ 14, -34 ≤ l ≤ 33	-17 ≤ h ≤ 17, -33 ≤ k ≤ 33, -14 ≤ l ≤ 14	-14 ≤ h ≤ 14, -18 ≤ k ≤ 17, -28 ≤ l ≤ 29
Reflections collected	84467	30647	44508	71399	55496	53770
Independent reflections	10164 [R_{int} = 0.0368, R_{sigma} = 0.0238]	7880 [R_{int} = 0.0463, R_{sigma} = 0.0427]	10266 [R_{int} = 0.0455, R_{sigma} = 0.0414]	10147 [R_{int} = 0.0513, R_{sigma} = 0.0382]	8994 [R_{int} = 0.0478, R_{sigma} = 0.0414]	15147 [R_{int} = 0.1964, R_{sigma} = 0.2384]
Data/restraints/parameters	10164/0/349	7880/0/358	10266/2/398	10147/0/358	8994/1/367	15147/0/770
Goodness-of-fit on F^2	1.011	1.014	1.044	1.034	1.023	1.039
Final R indexes [I >= 2σ (I)]	R_1 = 0.0275, wR_2 = 0.0690	R_1 = 0.0316, wR_2 = 0.0710	R_1 = 0.0450, wR_2 = 0.1092	R_1 = 0.0295, wR_2 = 0.0647	R_1 = 0.0275, wR_2 = 0.0520	R_1 = 0.1046, wR_2 = 0.2474
Final R indexes [all data]	R_1 = 0.0441, wR_2 = 0.0775	R_1 = 0.0487, wR_2 = 0.0777	R_1 = 0.0717, wR_2 = 0.1223	R_1 = 0.0427, wR_2 = 0.0695	R_1 = 0.0343, wR_2 = 0.0543	R_1 = 0.2219, wR_2 = 0.3116
Largest diff. peak/hole/e Å^{-3}	0.47/-0.34	0.52/-0.34	1.03/-0.91	0.50/-0.39	0.40/-0.33	2.43/-1.89
Flack parameter	–	–	–	–	0.002(12)	–

Table S3. Bond Lengths for **4a**

Atom	Atom	Length/Å	Atom	Atom	Length/Å
Ru1	Cl2	2.3403(4)	C14	C18	1.509(3)
Ru1	Cl1	2.3464(4)	C15	C16	1.389(3)
Ru1	N2	2.2672(14)	C16	C19	1.494(3)
Ru1	C2	2.0407(15)	C21	C22	1.392(3)
Ru1	C38	1.8227(16)	C21	C26	1.396(3)
N1	C2	1.345(2)	C22	C23	1.386(3)
N1	C5	1.476(2)	C22	C27	1.504(3)
N1	C11	1.431(2)	C23	C24	1.374(4)
N2	C31	1.504(2)	C24	C25	1.364(4)
N2	C40	1.477(2)	C24	C28	1.527(4)

N2	C41	1.483(2)	C25	C26	1.390(3)
N3	C2	1.3491(19)	C26	C29	1.501(3)
N3	C4	1.471(2)	C31	C32	1.511(2)
N3	C21	1.437(2)	C31	C39	1.538(2)
C4	C5	1.504(3)	C32	C33	1.388(2)
C11	C12	1.391(2)	C32	C37	1.414(2)
C11	C16	1.396(2)	C33	C34	1.374(3)
C12	C13	1.390(3)	C34	C35	1.362(3)
C12	C17	1.503(3)	C35	C36	1.385(3)
C13	C14	1.397(4)	C36	C37	1.402(2)
C14	C15	1.374(3)	C37	C38	1.454(2)

Table S4. Bond Angles for **4a**

**Atom**	**Atom**	**Atom**	**Angle/˚**	**Atom**	**Atom**	**Atom**	**Angle/˚**
Cl2	Ru1	Cl1	157.83(2)	C15	C14	C13	118.05(19)
N2	Ru1	Cl2	88.13(4)	C15	C14	C18	121.6(3)
N2	Ru1	Cl1	89.27(4)	C14	C15	C16	122.6(2)
C2	Ru1	Cl2	94.24(4)	C11	C16	C19	121.29(16)
C2	Ru1	Cl1	85.54(4)	C15	C16	C11	117.69(18)
C2	Ru1	N2	171.70(6)	C15	C16	C19	121.02(18)
C38	Ru1	Cl2	100.13(5)	C22	C21	N3	118.49(17)
C38	Ru1	Cl1	101.76(5)	C22	C21	C26	121.34(17)
C38	Ru1	N2	87.60(6)	C26	C21	N3	119.92(17)
C38	Ru1	C2	99.78(7)	C21	C22	C27	122.26(18)
C2	N1	C5	113.91(13)	C23	C22	C21	117.6(2)
C2	N1	C11	128.19(13)	C23	C22	C27	120.0(2)
C11	N1	C5	117.51(13)	C24	C23	C22	122.0(2)
C31	N2	Ru1	108.44(9)	C23	C24	C28	119.9(3)
C40	N2	Ru1	101.20(11)	C25	C24	C23	119.0(2)
C40	N2	C31	111.83(14)	C25	C24	C28	121.1(3)
C40	N2	C41	108.02(15)	C24	C25	C26	121.9(2)
C41	N2	Ru1	116.82(11)	C21	C26	C29	122.38(18)
C41	N2	C31	110.26(14)	C25	C26	C21	117.8(2)
C2	N3	C4	114.23(13)	C25	C26	C29	119.8(2)
C2	N3	C21	126.13(14)	N2	C31	C32	109.69(13)
C21	N3	C4	119.17(13)	N2	C31	C39	114.29(15)
N1	C2	Ru1	132.19(11)	C32	C31	C39	114.01(16)
N1	C2	N3	106.28(13)	C33	C32	C31	121.29(17)
N3	C2	Ru1	120.93(11)	C33	C32	C37	117.72(17)
N3	C4	C5	102.61(13)	C37	C32	C31	120.94(14)

N1	C5	C4	102.92(14)	C34	C33	C32	122.3(2)
C12	C11	N1	118.74(15)	C35	C34	C33	120.51(19)
C12	C11	C16	121.84(16)	C34	C35	C36	119.2(2)
C16	C11	N1	119.37(15)	C35	C36	C37	121.43(19)
C11	C12	C17	121.12(16)	C32	C37	C38	125.65(15)
C13	C12	C11	117.98(18)	C36	C37	C32	118.83(15)
C13	C12	C17	120.84(18)	C36	C37	C38	115.52(16)
C12	C13	C14	121.8(2)	C37	C38	Ru1	130.36(13)
C13	C14	C18	120.3(2)				

Table S5. Hydrogen Bonds for **4a**

**D**	**H**	**A**	**d(D-H)/Å**	**d(H-A)/Å**	**d(D-A)/Å**	**D-H-A/°**
C31	H31A	Cl1	0.98	2.47	3.2563(18)	136.8
C40	H40A	Cl1	0.96	2.71	3.375(2)	127.0
C41	H41B	Cl2	0.96	2.55	3.141(2)	119.8

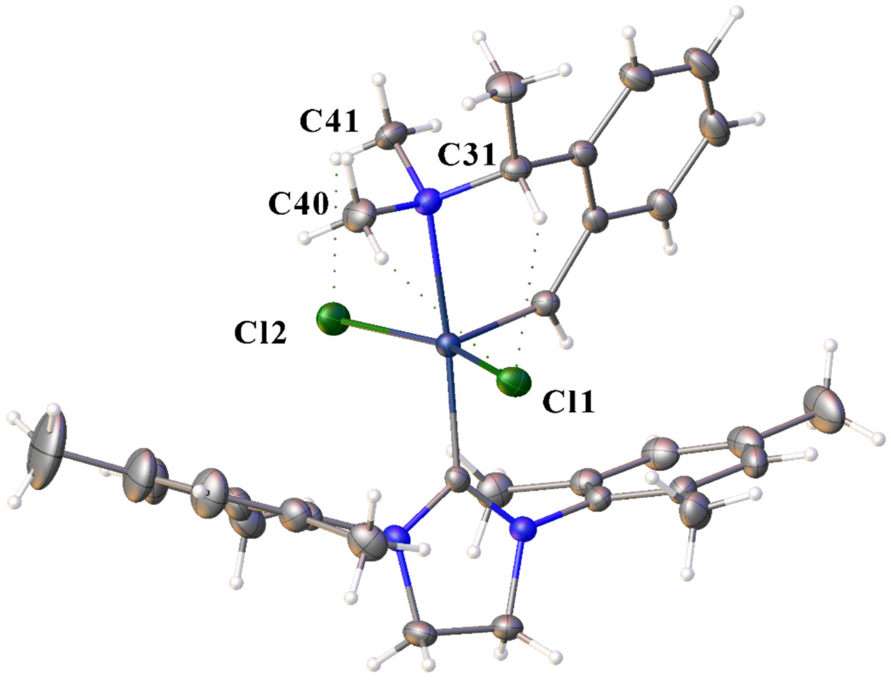

Figure S5. View showing intramolecular hydrogen bonds in **4a**.

Table S6. Torsion Angles for **4a**

**A**	**B**	**C**	**D**	**Angle/˚**	**A**	**B**	**C**	**D**	**Angle/˚**
Ru1	N2	C31	C32	70.28(14)	C14	C15	C16	C11	-0.8(3)
Ru1	N2	C31	C39	-160.25(13)	C14	C15	C16	C19	179.0(2)
Cl2	Ru1	C38	C37	102.98(15)	C16	C11	C12	C13	-1.9(3)
Cl1	Ru1	C38	C37	-73.44(15)	C16	C11	C12	C17	-179.11(17)

N1	C11	C12	C13	-179.28(15)	C17	C12	C13	C14	178.05(19)
N1	C11	C12	C17	3.5(2)	C18	C14	C15	C16	178.5(2)
N1	C11	C16	C15	179.26(16)	C21	N3	C2	Ru1	16.9(2)
N1	C11	C16	C19	-0.6(2)	C21	N3	C2	N1	-170.91(15)
N2	Ru1	C38	C37	15.31(15)	C21	N3	C4	C5	170.44(16)
N2	C31	C32	C33	136.13(16)	C21	C22	C23	C24	-3.5(4)
N2	C31	C32	C37	-46.4(2)	C22	C21	C26	C25	-5.8(3)
N3	C4	C5	N1	2.19(19)	C22	C21	C26	C29	170.24(19)
N3	C21	C22	C23	-178.92(19)	C22	C23	C24	C25	-0.9(5)
N3	C21	C22	C27	4.6(3)	C22	C23	C24	C28	179.5(4)
N3	C21	C26	C25	-179.9(2)	C23	C24	C25	C26	2.1(5)
N3	C21	C26	C29	-3.8(3)	C24	C25	C26	C21	1.2(4)
C2	Ru1	C38	C37	-160.88(15)	C24	C25	C26	C29	-175.0(3)
C2	N1	C5	C4	-1.8(2)	C26	C21	C22	C23	7.0(3)
C2	N1	C11	C12	-84.0(2)	C26	C21	C22	C27	-169.50(19)
C2	N1	C11	C16	98.6(2)	C27	C22	C23	C24	173.0(3)
C2	N3	C4	C5	-2.2(2)	C28	C24	C25	C26	-178.4(4)
C2	N3	C21	C22	83.2(2)	C31	C32	C33	C34	176.55(18)
C2	N3	C21	C26	-102.6(2)	C31	C32	C37	C36	-176.59(15)
C4	N3	C2	Ru1	-171.06(12)	C31	C32	C37	C38	2.5(2)
C4	N3	C2	N1	1.10(19)	C32	C33	C34	C35	0.6(3)
C4	N3	C21	C22	-88.4(2)	C32	C37	C38	Ru1	7.0(2)
C4	N3	C21	C26	85.8(2)	C33	C32	C37	C36	0.9(2)
C5	N1	C2	Ru1	171.45(12)	C33	C32	C37	C38	180.00(16)
C5	N1	C2	N3	0.54(18)	C33	C34	C35	C36	-0.2(3)
C5	N1	C11	C12	88.43(19)	C34	C35	C36	C37	0.2(3)
C5	N1	C11	C16	-89.04(19)	C35	C36	C37	C32	-0.6(3)
C11	N1	C2	Ru1	-15.9(2)	C35	C36	C37	C38	-179.76(16)
C11	N1	C2	N3	173.15(15)	C36	C37	C38	Ru1	-173.85(13)
C11	N1	C5	C4	-175.29(15)	C37	C32	C33	C34	-0.9(3)
C11	C12	C13	C14	0.8(3)	C39	C31	C32	C33	6.5(2)
C12	C11	C16	C15	1.9(3)	C39	C31	C32	C37	-176.07(16)
C12	C11	C16	C19	-177.97(17)	C40	N2	C31	C32	-178.99(14)
C12	C13	C14	C15	0.2(3)	C40	N2	C31	C39	-49.5(2)
C12	C13	C14	C18	-178.5(2)	C41	N2	C31	C32	-58.78(17)
C13	C14	C15	C16	-0.2(3)	C41	N2	C31	C39	70.7(2)

Table S7. Bond Lengths for **4b**

Atom	Atom	Length/Å	Atom	Atom	Length/Å
Ru1	Cl1	2.3540(6)	C15	C16	1.395(3)

Ru1	Cl2	2.3608(6)	C16	C19	1.505(3)
Ru1	N2	2.2629(18)	C21	C22	1.405(3)
Ru1	C2	2.048(2)	C21	C26	1.398(3)
Ru1	C38	1.822(2)	C22	C23	1.390(3)
N1	C2	1.350(3)	C22	C27	1.508(3)
N1	C5	1.475(3)	C23	C24	1.385(4)
N1	C11	1.434(3)	C24	C25	1.389(4)
N2	C31	1.519(3)	C24	C28	1.508(3)
N2	C40	1.481(3)	C25	C26	1.392(3)
N2	C41	1.497(3)	C26	C29	1.499(3)
N3	C2	1.356(3)	C31	C32	1.516(3)
N3	C4	1.476(3)	C31	C39	1.527(3)
N3	C21	1.437(3)	C32	C33	1.394(3)
C4	C5	1.522(3)	C32	C37	1.418(3)
C11	C12	1.400(3)	C33	C34	1.394(3)
C11	C16	1.396(3)	C34	C35	1.376(4)
C12	C13	1.394(3)	C35	C36	1.391(3)
C12	C17	1.501(3)	C36	C37	1.407(3)
C13	C14	1.384(4)	C37	C38	1.459(3)
C14	C15	1.398(3)	C41	C42	1.523(3)
C14	C18	1.506(3)			

Table S8. Bond Angles for **4b**

**Atom**	**Atom**	**Atom**	**Angle/°**	**Atom**	**Atom**	**Atom**	**Angle/°**
Cl1	Ru1	Cl2	161.09(2)	C13	C14	C18	120.9(2)
N2	Ru1	Cl1	88.18(5)	C15	C14	C18	120.8(2)
N2	Ru1	Cl2	90.27(5)	C16	C15	C14	121.8(2)
C2	Ru1	Cl1	85.90(6)	C11	C16	C19	120.9(2)
C2	Ru1	Cl2	93.50(6)	C15	C16	C11	117.9(2)
C2	Ru1	N2	171.80(7)	C15	C16	C19	121.2(2)
C38	Ru1	Cl1	103.54(7)	C22	C21	N3	119.5(2)
C38	Ru1	Cl2	95.26(7)	C26	C21	N3	118.8(2)
C38	Ru1	N2	88.66(8)	C26	C21	C22	121.3(2)
C38	Ru1	C2	98.24(9)	C21	C22	C27	121.9(2)
C2	N1	C5	114.40(17)	C23	C22	C21	118.1(2)
C2	N1	C11	128.47(18)	C23	C22	C27	120.0(2)
C11	N1	C5	117.00(17)	C24	C23	C22	122.0(2)
C31	N2	Ru1	107.97(13)	C23	C24	C25	118.3(2)
C40	N2	Ru1	115.74(14)	C23	C24	C28	121.0(3)
C40	N2	C31	109.69(18)	C25	C24	C28	120.6(3)

C40	N2	C41	108.59(18)	C24	C25	C26	122.2(2)
C41	N2	Ru1	104.66(13)	C21	C26	C29	122.6(2)
C41	N2	C31	110.04(18)	C25	C26	C21	117.8(2)
C2	N3	C4	114.44(17)	C25	C26	C29	119.5(2)
C2	N3	C21	126.11(18)	N2	C31	C39	114.67(19)
C21	N3	C4	118.51(17)	C32	C31	N2	110.12(18)
N1	C2	Ru1	132.29(16)	C32	C31	C39	113.81(19)
N1	C2	N3	106.08(18)	C33	C32	C31	121.0(2)
N3	C2	Ru1	120.96(15)	C33	C32	C37	118.1(2)
N3	C4	C5	102.35(17)	C37	C32	C31	120.8(2)
N1	C5	C4	102.73(17)	C32	C33	C34	121.9(2)
C12	C11	N1	119.2(2)	C35	C34	C33	120.3(2)
C16	C11	N1	118.74(19)	C34	C35	C36	119.1(2)
C16	C11	C12	122.0(2)	C35	C36	C37	121.6(2)
C11	C12	C17	120.8(2)	C32	C37	C38	125.2(2)
C13	C12	C11	117.8(2)	C36	C37	C32	119.0(2)
C13	C12	C17	121.5(2)	C36	C37	C38	115.8(2)
C14	C13	C12	122.2(2)	C37	C38	Ru1	130.49(17)
C13	C14	C15	118.3(2)	N2	C41	C42	113.2(2)

Table S9. Hydrogen Bonds for **4b**

**D**	**H**	**A**	**d(D-H)/Å**	**d(H-A)/Å**	**d(D-A)/Å**	**D-H-A/°**
C4	H4A	Cl2[1]	0.99	2.74	3.527(2)	136.6
C5	H5B	Cl2[1]	0.99	2.82	3.567(2)	133.1
C31	H31A	Cl1	1.00	2.48	3.266(2)	135.6
C40	H40B	Cl2	0.98	2.63	3.164(3)	114.1
C41	H41B	Cl1	0.99	2.81	3.342(2)	114.6

[1]1/2-X,-1/2+Y,1/2-Z

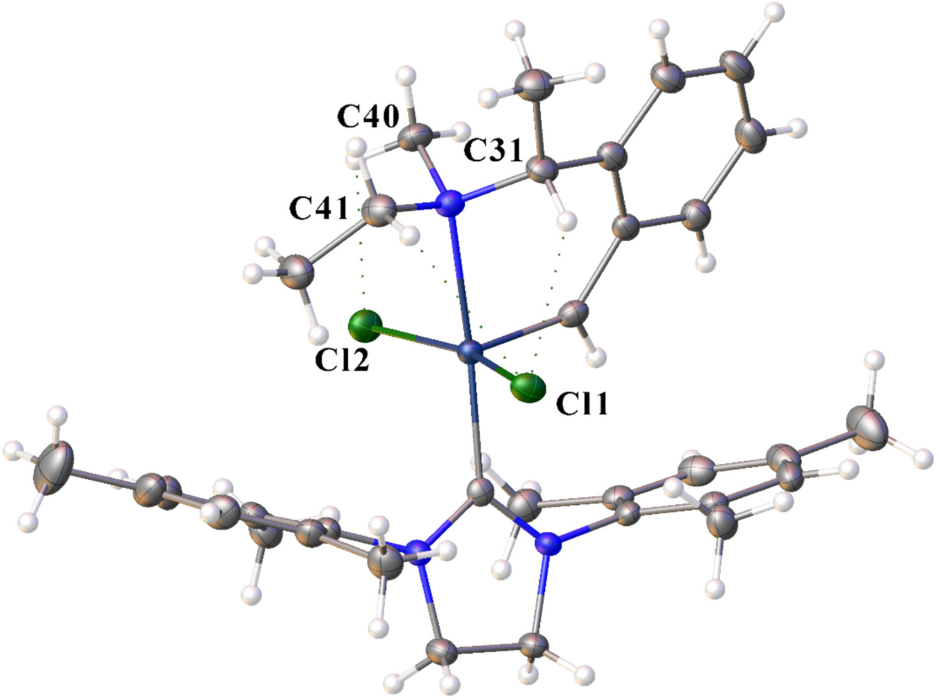

Figure S6. View showing intramolecular hydrogen bonds in **4b**.

Table S10. Torsion Angles for **4b**

A	B	C	D	Angle/°	A	B	C	D	Angle/°
Ru1	N2	C31	C32	69.84(19)	C14	C15	C16	C19	177.2(2)
Ru1	N2	C31	C39	-160.25(16)	C16	C11	C12	C13	1.3(3)
Ru1	N2	C41	C42	-45.9(2)	C16	C11	C12	C17	-179.3(2)
Cl1	Ru1	C38	C37	-74.2(2)	C17	C12	C13	C14	-180.0(2)
Cl2	Ru1	C38	C37	103.7(2)	C18	C14	C15	C16	-178.6(2)
N1	C11	C12	C13	178.36(19)	C21	N3	C2	Ru1	19.0(3)
N1	C11	C12	C17	-2.2(3)	C21	N3	C2	N1	-169.2(2)
N1	C11	C16	C15	-178.18(19)	C21	N3	C4	C5	169.95(19)
N1	C11	C16	C19	4.7(3)	C21	C22	C23	C24	0.8(4)
N2	Ru1	C38	C37	13.6(2)	C22	C21	C26	C25	7.3(3)
N2	C31	C32	C33	135.5(2)	C22	C21	C26	C29	-167.9(2)
N2	C31	C32	C37	-48.2(3)	C22	C23	C24	C25	2.6(4)
N3	C4	C5	N1	0.0(2)	C22	C23	C24	C28	179.8(3)
N3	C21	C22	C23	-179.2(2)	C23	C24	C25	C26	-1.1(4)
N3	C21	C22	C27	-1.8(3)	C24	C25	C26	C21	-3.8(4)
N3	C21	C26	C25	-179.4(2)	C24	C25	C26	C29	171.6(2)
N3	C21	C26	C29	5.5(3)	C26	C21	C22	C23	-5.8(3)
C2	Ru1	C38	C37	-162.0(2)	C26	C21	C22	C27	171.5(2)
C2	N1	C5	C4	-0.3(2)	C27	C22	C23	C24	-176.6(2)
C2	N1	C11	C12	96.8(3)	C28	C24	C25	C26	-178.3(3)

C2	N1	C11	C16	-86.0(3)	C31	N2	C41	C42	-161.7(2)
C2	N3	C4	C5	0.4(3)	C31	C32	C33	C34	175.8(2)
C2	N3	C21	C22	-107.9(3)	C31	C32	C37	C36	-175.7(2)
C2	N3	C21	C26	78.6(3)	C31	C32	C37	C38	4.0(3)
C4	N3	C2	Ru1	-172.31(15)	C32	C33	C34	C35	0.6(4)
C4	N3	C2	N1	-0.6(3)	C32	C37	C38	Ru1	7.8(3)
C4	N3	C21	C22	83.9(3)	C33	C32	C37	C36	0.7(3)
C4	N3	C21	C26	-89.6(3)	C33	C32	C37	C38	-179.6(2)
C5	N1	C2	Ru1	170.97(17)	C33	C34	C35	C36	-0.5(4)
C5	N1	C2	N3	0.5(2)	C34	C35	C36	C37	0.6(4)
C5	N1	C11	C12	-87.5(2)	C35	C36	C37	C32	-0.7(3)
C5	N1	C11	C16	89.6(2)	C35	C36	C37	C38	179.6(2)
C11	N1	C2	Ru1	-13.3(3)	C36	C37	C38	Ru1	-172.41(17)
C11	N1	C2	N3	176.3(2)	C37	C32	C33	C34	-0.7(3)
C11	N1	C5	C4	-176.55(19)	C39	C31	C32	C33	5.1(3)
C11	C12	C13	C14	-0.5(3)	C39	C31	C32	C37	-178.6(2)
C12	C11	C16	C15	-1.1(3)	C40	N2	C31	C32	-57.1(2)
C12	C11	C16	C19	-178.2(2)	C40	N2	C31	C39	72.8(2)
C12	C13	C14	C15	-0.4(4)	C40	N2	C41	C42	78.3(2)
C12	C13	C14	C18	178.8(2)	C41	N2	C31	C32	-176.49(18)
C13	C14	C15	C16	0.6(4)	C41	N2	C31	C39	-46.6(3)
C14	C15	C16	C11	0.1(3)					

Table S11. Bond Lengths for **4c**

Atom	Atom	Length/Å	Atom	Atom	Length/Å
Ru1	Cl1	2.3509(7)	C21	C22	1.392(4)
Ru1	Cl2	2.3654(7)	C21	C26	1.408(4)
Ru1	N2	2.265(2)	C22	C23	1.398(4)
Ru1	C2	2.042(3)	C22	C27	1.507(5)
Ru1	C38	1.823(3)	C23	C24	1.384(5)
N1	C2	1.348(3)	C24	C25	1.382(6)
N1	C5	1.483(4)	C24	C28	1.515(5)
N1	C11	1.430(3)	C25	C26	1.394(5)
N2	C31	1.516(4)	C26	C29	1.498(5)
N2	C40	1.502(4)	C31	C32	1.525(4)
N2	C40A	1.502(4)	C31	C39	1.535(5)
N2	C42	1.495(4)	C32	C33	1.395(4)
N3	C2	1.364(3)	C32	C37	1.421(4)
N3	C4	1.472(4)	C33	C34	1.380(5)
N3	C21	1.440(4)	C34	C35	1.379(5)

C4	C5	1.515(4)	C35	C36	1.389(4)
C11	C12	1.397(4)	C36	C37	1.400(4)
C11	C16	1.400(4)	C37	C38	1.457(4)
C12	C13	1.390(4)	C40	C41	1.359(8)
C12	C17	1.500(4)	C40A	C41A	1.300(14)
C13	C14	1.392(5)	C42	C43	1.510(5)
C14	C15	1.385(5)	C1S	C2S	1.542(10)
C14	C18	1.517(5)	C2S	C3S	1.500(11)
C15	C16	1.394(4)	C3S	C3S[1]	1.546(18)
C16	C19	1.507(4)			

[1]-X,+Y,3/2-Z

Table S12. Bond Angles for **4c**

**Atom**	**Atom**	**Atom**	**Angle/˚**	**Atom**	**Atom**	**Atom**	**Angle/˚**
Cl1	Ru1	Cl2	161.36(3)	C15	C14	C18	121.4(3)
N2	Ru1	Cl1	88.32(7)	C14	C15	C16	121.9(3)
N2	Ru1	Cl2	90.71(7)	C11	C16	C19	120.3(3)
C2	Ru1	Cl1	86.22(7)	C15	C16	C11	117.9(3)
C2	Ru1	Cl2	92.58(7)	C15	C16	C19	121.7(3)
C2	Ru1	N2	171.97(10)	C22	C21	N3	119.7(3)
C38	Ru1	Cl1	103.20(9)	C22	C21	C26	122.0(3)
C38	Ru1	Cl2	95.38(9)	C26	C21	N3	117.9(3)
C38	Ru1	N2	88.69(11)	C21	C22	C23	118.0(3)
C38	Ru1	C2	98.29(11)	C21	C22	C27	122.5(3)
C2	N1	C5	114.1(2)	C23	C22	C27	119.4(3)
C2	N1	C11	128.9(2)	C24	C23	C22	121.5(4)
C11	N1	C5	116.9(2)	C23	C24	C28	120.8(4)
C31	N2	Ru1	107.95(17)	C25	C24	C23	118.8(3)
C40	N2	Ru1	114.6(2)	C25	C24	C28	120.4(4)
C40	N2	C31	110.9(2)	C24	C25	C26	122.5(3)
C40A	N2	Ru1	114.6(2)	C21	C26	C29	122.6(3)
C40A	N2	C31	110.9(2)	C25	C26	C21	116.9(3)
C42	N2	Ru1	104.93(18)	C25	C26	C29	120.4(3)
C42	N2	C31	109.9(2)	N2	C31	C32	110.1(2)
C42	N2	C40	108.4(2)	N2	C31	C39	116.1(3)
C42	N2	C40A	108.4(2)	C32	C31	C39	113.7(3)
C2	N3	C4	114.2(2)	C33	C32	C31	120.9(3)
C2	N3	C21	126.8(2)	C33	C32	C37	117.9(3)
C21	N3	C4	118.4(2)	C37	C32	C31	121.1(3)
N1	C2	Ru1	132.31(19)	C34	C33	C32	122.2(3)

N1	C2	N3	106.2(2)	C35	C34	C33	120.3(3)
N3	C2	Ru1	121.07(19)	C34	C35	C36	119.0(3)
N3	C4	C5	102.7(2)	C35	C36	C37	121.9(3)
N1	C5	C4	102.8(2)	C32	C37	C38	125.3(3)
C12	C11	N1	119.3(2)	C36	C37	C32	118.7(3)
C12	C11	C16	121.8(3)	C36	C37	C38	116.0(3)
C16	C11	N1	118.9(2)	C37	C38	Ru1	130.3(2)
C11	C12	C17	120.9(3)	C41	C40	N2	123.0(5)
C13	C12	C11	117.9(3)	C41A	C40A	N2	125.8(10)
C13	C12	C17	121.2(3)	N2	C42	C43	114.1(3)
C12	C13	C14	122.0(3)	C3S	C2S	C1S	106.7(8)
C13	C14	C18	120.1(4)	C2S	C3S	C3S[1]	113.5(6)
C15	C14	C13	118.5(3)				

[1]-X,+Y,3/2-Z

Table S13. Hydrogen Bonds for **4c**

**D**	**H**	**A**	**d(D-H)/Å**	**d(H-A)/Å**	**d(D-A)/Å**	**D-H-A/°**
C4	H4A	Cl2[1]	0.99	2.79	3.587(3)	137.5
C5	H5B	Cl2[1]	0.99	2.88	3.631(3)	133.7
C31	H31A	Cl1	1.00	2.45	3.251(3)	137.0
C40A	H40C	Cl2	0.99	2.48	3.160(4)	125.8
C42	H42B	Cl1	0.99	2.85	3.372(3)	113.8

[1]1/2-X,-1/2+Y,3/2-Z

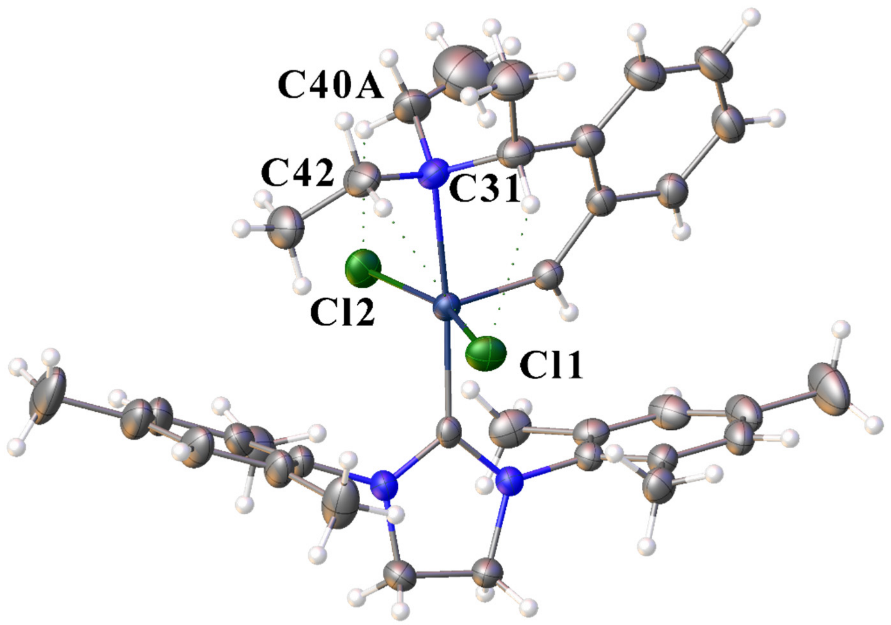

Figure S7. View showing intramolecular hydrogen bonds in **4c**. Only the main part of disordered C_2H_5 group (participating in H-bonding) is shown.

Table S14. Torsion Angles for **4c**

A	B	C	D	Angle/°	A	B	C	D	Angle/°
Ru1	N2	C31	C32	69.5(2)	C17	C12	C13	C14	179.4(3)
Ru1	N2	C31	C39	-159.5(2)	C18	C14	C15	C16	-179.0(3)
Ru1	N2	C40	C41	-168.2(5)	C21	N3	C2	Ru1	16.3(4)
Ru1	N2	C40A	C41A	-75.8(12)	C21	N3	C2	N1	-170.2(3)
Ru1	N2	C42	C43	-45.3(3)	C21	N3	C4	C5	170.5(3)
Cl1	Ru1	C38	C37	-73.1(3)	C21	C22	C23	C24	0.0(6)
Cl2	Ru1	C38	C37	105.5(3)	C22	C21	C26	C25	6.8(5)
N1	C11	C12	C13	178.1(3)	C22	C21	C26	C29	-168.8(3)
N1	C11	C12	C17	-2.3(4)	C22	C23	C24	C25	3.0(6)
N1	C11	C16	C15	-177.8(3)	C22	C23	C24	C28	-179.6(4)
N1	C11	C16	C19	4.8(4)	C23	C24	C25	C26	-1.2(6)
N2	Ru1	C38	C37	14.9(3)	C24	C25	C26	C21	-3.6(5)
N2	C31	C32	C33	136.2(3)	C24	C25	C26	C29	172.1(3)
N2	C31	C32	C37	-47.0(4)	C26	C21	C22	C23	-5.1(5)
N3	C4	C5	N1	1.0(3)	C26	C21	C22	C27	172.0(3)
N3	C21	C22	C23	-178.1(3)	C27	C22	C23	C24	-177.2(4)
N3	C21	C22	C27	-1.0(5)	C28	C24	C25	C26	-178.5(4)
N3	C21	C26	C25	180.0(3)	C31	N2	C40	C41	-45.7(6)
N3	C21	C26	C29	4.3(4)	C31	N2	C40A	C41A	46.7(12)
C2	Ru1	C38	C37	-161.1(3)	C31	N2	C42	C43	-161.1(3)
C2	N1	C5	C4	-0.9(3)	C31	C32	C33	C34	176.3(3)
C2	N1	C11	C12	96.0(3)	C31	C32	C37	C36	-175.5(3)
C2	N1	C11	C16	-87.3(4)	C31	C32	C37	C38	3.1(4)
C2	N3	C4	C5	-0.8(3)	C32	C33	C34	C35	-0.8(5)
C2	N3	C21	C22	-104.5(4)	C32	C37	C38	Ru1	7.0(4)
C2	N3	C21	C26	82.2(4)	C33	C32	C37	C36	1.4(4)
C4	N3	C2	Ru1	-173.2(2)	C33	C32	C37	C38	-179.9(3)
C4	N3	C2	N1	0.3(3)	C33	C34	C35	C36	1.4(5)
C4	N3	C21	C22	85.4(4)	C34	C35	C36	C37	-0.6(5)
C4	N3	C21	C26	-87.9(3)	C35	C36	C37	C32	-0.8(4)
C5	N1	C2	Ru1	172.9(2)	C35	C36	C37	C38	-179.6(3)
C5	N1	C2	N3	0.5(3)	C36	C37	C38	Ru1	-174.3(2)
C5	N1	C11	C12	-87.5(3)	C37	C32	C33	C34	-0.6(5)
C5	N1	C11	C16	89.2(3)	C39	C31	C32	C33	3.9(4)
C11	N1	C2	Ru1	-10.5(4)	C39	C31	C32	C37	-179.2(3)
C11	N1	C2	N3	177.1(3)	C40	N2	C31	C32	-56.8(3)
C11	N1	C5	C4	-178.0(2)	C40	N2	C31	C39	74.2(4)

C11	C12	C13	C14	-1.0(5)	C40	N2	C42	C43	77.5(3)
C12	C11	C16	C15	-1.1(4)	C40A	N2	C31	C32	-56.8(3)
C12	C11	C16	C19	-178.6(3)	C40A	N2	C31	C39	74.2(4)
C12	C13	C14	C15	0.3(5)	C40A	N2	C42	C43	77.5(3)
C12	C13	C14	C18	179.4(3)	C42	N2	C31	C32	-176.6(2)
C13	C14	C15	C16	0.1(5)	C42	N2	C31	C39	-45.6(4)
C14	C15	C16	C11	0.3(5)	C42	N2	C40	C41	75.0(6)
C14	C15	C16	C19	177.8(3)	C42	N2	C40A	C41A	167.4(11)
C16	C11	C12	C13	1.5(4)	C1S	C2S	C3S	C3S[1]	173.6(7)
C16	C11	C12	C17	-178.9(3)					

[1]-X,+Y,3/2-Z

Table S15. Bond Lengths for **5a**

Atom	Atom	Length/Å	Atom	Atom	Length/Å
Ru1	Cl1	2.3666(4)	C15	C16	1.405(2)
Ru1	Cl2	2.3604(4)	C16	C19	1.505(2)
Ru1	N2	2.2517(14)	C21	C22	1.401(3)
Ru1	C2	2.0401(16)	C21	C26	1.404(2)
Ru1	C38	1.8281(16)	C22	C23	1.393(3)
N1	C2	1.351(2)	C22	C27	1.512(2)
N1	C5	1.479(2)	C23	C24	1.393(3)
N1	C11	1.436(2)	C24	C25	1.391(3)
N2	C31	1.506(2)	C24	C28	1.516(3)
N2	C41	1.485(2)	C25	C26	1.401(3)
N2	C42	1.489(2)	C26	C29	1.506(3)
N3	C2	1.358(2)	C31	C32	1.526(2)
N3	C4	1.474(2)	C31	C39	1.539(2)
N3	C21	1.444(2)	C32	C33	1.396(2)
C4	C5	1.533(2)	C32	C37	1.422(2)
C11	C12	1.404(2)	C33	C34	1.391(3)
C11	C16	1.393(2)	C34	C35	1.383(3)
C12	C13	1.395(2)	C35	C36	1.386(2)
C12	C17	1.507(2)	C36	C37	1.411(2)
C13	C14	1.387(3)	C37	C38	1.468(2)
C14	C15	1.391(3)	C39	C40	1.531(3)
C14	C18	1.515(3)			

Table S16. Bond Angles for **5a**

Atom	Atom	Atom	Angle/˚	Atom	Atom	Atom	Angle/˚
Cl2	Ru1	Cl1	161.542(16)	C13	C14	C18	119.95(18)

N2	Ru1	Cl1	90.02(4)	C15	C14	C18	121.37(19)
N2	Ru1	Cl2	87.99(4)	C14	C15	C16	121.81(17)
C2	Ru1	Cl1	85.37(4)	C11	C16	C15	117.64(16)
C2	Ru1	Cl2	93.60(4)	C11	C16	C19	121.15(16)
C2	Ru1	N2	170.06(6)	C15	C16	C19	121.20(17)
C38	Ru1	Cl1	98.73(5)	C22	C21	N3	119.03(15)
C38	Ru1	Cl2	99.54(5)	C22	C21	C26	121.25(16)
C38	Ru1	N2	88.19(6)	C26	C21	N3	119.58(16)
C38	Ru1	C2	101.20(7)	C21	C22	C27	122.40(16)
C2	N1	C5	114.01(13)	C23	C22	C21	118.12(17)
C2	N1	C11	126.66(14)	C23	C22	C27	119.39(17)
C11	N1	C5	118.03(13)	C22	C23	C24	122.09(19)
C31	N2	Ru1	109.91(10)	C23	C24	C28	120.8(2)
C41	N2	Ru1	117.33(10)	C25	C24	C23	118.20(18)
C41	N2	C31	109.94(13)	C25	C24	C28	121.0(2)
C41	N2	C42	108.19(13)	C24	C25	C26	121.87(18)
C42	N2	Ru1	98.96(10)	C21	C26	C29	122.00(16)
C42	N2	C31	112.10(13)	C25	C26	C21	117.97(18)
C2	N3	C4	114.28(13)	C25	C26	C29	119.93(17)
C2	N3	C21	124.69(14)	N2	C31	C32	109.76(13)
C21	N3	C4	120.70(14)	N2	C31	C39	113.14(14)
N1	C2	Ru1	132.71(12)	C32	C31	C39	115.16(15)
N1	C2	N3	106.62(14)	C33	C32	C31	120.21(15)
N3	C2	Ru1	119.98(11)	C33	C32	C37	117.68(15)
N3	C4	C5	102.30(13)	C37	C32	C31	122.06(15)
N1	C5	C4	102.63(13)	C34	C33	C32	122.59(16)
C12	C11	N1	118.15(15)	C35	C34	C33	119.82(17)
C16	C11	N1	119.69(15)	C34	C35	C36	119.11(17)
C16	C11	C12	122.08(15)	C35	C36	C37	122.02(16)
C11	C12	C17	121.30(15)	C32	C37	C38	125.89(15)
C13	C12	C11	117.91(16)	C36	C37	C32	118.77(15)
C13	C12	C17	120.77(16)	C36	C37	C38	115.33(14)
C14	C13	C12	121.87(17)	C37	C38	Ru1	129.10(12)
C13	C14	C15	118.68(16)	C40	C39	C31	112.92(16)

Table S17. Hydrogen Bonds for **5a**

D	H	A	d(D-H)/Å	d(H-A)/Å	d(D-A)/Å	D-H-A/°
C5	H5B	Cl2[1]	0.99	2.83	3.6133(18)	136.1
C31	H31A	Cl1	1.00	2.46	3.2483(18)	135.6
C41	H41B	Cl2	0.98	2.51	3.1389(18)	121.4

C42	H42A	Cl1	0.98	2.77	3.4341(19)	126.0

[1]1/2-X,1/2+Y,1/2-Z

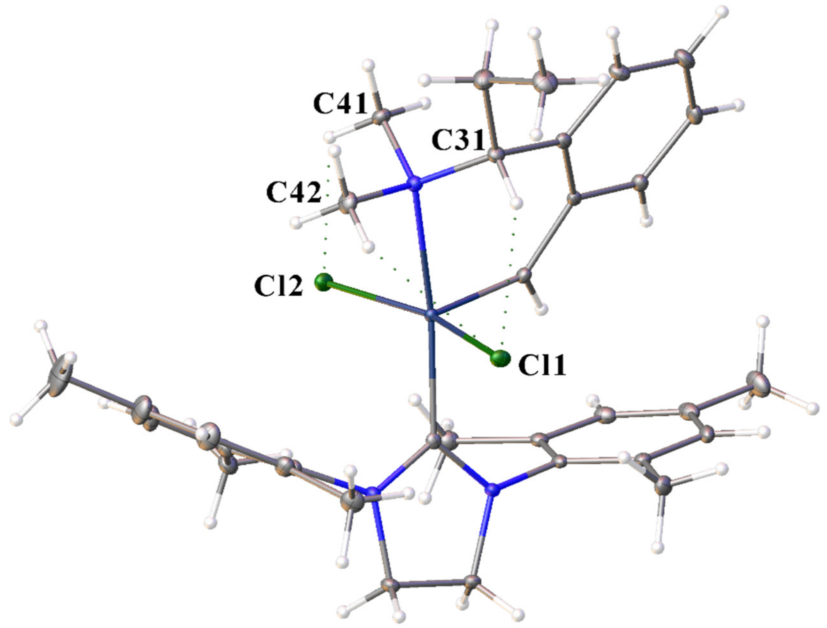

Figure S8. View showing intramolecular hydrogen bonds in **5a**.

Table S18. Torsion Angles for **5a**

A	B	C	D	Angle/°	A	B	C	D	Angle/°
Ru1	N2	C31	C32	67.73(14)	C14	C15	C16	C11	-0.6(3)
Ru1	N2	C31	C39	-162.12(12)	C14	C15	C16	C19	178.61(17)
Cl1	Ru1	C38	C37	-71.23(15)	C16	C11	C12	C13	-0.7(2)
Cl2	Ru1	C38	C37	106.16(15)	C16	C11	C12	C17	-179.28(16)
N1	C11	C12	C13	-177.48(14)	C17	C12	C13	C14	178.33(16)
N1	C11	C12	C17	4.0(2)	C18	C14	C15	C16	179.96(17)
N1	C11	C16	C15	177.80(15)	C21	N3	C2	Ru1	16.3(2)
N1	C11	C16	C19	-1.4(2)	C21	N3	C2	N1	-172.07(15)
N2	Ru1	C38	C37	18.51(15)	C21	N3	C4	C5	170.29(14)
N2	C31	C32	C33	140.81(16)	C21	C22	C23	C24	-2.6(3)
N2	C31	C32	C37	-41.8(2)	C22	C21	C26	C25	-7.2(3)
N2	C31	C39	C40	146.26(16)	C22	C21	C26	C29	169.14(18)
N3	C4	C5	N1	3.84(16)	C22	C23	C24	C25	-2.8(4)
N3	C21	C22	C23	-176.47(17)	C22	C23	C24	C28	179.1(2)
N3	C21	C22	C27	7.0(3)	C23	C24	C25	C26	3.4(4)
N3	C21	C26	C25	177.08(17)	C24	C25	C26	C21	1.5(3)
N3	C21	C26	C29	-6.6(3)	C24	C25	C26	C29	-174.9(2)
C2	Ru1	C38	C37	-158.20(15)	C26	C21	C22	C23	7.7(3)
C2	N1	C5	C4	-3.53(18)	C26	C21	C22	C27	-168.83(17)

C2	N1	C11	C12	-78.1(2)	C27	C22	C23	C24	174.0(2)
C2	N1	C11	C16	105.04(19)	C28	C24	C25	C26	-178.5(2)
C2	N3	C4	C5	-3.46(18)	C31	C32	C33	C34	177.03(17)
C2	N3	C21	C22	83.2(2)	C31	C32	C37	C36	-178.02(15)
C2	N3	C21	C26	-100.9(2)	C31	C32	C37	C38	0.8(3)
C4	N3	C2	Ru1	-170.24(11)	C32	C31	C39	C40	-86.4(2)
C4	N3	C2	N1	1.39(18)	C32	C33	C34	C35	1.2(3)
C4	N3	C21	C22	-89.9(2)	C32	C37	C38	Ru1	4.2(3)
C4	N3	C21	C26	86.0(2)	C33	C32	C37	C36	-0.6(2)
C5	N1	C2	Ru1	171.62(12)	C33	C32	C37	C38	178.26(16)
C5	N1	C2	N3	1.50(18)	C33	C34	C35	C36	-0.9(3)
C5	N1	C11	C12	88.03(18)	C34	C35	C36	C37	-0.2(3)
C5	N1	C11	C16	-88.79(19)	C35	C36	C37	C32	0.9(3)
C11	N1	C2	Ru1	-21.7(2)	C35	C36	C37	C38	-178.05(16)
C11	N1	C2	N3	168.15(15)	C36	C37	C38	Ru1	-176.89(13)
C11	N1	C5	C4	-171.41(14)	C37	C32	C33	C34	-0.5(3)
C11	C12	C13	C14	-0.2(3)	C39	C31	C32	C33	11.8(2)
C12	C11	C16	C15	1.1(2)	C39	C31	C32	C37	-170.88(16)
C12	C11	C16	C19	-178.06(16)	C41	N2	C31	C32	-62.87(17)
C12	C13	C14	C15	0.7(3)	C41	N2	C31	C39	67.28(18)
C12	C13	C14	C18	-179.55(17)	C42	N2	C31	C32	176.75(14)
C13	C14	C15	C16	-0.3(3)	C42	N2	C31	C39	-53.10(19)

Table S19. Bond Lengths for **5b**

Atom	Atom	Length/Å	Atom	Atom	Length/Å
Ru1	Cl1	2.3594(7)	C15	C16	1.403(4)
Ru1	Cl2	2.3499(7)	C16	C19	1.513(4)
Ru1	N2	2.272(2)	C21	C22	1.409(4)
Ru1	C2	2.059(3)	C21	C26	1.402(4)
Ru1	C38	1.829(3)	C22	C23	1.390(4)
N1	C2	1.350(3)	C22	C27	1.508(4)
N1	C5	1.481(3)	C23	C24	1.395(4)
N1	C11	1.442(3)	C24	C25	1.390(4)
N2	C31	1.520(4)	C24	C28	1.512(4)
N2	C41	1.491(4)	C25	C26	1.397(4)
N2	C42	1.506(4)	C26	C29	1.515(4)
N3	C2	1.359(3)	C31	C32	1.524(4)
N3	C4	1.476(3)	C31	C39	1.533(4)
N3	C21	1.441(3)	C32	C33	1.396(4)
C4	C5	1.522(4)	C32	C37	1.424(4)

C11	C12	1.396(4)	C33	C34	1.390(4)
C11	C16	1.400(4)	C34	C35	1.380(5)
C12	C13	1.400(4)	C35	C36	1.386(4)
C12	C17	1.502(4)	C36	C37	1.412(4)
C13	C14	1.400(4)	C37	C38	1.463(4)
C14	C15	1.395(4)	C39	C40	1.535(5)
C14	C18	1.515(4)	C42	C43	1.523(4)

Table S20. Bond Angles for **5b**

Atom	Atom	Atom	Angle/°	Atom	Atom	Atom	Angle/°
Cl2	Ru1	Cl1	159.02(3)	C15	C14	C18	120.8(3)
N2	Ru1	Cl1	88.17(6)	C14	C15	C16	122.0(3)
N2	Ru1	Cl2	89.92(6)	C11	C16	C15	117.8(3)
C2	Ru1	Cl1	86.54(7)	C11	C16	C19	121.3(3)
C2	Ru1	Cl2	93.18(7)	C15	C16	C19	120.8(3)
C2	Ru1	N2	172.62(9)	C22	C21	N3	119.2(2)
C38	Ru1	Cl1	103.91(9)	C26	C21	N3	119.1(2)
C38	Ru1	Cl2	96.98(9)	C26	C21	C22	121.2(3)
C38	Ru1	N2	89.78(11)	C21	C22	C27	122.0(3)
C38	Ru1	C2	96.48(11)	C23	C22	C21	118.1(3)
C2	N1	C5	114.1(2)	C23	C22	C27	119.9(2)
C2	N1	C11	129.9(2)	C22	C23	C24	122.2(3)
C11	N1	C5	115.7(2)	C23	C24	C28	119.9(3)
C31	N2	Ru1	108.20(16)	C25	C24	C23	118.1(3)
C41	N2	Ru1	116.22(18)	C25	C24	C28	121.9(3)
C41	N2	C31	109.9(2)	C24	C25	C26	122.2(3)
C41	N2	C42	107.7(2)	C21	C26	C29	122.4(3)
C42	N2	Ru1	104.29(16)	C25	C26	C21	118.0(3)
C42	N2	C31	110.4(2)	C25	C26	C29	119.4(3)
C2	N3	C4	114.3(2)	N2	C31	C32	109.8(2)
C2	N3	C21	127.5(2)	N2	C31	C39	116.0(2)
C21	N3	C4	117.9(2)	C32	C31	C39	114.1(3)
N1	C2	Ru1	132.39(19)	C33	C32	C31	121.3(3)
N1	C2	N3	106.2(2)	C33	C32	C37	117.8(3)
N3	C2	Ru1	120.75(19)	C37	C32	C31	120.9(2)
N3	C4	C5	102.3(2)	C34	C33	C32	122.0(3)
N1	C5	C4	102.7(2)	C35	C34	C33	120.5(3)
C12	C11	N1	119.2(2)	C34	C35	C36	119.0(3)
C12	C11	C16	122.1(2)	C35	C36	C37	121.8(3)
C16	C11	N1	118.5(2)	C32	C37	C38	125.2(3)

C11	C12	C13	118.0(3)	C36	C37	C32	118.9(3)
C11	C12	C17	120.8(2)	C36	C37	C38	115.9(2)
C13	C12	C17	121.1(3)	C37	C38	Ru1	129.9(2)
C14	C13	C12	121.9(3)	C31	C39	C40	113.0(3)
C13	C14	C18	121.0(3)	N2	C42	C43	113.0(2)
C15	C14	C13	118.2(3)				

Table S21. Hydrogen Bonds for **5b**

D	H	A	d(D-H)/Å	d(H-A)/Å	d(D-A)/Å	D-H-A/°
C4	H4A	Cl2[1]	0.99	2.66	3.466(3)	138.9
C5	H5B	Cl2[1]	0.99	2.86	3.575(3)	130.1
C31	H31A	Cl1	1.00	2.54	3.325(3)	134.9
C41	H41B	Cl2	0.98	2.64	3.157(3)	113.4
C42	H42B	Cl1	0.99	2.78	3.304(3)	113.9
C43	H43A	Cl1[2]	0.98	2.75	3.716(3)	169.6

[1]1-X,1-Y,1/2+Z; [2]-1/2+X,3/2-Y,+Z

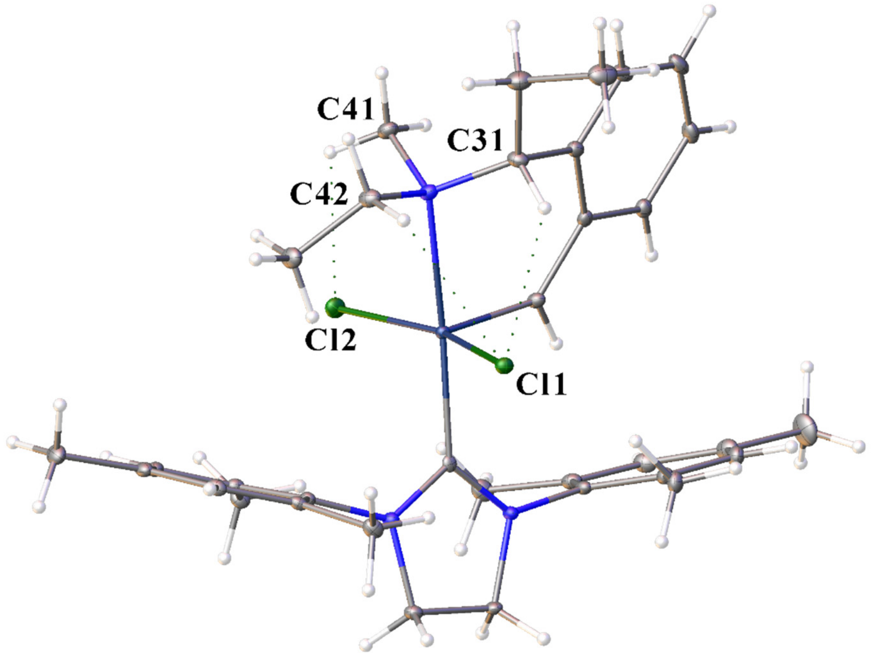

Figure S9. View showing intramolecular hydrogen bonds in **5b**.

Table S22. Torsion Angles for **5b**

A	B	C	D	Angle/˚	A	B	C	D	Angle/˚
Ru1	N2	C31	C32	69.3(2)	C14	C15	C16	C19	176.4(3)
Ru1	N2	C31	C39	-159.4(2)	C16	C11	C12	C13	0.7(4)
Ru1	N2	C42	C43	-44.6(3)	C16	C11	C12	C17	179.4(3)
Cl1	Ru1	C38	C37	-78.0(3)	C17	C12	C13	C14	-179.8(3)

Cl2	Ru1	C38	C37	99.9(3)	C18	C14	C15	C16	179.9(3)
N1	C11	C12	C13	176.5(2)	C21	N3	C2	Ru1	16.8(4)
N1	C11	C12	C17	-4.8(4)	C21	N3	C2	N1	-171.0(2)
N1	C11	C16	C15	-175.4(3)	C21	N3	C4	C5	168.8(2)
N1	C11	C16	C19	6.9(4)	C21	C22	C23	C24	1.1(4)
N2	Ru1	C38	C37	10.0(3)	C22	C21	C26	C25	6.0(4)
N2	C31	C32	C33	131.2(3)	C22	C21	C26	C29	-168.3(3)
N2	C31	C32	C37	-51.2(3)	C22	C23	C24	C25	1.4(4)
N2	C31	C39	C40	155.1(3)	C22	C23	C24	C28	178.8(3)
N3	C4	C5	N1	5.9(3)	C23	C24	C25	C26	-0.1(4)
N3	C21	C22	C23	-176.6(2)	C24	C25	C26	C21	-3.5(4)
N3	C21	C22	C27	0.2(4)	C24	C25	C26	C29	171.0(3)
N3	C21	C26	C25	177.8(2)	C26	C21	C22	C23	-4.8(4)
N3	C21	C26	C29	3.5(4)	C26	C21	C22	C27	172.0(3)
C2	Ru1	C38	C37	-166.0(3)	C27	C22	C23	C24	-175.8(3)
C2	N1	C5	C4	-4.8(3)	C28	C24	C25	C26	-177.6(3)
C2	N1	C11	C12	93.6(3)	C31	N2	C42	C43	-160.6(2)
C2	N1	C11	C16	-90.4(3)	C31	C32	C33	C34	176.8(3)
C2	N3	C4	C5	-6.0(3)	C31	C32	C37	C36	-175.5(3)
C2	N3	C21	C22	-106.1(3)	C31	C32	C37	C38	6.8(4)
C2	N3	C21	C26	81.9(3)	C32	C31	C39	C40	-75.7(4)
C4	N3	C2	Ru1	-168.97(18)	C32	C33	C34	C35	-1.0(5)
C4	N3	C2	N1	3.2(3)	C32	C37	C38	Ru1	9.3(4)
C4	N3	C21	C22	79.8(3)	C33	C32	C37	C36	2.1(4)
C4	N3	C21	C26	-92.1(3)	C33	C32	C37	C38	-175.6(3)
C5	N1	C2	Ru1	172.1(2)	C33	C34	C35	C36	1.6(5)
C5	N1	C2	N3	1.3(3)	C34	C35	C36	C37	-0.2(4)
C5	N1	C11	C12	-93.4(3)	C35	C36	C37	C32	-1.6(4)
C5	N1	C11	C16	82.6(3)	C35	C36	C37	C38	176.3(3)
C11	N1	C2	Ru1	-14.8(4)	C36	C37	C38	Ru1	-168.5(2)
C11	N1	C2	N3	174.4(2)	C37	C32	C33	C34	-0.9(4)
C11	N1	C5	C4	-179.0(2)	C39	C31	C32	C33	-1.1(4)
C11	C12	C13	C14	-1.1(4)	C39	C31	C32	C37	176.5(3)
C12	C11	C16	C15	0.5(4)	C41	N2	C31	C32	-58.5(3)
C12	C11	C16	C19	-177.3(3)	C41	N2	C31	C39	72.8(3)
C12	C13	C14	C15	0.2(5)	C41	N2	C42	C43	79.4(3)
C12	C13	C14	C18	-178.6(3)	C42	N2	C31	C32	-177.1(2)
C13	C14	C15	C16	1.0(5)	C42	N2	C31	C39	-45.8(3)
C14	C15	C16	C11	-1.4(5)					

Table S23. Bond Lengths for **5d**

Atom	Atom	Length/Å	Atom	Atom	Length/Å
Ru1	Cl1	2.363(3)	Ru1A	Cl1A	2.362(3)
Ru1	Cl2	2.395(3)	Ru1A	Cl2A	2.389(3)
Ru1	N2	2.371(10)	Ru1A	N2A	2.358(11)
Ru1	C2	2.005(12)	Ru1A	C2A	2.030(11)
Ru1	C38	1.808(12)	Ru1A	C38A	1.841(12)
O1	C42	1.430(14)	O1A	C42A	1.424(14)
O1	C43	1.442(14)	O1A	C43A	1.446(15)
N1	C2	1.370(14)	N1A	C2A	1.339(14)
N1	C5	1.470(14)	N1A	C5A	1.501(15)
N1	C11	1.427(15)	N1A	C11A	1.433(14)
N2	C31	1.500(14)	N2A	C31A	1.523(16)
N2	C41	1.502(14)	N2A	C41A	1.508(15)
N2	C44	1.519(16)	N2A	C44A	1.466(15)
N3	C2	1.378(14)	N3A	C2A	1.369(14)
N3	C4	1.487(15)	N3A	C4A	1.492(14)
N3	C21	1.425(15)	N3A	C21A	1.434(14)
C4	C5	1.513(17)	C4A	C5A	1.522(17)
C11	C12	1.394(17)	C11A	C12A	1.378(17)
C11	C16	1.420(16)	C11A	C16A	1.414(16)
C12	C13	1.393(18)	C12A	C13A	1.380(18)
C12	C17	1.518(16)	C12A	C17A	1.515(16)
C13	C14	1.380(18)	C13A	C14A	1.417(18)
C14	C15	1.360(17)	C14A	C15A	1.389(18)
C14	C18	1.530(18)	C14A	C18A	1.517(17)
C15	C16	1.396(16)	C15A	C16A	1.396(17)
C16	C19	1.511(16)	C16A	C19A	1.496(16)
C21	C22	1.417(16)	C21A	C22A	1.415(16)
C21	C26	1.422(16)	C21A	C26A	1.404(16)
C22	C23	1.383(16)	C22A	C23A	1.403(16)
C22	C27	1.504(16)	C22A	C27A	1.518(15)
C23	C24	1.381(16)	C23A	C24A	1.379(16)
C24	C25	1.417(16)	C24A	C25A	1.405(15)
C24	C28	1.496(16)	C24A	C28A	1.519(16)
C25	C26	1.372(16)	C25A	C26A	1.368(16)
C26	C29	1.505(17)	C26A	C29A	1.538(17)
C31	C32	1.524(16)	C31A	C32A	1.497(19)
C31	C39	1.529(18)	C31A	C39A	1.522(18)
C32	C33	1.371(18)	C32A	C33A	1.391(19)

C32	C37	1.382(17)	C32A	C37A	1.423(19)
C33	C34	1.397(19)	C33A	C34A	1.39(2)
C34	C35	1.384(19)	C34A	C35A	1.44(2)
C35	C36	1.391(19)	C35A	C36A	1.374(19)
C36	C37	1.416(17)	C36A	C37A	1.376(18)
C37	C38	1.479(18)	C37A	C38A	1.439(17)
C39	C40	1.547(18)	C39A	C40A	1.533(18)
C41	C42	1.520(16)	C41A	C42A	1.523(17)
C43	C44	1.469(17)	C43A	C44A	1.522(19)

Table S24. Bond Angles for **5d**

**Atom**	**Atom**	**Atom**	**Angle/˚**	**Atom**	**Atom**	**Atom**	**Angle/˚**
Cl1	Ru1	Cl2	167.71(11)	Cl1A	Ru1A	Cl2A	168.22(11)
Cl1	Ru1	N2	88.5(2)	N2A	Ru1A	Cl1A	88.5(2)
N2	Ru1	Cl2	92.7(2)	N2A	Ru1A	Cl2A	93.0(2)
C2	Ru1	Cl1	91.3(3)	C2A	Ru1A	Cl1A	90.0(3)
C2	Ru1	Cl2	85.5(3)	C2A	Ru1A	Cl2A	86.7(3)
C2	Ru1	N2	170.5(4)	C2A	Ru1A	N2A	170.9(4)
C38	Ru1	Cl1	98.4(4)	C38A	Ru1A	Cl1A	97.7(4)
C38	Ru1	Cl2	93.8(4)	C38A	Ru1A	Cl2A	94.0(4)
C38	Ru1	N2	90.1(5)	C38A	Ru1A	N2A	91.7(5)
C38	Ru1	C2	99.3(5)	C38A	Ru1A	C2A	97.5(5)
C42	O1	C43	107.8(9)	C42A	O1A	C43A	107.7(9)
C2	N1	C5	113.7(10)	C2A	N1A	C5A	112.6(9)
C2	N1	C11	127.2(9)	C2A	N1A	C11A	129.6(10)
C11	N1	C5	117.2(9)	C11A	N1A	C5A	117.4(9)
C31	N2	Ru1	110.9(7)	C31A	N2A	Ru1A	110.4(7)
C31	N2	C41	108.7(8)	C41A	N2A	Ru1A	114.7(7)
C31	N2	C44	106.6(9)	C41A	N2A	C31A	108.4(9)
C41	N2	Ru1	113.4(7)	C44A	N2A	Ru1A	110.6(8)
C41	N2	C44	106.9(9)	C44A	N2A	C31A	107.4(10)
C44	N2	Ru1	110.0(7)	C44A	N2A	C41A	105.0(10)
C2	N3	C4	113.3(10)	C2A	N3A	C4A	111.8(9)
C2	N3	C21	125.2(9)	C2A	N3A	C21A	127.3(9)
C21	N3	C4	120.7(9)	C21A	N3A	C4A	119.6(9)
N1	C2	Ru1	134.6(8)	N1A	C2A	Ru1A	134.1(9)
N1	C2	N3	105.4(10)	N1A	C2A	N3A	108.4(9)
N3	C2	Ru1	119.4(8)	N3A	C2A	Ru1A	117.1(8)
N3	C4	C5	102.0(10)	N3A	C4A	C5A	102.8(9)
N1	C5	C4	103.0(10)	N1A	C5A	C4A	102.0(9)

C12	C11	N1	120.0(10)	C12A	C11A	N1A	119.3(10)
C12	C11	C16	121.5(11)	C12A	C11A	C16A	123.5(11)
C16	C11	N1	118.4(11)	C16A	C11A	N1A	117.0(10)
C11	C12	C17	120.5(12)	C11A	C12A	C13A	117.4(11)
C13	C12	C11	117.6(11)	C11A	C12A	C17A	121.6(11)
C13	C12	C17	122.0(12)	C13A	C12A	C17A	121.0(12)
C14	C13	C12	122.4(13)	C12A	C13A	C14A	121.9(12)
C13	C14	C18	121.5(13)	C13A	C14A	C18A	119.8(13)
C15	C14	C13	118.7(12)	C15A	C14A	C13A	118.5(11)
C15	C14	C18	119.7(12)	C15A	C14A	C18A	121.8(12)
C14	C15	C16	122.9(11)	C14A	C15A	C16A	121.5(11)
C11	C16	C19	121.6(11)	C11A	C16A	C19A	121.2(11)
C15	C16	C11	116.9(11)	C15A	C16A	C11A	117.0(11)
C15	C16	C19	121.5(10)	C15A	C16A	C19A	121.8(11)
C22	C21	N3	120.5(11)	C22A	C21A	N3A	120.9(10)
C22	C21	C26	121.7(11)	C26A	C21A	N3A	118.4(10)
C26	C21	N3	117.6(11)	C26A	C21A	C22A	120.5(11)
C21	C22	C27	122.0(10)	C21A	C22A	C27A	120.8(11)
C23	C22	C21	116.3(11)	C23A	C22A	C21A	117.3(10)
C23	C22	C27	121.6(10)	C23A	C22A	C27A	121.6(11)
C24	C23	C22	124.1(11)	C24A	C23A	C22A	122.3(11)
C23	C24	C25	116.9(11)	C23A	C24A	C25A	118.5(11)
C23	C24	C28	123.6(11)	C23A	C24A	C28A	121.1(11)
C25	C24	C28	119.4(11)	C25A	C24A	C28A	120.3(11)
C26	C25	C24	122.5(11)	C26A	C25A	C24A	121.1(11)
C21	C26	C29	122.2(11)	C21A	C26A	C29A	119.8(11)
C25	C26	C21	117.4(11)	C25A	C26A	C21A	119.8(11)
C25	C26	C29	120.3(11)	C25A	C26A	C29A	120.3(11)
N2	C31	C32	111.9(9)	N2A	C31A	C39A	114.7(11)
N2	C31	C39	114.0(10)	C32A	C31A	N2A	110.4(10)
C32	C31	C39	110.2(11)	C32A	C31A	C39A	111.4(12)
C33	C32	C31	118.8(12)	C33A	C32A	C31A	120.7(14)
C33	C32	C37	121.2(13)	C33A	C32A	C37A	118.1(13)
C37	C32	C31	119.8(11)	C37A	C32A	C31A	120.8(12)
C32	C33	C34	120.2(14)	C32A	C33A	C34A	122.7(15)
C35	C34	C33	119.9(14)	C33A	C34A	C35A	118.4(13)
C34	C35	C36	119.8(13)	C36A	C35A	C34A	118.0(14)
C35	C36	C37	120.2(13)	C35A	C36A	C37A	123.8(15)
C32	C37	C36	118.6(12)	C32A	C37A	C38A	122.4(12)
C32	C37	C38	122.5(11)	C36A	C37A	C32A	118.9(13)

C36	C37	C38	118.7(11)	C36A	C37A	C38A	118.4(12)
C37	C38	Ru1	124.1(9)	C37A	C38A	Ru1A	122.5(9)
C31	C39	C40	112.4(12)	C31A	C39A	C40A	115.4(12)
N2	C41	C42	112.0(9)	N2A	C41A	C42A	111.1(10)
O1	C42	C41	111.8(11)	O1A	C42A	C41A	111.3(11)
O1	C43	C44	113.3(10)	O1A	C43A	C44A	111.1(10)
C43	C44	N2	109.8(10)	N2A	C44A	C43A	112.1(11)

Table S25. Hydrogen Bonds for **5d**

**D**	**H**	**A**	**d(D-H)/Å**	**d(H-A)/Å**	**d(D-A)/Å**	**D-H-A/°**
C4	H4A	Cl1A[1]	0.99	2.73	3.375(12)	123.6
C5	H5B	Cl1A[1]	0.99	2.80	3.480(12)	126.3
C41	H41B	Cl1	0.99	2.58	3.232(12)	123.2
C43	H43B	Cl2	0.99	2.68	3.318(12)	122.0
C44	H44B	Cl2	0.99	2.61	3.194(13)	117.7
C4A	H4AA	Cl1[2]	0.99	2.76	3.478(12)	129.8
C5A	H5AB	Cl1[2]	0.99	2.82	3.547(12)	130.9
C41A	H41D	Cl1A	0.99	2.65	3.259(13)	119.9
C43A	H43D	Cl2A	0.99	2.70	3.326(14)	121.8
C44A	H44C	Cl2A	0.99	2.64	3.205(14)	116.6

[1]1-X,1-Y,1-Z; [2]1-X,2-Y,1-Z

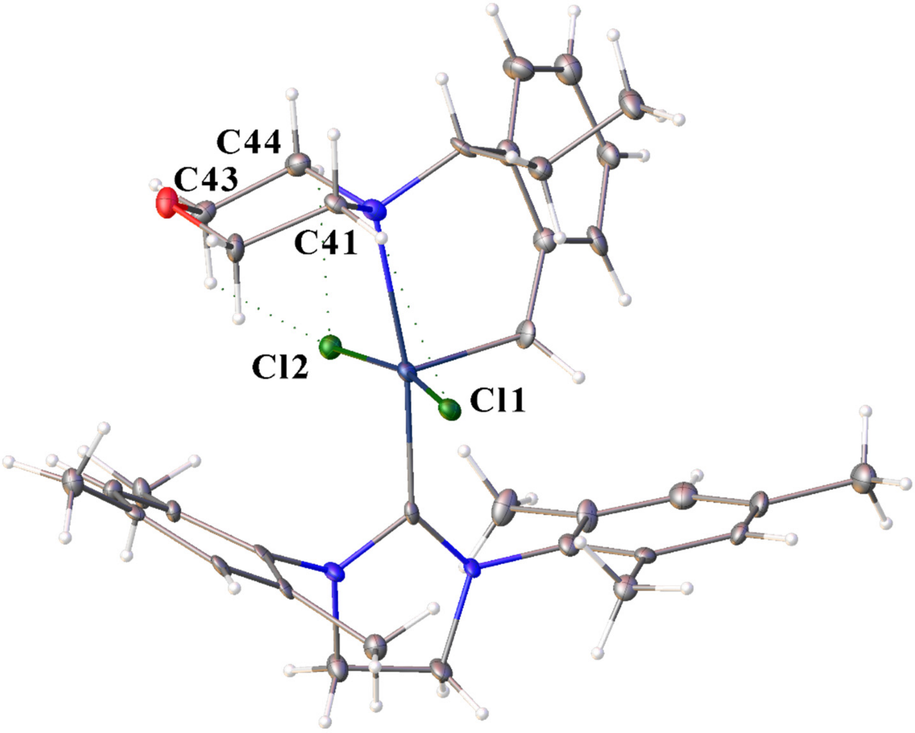

Figure S10. View showing intramolecular hydrogen bonds in **5d**. Only one independent molecule of the complex is shown.

Table S26. Torsion Angles for **5d**

A	B	C	D	Angle/°	A	B	C	D	Angle/°
Ru1	N2	C31	C32	52.7(11)	Ru1A	N2A	C31A	C32A	52.8(12)
Ru1	N2	C31	C39	-73.3(11)	Ru1A	N2A	C31A	C39A	-74.0(11)
Ru1	N2	C41	C42	-67.6(11)	Ru1A	N2A	C41A	C42A	-64.3(11)
Ru1	N2	C44	C43	68.2(11)	Ru1A	N2A	C44A	C43A	66.9(12)
Cl1	Ru1	C38	C37	-127.2(10)	Cl1A	Ru1A	C38A	C37A	-126.5(10)
Cl2	Ru1	C38	C37	54.1(10)	Cl2A	Ru1A	C38A	C37A	55.3(10)
O1	C43	C44	N2	61.9(13)	O1A	C43A	C44A	N2A	61.2(15)
N1	C11	C12	C13	-179.1(12)	N1A	C11A	C12A	C13A	-178.7(12)
N1	C11	C12	C17	0.7(18)	N1A	C11A	C12A	C17A	2.1(18)
N1	C11	C16	C15	179.2(10)	N1A	C11A	C16A	C15A	178.1(11)
N1	C11	C16	C19	-1.6(16)	N1A	C11A	C16A	C19A	-2.5(17)
N2	Ru1	C38	C37	-38.7(10)	N2A	Ru1A	C38A	C37A	-37.8(10)
N2	C31	C32	C33	121.6(13)	N2A	C31A	C32A	C33A	121.5(14)
N2	C31	C32	C37	-63.5(15)	N2A	C31A	C32A	C37A	-65.9(16)
N2	C31	C39	C40	179.0(10)	N2A	C31A	C39A	C40A	-177.5(11)
N2	C41	C42	O1	-57.5(13)	N2A	C41A	C42A	O1A	-61.6(13)
N3	C4	C5	N1	-15.0(13)	N3A	C4A	C5A	N1A	-14.8(12)
N3	C21	C22	C23	-176.0(10)	N3A	C21A	C22A	C23A	-178.8(10)
N3	C21	C22	C27	7.1(16)	N3A	C21A	C22A	C27A	7.9(17)
N3	C21	C26	C25	176.5(10)	N3A	C21A	C26A	C25A	178.7(10)
N3	C21	C26	C29	-4.1(15)	N3A	C21A	C26A	C29A	-5.3(15)
C2	Ru1	C38	C37	140.2(10)	C2A	Ru1A	C38A	C37A	142.5(10)
C2	N1	C5	C4	13.8(14)	C2A	N1A	C5A	C4A	14.1(13)
C2	N1	C11	C12	-105.5(14)	C2A	N1A	C11A	C12A	-102.1(14)
C2	N1	C11	C16	76.5(16)	C2A	N1A	C11A	C16A	82.6(15)
C2	N3	C4	C5	13.3(14)	C2A	N3A	C4A	C5A	12.6(13)
C2	N3	C21	C22	97.9(14)	C2A	N3A	C21A	C22A	99.8(13)
C2	N3	C21	C26	-87.3(14)	C2A	N3A	C21A	C26A	-84.7(14)
C4	N3	C2	Ru1	167.2(8)	C4A	N3A	C2A	Ru1A	169.0(8)
C4	N3	C2	N1	-5.2(14)	C4A	N3A	C2A	N1A	-4.2(12)
C4	N3	C21	C22	-92.7(14)	C4A	N3A	C21A	C22A	-94.6(14)
C4	N3	C21	C26	82.1(14)	C4A	N3A	C21A	C26A	80.9(12)
C5	N1	C2	Ru1	-176.4(9)	C5A	N1A	C2A	Ru1A	-178.2(8)
C5	N1	C2	N3	-5.8(13)	C5A	N1A	C2A	N3A	-6.6(12)
C5	N1	C11	C12	90.8(15)	C5A	N1A	C11A	C12A	85.6(14)

C5	N1	C11	C16	-87.2(13)	C5A	N1A	C11A	C16A	-89.7(13)
C11	N1	C2	Ru1	19.3(18)	C11A	N1A	C2A	Ru1A	9.2(17)
C11	N1	C2	N3	-170.0(11)	C11A	N1A	C2A	N3A	-179.2(10)
C11	N1	C5	C4	179.7(10)	C11A	N1A	C5A	C4A	-172.4(10)
C11	C12	C13	C14	0(2)	C11A	C12A	C13A	C14A	1(2)
C12	C11	C16	C15	1.3(17)	C12A	C11A	C16A	C15A	3.0(18)
C12	C11	C16	C19	-179.6(11)	C12A	C11A	C16A	C19A	-177.6(12)
C12	C13	C14	C15	0(2)	C12A	C13A	C14A	C15A	1(2)
C12	C13	C14	C18	-177.9(13)	C12A	C13A	C14A	C18A	-179.7(14)
C13	C14	C15	C16	0.1(19)	C13A	C14A	C15A	C16A	-2(2)
C14	C15	C16	C11	-0.8(17)	C14A	C15A	C16A	C11A	0.0(18)
C14	C15	C16	C19	-179.9(11)	C14A	C15A	C16A	C19A	-179.4(12)
C16	C11	C12	C13	-1.2(19)	C16A	C11A	C12A	C13A	-3.7(19)
C16	C11	C12	C17	178.6(12)	C16A	C11A	C12A	C17A	177.1(12)
C17	C12	C13	C14	-179.3(13)	C17A	C12A	C13A	C14A	-179.4(13)
C18	C14	C15	C16	178.1(12)	C18A	C14A	C15A	C16A	179.0(14)
C21	N3	C2	Ru1	-22.7(15)	C21A	N3A	C2A	Ru1A	-24.5(14)
C21	N3	C2	N1	164.9(11)	C21A	N3A	C2A	N1A	162.3(10)
C21	N3	C4	C5	-157.3(11)	C21A	N3A	C4A	C5A	-155.0(10)
C21	C22	C23	C24	-1.6(17)	C21A	C22A	C23A	C24A	-0.1(17)
C22	C21	C26	C25	-8.8(16)	C22A	C21A	C26A	C25A	-5.8(16)
C22	C21	C26	C29	170.6(11)	C22A	C21A	C26A	C29A	170.2(11)
C22	C23	C24	C25	-6.2(17)	C22A	C23A	C24A	C25A	-5.5(17)
C22	C23	C24	C28	174.6(12)	C22A	C23A	C24A	C28A	175.3(11)
C23	C24	C25	C26	6.8(16)	C23A	C24A	C25A	C26A	5.6(17)
C24	C25	C26	C21	0.4(16)	C24A	C25A	C26A	C21A	0.0(16)
C24	C25	C26	C29	-179.0(10)	C24A	C25A	C26A	C29A	-176.0(11)
C26	C21	C22	C23	9.4(16)	C26A	C21A	C22A	C23A	5.9(16)
C26	C21	C22	C27	-167.4(11)	C26A	C21A	C22A	C27A	-167.5(10)
C27	C22	C23	C24	175.2(11)	C27A	C22A	C23A	C24A	173.1(11)
C28	C24	C25	C26	-173.9(11)	C28A	C24A	C25A	C26A	-175.2(10)
C31	N2	C41	C42	168.5(10)	C31A	N2A	C41A	C42A	171.9(10)
C31	N2	C44	C43	-171.5(10)	C31A	N2A	C44A	C43A	-172.6(10)
C31	C32	C33	C34	173.9(13)	C31A	C32A	C33A	C34A	171.3(14)
C31	C32	C37	C36	-172.1(11)	C31A	C32A	C37A	C36A	-170.1(12)
C31	C32	C37	C38	12.3(18)	C31A	C32A	C37A	C38A	15(2)
C32	C31	C39	C40	52.1(14)	C32A	C31A	C39A	C40A	56.2(15)
C32	C33	C34	C35	-1(2)	C32A	C33A	C34A	C35A	0(2)
C32	C37	C38	Ru1	45.4(16)	C32A	C37A	C38A	Ru1A	43.1(16)
C33	C32	C37	C36	2.8(19)	C33A	C32A	C37A	C36A	3(2)

C33	C32	C37	C38	-172.9(13)	C33A	C32A	C37A	C38A	-172.2(13)
C33	C34	C35	C36	2(2)	C33A	C34A	C35A	C36A	1(2)
C34	C35	C36	C37	0.0(19)	C34A	C35A	C36A	C37A	1(2)
C35	C36	C37	C32	-2.2(19)	C35A	C36A	C37A	C32A	-2(2)
C35	C36	C37	C38	173.6(12)	C35A	C36A	C37A	C38A	172.7(12)
C36	C37	C38	Ru1	-130.2(11)	C36A	C37A	C38A	Ru1A	-131.8(11)
C37	C32	C33	C34	-1(2)	C37A	C32A	C33A	C34A	-2(2)
C39	C31	C32	C33	-110.4(14)	C39A	C31A	C32A	C33A	-109.8(16)
C39	C31	C32	C37	64.6(14)	C39A	C31A	C32A	C37A	62.7(16)
C41	N2	C31	C32	178.1(10)	C41A	N2A	C31A	C32A	179.2(11)
C41	N2	C31	C39	52.1(13)	C41A	N2A	C31A	C39A	52.4(14)
C41	N2	C44	C43	-55.3(12)	C41A	N2A	C44A	C43A	-57.4(13)
C42	O1	C43	C44	-61.7(13)	C42A	O1A	C43A	C44A	-59.2(14)
C43	O1	C42	C41	57.7(13)	C43A	O1A	C42A	C41A	59.9(13)
C44	N2	C31	C32	-67.0(13)	C44A	N2A	C31A	C32A	-67.8(14)
C44	N2	C31	C39	167.0(10)	C44A	N2A	C31A	C39A	165.3(11)
C44	N2	C41	C42	53.8(12)	C44A	N2A	C41A	C42A	57.3(13)

9. *References*

1. Perecim, G.P.; Rodrigues, A.; Raminelli, C. A Convenient Formation of Aporphine Core via Benzyne Chemistry: Conforma-tional Analysis and Synthesis of (*R*)-Aporphine. *Tetrahedron Lett.* **2015**, *56*, 6848–6851. https://doi.org/10.1016/j.tetlet.2015.10.083
2. Kingsbury, J.S.; Harrity, J.P.A.; Bonitatebus, P.J.; Hoveyda, A.H. A Recyclable Ru-Based Metathesis Catalyst. *J. Am. Chem. Soc.* **1999**, *121*, 791–799. https://doi.org/10.1021/ja983222u.
3. Bruker AXS Inc.: Madison, WI, USA. SAINT, V8.40B 2020 *Cif File Entries for the Purdue Quest Diffractometer (June 2020)*;
4. Krause, L.; Herbst-Irmer, R.; Sheldrick, G.M.; Stalke, D. Comparison of Silver and Molybdenum Microfocus X-Ray Sources for Single-Crystal Structure Determination. *J. Appl. Cryst.* **2015**, *48*, 3–10. https://doi.org/10.1107/S1600576714022985.
5. Sheldrick, G.M. *SHELXT* – Integrated Space-Group and Crystal-Structure Determination. *Acta Cryst.* **2015**, *A71*, 3–8. https://doi.org/10.1107/S2053273314026370.
6. Sheldrick, G.M. Crystal Structure Refinement with *SHELXL*. *Acta Cryst.* **2015**, *C71*, 3–8. https://doi.org/10.1107/S2053229614024218.
7. Spek, A.L. *PLATON* SQUEEZE: A Tool for the Calculation of the Disordered Solvent Contribution to the Calculated Structure Factors. *Acta Cryst.* **2015**, *C71*, 9–18. https://doi.org/10.1107/S2053229614024929.
8. Dolomanov, O.V.; Bourhis, L.J.; Gildea, R.J.; Howard, J.A.K.; Puschmann, H. *OLEX2* : A Complete Structure Solution, Refinement and Analysis Program. *J. Appl. Cryst.* **2009**, *42*, 339–341. https://doi.org/10.1107/S0021889808042726.

10. *Copies of NMR, FTIR and mass spectra for compounds 1−5*
